# Transcriptomic profiling of linolenic acid-responsive genes in ROS signaling from RNA-seq data in *Arabidopsis*

**DOI:** 10.3389/fpls.2015.00122

**Published:** 2015-03-17

**Authors:** Capilla Mata-Pérez, Beatriz Sánchez-Calvo, Juan C. Begara-Morales, Francisco Luque, Jaime Jiménez-Ruiz, María N. Padilla, Jesús Fierro-Risco, Raquel Valderrama, Ana Fernández-Ocaña, Francisco J. Corpas, Juan B. Barroso

**Affiliations:** ^1^Group of Biochemistry and Cell Signaling in Nitric Oxide, Department of Experimental Biology, Area of Biochemistry and Molecular Biology, University of JaénJaén, Spain; ^2^Department of Experimental Biology, Center for Advanced Studies in Olive Grove and Olive Oils, University of JaénJaén, Spain; ^3^Group of Antioxidants, Free Radicals and Nitric Oxide in Biotechnology, Food and Agriculture, Department of Biochemistry, Cellular and Molecular Biology of Plants, Estación Experimental del Zaidín, Consejo Superior de Investigaciones CientíficasGranada, Spain

**Keywords:** linolenic acid, *Arabidopsis*, massively parallel sequencing, RNA-seq, methionine sulfoxide reductase, oxidative stress, ROS signaling

## Abstract

Linolenic acid (Ln) released from chloroplast membrane galactolipids is a precursor of the phytohormone jasmonic acid (JA). The involvement of this hormone in different plant biological processes, such as responses to biotic stress conditions, has been extensively studied. However, the role of Ln in the regulation of gene expression during abiotic stress situations mediated by cellular redox changes and/or by oxidative stress processes remains poorly understood. An RNA-seq approach has increased our knowledge of the interplay among Ln, oxidative stress and ROS signaling that mediates abiotic stress conditions. Transcriptome analysis with the aid of RNA-seq in the absence of oxidative stress revealed that the incubation of *Arabidopsis thaliana* cell suspension cultures (ACSC) with Ln resulted in the modulation of 7525 genes, of which 3034 genes had a 2-fold-change, being 533 up- and 2501 down-regulated genes, respectively. Thus, RNA-seq data analysis showed that an important set of these genes were associated with the jasmonic acid biosynthetic pathway including lypoxygenases (*LOXs*) and Allene oxide cyclases (*AOCs*). In addition, several transcription factor families involved in the response to biotic stress conditions (pathogen attacks or herbivore feeding), such as *WRKY, JAZ, MYC*, and *LRR* were also modified in response to Ln. However, this study also shows that Ln has the capacity to modulate the expression of genes involved in the response to abiotic stress conditions, particularly those mediated by ROS signaling. In this regard, we were able to identify new targets such as galactinol synthase 1 (*GOLS1*), methionine sulfoxide reductase (*MSR*) and alkenal reductase in ACSC. It is therefore possible to suggest that, in the absence of any oxidative stress, Ln is capable of modulating new sets of genes involved in the signaling mechanism mediated by additional abiotic stresses (salinity, UV and high light intensity) and especially in stresses mediated by ROS.

## Introduction

The poly-unsaturated fatty acid α-Linolenic acid (Ln), with an 18-carbon chain and three *cis* double bonds, is an essential omega-3 fatty acid and organic compound found in seeds such as flaxseed, chia, soybean and various vegetable oils.

Ln, which can be released from several complex fatty acids mainly located in the membranes of organelles such as chloroplast is a precursor of the jasmonic acid (JA) phytohormone and, consequently, of the oxylipin pathway. It is generally accepted that phospholipase 1 (PLA_1_) is able to release Ln from the *sn1* position of galactolipids which is responsible for generating the JA substrate. The oxygenation of Ln is the initial step in JA biosynthesis which is carried out by plastid-located lipoxygenases (LOXs) at C-13 and is followed by the dehydration of 13-hydroperoxy-octadecatrienoic acid caused by allene oxide synthase (AOS). The unstable epoxide generated is then cyclized stereospecifically and converted into 12-oxo-phytodienoic acid (OPDA) by allene oxide cyclase (AOC) followed by the reduction of OPDA to 3-oxo-2-(2′(Z)-pentenyl)-cyclopentane-1 octanoic acid by the *Arabidopsis* OPDA reductase (OPR3). Finally, the subsequent shortening of the carboxylic acid side chain is caused by the fatty acid β –oxidation machinery and is initiated by Acyl-CoA-oxydase1 (ACX1) (Wasternack and Hause, 2013; Wasternack, 2014b).

The regulation of JA biosynthesis is determined by a positive feedback loop, substrate availability and tissue specificity (Wasternack, 2007). This phytohormone mediates several processes during plant growth such as male and female organ as well as embryo development, sex determination in maize, seed germination, seedling development, root growth, gravitropism, trichome and tuber formation, leaf movement and leaf senescence. Other processes mediated by JA include plant responses to desiccation, ozone, UV, osmotic, cold and light stresses as well as secondary metabolite formation and seasonal and circadian rhythm adaptations (Wasternack, 2014a).

Previously, the development of microarray technology enabled researchers to carry out large-scale studies and to analyze the response of thousands of genes in a single experiment. This technology has been used to analyze methyl-jasmonate-responsive genes in *Arabidopsis thaliana* cells (Pauwels et al., 2008) and to evaluate the role of different phytohormone-induced transcription factors in differential responses to damage caused by two different insect herbivores (Rehrig et al., 2014). These studies indicate that the application of jasmonate-derived molecules at an early stage prompted the expression of genes from the JA biosynthetic pathway and a later response in which a cellular metabolism is reprogrammed and cell cycle progression takes place, with the addition of a different response depending on the herbivore involved. Although most of these studies use jasmonate-derived molecules, to our knowledge, Ln, the principal component that engenders this pathway, has not been studied. Thus, jasmonate-responsive genes in *A. thaliana* have been identified with the aid of medium- and large-scale transcriptomic analyses using microarray technology (Pauwels et al., 2008). In recent years, new high-throughput sequencing methods, called massively parallel sequencing or RNA-seq, have emerged as a useful tool that could replace and improve upon existing methods given their advantages as compared to array-based methods (Wilhelm and Landry, 2009; Van Verk et al., 2013); (i) they do not depend on prior descriptions of the genomic sequence of the target species, making it easier to carry out gene expression studies of complex organisms; (ii) RNA-seq technology not only enables gene expression to be quantified but also facilitates the simultaneous identification of different isoforms, promoters, transcription start sites (TSS) and alternative splicing sites; (iii) RNA-seq is capable of detecting low-abundance transcripts; (iv) RNA-seq output is at the theoretical maximum of base pair resolution. As a result, RNA-seq data for higher plants have very recently started to be compiled (Lee et al., 2010; Li et al., 2012; De Cremer et al., 2013; Donà et al., 2013; Postnikova et al., 2013; Van Moerkercke et al., 2013).

The aim of the present study is to deepen our knowledge of how the linolenic acid precursor of jasmonic acid mediates new plant defense signaling pathways. The data indicate that Ln modulates the gene expression in the response to both biotic and abiotic stresses, which are especially mediated by reactive oxygen species (ROS). In this regard, a large-scale gene expression analysis has been carried out using paired-end RNA-seq technology developed by Illumina and is the first study of *Arabidopsis* to use this technique in order to increase our understanding of the Ln- signaling mechanism in the ROS metabolism. This technology has enabled us to gain an insight into transcriptional information and biological pathways that respond to Ln signaling which could not previously be identified by array-based methods applied to plants.

## Materials and methods

### Plant material, growth conditions, and treatments

*A. thaliana* cell suspension cultures (ACSC) were maintained in 200 ml liquid growth medium (Jouanneau and Péaud-Lenoël, 1967; Axelos et al., 1992) by gentle agitation at 120 rpm and 24°C under continuous 50 μE m^−2^ s^−1^ PAR (photosynthetically active radiation) illumination in an incubator shaker (Multitron Standard model, Infors HT). Cells were subcultured with a 1/20th dilution every 7 days. To analyze the involvement of linolenic acid (Ln) in the mechanism of gene expression regulation, 9-day-old ACSC were incubated with 1 mM Ln (which is equivalent to 10 μmol Ln/ g FW) and methanol (a fatty acid vehicle) and distilled water as controls. The treatments were applied under non-stress conditions. Due to ACSC growth in liquid medium, first step was the extraction by vacuum of liquid and pellets of cells were then harvested and used for RNA isolation. Samples were designated as control (C), vehicle (MeOH) and linolenic acid (Ln) ACSC.

### RNA sample preparation and high-throughput sequencing

Total RNA from pooled ACSC was obtained using Trizol Reagent (Gibco-BRL), as described in the manufacturer's manual. RNA was then purified using a Spectrum Plant Total RNA kit (Sigma-Aldrich, St Louis, MO, USA) according to the manufacturer's instructions. Any DNA contamination was removed by DNase I treatment on column (Roche, Basel, Switzerland). The RNA quality tests and concentrations were determined using a NanoVue™ Plus Spectrophotometer (GE Healthcare). RNA was pooled from each sample and complementary DNA (cDNA) libraries and sequencing in an Illumina HiSeq 1000 sequencer were generated by GeneSystems (Valencia, Spain). Two replicates of each sample were sequenced on different lanes in the flow cell.

### Bioinformatic analysis

Quality control of sequencing was carried out using FastQC software (V0.10.1). Gene-expression was studied using DNAStar (ArrayStar 4) Qseq software for RNA-seq analysis (www.dnastar.com) with the TAIR10 database as template. For mapping purposes, we used the *k*-*mer* = 63 and 95% of matches parameters and the reads per kilobase per million mapped reads (RPKM) default normalization method. The gene ontology (GO) terms were loaded in the Blast2GO suite V.2.7.2 (Conesa et al., 2005; Conesa and Götz, 2008) in order to statistically analyze GO-term enrichment. Blast2GO integrated the Gossip package for statistical assessment of differences in GO-term abundance between two sets of sequences (Blüthgen et al., 2004). This package uses Fisher's exact test and corrects for multiple testing. A one-tailed Fisher's exact test was carried out using a false discovery rate (FDR) with a filter value of <0.01. Blast2GO returns GO terms over-represented at a specified significance value (Conesa and Götz, 2008). The results were saved in a Microsoft Excel datasheet, and charts were generated.

For functional annotation purposes, genes studied showing significant expression-level changes in response to linolenic treatment were analyzed using DAVID (Dennis et al., 2003; Da Wei Huang and Lempicki, 2008) and TAIR (http://www.arabidopsis.org/tools/bulk/go/index.jsp) databases.

### Measurement of thiobarbituric acid reactive substances (TBARS)

TBARS were measured using the method described above (Stewart and Bewley, 1980) involving the spectrophotometric measurement of the pink pigment produced by the reaction of thiobarbituric acid (TBA) with malondialdehyde (MDA) and other secondary lipid peroxidation products. The evaluation of absorbance at 532 nm gives a measure of the extent of lipid degradation. ACSC treated with 1 mM Ln were homogenized with liquid nitrogen to obtain a fine powder and were mixed with a solution containing 918 mM trichloroacetic acid, 25.6 mM thiobarbituric acid and 250 mM HCl (ratio 1/5; w/v). The mixture was heated at 85°C for 30 min, the reaction was stopped by abrupt placement in an ice-bath and the cooled mixture was centrifuged at 10000 g for 10 min. Absorbance at 532 and 600 nm was measured and, in order to correct for background absorption, absorbance values at 600 nm were subtracted from those at 532 nm, with the latter representing the absorption maximum of the TBA: MDA adduct. A molar extinction coefficient of 156,000 (1.56 10^5^ M^−1^ cm^−1^) was used. All determinations were performed in triplicate and expressed in pmol MDA/g fresh weight.

### Spectrophotometric determination of hydrogen peroxide (H_2_O_2_)

Hydrogen peroxide (H_2_O_2_) content was measured as described by Jiang et al. (1990). Samples were incubated with a solution composed of 500 μM ammonium ferrous sulfate, 50 mM sulphuric acid, 200 μM xylenol orange and 200 mM sorbitol (ratio 1/2.5; w/v) in the dark for 45 min and then centrifuged at 2400 g for 10 min. Absorbance at 560 nm was measured and H_2_O_2_ concentration was estimated as H_2_O_2_ equivalent using a standard curve determined with commercial H_2_O_2_.

### Determination of protein carbonyl content

All procedures were performed at 0–4°C. Cell cultures were ground to a powder using a mortar with liquid nitrogen and were suspended in 100 mM Tris-HCl buffer, pH 7.5 (ratio 1/2; w/v) containing 5% (w/v) sucrose, 7% (w/v) PVPP, 0.05% Triton x-100, 0.1 mM EDTA, 1 mM PMSF and a commercial cocktail of protease inhibitors containing AEBSF, 1,10-phenantroline, pepstatine A, leupeptine, bestatine and E-64 (Sigma, St. Louis, MO, USA). Homogenates were filtered through one layer of Miracloth (Calbiochem, San Diego, CA, USA) and centrifuged at 3000 g for 10 min. A 2% (w/v) streptomycin sulfate solution was added to each sample in order to precipitate nucleic acids, which may cause an erroneously high estimate of protein-bound carbonyl, agitated for 20 min and then centrifuged at 2000 g for 10 min. A 10 mM DNPH solution in 2 N HCl was then added to the protein pellet of each sample, with 2 N HCl only being added to corresponding sample aliquot reagent blanks. Samples were allowed to stand in the dark at room temperature for 1 h and were then precipitated with 20% (w/v) TCA for 15 min and centrifuged at 5000 g for 10 min. Protein pellets were washed three times with ethanol/ethyl acetate (1:1, v/v) to remove any free DNPH. Samples were then resuspended in 6 M guanidine hydrochloride, dissolved in 20 mM phosphate buffer, pH 2.3, at 37°C for 15 min with vortex mixing. Carbonyl content was determined from absorbance at 370 nm using a molar absorption coefficient of 22,000 M^−1^ cm^−1^ for DNPH (Levine et al., 1990) and also by measuring protein content at 280 nm. Simultaneously, a BSA standard curve, dissolved in 6 M guanidine hydrochloride and incubated at 37°C for 15 min, was constructed.

### Fatty acid analysis of *Arabidopsis thaliana*

The *Arabidopsis* lipid fraction of cell suspension cultures was analyzed using a gas chromatograph (GC) (Agilent 7890A). The Meth-Prep II (Alltech Chemicals Cat. No. 18007) GC reagent was used for the transesterification of the *Arabidopsis* lipid fraction for gas chromatographic analysis purposes, and a standard oil mixture (Supelco ref. 18919-1AMP) was used to calibrate the gas chromatograph. Each sample was placed in a microvial and evaporated under a stream of nitrogen, the lipids were dissolved in 48 μl benzene and 50 μl Meth Prep II reagent was added. Following the derivatization stage, a GC/MS analysis was carried out by injecting a 1 μl solution. Analyses were carried out in a 7890A GC system (Agilent, USA) equipped with an SP-2560 capillary column (100 m × 0.25 mm × 0.25 μm) and a Quattro micro GC mass spectrometer (Waters, USA). The GC column procedure was as follows: initial temperature 140°C, maintained for 5 min, increased at 4°C min^−1^ to 250°C with a split ratio at injector port of 1:10.

### Quantitative real-time reverse transcriptase–PCR (qRT–PCR)

Total RNA from pooled of control, vehicle and linolenic acid ACSC was isolated as above, and first-strand cDNA was synthesized using the First Strand cDNA Synthesis kit (Roche) in a final volume of 20 μl according to the manufacturer's instructions. Real-time PCR was performed in a CFX384 real-time PCR Detection System (Bio-Rad). Amplifications were carried out in 5 μl of total volume containing 5 ng of cDNA, 2 μM of specific primers (see Supplemental Table 9) and SsoFast EvaGreen Supermix (Bio-Rad). PCR conditions used consisted of an initial denaturation at 98°C for 30 s, followed by 39 cycles at 98°C, 5 s and 60°C, 30 s. After cycling, melting curves of the reaction were run from 72°C to 82°C. Results were normalized using Actin12 (AT3G46520), 18S rRNA (AT2G01010), and L2 (AT2G44065) as internal controls.

### Data availability

The Illumina sequenced read data reported in this article have been deposited in the National Center for Biotechnology Information (NCBI) Sequence Read Archive and are available under the Accession Numbers Bioproject ID: PRJNA273982 and SRP Study Accession: SRP052987.

## Results

Fatty acid composition of *Arabidopsis* cell suspension cultures (ACSC) showed that linolenic acid (Ln) was the most abundant fatty acid, accounting for approximately 50% of total (0.134 μmol Ln/ g FW), followed by linoleic acid, with 20% of total (Supplemental Table 1), showing that these data were consistent with those obtained by Bonaventure et al. (2003). As Ln is the jasmonic acid precursor and the major fatty acid in ACSC and given that Ln-treatment did not cause oxidative damage in ACSC, it was therefore selected for this study.

In addition, ACSC treatment with 1 mM Ln for 1 h did not increase ROS content such as H_2_O_2_ or caused oxidative damage as lipid and protein oxidation markers such as malondialdehyde (MDA) and protein carbonyl content, respectively, were not affected (Supplemental Table 2).

### Transcriptomic analysis of Ln-responsive genes in ACSC

ACSC treated with 1 mM Ln (10 μmol Ln/ g FW) to observe a clear gene expression response due to Ln-treatment, methanol (vehicle) and distilled water (control) were harvested and used for total RNA isolation. Paired-end libraries were then prepared and sequenced as described in Material and Methods. Firstly, sequence quality was checked using the FastQC program and only reads with high quality values (≥Q30) were entered for mapping purposes (Phred values of 30 units or more which corresponds to a sequencing error rate of 0.1%). RNA-seq analysis using DNAstar QSeq mapped the reads to the *Arabidopsis* transcriptome database (TAIR10) with highly stringent parameters of kmer = 63 and 95% of matches.

**Figure 1:**
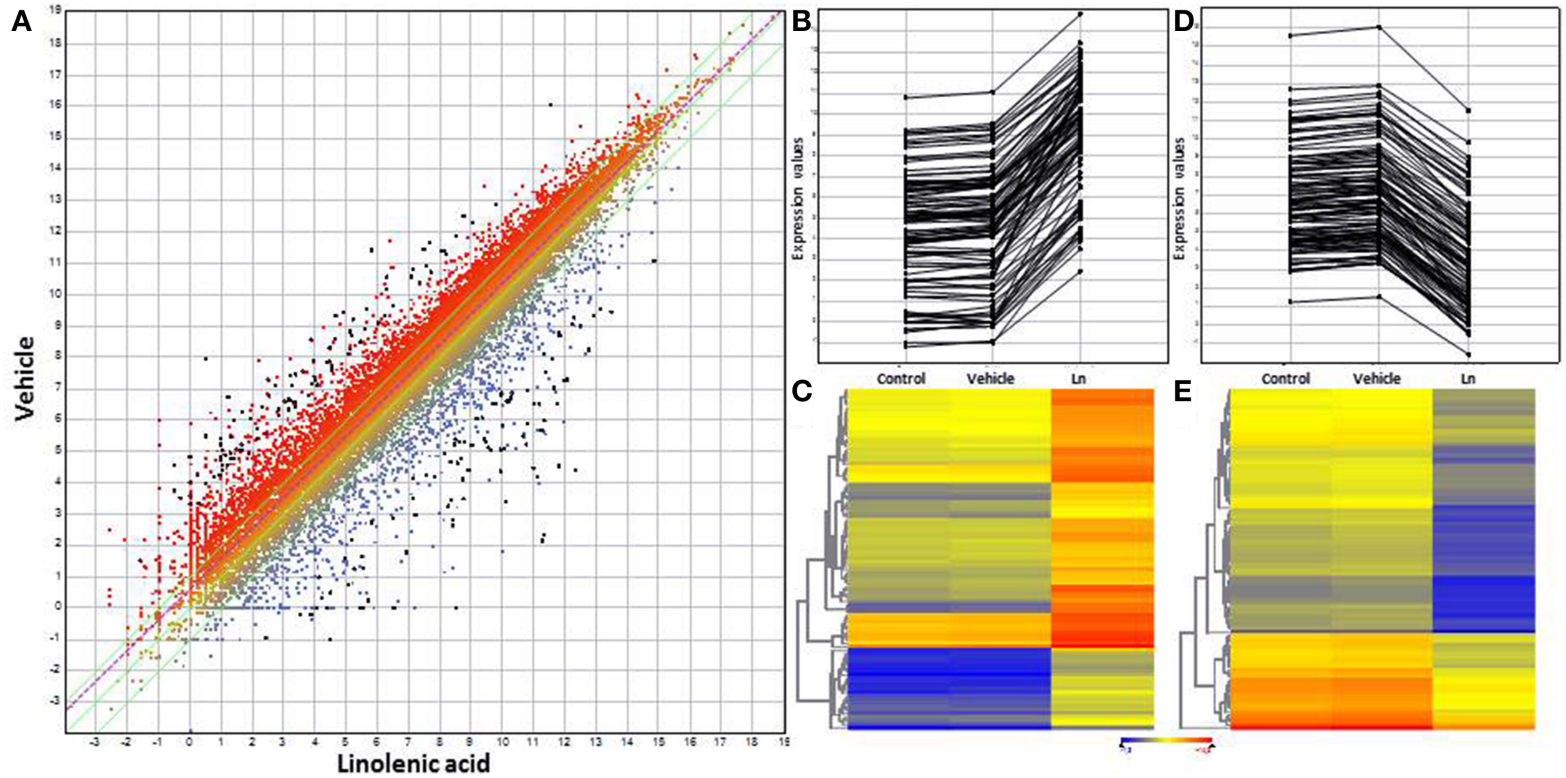
**Scatter plot, line plot and heat map for 8-fold-change genes using DNAStar software. (A)** Scatter plot of total Ln-responsive genes. **(B)** Line plot of overexpressed genes. **(C)** Heat map for overexpressed genes. **(D)** Line plot of repressed genes. **(E)** Heat map for repressed genes. Distilled water and methanol-responsive genes were used to filter the results of Ln treatment. All graphs show 8-fold-change genes with 95% significant differential expression obtained by t-student test from whole Ln-responsive genes.

The gene-expression profile of two ACSC groups was firstly compared, one treated with distilled water and the other with 1 mM Ln (control vs. Ln). Importantly, to eliminate genes that respond to methanol, this comparison was filtered using vehicle-responsive genes, with control vs. vehicle comparison eliminating genes with a 1.5 FC due to methanol, henceforth referred to as control. RNA-seq analysis showed that Ln caused significant changes (95% of matches, *p* < 0.05) in the gene-expression levels of 8947 ACSC genes, 7525 genes with annotations and 1422 genes coding for hypothetical proteins not considered for the rest of this study. From these 7525 genes, we selected 2FC up and down Ln-responsive genes showing the modulation of 3034 genes, from which 533 were up- (Supplemental Table 7) and 2501 were down-regulated (Supplemental Table 8). Due to this large number of genes, we only show the trend for genes differentially expressed with 8FC up and down (Figure 1). In this sense, Figure 1A shows the scatter plot of total genes whose expression changes significantly in response to Ln. Panels 1B and 1C represent the line plot and heat map of 8FC over-expressed genes and panels 1D and 1E indicate the line plot and heat map of 8FC repressed genes.

**Figure 2:**
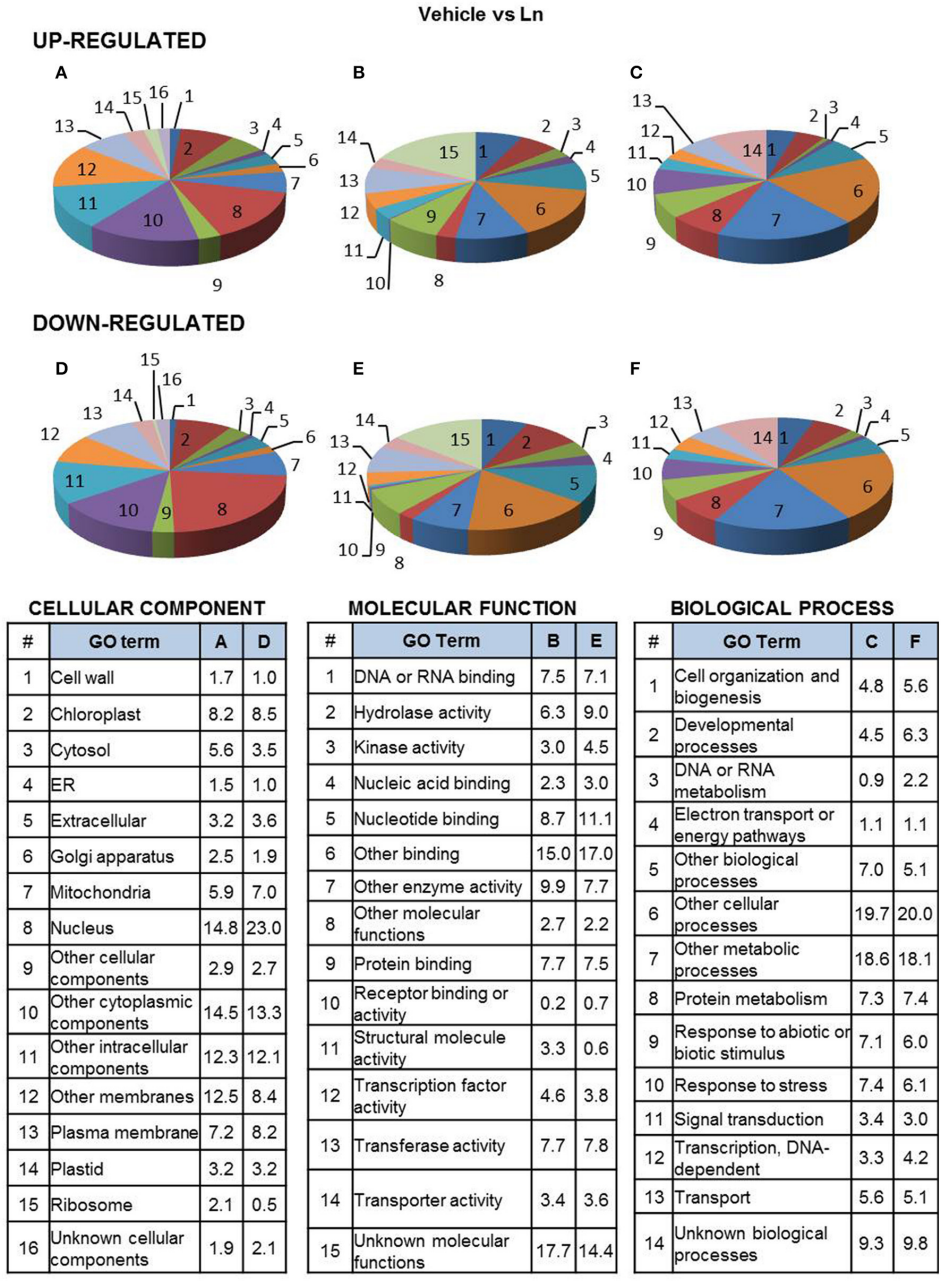
**Functional classification of Ln-responsive genes in ACSC**. Genes were classified by functional categories under the following gene ontology terms: cellular component **(A,D)**, molecular function **(B,E)**, biological process **(C,F)**, up-regulated genes **(A–C)** and down-regulated genes **(D–F)**. The number of genes assigned to each functional category is expressed as a percentage (%).

Functional classification of 2FC-Ln-induced genes shows that their products were mostly located in the nucleus, chloroplast and plasma membrane (Figure 2A). Furthermore, they were mostly characterized by nucleotide-, protein- and DNA/RNA-binding, transferase and hydrolase activity (Figure 2B). They were also predicted to be involved in the response to stress, biotic or abiotic stimuli and protein-metabolism processes (Figure 2C). On the other hand, the most abundant categories of 2FC-Ln-repressed genes in ACSC were also located in the nucleus, chloroplast, plasma membrane and mitochondria (Figure 2D). These genes showed hydrolase-, transferase-, nucleotide-, and protein-binding activity (Figure 2E) and were associated with the protein metabolism, developmental processes and stress responses (Figure 2F).

**Figure 3:**
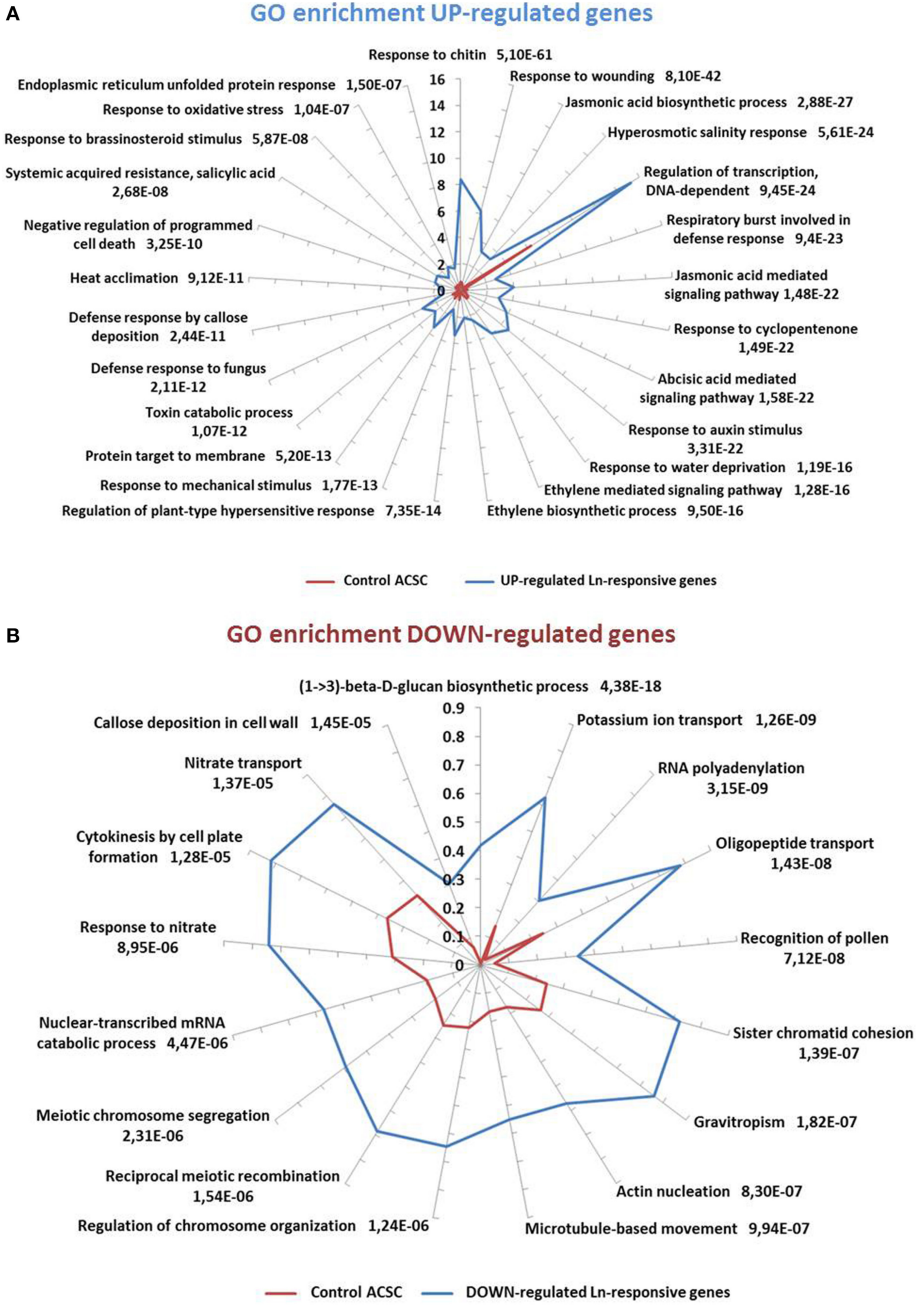
**GO-term-enriched graph of biological processes of Ln-responsive genes. (A)** Up-regulated genes. Node filter was set at FDR < 1e-5. **(B)** Down-regulated genes. Node filter was set at FDR < 0.001. Bars for up- and down-regulated genes are labeled with their corresponding *P*-values in Fisher's exact test against expressed control ACSC genes.

On the basis of these results, we decided to carry out a more detailed analysis of biological processes involved in Ln treatments. To do this, the GO terms obtained by the analysis were loaded in Blast2GO suite V.2.7.2 to statistically analyze GO-term enrichment. The unchecked two-tail box was used to analyze only positive enrichment. The test was carried out using a filter cut-off value of FDR < 1e^−5^ for up-regulated genes and an FDR < 0.001 for down-regulated genes. The results are shown in Figure 3 as the percentage of sequences annotated for each biological process GO term for both ACSC and Ln-responsive genes. Bars are labeled with their corresponding P-values in Fisher's exact test. Ln treatment of ACSC produced a significant response in the GO terms of up-regulated genes (Figure 3A). The GO terms of these genes were closely associated with biotic stress-related processes such as responses to chitin and wounding and were closely connected with biosynthesis and the JA signaling pathway. GO enrichment analysis also highlighted the over-representation of processes associated with other important phytohormones such as abscisic acid, auxin, ethylene, salicylic acid and brassinosteroids. With regard to abiotic stress, Ln treatment affected hyperosmotic salinity responses and heat acclimation, specifically in responses to oxidative stress through the response to hydrogen peroxide. With regard to the GO terms of down-regulated genes (Figure 3B), we found that the level of over-representation of biological processes was lower than in up-regulated genes. These processes were mostly associated with the synthesis of cell wall depicted by a (1-> 3)-beta-D-glucan biosynthetic process and callose deposition in cell wall together with the transport of potassium, oligopeptides and nitrate. Finally, mitotic- and meiotic-related processes such as sister chromatid cohesion, chromosome organization regulation, reciprocal meiotic recombination and meiotic chromosome segregation were observed.

### Linolenic acid-responsive genes involved in jasmonate- and biotic stress-related processes

Ln can be released from the plasma membrane by certain lipase enzymes in response to stress (Yang et al., 2012) and, for this reason, the regulation of JA-related genes in response to Ln treatment was predicted. Several genes involved in the JA biosynthetic pathway were induced: *DGL (AT1G05800), LOX4 (AT1G72520), LOX3 (AT1G17420), AOS (AT5G42650), AOC3 (AT3G25780), OPR3 (AT2G06050)*, and *OPCL1 (AT1G20510)* (see Supplemental Table 3). Furthermore, several *JAZ* genes encoding proteins that repress JA signaling and are targeted by E3-ubiquitin ligase SCF^*COI*1^ for proteasome degradation in response to JA (Chico et al., 2008), were up-regulated in response to Ln treatment. Among these, RNA-seq analysis showed an increase in the transcript levels of *JAZ10* (*AT5G13220*), *JAZ6* (*AT1G72450*), *JAZ5* (*AT1G17380*), *JAZ9* (*AT1G70700*), and *JAZ2* (*AT1G74950*). Two groups of induced transcriptional regulators of jasmonate biosynthesis and the JA-mediated signaling pathway were also detected. On the one hand, a set of activators were encoded mainly by *MYC2* (*AT1G32640*), a (bHLHzip)-type transcription factor (TF) that directly interacts with JAZ proteins, and *ORA47* (*AT1G74930*), the AP2/ERF protein postulated to be a positive regulator of JA biosynthesis (Pauwels et al., 2008). On the other hand, the repressors were encoded by *ZAT10* (*AT1G27730*) and *AZF2* (*AT3G19580*) that contain an ERF-associated amphiphilic repression domain and may act as both positive and negative regulators of plant defenses (Sakamoto et al., 2004; Mittler, 2006). In addition to the genes mentioned above, treatment of ACSC with Ln led to up-regulation of transcription factors from different families including ERF (*AT4G34410, AT1G28370, AT3G15210, AT5G47220*, and *AT3G23240*). *WRKY* (*AT1G80840, AT5G22570*), *bHLH* (*AT2G46510*) and *MYB* (Myeloblast) (*AT3G23250, AT3G06490, AT1G74430, AT1G57560, AT4G37260, AT2G16720, AT5G67300, AT3G28910, AT4G34990, AT4G38620*, and *AT5G37260*), mainly involved in the response to JA stimuli, and *NAC* (*AT5G08790, AT1G01720, AT1G52890, AT3G15500*, and *AT4G27410*) transcription factors were also found, as previously described (Pauwels et al., 2008). On the other hand, only a few genes playing a role in JA biosynthetic processes were down-regulated. *LOX5* (*AT3G22400*) activity in roots has been shown to facilitate green peach aphid colonization of *Arabidopsis* foliage (Nalam et al., 2012), indicating that the repression of this enzyme could act as an ACSC defense mechanism. Finally, in relation to the JA-mediated signaling pathway, Ln provoked gene down-regulation of carbonic anhydrase (CA) enzymes *AT5G14740* and *AT3G01500* and different *MYB* transcription factor families (*AT5G59780, AT2G36980, AT2G31180*, and *AT5G61420*) in response to JA stimuli.

The RNA-seq study shows that Ln provoked over-expression of *WRKY40* (*AT1G80840*) (see Supplemental Table 4) which was the most induced gene involved in defense responses, biotic stimulus and bacterium detection, negative defense response regulation and immune response regulation. WRKY40 is a pathogen-induced transcription factor that plays a positive role in JA-mediated defense (Xu et al., 2006), showing that Ln treatment launches a set of defense mechanisms against pathogen attacks. In this regard, *RRTF1* (*AT4G34410*), involved in responding to chitin and fungus and in respiratory burst as part of a defense response, was the most up-regulated gene in the RNA-seq analysis, with an FC of 496.97. With regard to pathogen attacks, Ln treatment led to the up-regulation of *BAG2* (*AT5G62100*) which belongs to the BAG (Bcl-2-associated athanogene) protein family and also with *JAS1/JAZ10* (*AT5G13220*), playing a role in systemic acquired resistance (SAR), being another highly up-regulated gene. With respect to Ln-repressed genes, a broad diversity of genes was associated with a variety of cellular processes such as *LRR* proteins *AT1G74360, AT1G51790, AT1G34420, AT1G35710*, and *AT4G08850*, which mainly respond to chitin and respiratory burst associated with defense responses. A large set of disease-resistant proteins were also down-regulated in response to Ln treatment, specifically in relation to defense responses and defense response signaling pathways (see Supplemental Table 4). This family of genes, of which Ln is a repressor, may be involved in responses to pathogens and abiotic stress (Rushton et al., 2010).

### Linolenic acid-responsive genes involved in abiotic stress situations

**Figure 4:**
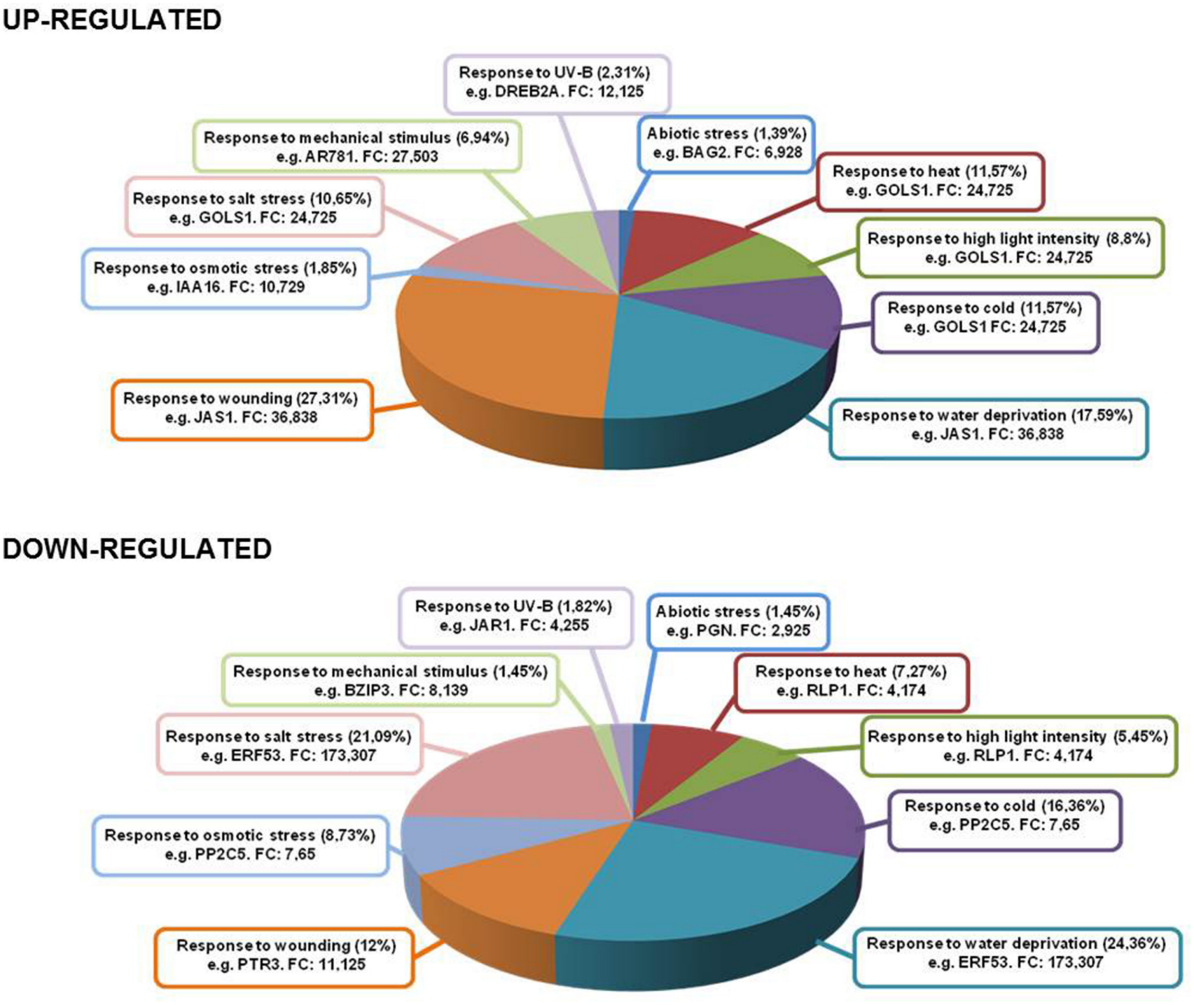
**Genes up- and down-regulated under several abiotic stress conditions**. Inside each box are depicted the type of stress and most representative gene in that category with its fold change.

The treatment of ACSC with 1 mM Ln provoked the over-expression of genes involved in different abiotic stress situations (Figure 4). The highest induction levels (*FC* = 24.725 up) were found in galactinol synthase (*GOLS1, AT2G47180*) which responds to heat, high light intensity, cold, salt stress and water deprivation (see Supplemental Table 5). Furthermore, different genes associated with heat responses also responded to Ln treatment such as heat stress transcription factors (*AT1G52560, AT2G26150, AT4G25200, AT2G20560, AT5G56030, AT2G32120, AT1G74310, AT5G37670, AT4G21320*, and *AT1G16030*) and to water deprivation as transcription factor *DREB2A* (*AT5G05410*) was highly induced. This gene has been shown to respond to dehydration and high salt stress (Liu et al., 1998). As is well known, Ln induces the jasmonate pathway involved in the response to biotic stress situations and wounding among other processes. An important set of over-expressed genes, accounting for 27.31% of the total, was involved in the response to wounding, with, as described previously, the most up-regulated gene being *JAS1/JAZ10* among other JAZ proteins. *JAZ* genes are rapidly induced by JA, suggesting the presence of a negative feedback loop to replenish the JAZ protein pool and to dampen the response to JA (Chini et al., 2007; Thines et al., 2007). A high percentage of the Ln-repressed genes were involved in the response to water deprivation and salt stress, accounting for 24.36 and 21.09% of down-regulated Ln-responsive genes, respectively. The highly repressed *AtERF53* (*AT2G20880*) is associated with drought stress responses (Cheng et al., 2012), while peptide transporter 3 (*AT5G46050*), involved in the response to wounding, was also repressed. Finally, another important group of down-regulated genes was activated in the response to cold and osmotic stress (16.36 and 8.73%, respectively), with the inhibition of PP2C5 (*AT2G40180*) acting as an MAPK phosphatase that controls MAPK levels and thus modulates innate immunity, JA and ethylene levels in *Arabidopsis* (Schweighofer et al., 2007).

### Linolenic acid-responsive genes in oxidative stress situations

**Figure 5:**
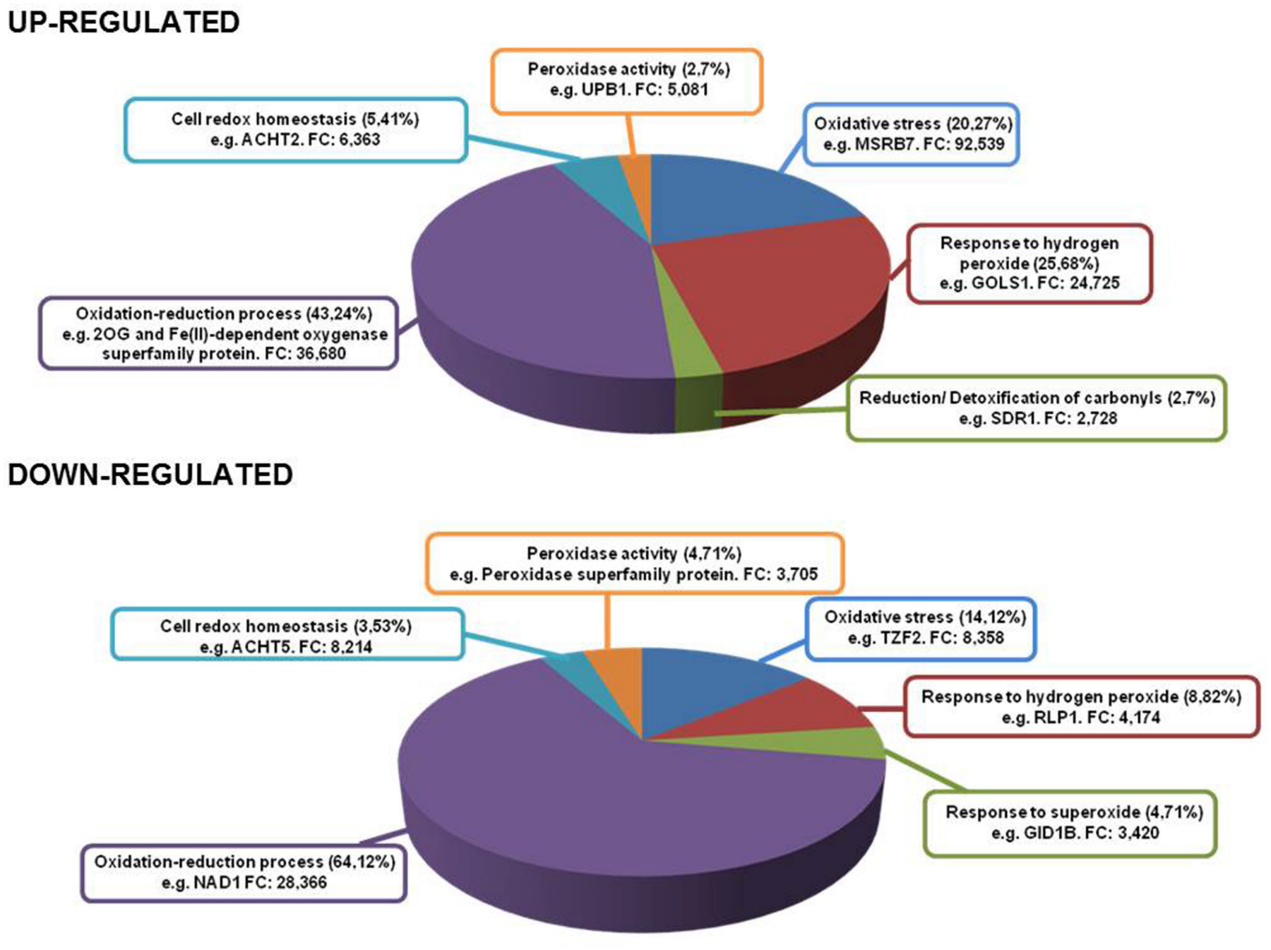
**Genes up- and down-regulated in different oxidative stress conditions**. Inside each box are depicted the type of stress and most representative gene in that category with its fold change.

With the aid of this (see Supplemental Table 6) transcriptomic analysis, we found a large amount of heat shock proteins (HSPs) and heat shock transcription factors (HSFs) associated with the responses to hydrogen peroxide (H_2_O_2_), accounting for 25.68% of total genes up-regulated (*AT1G52560, AT2G26150, AT4G25200, AT2G20560, AT2G32120, AT1G74310, AT5G37670, AT4G21320*, and *AT1G16030*) (Figure 5). Recently, *HsfA2* expression has been shown to be induced under different types of oxidative stress conditions such as H_2_O_2_treatment (Miller and Mittler, 2006), with its overexpression producing a higher level of tolerance to several environmental stresses (Li et al., 2005). Furthermore, Ln treatment induced the expression of a set of glutathione S-transferases (*AT2G29480, AT2G29470, AT2G29420, AT2G29490, AT2G29450, AT2G29460, AT2G47730, AT1G17170*, and *AT2G29440*) and of methionine sulfoxide reductase *B7* (MSRB7, *AT4G21830*, FC 92.539 up). In this respect, another up-regulated enzyme associated with the detoxification of oxidized proteins was the alkenal reductase (*AT5G16970*). The largest percentage of up-regulated genes was associated with oxidation-reduction processes which accounted for 43.24% of total genes. Among them, we found members of 2-oxoglutarate (2OG) and Fe(II)-dependent oxygenase superfamily, as the most over-expressed genes, and a significant proportion of members of the cytochrome P450 superfamily (CYP450) (*AT5G06900, AT4G19230, AT1G64950, AT2G27690, AT5G63450, AT5G47990, AT4G15393, AT5G06905, AT4G31500*, and *AT5G25180*) encoding for fatty acid hydroxylases among others. These enzymes are capable of producing different compounds of cutin, a part of the cuticle that protects plants against various stresses (Kolattukudy, 1980). We also detected the induction of alternative oxidase 1D (*AT1G32350*) which plays an important role in metabolic and signaling homeostasis during abiotic and biotic stress in plants (Vanlerberghe, 2013). Finally, we also observed gene up-regulation of the monodehydroascorbate reductase 3 (*MDAR3*) enzyme (*AT3G09940*) which encodes an enzyme involved in the oxidation-reduction process. This enzyme is present in the ascorbate-glutathione cycle and is responsible for the regeneration of reduced ascorbate, a major antioxidant in plant cells. In this regard, an increase in the transcript levels of the peroxisomal MDAR1 gene has been observed in *Pisum sativum* leaves subjected to mechanical wounding (Leterrier et al., 2005). This indicates that MDAR3 gene induction in *Arabidopsis* caused by Ln treatment may initiate the functioning of the ascorbate-glutathione cycle and thus control ROS production.

On the other hand, Ln treatment generated the down-regulation of several genes involved in oxidative stress (14.12%) such as *OXS2* (*AT2G41900*) and *OXS3* (*AT5G56550*). These genes have been shown to play a role in stress tolerance as they may act as chromatin remodeling factors in relation to the stress response to protect DNA or alter its transcriptional selectivity (Blanvillain et al., 2009). Finally, the most abundant down-regulated functional category was the oxidation-reduction process, accounting for 64.12% of total suppressed genes. Among these genes, the electron transport chain was inhibited, principally the components of NADH dehydrogenase complex I and cytochrome oxidase complex III (*AT2G07785, AT2G07689, ATMG00650, ATMG00285, AT4G16790, ATMG01360, ATMG00513, ATCG00890, ATCG01250, ATMG00580, ATMG00990, ATCG01090, ATMG00510, ATCG01080, ATCG01070, ATCG01100, ATCG01050, AT2G07751*, and *ATCG01010*). It has been reported that most photosynthesis-related genes are down-regulated after herbivore attack and may allow attacked plants to reinvest resources in other processes such as defense (Halitschke et al., 2001; Hui et al., 2003). This indicates that Ln was capable of reducing photosynthesis-related genes, possibly to counterbalance the induction of defense traits.

### Validation of Ln-responsive genes by quantitative real-time reverse transcription–PCR (qRT–PCR)

**Figure 6:**
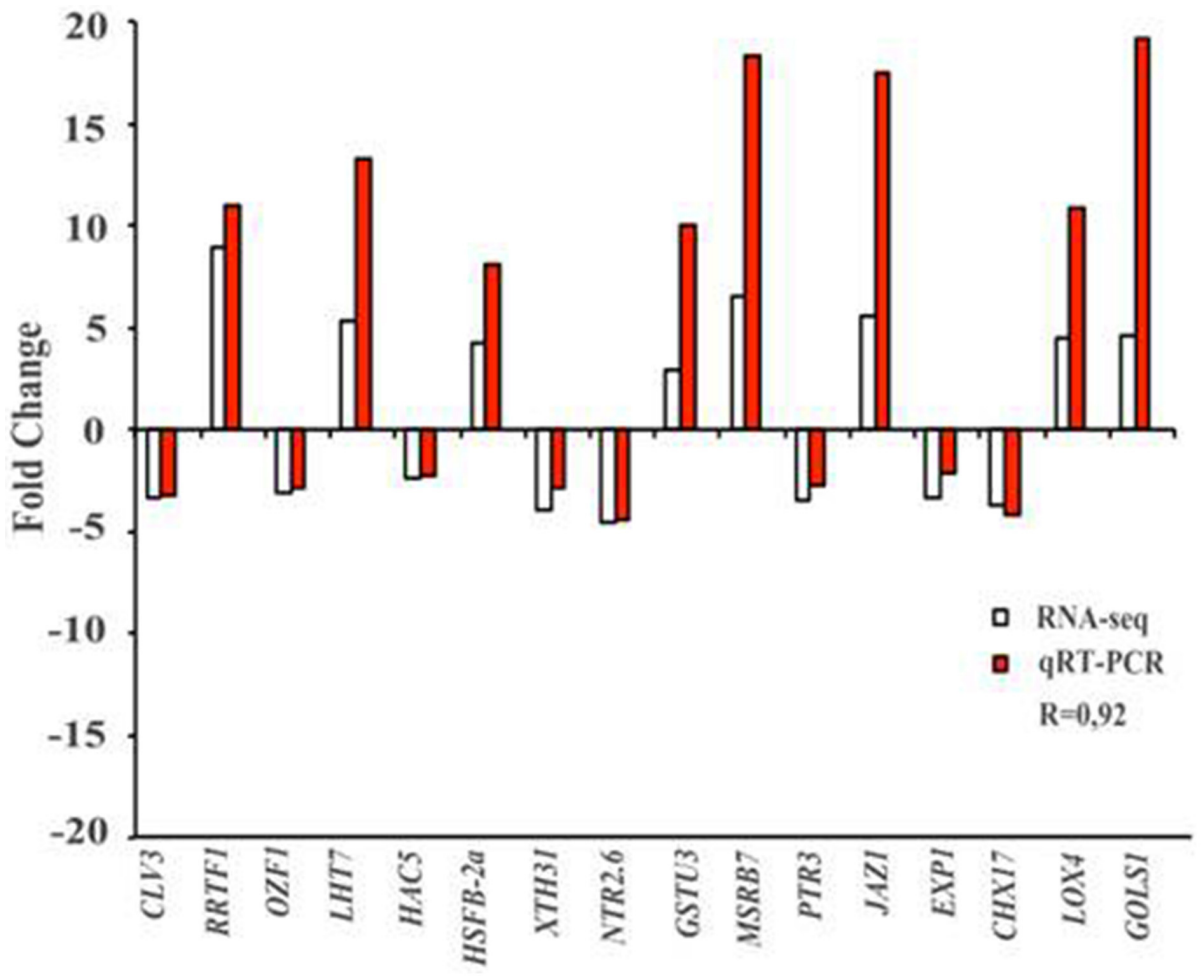
**qRT–PCR validation of RNA-seq results**. Sixteen genes identified previously as Ln-responsive genes by RNA-seq (white bar) in ACSC were randomly selected to analyze, by qRT–PCR the differential expression changes (red bars). Comparison of fold change of RNA-seq and qRT–PCR showed a correlation coefficient of 0.92, indicating that RNA-seq results were reliable. Results were average of two independent samples in triplicate. Standard deviations were less than 5% in all cases. *NRT2.6* (AT3G45060), *Arabidopsis thaliana* high affinity nitrate transporter 2.6; *XTH31* (AT3G44990), *Arabidopsis thaliana* xyloglucan endotransglycosylase/hydrolase 31; *CHX17* (AT4G23700), *Arabidopsis thaliana* cation/H(+) antiporter 17; *PTR3* (AT5G46050), *Arabidopsis thaliana* putative peptide transporter protein 3; *CLV3* (AT2G27250), *Arabidopsis thaliana* protein CLAVATA 3; *OZF1* (AT2G19810), *Arabidopsis thaliana* Oxidation-related Zinc Finger protein 1; *HAC5* (AT3G12980), *Arabidopsis thaliana* histone acetyltransferase 5; *JAZ1* (AT1G19180), *Arabidopsis thaliana* jasmonate-zim-domain protein 1; *LHT7* (AT4G35180), *Arabidopsis thaliana* LYS/HIS transporter 7; *GOLS1* (AT2G47180), *Arabidopsis thaliana* galactinol synthase 1; *HSFB-2a* (AT5G62020), *Arabidopsis thaliana* heat stress transcription factor B-2a; GSTU3 (AT2G29470), *Arabidopsis thaliana* glutathione S-transferase tau 3*; EXP1* (AT1G69530), *Arabidopsis thaliana* Alpha-Expansin protein 1; *MSRB7* (AT4G21830), *Arabidopsis thaliana* methionine sulfoxide reductase B7; *LOX4* (AT1G72520), *Arabidopsis thaliana* lipoxygenase 4; *RRTF1* (AT4G34410), *Arabidopsis thaliana* redox responsive transcription factor.

To validate RNA-seq results, we randomly assigned several Ln-responsive genes to conduct the expression analysis by qRT–PCR. Figure 6 shows the comparison between the qRT–PCR and RNA-seq analysis, showing that all the Ln-responsive genes tested and previously identified by RNA-seq were confirmed by qRT–PCR. The results showed a positive correlation between the two approaches (with a correlation coefficient of 0.92), indicating that the RNA-seq expression analysis performed is highly reliable.

## Discussion

LN is an important molecule in plant physiology due to is the precursor of jasmonate pathway, a key component of plant defense (Wasternack and Hause, 2013). In this respect, Ln has been shown to be involved in the plant's gene responses to pathogen attacks and mechanical wounding caused by insect feeding (Wasternack, 2014a). However, little is known about the role played by Ln in gene responses to other abiotic stress situations and specifically those mediated by genes which regulate the cellular redox state and/or mediated by an oxidative stress. With the aid of RNA-seq technology, we have analyzed the transcriptional effect of Ln on ACSC by using a Ln treatment which does not provoke oxidative stress, thus enabling new gene sets involved in ROS cross-talk signaling to be identified.

Our findings indicate that Ln is associated with the plant's response to different abiotic stress conditions including hyperosmotic salinity and heat acclimation. Finally, one of the most interesting processes we observed was the response to oxidative stress which led us to analyze the interaction between Ln and signaling mediated by oxidative stress-related processes, about which very little is known.

### Linolenic acid regulates biosynthesis and signaling of jasmonates in ACSC. involvement of linolenic acid in biotic stress situations

Ln treatment provoked the over-expression of several genes associated with jasmonate-biosynthetic processes, including lipoxygenases which constitute the first step in the formation of these molecules (Wasternack, 2014b). This phenomenon is probably due to an increase in JA levels after Ln treatment followed by a self-activation stage in JA biosynthesis that has been reported extensively in the literature (Wasternack, 2007). In addition, the application of Ln to ACSC resulted in the up-regulation of *ERF1* (*AT3G23240*) which acts downstream of the intersection between ethylene and jasmonate pathways. It has also been suggested that this gene is a key element in the integration of both signals for regulating defense response genes (Lorenzo et al., 2003). We also observed an increase in the number of transcripts of several transcription factor families such as *WRKY, bHLH, MYB*, and *NAC*. The over-expression of all these genes shows that poly-unsaturated fatty acid Ln induces the oxylipin pathway in order to produce JA-related phytohormones and consequently all components involved in its generation and functioning. In addition, several down-regulated Ln genes active in jasmonate-related processes were detected. Carbonic anhydrase (CA) was found to be a down-regulated enzyme. In this regard, in some stress situations such as wounding and herbivore attacks, CA gene expression in wounded *Capsicum annuum* leaves was observed to be lower than that in control leaves. This suggests that proteins playing a role in photosynthesis are down-regulated by metabolic reconfiguration in order to maintain a balance between defense and tolerance (Mahajan et al., 2014). Furthermore, several *MYB* transcription factors were down-regulated. In plants, *MYB* genes are a large family functionally active in regulating several defense processes (Kirik et al., 1998; Stracke et al., 2001), indicating that Ln launches a mechanism capable of improving the plant's defenses in different stress situations.

The release of Ln from membranes in response to intracellular signaling events is a key stage in the activation of defensive genes, with phospholipases being the enzymes responsible for both basal and stimulus-induced production of JA. Phospholipase A has actually been active in wound- and systemin-induced JA formation in tomato (Narváez-Vásquez et al., 1999), while phospholipase D α1 also promotes wound-induced accumulation of free Ln and JA in *Arabidopsis* (Zien et al., 2001; He et al., 2002). Elicitation of wound responses in plants appearing upon mechanical wounding (abiotic stress) or herbivore attacks (biotic stress) is one of the most prominent examples and extensively studied areas where JA/JA-Ile is involved as a signal. In this regard, RNA-seq analysis enabled us to identify Ln-regulated genes which play a role in the biotic, abiotic and oxidative stress responses of ACSC. With respect to genes participating in biotic stress responses, our analysis revealed the over-expression of *WRKY40* and *RRTF1* transcription factors. Although the role played by *RRTF1* in plant defense still needs to be rigorously tested, recent reports indicate its involvement in regulating redox homeostasis during stress, with *RRTF1* expression being dependent on COI1, a key regulator of JA signaling (Khandelwal et al., 2008; Wang et al., 2008). A direct physical *in vivo* interaction has also been shown to exist between WRKY40 and RRTF1, indicating that this WRKY transcription factor acts as a direct transcriptional repressor of RRTF1 (Pandey et al., 2010). We also detected the up-regulation of the *BAG2* gene which has been identified as containing a regulator of plant programmed cell death (PCD). Given that AtBAG family members have been shown to inhibit plant PCD pathways in response to stress (Doukhanina et al., 2006), Ln may act as a promoter of cell survival and resistance to biotic stress situations. In this respect, we detected over-expression of the *JAS1*/*JAZ10* TF which is a negative regulator of JA signaling in *Arabidopsis* seedlings and of disease susceptibility to *Pseudomonas syringae* strain DC3000 (Demianski et al., 2012). This activity is mediated by the JAZ10.4 alternative splice variant that lacks the Jas motif in the C-terminal and mediates interaction with COI1 and MYC2 (Moreno et al., 2013). Up-regulation of this gene may be related to the JA self-regulation mechanism which controls jasmonate over-production.

### Linolenic acid induces key genes of enzymes involved in the plant's defense against abiotic stress

An important finding produced by RNA-seq data analysis was the regulation by Ln treatment of abiotic stress response genes. Most previous studies have focused on biotic stress responses and the oxylipin pathway following the application of JA-related molecules. Nevertheless, the role of these molecules in processes participating in abiotic stress situations, particularly in relation to oxidative stress, is not very well known. In this regard, we detected an up-regulation of the galactinol synthase enzyme (GOLS1, *AT2G47180*) with a FC of 24.725. GolS catalyzes the first stage in the biosynthesis of raffinose family oligosaccharides (RFOs) from UDP-galactose and also RFO-derived molecules like raffinose and stachyose which intervene in the accumulation of osmoprotectants during seed development. In this respect, galactinol synthase has been shown to play a key role in the accumulation of galactinol and raffinose under abiotic stress conditions such as drought, high salinity and cold (Taji et al., 2002). In addition to these findings, high intracellular levels of galactinol and raffinose have recently be demonstrated to correlate with increased tolerance to methylviologen (MV) treatment as well as salinity and chilling stress conditions. This suggests that these molecules may scavenge hydroxyl radicals protecting plant cells from oxidative damage caused by these stresses (Nishizawa et al., 2008). These findings indicate that Ln could mediate plant responses to abiotic stresses by inducing an important defense mechanism mediated by this galactinol synthase. It is important to note that a large percentage of up-regulated genes in response to Ln treatment encoded heat shock proteins (HSPs) or chaperones. As several different abiotic stress conditions can cause protein dysfunction, maintaining these proteins in their functional conformations and preventing the aggregation of non-native proteins are particularly important for cell survival under stress conditions. These HSPs can play a crucial role in protecting plants against stress by re-establishing the normal protein conformation and thus cellular homeostasis (Wang et al., 2004). In this regard, Ln could initiate a defense mechanism mediated by the induction of key components like these protein stabilizers involved in defending the plant against different abiotic stresses. In conclusion, Ln is capable of modulating the expression levels of different genes participating in a wide variety of abiotic processes such as drought, salinity and wounding. The Ln poly-unsaturated fatty acid is involved in other pathways not directly associated with biotic responses and also with several abiotic stress situations, indicating that it plays a very important role as a signaling mediator.

### Linolenic acid response against oxidative stress: induction of methione sulfoxide reductase (MSRB7) and alkenal reductase

Until now, most studies have focused on the exogenous administration of JA-related molecules such as methyl-jasmonate, which act as signaling molecules in order to induce responses to biotic stress situations like pathogen attacks and wounding induced by herbivores. For this reason, an important aim of this study has been to identify the genes and potential metabolic pathways involved in the regulation of the cellular redox state and/or mediated by an oxidative stress in response to Ln treatment under non-oxidative stress conditions. Remarkably, in this regard, we detected the over-expression of methione sulfoxide reductase B7 (*MSRB7*, FC 92.539). Methionine oxidation by ROS (John et al., 2001) leads to the formation of MetSO (Boschi-Muller et al., 2008) which could alter both the activity and conformation of many proteins (Dos Santos et al., 2005; Rouhier et al., 2006). This enzyme catalyzes the reduction of methionine sulfoxides back to methionine and can also repair oxidized proteins and protect against oxidative damage (Moskovitz, 2005). Begara-Morales et al. (2014) showed that *MSRB7* was highly induced by S-nitrosoglutathione (GSNO), suggesting that this enzyme plays a very important role in the oxidative metabolism and specifically in nitric oxide metabolism. It is therefore important to determine whether a poly-unsaturated fatty acid such as Ln is able to induce a key gene in an enzyme involved in protection against methionine oxidation, indicating crosstalk between redox status and the nitric oxide metabolism. Another important enzyme detected by this RNA-seq analysis relating to oxidized protein detoxification was the alkenal reductase enzyme. Oxidative stress produced in some biotic/ abiotic stress situations can lead to the production of ROS which damage biomolecules such as proteins and lipids. Because linoleic and linolenic acids are sources of many short-chain carbonyls due to peroxidation, biomolecules are threatened by the toxicity of reactive compounds including α,β-unsaturated carbonyls, which are involved in the pathophysiological effects associated with oxidative stress in cells and tissues (Yamauchi et al., 2011). This enzyme catalyzes the reduction of the α,β-unsaturated bond of reactive carbonyls which are active in anti-oxidative plant defenses (Mano et al., 2005). Finally, we also detected the induction of various glutathione S-transferase genes and a large percentage of several members of the CYP450 superfamily. In this regard, oxidative burst, involving the rapid production of enormous amounts of ROS, is one of the first mechanisms of defense against certain biotic and abiotic stresses such as wounding, cells subjected to mechanical stress or pathogen attacks. Faced with these oxidative attacks, damaged plants initiate a series of defense mechanisms by, for example, releasing Ln from cell membranes throughout several lipases. In this sense, we observed that Ln was able to enhance the expression of MSRB7 and alkenal reductase which could repair oxidative modifications in proteins caused by the high levels of ROS generated in these stress situations. Moreover, the glutathione metabolism appears to be of crucial importance due to the large number of glutathione S-transferase genes induced. These enzymes play a crucial role in detoxification of peroxidised lipids, thus contributing to the defense response potentiated by Ln. Furthermore, the induction of genes from members of the *CYP450* family generates compounds as protectors and potent elicitors of defense mechanisms (Kolattukudy, 1980; Schweizer et al., 1998). In this regard, *CYP94C1* has been reported to be induced at the transcriptional level by the methyl-jasmonate stress hormone (Kandel et al., 2007) which corroborates the findings obtained by this RNA-seq analysis.

## Author contributions

The experiments were conceived and designed by: JBB, FC, FL, BS, CM, and JCB. The experiments were performed by: CM, BS, JCB, MP, RV, FL, AF, JJ, JF, FC, and JBB. The data were analyzed by: CM, JBB, JCB, and FL. The paper was written by: CM, JBB, FC, FL, and JCB.

### Conflict of interest statement

The authors declare that the research was conducted in the absence of any commercial or financial relationships that could be construed as a potential conflict of interest.

## Supplementary material

Supplemental Table 1 **Fatty acid composition of *Arabidopsis thaliana* cell suspension cultures (ACSC)**.

Supplemental Table 2 **Effect of Ln on oxidative stress markers**.

Supplemental Table 3 **Ln-responsive genes involved in jasmonate-related processes**.

Supplemental Table 4 **Ln-responsive genes involved in biotic stress situations**.

Supplemental Table 5 **Ln-responsive genes involved in abiotic stress situations**.

Supplemental Table 6 **Ln-responsive genes involved in oxidative stress conditions**.

Supplemental Table 7 **2FC-up-regulated genes by Linolenic acid-treatment**.

Supplemental Table 8 **2FC-down-regulated genes by Linolenic acid-treatment**.

Supplemental Table 9 **Oligonucleotides used for qRT-PCR**.
