# Supplementary material for: Transcriptomic profiling of linolenic acid-responsive genes in ROS signaling from RNA-seq data in Arabidopsis
Source: Front Plant Sci. 2015 Mar 17;6:122. doi: 10.3389/fpls.2015.00122 (PMC4362301; doi:10.3389/fpls.2015.00122)
Supplement: Supplemental Table 1 — Fatty acid composition of Arabidopsis thaliana cell suspension cultures (ACSC). [file DataSheet1.ZIP › Table 4.PDF]

Biotic stress-related genes (Control vs Ln UP)

| FC | ID | Biotic stress |
|---|---|---|
| 6,928 | AT5G62100 | **ATBAG2, BAG2, BCL-2-ASSOCIATED ATHANOGENE 2.** |
| 5,421 | AT1G79680 | **ATWAKL10, WAKL10, WALL ASSOCIATED KINASE (WAK)-LIKE 10.** |
| 4,975 | AT2G46240 | **ARABIDOPSIS THALIANA BCL-2-ASSOCIATED ATHANOGENE 6, ATBAG6, BAG6, BCL-2-ASSOCIATED ATHANOGENE 6.** |
| 2,975 | AT1G61340 | **ATFBS1, F-BOX STRESS INDUCED 1, FBS1.** |
| | | **Response to chitin** |
| 30,219 | AT4G17490 | **ATERF6, ERF-6-6, ERF6, ETHYLENE RESPONSIVE ELEMENT BINDING FACTOR 6.** |
| 27,503 | AT2G26530 | **AR781.** |
| 26,669 | AT1G80840 | **ATWRKY40, WRKY DNA-BINDING PROTEIN 40, WRKY40.** |
| 24,812 | AT1G17380 | **JASMONATE-ZIM-DOMAIN PROTEIN 5, JAZ5, TIFY11A.** |
| 22,834 | AT1G72520 | **ARABIDOPSIS THALIANA LIPOXYGENASE 4, ATLOX4, LIPOXYGENASE 4, LOX4.** |
| 22,102 | AT4G17500 | **ATERF-1, ERF-1, ETHYLENE RESPONSIVE ELEMENT BINDING FACTOR 1.** |
| 19,423 | AT5G62020 | **ARABIDOPSIS THALIANA HEAT SHOCK TRANSCRIPTION FACTOR B2A, AT-HSFB2A, HEAT SHOCK TRANSCRIPTION FACTOR B2A, HSFB2A.** |
| 15,027 | AT3G44260 | **ATCAF1A, CAF1A, CCR4- ASSOCIATED FACTOR 1A.** |
| 12,267 | AT1G32640 | **ATMYC2, JAI1, JASMONATE INSENSITIVE 1, JIN1, MYC2, RD22BP1, ZBF1.** |
| 12,125 | AT5G05410 | **DEHYDRATION-RESPONSIVE ELEMENT BINDING PROTEIN 2, DRE-BINDING PROTEIN 2A, DREB2, DREB2A.** |
| 11,762 | AT1G28370 | **ATERF11, ERF DOMAIN PROTEIN 11, ERF11.** |
| 11,405 | AT2G26150 | **ATHSFA2, HEAT SHOCK TRANSCRIPTION FACTOR A2, HSFA2.** |
| 11,382 | AT1G17420 | **ARABIDOPSIS THALIANA LIPOXYGENASE 3, ATLOX3, LIPOXYGENASE 3, LOX3.** |
| 9,711 | AT5G42650 | **ALLENE OXIDE SYNTHASE, AOS, CYP74A, CYTOCHROME P450 74A, DDE2, DELAYED DEHISCENCE 2.** |
| 9,223 | AT3G23250 | **ATMYB15, ATY19, MYB DOMAIN PROTEIN 15, MYB15.** |
| 8,662 | AT5G47230 | **ATERF-5, ATERF5, ERF5, ETHYLENE RESPONSIVE ELEMENT BINDING FACTOR 5, ETHYLENE RESPONSIVE ELEMENT BINDING FACTOR- 5.** |
| 8,635 | AT3G15210 | **ATERF-4, ATERF4, ERF4, ETHYLENE RESPONSIVE ELEMENT BINDING FACTOR 4, RAP2.5, RELATED TO AP2 5.** |
| 7,910 | AT1G66090 | **Disease resistance protein.** |
| 7,847 | AT5G04340 | **C2H2, COLD INDUCED ZINC FINGER PROTEIN 2, CZF2, ZAT6, ZINC FINGER OF ARABIDOPSIS THALIANA 6.** |
| 7,230 | AT5G59820 | **ATZAT12, RESPONSIVE TO HIGH LIGHT 41, RHL41, ZAT12.** |
| 6,299 | AT5G57560 | **TCH4, TOUCH 4, XTH22, XYLOGLUCAN ENDOTRANSGLUCOSYLASE/HYDROLASE 22.** |
| 5,890 | AT1G73500 | **ATMKK9, MAP KINASE KINASE 9, MKK9.** |
| 5,839 | AT5G47220 | **ATERF-2, ATERF2, ERF2, ETHYLENE RESPONSE FACTOR- 2, ETHYLENE RESPONSIVE ELEMENT BINDING FACTOR 2.** |
| 5,775 | AT1G27730 | **SALT TOLERANCE ZINC FINGER, STZ, ZAT10.** |
| 5,648 | AT1G66160 | **"CYS, MET, PRO, AND GLY PROTEIN 1", ATCMPG1, CMPG1.** |
| 5,347 | AT5G37770 | **CALMODULIN-LIKE 24, CML24, TCH2, TOUCH 2.** |
| 5,119 | AT3G28210 | **PMZ, SAP12, STRESS-ASSOCIATED PROTEIN 12.** |
| 5,092 | AT2G37430 | **C2H2 and C2HC zinc fingers superfamily protein.** |

Biotic stress-related genes (Control vs Ln UP)

| | | |
|---|---|---|
| 5,084 | AT1G01720 | **ANAC002, ARABIDOPSIS NAC DOMAIN CONTAINING PROTEIN 2, ATAF1**. |
| 5,079 | AT5G03380 | **Heavy metal transport/detoxification superfamily protein**. |
| 5,076 | AT2G35930 | **ATPUB23, PLANT U-BOX 23, PUB23**. |
| 4,738 | AT3G50060 | **MYB DOMAIN PROTEIN 77, MYB77**. |
| 4,726 | AT2G18060 | **ANAC037, ARABIDOPSIS NAC DOMAIN CONTAINING PROTEIN 37, VASCULAR RELATED NAC-DOMAIN PROTEIN 1, VND1**. |
| 4,724 | AT1G20510 | **OPC-8:0 COA LIGASE1, OPCL1**. |
| 4,417 | AT1G76650 | **CALMODULIN-LIKE 38, CML38**. |
| 4,396 | AT3G25250 | **AGC2, AGC2-1, ATOXI1, OXI1, OXIDATIVE SIGNAL-INDUCIBLE1**. |
| 4,074 | AT3G19580 | **AZF2, ZF2, ZINC-FINGER PROTEIN 2**. |
| 3,925 | AT3G52400 | **ATSYP122, SYNTAXIN OF PLANTS 122, SYP122.** |
| 3,908 | AT3G62720 | **ATXT1, XT1, XXT1, XYG XYLOSYLTRANSFERASE 1, XYLOSYLTRANSFERASE 1**. |
| 3,797 | AT4G24570 | **DIC2, DICARBOXYLATE CARRIER 2.** |
| 3,680 | AT4G37260 | **ATMYB73, MYB DOMAIN PROTEIN 73, MYB73.** |
| 3,667 | AT3G46090 | **ZAT7.** |
| 3,655 | AT1G62300 | **ATWRKY6, WRKY6**. |
| 3,637 | AT1G02450 | **NIM1-INTERACTING 1, NIMIN-1, NIMIN1.** |
| 3,628 | AT1G15520 | **ABCG40, ARABIDOPSIS THALIANA ATP-BINDING CASSETTE G40, ATABCG40, ATP-BINDING CASSETTE G40, ATPDR12, PDR12, PLEIOTROPIC DRUG RESISTANCE 12.** |
| 3,622 | AT2G35980 | **ARABIDOPSIS NDR1/HIN1-LIKE 10, ATNHL10, NDR1/HIN1-LIKE, NHL10, YELLOW-LEAF-SPECIFIC GENE 9, YLS9**. |
| 3,536 | AT1G61890 | **MATE efflux family protein**. |
| 3,534 | AT1G04100 | **IAA10, INDOLEACETIC ACID-INDUCED PROTEIN 10**. I |
| 3,501 | AT3G50260 | **ATERF011, CEJ1, COOPERATIVELY REGULATED BY ETHYLENE AND JASMONATE 1, DEAR1, DREB AND EAR MOTIF PROTEIN 1.** |
| 3,492 | AT5G62520 | **SIMILAR TO RCD ONE 5, SRO5.** |
| 3,459 | AT5G67300 | **ARABIDOPSIS THALIANA MYB DOMAIN PROTEIN 44, ATMYB44, ATMYBR1, MYB DOMAIN PROTEIN R1, MYB44, MYBR1**. |
| 3,419 | AT3G04640 | **Glycine-rich protein**. |
| 3,373 | AT4G39030 | **EDS5, ENHANCED DISEASE SUSCEPTIBILITY 5, SALICYLIC ACID INDUCTION DEFICIENT 1, SCORD3, SID1, SUSCEPTIBLE TO CORONATINE-DEFICIENT PST DC3000 3**. |
| 3,193 | AT4G39670 | **Glycolipid transfer protein (GLTP) family protein**. |
| 3,134 | AT2G33580 | **LYK5, LYSM-CONTAINING RECEPTOR-LIKE KINASE 5**. |
| 3,018 | AT5G18150 | **Methyltransferase-related protein.** |
| 2,975 | AT1G61340 | **ATFBS1, F-BOX STRESS INDUCED 1, FBS1**. |
| 2,905 | AT1G24140 | **Matrix metalloprotease domain-containing protein**. |
| 2,867 | AT4G25390 | **Protein kinase family protein**. |
| 2,772 | AT5G66070 | **NEP1-interacting protein-like 1.** |
| 2,747 | AT4G34150 | **Calcium-dependent lipid-binding domain-containing protein.** |
| 2,739 | AT2G30040 | **MAPKKK14, MITOGEN-ACTIVATED PROTEIN KINASE KINASE KINASE 14**. |
| 2,594 | AT2G32030 | **Acyl-CoA N-acyltransferases (NAT) superfamily protein**. |
| 2,573 | AT2G22500 | **ATPUMP5, DIC1, DICARBOXYLATE CARRIER 1, PLANT UNCOUPLING** |

Biotic stress-related genes (Control vs Ln UP)

| | | |
|---|---|---|
| | | **MITOCHONDRIAL PROTEIN 5, UCP5, UNCOUPLING PROTEIN 5**. |
| 2,553 | AT4G24160 | Encodes a soluble **lysophosphatidic acid acyltransferase** with additional triacylglycerol lipase and phosphatidylcholine hydrolyzing enzymatic activities. |
| 2,465 | AT4G33920 | **Putative protein phosphatase 2C 63**. |
| 2,430 | AT5G25930 | **Protein kinase family protein with leucine-rich repeat domain.** |
| 2,415 | AT3G55980 | **ATSZF1, SALT-INDUCIBLE ZINC FINGER 1, SZF1**. |
| 2,382 | AT5G64870 | **SPFH/Band 7/PHB domain-containing membrane-associated protein family.** |
| 2,357 | AT5G64310 | **AGP1, ARABINOGALACTAN PROTEIN 1, ATAGP1**. |
| 2,351 | AT3G44190 | **FAD/NAD(P)-binding oxidoreductase family protein**. |
| 2,303 | AT1G09070 | **(AT)SRC2, SOYBEAN GENE REGULATED BY COLD-2, SRC2**. |
| 2,266 | AT3G11840 | **PLANT U-BOX 24, PUB24**. |
| 2,249 | AT3G52800 | **Zinc finger A20 and AN1 domain-containing stress-associated protein 6**. |
| 2,213 | AT4G11280 | **1-AMINOCYCLOPROPANE-1-CARBOXYLIC ACID (ACC) SYNTHASE 6, ACS6, ATACS6**. |
| 2,213 | AT5G59550 | **ARABIDOPSIS THALIANA RING AND DOMAIN OF UNKNOWN FUNCTION 1117 2, ATRDUF2, RDUF2, RING AND DOMAIN OF UNKNOWN FUNCTION 1117 2**. |
| 2,126 | AT1G50740 | **Transmembrane proteins 14C**. |
| 2,057 | AT4G32440 | **Plant tudor-like RNA-binding protein**. |
| 2,044 | AT5G22570 | **ARABIDOPSIS THALIANA WRKY DNA-BINDING PROTEIN 38, ATWRKY38, WRKY DNA-BINDING PROTEIN 38, WRKY38.** |
| 2,034 | AT1G80820 | **ATCCR2, CCR2, CINNAMOYL COA REDUCTASE**. |
| | | **Response to fungus** |
| 36,838 | AT5G13220 | **JAS1, JASMONATE-ASSOCIATED 1, JASMONATE-ZIM-DOMAIN PROTEIN 10, JAZ10, TIFY DOMAIN PROTEIN 9, TIFY9**. |
| 36,680 | AT5G05600 | **2-oxoglutarate (2OG) and Fe(II)-dependent oxygenase superfamily protein.** |
| 26,669 | AT1G80840 | **ATWRKY40, WRKY DNA-BINDING PROTEIN 40, WRKY40.** |
| 25,665 | AT1G72450 | **JASMONATE-ZIM-DOMAIN PROTEIN 6, JAZ6, TIFY DOMAIN PROTEIN 11B, TIFY11B.** |
| 24,812 | AT1G17380 | **JASMONATE-ZIM-DOMAIN PROTEIN 5, JAZ5, TIFY11A.** |
| 24,266 | AT1G44350 | **IAA-LEUCINE RESISTANT (ILR)-LIKE GENE 6, ILL6.** |
| 22,834 | AT1G72520 | **ARABIDOPSIS THALIANA LIPOXYGENASE 4, ATLOX4, LIPOXYGENASE 4, LOX4.** |
| 16,058 | AT5G08790 | **ANAC081, ARABIDOPSIS NAC DOMAIN CONTAINING PROTEIN 81, ATAF2**. |
| 13,449 | AT3G25780 | **ALLENE OXIDE CYCLASE 3, AOC3**. |
| 12,956 | AT5G13220 | **JAS1, JASMONATE-ASSOCIATED 1, JASMONATE-ZIM-DOMAIN PROTEIN 10, JAZ10, TIFY DOMAIN PROTEIN 9, TIFY9**. |
| 12,267 | AT1G32640 | **ATMYC2, JAI1, JASMONATE INSENSITIVE 1, JIN1, MYC2, RD22BP1, ZBF1.** |
| 11,762 | AT1G28370 | **ATERF11, ERF DOMAIN PROTEIN 11, ERF11.** |
| 11,382 | AT1G17420 | **ARABIDOPSIS THALIANA LIPOXYGENASE 3, ATLOX3, LIPOXYGENASE 3, LOX3.** |
| 9,711 | AT5G42650 | **ALLENE OXIDE SYNTHASE, AOS, CYP74A, CYTOCHROME P450 74A,** |

Biotic stress-related genes (Control vs Ln UP)

| | | |
|---|---|---|
| | | **DDE2, DELAYED DEHISCENCE 2.** |
| 9,619 | AT1G70700 | **JASMONATE-ZIM-DOMAIN PROTEIN 9, JAZ9, TIFY7.** |
| 8,813 | AT1G74950 | **JASMONATE-ZIM-DOMAIN PROTEIN 2, JAZ2, TIFY10B**. |
| 8,666 | AT4G19230 | **"CYTOCHROME P450, FAMILY 707, SUBFAMILY A, POLYPEPTIDE 1", CYP707A1**. |
| 8,056 | AT5G20230 | **ATBCB, BCB, BLUE COPPER BINDING PROTEIN, BLUE-COPPER-BINDING PROTEIN, SAG14, SENESCENCE ASSOCIATED GENE 14**. |
| 7,910 | AT1G66090 | **Disease resistance protein**. |
| 7,230 | AT5G59820 | **ATZAT12, RESPONSIVE TO HIGH LIGHT 41, RHL41, ZAT12.** |
| 6,480 | AT1G05800 | **DGL, DONGLE.** |
| 5,846 | AT2G27690 | **"CYTOCHROME P450, FAMILY 94, SUBFAMILY C, POLYPEPTIDE 1", CYP94C1**. |
| 5,775 | AT1G27730 | **SALT TOLERANCE ZINC FINGER, STZ, ZAT10**. |
| 5,603 | AT2G06050 | **ATOPR3, DDE1, DELAYED DEHISCENCE 1, OPR3, OXOPHYTODIENOATE-REDUCTASE 3**. |
| 5,421 | AT1G79680 | **ATWAKL10, WAKL10, WALL ASSOCIATED KINASE (WAK)-LIKE 10.** |
| 5,404 | AT3G06490 | **ATMYB108, BOS1, BOTRYTIS-SUSCEPTIBLE1, MYB DOMAIN PROTEIN 108, MYB108**. |
| 5,084 | AT1G01720 | **ANAC002, ARABIDOPSIS NAC DOMAIN CONTAINING PROTEIN 2, ATAF1**. |
| 5,076 | AT2G35930 | **ATPUB23, PLANT U-BOX 23, PUB23**. |
| 4,975 | AT2G46240 | **ARABIDOPSIS THALIANA BCL-2-ASSOCIATED ATHANOGENE 6, ATBAG6, BAG6, BCL-2-ASSOCIATED ATHANOGENE 6**. |
| 4,724 | AT1G20510 | **OPC-8:0 COA LIGASE1, OPCL1**. |
| 4,624 | AT2G05940 | **RIPK, RPM1-INDUCED PROTEIN KINASE**. |
| 4,417 | AT1G76650 | **CALMODULIN-LIKE 38, CML38**. |
| 4,329 | AT5G38280 | **PR5-LIKE RECEPTOR KINASE, PR5K**. |
| 3,925 | AT3G52400 | **ATSYP122, SYNTAXIN OF PLANTS 122, SYP122.** |
| 3,637 | AT1G02450 | **NIM1-INTERACTING 1, NIMIN-1, NIMIN1.** |
| 3,628 | AT1G15520 | **ABCG40, ARABIDOPSIS THALIANA ATP-BINDING CASSETTE G40, ATABCG40, ATP-BINDING CASSETTE G40, ATPDR12, PDR12, PLEIOTROPIC DRUG RESISTANCE 12.** |
| 3,622 | AT2G35980 | **ARABIDOPSIS NDR1/HIN1-LIKE 10, ATNHL10, NDR1/HIN1-LIKE, NHL10, YELLOW-LEAF-SPECIFIC GENE 9, YLS9**. |
| 3,459 | AT5G67300 | **ARABIDOPSIS THALIANA MYB DOMAIN PROTEIN 44, ATMYB44, ATMYBR1, MYB DOMAIN PROTEIN R1, MYB44, MYBR1**. |
| 3,420 | AT3G51450 | **Calcium-dependent phosphotriesterase superfamily protein**. |
| 3,373 | AT4G39030 | **EDS5, ENHANCED DISEASE SUSCEPTIBILITY 5, SALICYLIC ACID INDUCTION DEFICIENT 1, SCORD3, SID1, SUSCEPTIBLE TO CORONATINE-DEFICIENT PST DC3000 3**. |
| 3,264 | AT3G15500 | **ANAC055, ATNAC3, NAC DOMAIN CONTAINING PROTEIN 3, NAC DOMAIN CONTAINING PROTEIN 55, NAC055, NAC3**. |
| 3,193 | AT4G39670 | **Glycolipid transfer protein (GLTP) family protein**. |
| 3,091 | AT5G53750 | **CBS domain-containing protein**. |
| 2,594 | AT2G32030 | **Acyl-CoA N-acyltransferases (NAT) superfamily protein**. |
| 2,478 | AT2G46510 | **ABA-INDUCIBLE BHLH-TYPE TRANSCRIPTION FACTOR, AIB, ATAIB.** |
| 2,470 | AT5G52020 | **ERF025**. |
| 2,415 | AT3G55980 | **ATSZF1, SALT-INDUCIBLE ZINC FINGER 1, SZF1**. |

Biotic stress-related genes (Control vs Ln UP)

| | | |
|---|---|---|
| 2,386 | AT2G30020 | Encodes **AP2C1**. |
| 2,266 | AT3G11840 | **PLANT U-BOX 24, PUB24**. |
| 2,191 | AT5G42050 | **DCD (Development and Cell Death) domain protein**. |
| 2,126 | AT1G50740 | **Transmembrane proteins 14C**. |
| 2,044 | AT5G22570 | **ARABIDOPSIS THALIANA WRKY DNA-BINDING PROTEIN 38, ATWRKY38, WRKY DNA-BINDING PROTEIN 38, WRKY38.** |
| 2,034 | AT1G80820 | **ATCCR2, CCR2, CINNAMOYL COA REDUCTASE**. |
| 2,010 | AT5G61890 | **ERF114**. |
| | | **Pathogen attack** |
| 6,928 | AT5G62100 | **ATBAG2, BAG2, BCL-2-ASSOCIATED ATHANOGENE 2**. |
| 4,975 | AT2G46240 | **ARABIDOPSIS THALIANA BCL-2-ASSOCIATED ATHANOGENE 6, ATBAG6, BAG6, BCL-2-ASSOCIATED ATHANOGENE 6**. |
| | | **Defense response & Defense response signaling pathway** |
| 26,669 | AT1G80840 | **ATWRKY40, WRKY DNA-BINDING PROTEIN 40, WRKY40.** |
| 22,834 | AT1G72520 | **ARABIDOPSIS THALIANA LIPOXYGENASE 4, ATLOX4, LIPOXYGENASE 4, LOX4.** |
| 15,292 | AT2G32140 | **Transmembrane receptors.** |
| 15,027 | AT3G44260 | **ATCAF1A, CAF1A, CCR4- ASSOCIATED FACTOR 1A.** |
| 13,449 | AT3G25780 | **ALLENE OXIDE CYCLASE 3, AOC3**. |
| 12,267 | AT1G32640 | **ATMYC2, JAI1, JASMONATE INSENSITIVE 1, JIN1, MYC2, RD22BP1, ZBF1.** |
| 11,762 | AT1G28370 | **ATERF11, ERF DOMAIN PROTEIN 11, ERF11.** |
| 9,711 | AT5G42650 | **ALLENE OXIDE SYNTHASE, AOS, CYP74A, CYTOCHROME P450 74A, DDE2, DELAYED DEHISCENCE 2.** |
| 9,223 | AT3G23250 | **ATMYB15, ATY19, MYB DOMAIN PROTEIN 15, MYB15**. |
| 8,666 | AT4G19230 | **"CYTOCHROME P450, FAMILY 707, SUBFAMILY A, POLYPEPTIDE 1", CYP707A1**. |
| 8,056 | AT5G20230 | **ATBCB, BCB, BLUE COPPER BINDING PROTEIN, BLUE-COPPER-BINDING PROTEIN, SAG14, SENESCENCE ASSOCIATED GENE 14**. |
| 7,910 | AT1G66090 | **Disease resistance protein**. |
| 7,597 | AT5G52050 | **MATE efflux family protein**. |
| 6,480 | AT1G05800 | **DGL, DONGLE.** |
| 5,404 | AT3G06490 | **ATMYB108, BOS1, BOTRYTIS-SUSCEPTIBLE1, MYB DOMAIN PROTEIN 108, MYB108**. |
| 5,076 | AT2G35930 | **ATPUB23, PLANT U-BOX 23, PUB23**. |
| 5,000 | AT4G23280 | **CRK20, CYSTEINE-RICH RLK (RECEPTOR-LIKE PROTEIN KINASE) 20**. |
| 4,975 | AT2G46240 | **ARABIDOPSIS THALIANA BCL-2-ASSOCIATED ATHANOGENE 6, ATBAG6, BAG6, BCL-2-ASSOCIATED ATHANOGENE 6**. |
| 4,951 | AT3G18690 | **MAP KINASE SUBSTRATE 1, MKS1**. |
| 4,624 | AT2G05940 | **RIPK, RPM1-INDUCED PROTEIN KINASE**. |
| 4,189 | AT5G56030 | **ATHSP90.2, EARLY-RESPONSIVE TO DEHYDRATION 8, ERD8, HEAT SHOCK PROTEIN 90.2, HEAT SHOCK PROTEIN 81-2, HEAT SHOCK PROTEIN 81.2, HEAT SHOCK PROTEIN 90.2, HSP81-2, HSP81.2, HSP90.2.** A |
| 3,925 | AT3G52400 | **ATSYP122, SYNTAXIN OF PLANTS 122, SYP122.** |

Biotic stress-related genes (Control vs Ln UP)

| | | |
|---|---|---|
| 3,637 | AT1G02450 | **NIM1-INTERACTING 1, NIMIN-1, NIMIN1.** |
| 3,628 | AT1G15520 | **ABCG40, ARABIDOPSIS THALIANA ATP-BINDING CASSETTE G40, ATABCG40, ATP-BINDING CASSETTE G40, ATPDR12, PDR12, PLEIOTROPIC DRUG RESISTANCE 12.** |
| 3,622 | AT2G35980 | **ARABIDOPSIS NDR1/HIN1-LIKE 10, ATNHL10, NDR1/HIN1-LIKE, NHL10, YELLOW-LEAF-SPECIFIC GENE 9, YLS9**. |
| 3,541 | AT1G74100 | **ARABIDOPSIS SULFOTRANSFERASE 5A, ATSOT16, ATST5A, CORI-7, CORONATINE INDUCED-7, SOT16, SULFOTRANSFERASE 16.** |
| 3,501 | AT3G50260 | **ATERF011, CEJ1, COOPERATIVELY REGULATED BY ETHYLENE AND JASMONATE 1, DEAR1, DREB AND EAR MOTIF PROTEIN 1.** |
| 3,459 | AT5G67300 | **ARABIDOPSIS THALIANA MYB DOMAIN PROTEIN 44, ATMYB44, ATMYBR1, MYB DOMAIN PROTEIN R1, MYB44, MYBR1**. |
| 3,426 | AT5G42380 | **CALMODULIN LIKE 37, CALMODULIN LIKE 39, CML37, CML39**. |
| 3,373 | AT4G39030 | **EDS5, ENHANCED DISEASE SUSCEPTIBILITY 5, SALICYLIC ACID INDUCTION DEFICIENT 1, SCORD3, SID1, SUSCEPTIBLE TO CORONATINE-DEFICIENT PST DC3000 3**. |
| 3,264 | AT3G15500 | **ANAC055, ATNAC3, NAC DOMAIN CONTAINING PROTEIN 3, NAC DOMAIN CONTAINING PROTEIN 55, NAC055, NAC3**. |
| 3,193 | AT4G39670 | **Glycolipid transfer protein (GLTP) family protein**. |
| 2,747 | AT4G34150 | **Calcium-dependent lipid-binding domain-containing protein.** |
| 2,594 | AT2G32030 | **Acyl-CoA N-acyltransferases (NAT) superfamily protein**. |
| 2,553 | AT4G24160 | Encodes a soluble **lysophosphatidic acid acyltransferase** with additional triacylglycerol lipase and phosphatidylcholine hydrolyzing enzymatic activities. |
| 2,481 | AT2G47730 | **ARABIDOPSIS THALIANA GLUTATHIONE S-TRANSFERASE PHI 8, ATGSTF5, ATGSTF8, GLUTATHIONE S-TRANSFERASE (CLASS PHI) 5, GLUTATHIONE S-TRANSFERASE PHI 8, GST6, GSTF8**. |
| 2,470 | AT5G52020 | **ERF025**. |
| 2,465 | AT4G33920 | **Putative protein phosphatase 2C 63**. |
| 2,430 | AT5G25930 | **Protein kinase family protein with leucine-rich repeat domain.** |
| 2,422 | AT5G01900 | **ARABIDOPSIS THALIANA WRKY DNA-BINDING PROTEIN 62, ATWRKY62, WRKY DNA-BINDING PROTEIN 62, WRKY62.** |
| 2,415 | AT3G55980 | **ATSZF1, SALT-INDUCIBLE ZINC FINGER 1, SZF1**. |
| 2,386 | AT2G30020 | Encodes **AP2C1**. |
| 2,335 | AT4G31500 | **"CYTOCHROME P450, FAMILY 83, SUBFAMILY B, POLYPEPTIDE 1", ALTERED TRYPTOPHAN REGULATION 4, ATR4, CYP83B1, RED ELONGATED 1, RED1, RNT1, RUNT 1, SUPERROOT 2, SUR2.** |
| 2,278 | AT1G18350 | **ATMKK7, BUD1, BUSHY AND DWARF 1, MAP KINASE KINASE 7, MAP KINASE KINASE7, MKK7**. |
| 2,268 | AT1G18590 | **ARABIDOPSIS SULFOTRANSFERASE 5C, ATSOT17, ATST5C, SOT17, SULFOTRANSFERASE 17.** |
| 2,266 | AT3G11840 | **PLANT U-BOX 24, PUB24**. |
| 2,191 | AT5G42050 | **DCD (Development and Cell Death) domain protein**. |
| 2,126 | AT1G50740 | **Transmembrane proteins 14C**. |
| 2,044 | AT5G22570 | **ARABIDOPSIS THALIANA WRKY DNA-BINDING PROTEIN 38, ATWRKY38, WRKY DNA-BINDING PROTEIN 38, WRKY38.** |
| 2,034 | AT1G80820 | **ATCCR2, CCR2, CINNAMOYL COA REDUCTASE**. |
| 2,010 | AT5G61890 | **ERF114**. |

Biotic stress-related genes (Control vs Ln UP)

| | | |
|---|---|---|
| | | **Detection of biotic stimulus** |
| 26,669 | AT1G80840 | **ATWRKY40, WRKY DNA-BINDING PROTEIN 40, WRKY40.** P |
| 9,711 | AT5G42650 | **ALLENE OXIDE SYNTHASE, AOS, CYP74A, CYTOCHROME P450 74A, DDE2, DELAYED DEHISCENCE 2.** |
| 3,925 | AT3G52400 | **ATSYP122, SYNTAXIN OF PLANTS 122, SYP122.** |
| 3,911 | AT1G32960 | **ATSBT3.3, SBT3.3.** |
| 3,637 | AT1G02450 | **NIM1-INTERACTING 1, NIMIN-1, NIMIN1.** |
| 3,628 | AT1G15520 | **ABCG40, ARABIDOPSIS THALIANA ATP-BINDING CASSETTE G40, ATABCG40, ATP-BINDING CASSETTE G40, ATPDR12, PDR12, PLEIOTROPIC DRUG RESISTANCE 12.** |
| 3,373 | AT4G39030 | **EDS5, ENHANCED DISEASE SUSCEPTIBILITY 5, SALICYLIC ACID INDUCTION DEFICIENT 1, SCORD3, SID1, SUSCEPTIBLE TO CORONATINE-DEFICIENT PST DC3000 3**. |
| 2,266 | AT3G11840 | **PLANT U-BOX 24, PUB24**. |
| 2,044 | AT5G22570 | **ARABIDOPSIS THALIANA WRKY DNA-BINDING PROTEIN 38, ATWRKY38, WRKY DNA-BINDING PROTEIN 38, WRKY38.** |
| | | **Detection of bacterium** |
| 26,669 | AT1G80840 | **ATWRKY40, WRKY DNA-BINDING PROTEIN 40, WRKY40.** |
| 3,637 | AT1G02450 | **NIM1-INTERACTING 1, NIMIN-1, NIMIN1.** |
| 2,044 | AT5G22570 | **ARABIDOPSIS THALIANA WRKY DNA-BINDING PROTEIN 38, ATWRKY38, WRKY DNA-BINDING PROTEIN 38, WRKY38.** |
| | | **Negative regulation of defense response** |
| 26,669 | AT1G80840 | **ATWRKY40, WRKY DNA-BINDING PROTEIN 40, WRKY40.** |
| 11,762 | AT1G28370 | **ATERF11, ERF DOMAIN PROTEIN 11, ERF11.** |
| 9,711 | AT5G42650 | **ALLENE OXIDE SYNTHASE, AOS, CYP74A, CYTOCHROME P450 74A, DDE2, DELAYED DEHISCENCE 2.** |
| 7,910 | AT1G66090 | **Disease resistance protein**. |
| 5,076 | AT2G35930 | **ATPUB23, PLANT U-BOX 23, PUB23**. |
| 4,624 | AT2G05940 | **RIPK, RPM1-INDUCED PROTEIN KINASE**. |
| 3,925 | AT3G52400 | **ATSYP122, SYNTAXIN OF PLANTS 122, SYP122.** |
| 3,637 | AT1G02450 | **NIM1-INTERACTING 1, NIMIN-1, NIMIN1.** |
| 3,628 | AT1G15520 | **ABCG40, ARABIDOPSIS THALIANA ATP-BINDING CASSETTE G40, ATABCG40, ATP-BINDING CASSETTE G40, ATPDR12, PDR12, PLEIOTROPIC DRUG RESISTANCE 12.** |
| 3,622 | AT2G35980 | **ARABIDOPSIS NDR1/HIN1-LIKE 10, ATNHL10, NDR1/HIN1-LIKE, NHL10, YELLOW-LEAF-SPECIFIC GENE 9, YLS9**. |
| 3,615 | AT5G35735 | **Auxin-responsive family protein.** |
| 3,373 | AT4G39030 | **EDS5, ENHANCED DISEASE SUSCEPTIBILITY 5, SALICYLIC ACID INDUCTION DEFICIENT 1, SCORD3, SID1, SUSCEPTIBLE TO CORONATINE-DEFICIENT PST DC3000 3**. |
| 3,264 | AT3G15500 | **ANAC055, ATNAC3, NAC DOMAIN CONTAINING PROTEIN 3, NAC DOMAIN CONTAINING PROTEIN 55, NAC055, NAC3**. |
| 3,193 | AT4G39670 | **Glycolipid transfer protein (GLTP) family protein**. |
| 2,266 | AT3G11840 | **PLANT U-BOX 24, PUB24**. |
| 2,191 | AT5G42050 | **DCD (Development and Cell Death) domain protein**. |
| 2,044 | AT5G22570 | **ARABIDOPSIS THALIANA WRKY DNA-BINDING PROTEIN 38,** |

Biotic stress-related genes (Control vs Ln UP)

| | | |
|---|---|---|
| | | ATWRKY38, WRKY DNA-BINDING PROTEIN 38, WRKY38. |
| | | **Regulation of immune response** |
| 26,669 | AT1G80840 | ATWRKY40, WRKY DNA-BINDING PROTEIN 40, WRKY40. |
| 3,637 | AT1G02450 | NIM1-INTERACTING 1, NIMIN-1, NIMIN1. |
| 2,044 | AT5G22570 | ARABIDOPSIS THALIANA WRKY DNA-BINDING PROTEIN 38, ATWRKY38, WRKY DNA-BINDING PROTEIN 38, WRKY38. |
| | | **Respiratory burst involved in defense response** |
| 30,219 | AT4G17490 | ATERF6, ERF-6-6, ERF6, ETHYLENE RESPONSIVE ELEMENT BINDING FACTOR 6. |
| 26,669 | AT1G80840 | ATWRKY40, WRKY DNA-BINDING PROTEIN 40, WRKY40. |
| 22,102 | AT4G17500 | ATERF-1, ERF-1, ETHYLENE RESPONSIVE ELEMENT BINDING FACTOR 1. |
| 19,423 | AT5G62020 | ARABIDOPSIS THALIANA HEAT SHOCK TRANSCRIPTION FACTOR B2A, AT-HSFB2A, HEAT SHOCK TRANSCRIPTION FACTOR B2A, HSFB2A. |
| 9,223 | AT3G23250 | ATMYB15, ATY19, MYB DOMAIN PROTEIN 15, MYB15. |
| 8,662 | AT5G47230 | ATERF-5, ATERF5, ERF5, ETHYLENE RESPONSIVE ELEMENT BINDING FACTOR 5, ETHYLENE RESPONSIVE ELEMENT BINDING FACTOR- 5. |
| 8,635 | AT3G15210 | ATERF-4, ATERF4, ERF4, ETHYLENE RESPONSIVE ELEMENT BINDING FACTOR 4, RAP2.5, RELATED TO AP2 5. |
| 5,839 | AT5G47220 | ATERF-2, ATERF2, ERF2, ETHYLENE RESPONSE FACTOR- 2, ETHYLENE RESPONSIVE ELEMENT BINDING FACTOR 2. |
| 5,775 | AT1G27730 | SALT TOLERANCE ZINC FINGER, STZ, ZAT10. |
| 5,648 | AT1G66160 | "CYS, MET, PRO, AND GLY PROTEIN 1", ATCMPG1, CMPG1. |
| 5,092 | AT2G37430 | C2H2 and C2HC zinc fingers superfamily protein. |
| 5,076 | AT2G35930 | ATPUB23, PLANT U-BOX 23, PUB23. |
| 4,074 | AT3G19580 | AZF2, ZF2, ZINC-FINGER PROTEIN 2. |
| 3,925 | AT3G52400 | ATSYP122, SYNTAXIN OF PLANTS 122, SYP122. |
| 3,655 | AT1G62300 | ATWRKY6, WRKY6. |
| 3,622 | AT2G35980 | ARABIDOPSIS NDR1/HIN1-LIKE 10, ATNHL10, NDR1/HIN1-LIKE, NHL10, YELLOW-LEAF-SPECIFIC GENE 9, YLS9. |
| 3,534 | AT1G04100 | IAA10, INDOLEACETIC ACID-INDUCED PROTEIN 10. |
| 3,501 | AT3G50260 | ATERF011, CEJ1, COOPERATIVELY REGULATED BY ETHYLENE AND JASMONATE 1, DEAR1, DREB AND EAR MOTIF PROTEIN 1. |
| 3,193 | AT4G39670 | Glycolipid transfer protein (GLTP) family protein. |
| 2,772 | AT5G66070 | NEP1-interacting protein-like 1. |
| 2,594 | AT2G32030 | Acyl-CoA N-acyltransferases (NAT) superfamily protein. |
| 2,430 | AT5G25930 | Protein kinase family protein with leucine-rich repeat domain. |
| 2,415 | AT3G55980 | ATSZF1, SALT-INDUCIBLE ZINC FINGER 1, SZF1. |
| 2,266 | AT3G11840 | PLANT U-BOX 24, PUB24. |
| 2,249 | AT3G52800 | Zinc finger A20 and AN1 domain-containing stress-associated protein 6. |
| 2,213 | AT5G59550 | ARABIDOPSIS THALIANA RING AND DOMAIN OF UNKNOWN FUNCTION 1117 2, ATRDUF2, RDUF2, RING AND DOMAIN OF UNKNOWN FUNCTION 1117 2. |
| | | **Systemic acquired resistance** |

Biotic stress-related genes (Control vs Ln UP)

| | | |
|---|---|---|
| 36,838 | AT5G13220 | **JAS1, JASMONATE-ASSOCIATED 1, JASMONATE-ZIM-DOMAIN PROTEIN 10, JAZ10, TIFY DOMAIN PROTEIN 9, TIFY9**. |
| 26,669 | AT1G80840 | **ATWRKY40, WRKY DNA-BINDING PROTEIN 40, WRKY40.** |
| 24,266 | AT1G44350 | **IAA-LEUCINE RESISTANT (ILR)-LIKE GENE 6, ILL6.** |
| 12,956 | AT5G13220 | **JAS1, JASMONATE-ASSOCIATED 1, JASMONATE-ZIM-DOMAIN PROTEIN 10, JAZ10, TIFY DOMAIN PROTEIN 9, TIFY9**. |
| 11,762 | AT1G28370 | **ATERF11, ERF DOMAIN PROTEIN 11, ERF11.** |
| 9,711 | AT5G42650 | **ALLENE OXIDE SYNTHASE, AOS, CYP74A, CYTOCHROME P450 74A, DDE2, DELAYED DEHISCENCE 2.** |
| 6,509 | AT1G43800 | **FLORAL TRANSITION AT THE MERISTEM1, FTM1**. |
| 5,076 | AT2G35930 | **ATPUB23, PLANT U-BOX 23, PUB23**. |
| 4,624 | AT2G05940 | **RIPK, RPM1-INDUCED PROTEIN KINASE**. |
| 4,397 | AT4G33070 | **Thiamine pyrophosphate dependent pyruvate decarboxylase family protein**. |
| 3,925 | AT3G52400 | **ATSYP122, SYNTAXIN OF PLANTS 122, SYP122.** |
| 3,637 | AT1G02450 | **NIM1-INTERACTING 1, NIMIN-1, NIMIN1.** |
| 3,628 | AT1G15520 | **ABCG40, ARABIDOPSIS THALIANA ATP-BINDING CASSETTE G40, ATABCG40, ATP-BINDING CASSETTE G40, ATPDR12, PDR12, PLEIOTROPIC DRUG RESISTANCE 12.** |
| 3,622 | AT2G35980 | **ARABIDOPSIS NDR1/HIN1-LIKE 10, ATNHL10, NDR1/HIN1-LIKE, NHL10, YELLOW-LEAF-SPECIFIC GENE 9, YLS9**. |
| 3,615 | AT5G35735 | **Auxin-responsive family protein.** |
| 3,373 | AT4G39030 | **EDS5, ENHANCED DISEASE SUSCEPTIBILITY 5, SALICYLIC ACID INDUCTION DEFICIENT 1, SCORD3, SID1, SUSCEPTIBLE TO CORONATINE-DEFICIENT PST DC3000 3**. |
| 3,264 | AT3G15500 | **ANAC055, ATNAC3, NAC DOMAIN CONTAINING PROTEIN 3, NAC DOMAIN CONTAINING PROTEIN 55, NAC055, NAC3**. |
| 2,380 | AT1G05300 | **ZINC TRANSPORTER 5 PRECURSOR, ZIP5**. |
| 2,281 | AT4G01895 | **Systemic acquired resistance (SAR) regulator protein NIMIN-1-related.** |
| 2,278 | AT1G18350 | **ATMKK7, BUD1, BUSHY AND DWARF 1, MAP KINASE KINASE 7, MAP KINASE KINASE7, MKK7**. |
| 2,266 | AT3G11840 | **PLANT U-BOX 24, PUB24**. |
| 2,191 | AT5G42050 | **DCD (Development and Cell Death) domain protein**. |
| 2,044 | AT5G22570 | **ARABIDOPSIS THALIANA WRKY DNA-BINDING PROTEIN 38, ATWRKY38, WRKY DNA-BINDING PROTEIN 38, WRKY38.** |

Biotic stress-related genes (Control vs Ln DOWN)

| FC | ID | Biotic stress |
|---|---|---|
| 2.925 | AT1G56570 | **PENTATRICOPEPTIDE REPEAT PROTEIN FOR GERMINATION ON NACL, PGN.** |
| 2.218 | AT2G40190 | **LEAF WILTING 3, LEW3.** |
| 2.098 | AT1G35515 | **HIGH RESPONSE TO OSMOTIC STRESS 10, HOS10, MYB8.** |
| 2.024 | AT2G33380 | **ARABIDOPSIS THALIANA CALEOSIN 3, ATCLO3, CALEOSIN 3, CLO-3, CLO3, RD20, RESPONSIVE TO DESSICATION 20.** |
| | | **Response to chitin** |
| 7.690 | AT2G36270 | **ABA INSENSITIVE 5, ABI5, GIA1, GROWTH-INSENSITIVITY TO ABA 1**. |
| 7.473 | AT5G67450 | **AZF1, ZF1, ZINC-FINGER PROTEIN 1.** |
| 6.989 | AT5G14740 | **BETA CA2, BETA CARBONIC ANHYDRASE 2, CA18, CA2, CARBONIC ANHYDRASE 18, CARBONIC ANHYDRASE 2.** |
| 6.681 | AT5G59780 | **ATMYB59, ATMYB59-1, ATMYB59-2, ATMYB59-3, MYB DOMAIN PROTEIN 59, MYB59**. |
| 4.160 | AT3G01500 | **ARABIDOPSIS THALIANA SALICYLIC ACID-BINDING PROTEIN 3, ATBCA1, ATSABP3, BETA CARBONIC ANHYDRASE 1, CA1, CARBONIC ANHYDRASE 1, SABP3, SALICYLIC ACID-BINDING PROTEIN 3**. |
| 3.819 | AT1G70140 | **ATFH8, FH8, FORMIN 8**. |
| 3.799 | AT1G74360 | **Leucine-rich repeat protein kinase family protein**. |
| 3.711 | AT1G51790 | **Leucine-rich repeat protein kinase family protein**. |
| 3.658 | AT4G31550 | **ATWRKY11, WRKY DNA-BINDING PROTEIN 11, WRKY11**. |
| 3.544 | AT4G30370 | **RING/U-box superfamily protein**. |
| 3.317 | AT2G19190 | **FLG22-INDUCED RECEPTOR-LIKE KINASE 1, FRK1**. |
| 3.299 | AT2G04240 | **XERICO**. |
| 3.095 | AT5G20480 | **EF-TU RECEPTOR, EFR**. |
| 3.072 | AT2G35000 | **ARABIDOPSIS TOXICOS EN LEVADURA 9, ATL9**. |
| 3.004 | AT1G71697 | **ATCK1, CHOLINE KINASE, CHOLINE KINASE 1, CK, CK1**. |
| 2.808 | AT4G18450 | Encodes a member of the **ERF (ethylene response factor) subfamily B-3 of ERF/AP2 transcription factor family**. |
| 2.786 | AT4G26090 | **RESISTANT TO P. SYRINGAE 2, RPS2.** |
| 2.783 | AT1G26420 | **FAD-binding Berberine family protein.** |
| 2.751 | AT5G38895 | **RING/U-box superfamily protein.** |
| 2.740 | AT5G63320 | **NPX1, NUCLEAR PROTEIN X1**. |
| 2.690 | AT3G61460 | **BRASSINOSTEROID-RESPONSIVE RING-H2, BRH1**. |
| 2.665 | AT4G18950 | **Integrin-linked protein kinase family.** |
| 2.657 | AT2G44490 | **BETA GLUCOSIDASE 26, BGLU26, PEN2, PENETRATION 2.** |
| 2.656 | AT1G61360 | **S-locus lectin protein kinase family protein.** |
| 2.576 | AT1G05010 | **ACO4, EAT1, EFE, ETHYLENE FORMING ENZYME, ETHYLENE-FORMING ENZYME**. |
| 2.572 | AT1G59870 | **ABCG36, ARABIDOPSIS PLEIOTROPIC DRUG RESISTANCE 8, ARABIDOPSIS THALIANA ATP-BINDING CASSETTE G36, ATABCG36, ATP-BINDING CASSETTE G36, ATPDR8, PDR8, PEN3, PENETRATION 3, PLEIOTROPIC DRUG RESISTANCE 8.** |
| 2.555 | AT4G37770 | **1-AMINO-CYCLOPROPANE-1-CARBOXYLATE SYNTHASE 8, ACS8.** |
| 2.549 | AT3G19380 | **PLANT U-BOX 25, PUB25.** |
| 2.502 | AT5G57220 | **"CYTOCHROME P450, FAMILY 81, SUBFAMILY F, POLYPEPTIDE 2", CYP81F2.** |
| 2.407 | AT4G23810 | **ATWRKY53, WRKY53**. |
| 2.359 | AT4G21390 | **B120**. |
| 2.359 | AT1G07520 | **GRAS family transcription factor**. |

Biotic stress-related genes (Control vs Ln DOWN)

| | | |
|---|---|---|
| 2.348 | AT5G05140 | **Transcription elongation factor (TFIIS) family protein.** |
| 2.347 | AT4G23550 | **ATWRKY29, WRKY29**. |
| 2.328 | AT2G28830 | **ATPUB12, PLANT U-BOX 12, PUB12.** |
| 2.297 | AT5G03680 | **PETAL LOSS, PTL.** |
| 2.253 | AT5G01540 | **L-TYPE LECTIN RECEPTOR KINASE-VI.2, LECRK-VI.2, LECRKA4.1, LECTIN RECEPTOR KINASE A4.1.** |
| 2.221 | AT5G63130 | **Octicosapeptide/Phox/Bem1p family protein**. |
| 2.217 | AT3G18710 | **ARABIDOPSIS THALIANA PLANT U-BOX 29, ATPUB29, PLANT U-BOX 29, PUB29.** |
| 2.167 | AT2G17770 | **ATBZIP27, BASIC REGION/LEUCINE ZIPPER MOTIF 27, BZIP27, FD PARALOG, FDP.** |
| 2.150 | AT5G61600 | **ERF104, ETHYLENE RESPONSE FACTOR 104.** |
| 2.085 | AT1G20823 | **RING/U-box superfamily protein. Involved in: response to chitin.** |
| 2.063 | AT4G11360 | **RHA1B, RING-H2 FINGER A1B.** |
| 2.059 | AT3G09010 | **Protein kinase superfamily protein.** |
| 2.044 | AT2G43620 | **Chitinase family protein**. |
| 2.018 | AT3G53180 | **NODGS, NODULIN/GLUTAMINE SYNTHASE-LIKE PROTEIN**. E |
| 2.012 | AT2G23320 | **ATWRKY15, WRKY DNA-BINDING PROTEIN 15, WRKY15.** |
| | | **Response to fungus** |
| 6.989 | AT5G14740 | **BETA CA2, BETA CARBONIC ANHYDRASE 2, CA18, CA2, CARBONIC ANHYDRASE 18, CARBONIC ANHYDRASE 2.** |
| 6.049 | AT2G15890 | **MATERNAL EFFECT EMBRYO ARREST 14, MEE14**. |
| 4.160 | AT3G01500 | **ARABIDOPSIS THALIANA SALICYLIC ACID-BINDING PROTEIN 3, ATBCA1, ATSABP3, BETA CARBONIC ANHYDRASE 1, CA1, CARBONIC ANHYDRASE 1, SABP3, SALICYLIC ACID-BINDING PROTEIN 3**. |
| 3.799 | AT1G74360 | **Leucine-rich repeat protein kinase family protein**. |
| 3.672 | AT4G11650 | **ATOSM34, OSM34, OSMOTIN 34**. |
| 3.518 | AT5G38710 | **Methylenetetrahydrofolate reductase family protein**. |
| 3.482 | AT1G75380 | **ATBBD1, BBD1, BIFUNCTIONAL NUCLEASE IN BASAL DEFENSE RESPONSE 1**. |
| 3.420 | AT3G63010 | **ATGID1B, GA INSENSITIVE DWARF1B, GID1B**. |
| 3.317 | AT2G19190 | **FLG22-INDUCED RECEPTOR-LIKE KINASE 1, FRK1**. |
| 3.095 | AT5G20480 | **EF-TU RECEPTOR, EFR**. |
| 3.072 | AT2G35000 | **ARABIDOPSIS TOXICOS EN LEVADURA 9, ATL9**. |
| 3.061 | AT2G37130 | **Peroxidase superfamily protein**. |
| 3.004 | AT1G71697 | **ATCK1, CHOLINE KINASE, CHOLINE KINASE 1, CK, CK1**. |
| 2.968 | AT2G39210 | **Major facilitator superfamily protein**. |
| 2.925 | AT1G56570 | **PENTATRICOPEPTIDE REPEAT PROTEIN FOR GERMINATION ON NACL, PGN**. |
| 2.866 | AT1G79670 | **RESISTANCE TO FUSARIUM OXYSPORUM 1, RFO1, WAKL22.** |
| 2.786 | AT4G26090 | **RESISTANT TO P. SYRINGAE 2, RPS2.** |
| 2.786 | AT1G07630 | **PLL5, POL-LIKE 5.** |
| 2.697 | AT1G08720 | **ATEDR1, EDR1, ENHANCED DISEASE RESISTANCE 1.** |
| 2.657 | AT2G44490 | **BETA GLUCOSIDASE 26, BGLU26, PEN2, PENETRATION 2.** |
| 2.602 | AT1G63880 | Encodes a **TIR-NBS-LRR class of disease resistance protein** effective against Leptosphaeria maculans. |
| 2.585 | AT4G16950 | **RECOGNITION OF PERONOSPORA PARASITICA 5, RPP5.** |
| 2.576 | AT1G05010 | **ACO4, EAT1, EFE, ETHYLENE FORMING ENZYME, ETHYLENE-FORMING ENZYME**. |
| 2.572 | AT1G59870 | **ABCG36, ARABIDOPSIS PLEIOTROPIC DRUG RESISTANCE 8, ARABIDOPSIS THALIANA** |

Biotic stress-related genes (Control vs Ln DOWN)

| | | |
|---|---|---|
| | | **ATP-BINDING CASSETTE G36, ATABCG36, ATP-BINDING CASSETTE G36, ATPDR8, PDR8, PEN3, PENETRATION 3, PLEIOTROPIC DRUG RESISTANCE 8.** |
| 2.502 | AT5G57220 | **"CYTOCHROME P450, FAMILY 81, SUBFAMILY F, POLYPEPTIDE 2", CYP81F2.** |
| 2.461 | AT2G47900 | **ATTLP3, TLP3, TUBBY LIKE PROTEIN 3.** |
| 2.458 | AT1G20440 | **ATCOR47, COLD-REGULATED 47, COR47, RD17.** |
| 2.434 | AT4G37150 | **ARABIDOPSIS THALIANA METHYL ESTERASE 9, ATMES9, MES9, METHYL ESTERASE 9.** |
| 2.407 | AT4G23810 | **ATWRKY53, WRKY53**. |
| 2.388 | AT2G31260 | **APG9, ATAPG9, AUTOPHAGY 9**. |
| 2.383 | AT1G51680 | **4-COUMARATE:COA LIGASE 1, 4CL.1, 4CL1, ARABIDOPSIS THALIANA 4-COUMARATE:COA LIGASE 1, AT4CL1.** |
| 2.378 | AT5G55390 | **EDM2, ENHANCED DOWNY MILDEW 2.** |
| 2.366 | AT5G67340 | **ARM repeat superfamily protein.** |
| 2.359 | AT4G21390 | **B120**. |
| 2.348 | AT5G05140 | **Transcription elongation factor (TFIIS) family protein.** |
| 2.311 | AT3G11340 | **UDP-DEPENDENT GLYCOSYLTRANSFERASE 76B1, UGT76B1.** |
| 2.259 | AT2G16390 | **CHR35, DEFECTIVE IN MERISTEM SILENCING 1, DEFECTIVE IN RNA-DIRECTED DNA METHYLATION 1, DMS1, DRD1**. |
| 2.258 | AT3G50970 | **LOW TEMPERATURE-INDUCED 30, LTI30, XERO2.** |
| 2.253 | AT5G01540 | **L-TYPE LECTIN RECEPTOR KINASE-VI.2, LECRK-VI.2, LECRKA4.1, LECTIN RECEPTOR KINASE A4.1.** |
| 2.184 | AT5G03280 | **ATEIN2, CKR1, CYTOKININ RESISTANT 1, EIN2, ENHANCED RESPONSE TO ABA3, ERA3, ETHYLENE INSENSITIVE 2, ORE2, ORE3, ORESARA 2, ORESARA 3, PIR2**. |
| 2.175 | AT1G64610 | **Transducin/WD40 repeat-like superfamily protein**. |
| 2.169 | AT1G08050 | **Zinc finger (C3HC4-type RING finger) family protein**. |
| 2.155 | AT5G46350 | **ARABIDOPSIS THALIANA WRKY DNA-BINDING PROTEIN 8, ATWRKY8, WRKY DNA-BINDING PROTEIN 8, WRKY8**. |
| 2.150 | AT5G61600 | **ERF104, ETHYLENE RESPONSE FACTOR 104.** |
| 2.135 | AT5G61420 | **ATMYB28, HAG1, HIGH ALIPHATIC GLUCOSINOLATE 1, MYB DOMAIN PROTEIN 28, MYB28, PMG1, PRODUCTION OF METHIONINE-DERIVED GLUCOSINOLATE 1.** |
| 2.130 | AT1G47128 | **RD21, RD21A, RESPONSIVE TO DEHYDRATION 21, RESPONSIVE TO DEHYDRATION 21A.** |
| 2.064 | AT3G14050 | **AT-RSH2, ATRSH2, RELA-SPOT HOMOLOG 2, RELA/SPOT HOMOLOG 2, RSH2**. |
| 2.061 | AT4G26080 | **ABA INSENSITIVE 1, ABI1, ATABI1**. |
| 2.024 | AT2G33380 | **ARABIDOPSIS THALIANA CALEOSIN 3, ATCLO3, CALEOSIN 3, CLO-3, CLO3, RD20, RESPONSIVE TO DESSICATION 20.** |
| 2.020 | AT3G61710 | **ATATG6, ATBECLIN1, ATG6, AUTOPHAGY 6, BECLIN1.** |
| | | **Defense response & Defense response signaling pathway** |
| 11.125 | AT5G46050 | **ARABIDOPSIS THALIANA PEPTIDE TRANSPORTER 3, ATPTR3, PEPTIDE TRANSPORTER 3, PTR3**. |
| 10.681 | AT2G22330 | **"CYTOCHROME P450, FAMILY 79, SUBFAMILY B, POLYPEPTIDE 3", CYP79B3**. |
| 7.798 | AT5G52830 | **ARABIDOPSIS THALIANA WRKY DNA-BINDING PROTEIN 27, ATWRKY27, WRKY DNA-BINDING PROTEIN 27, WRKY27**. |
| 6.989 | AT5G14740 | **BETA CA2, BETA CARBONIC ANHYDRASE 2, CA18, CA2, CARBONIC ANHYDRASE 18, CARBONIC ANHYDRASE 2.** |
| 6.964 | AT1G58390 | **Disease resistance protein (CC-NBS-LRR class) family.** |
| 6.852 | AT5G41070 | **DRB5, DSRNA-BINDING PROTEIN 5.** |

Biotic stress-related genes (Control vs Ln DOWN)

| | | |
|---|---|---|
| 6.491 | AT2G01830 | **AHK4, ARABIDOPSIS HISTIDINE KINASE 4, ATCRE1, CRE1, CYTOKININ RESPONSE 1, WOL, WOL1, WOODEN LEG, WOODEN LEG 1.** |
| 6.049 | AT2G15890 | **MATERNAL EFFECT EMBRYO ARREST 14, MEE14**. |
| 5.903 | AT1G42560 | **ARABIDOPSIS THALIANA MILDEW RESISTANCE LOCUS O 9, ATMLO9, MILDEW RESISTANCE LOCUS O 9, MLO9.** |
| 5.874 | AT5G60410 | **ATSIZ1, SIZ1**. |
| 5.432 | AT5G14740 | **BETA CA2, BETA CARBONIC ANHYDRASE 2, CA18, CA2, CARBONIC ANHYDRASE 18, CARBONIC ANHYDRASE 2**. |
| 5.144 | AT3G22400 | **ARABIDOPSIS THALIANA LIPOXYGENASE 5, ATLOX5, LOX5**. |
| 5.022 | AT5G45800 | **MATERNAL EFFECT EMBRYO ARREST 62, MEE62**. |
| 4.946 | AT2G15890 | **MATERNAL EFFECT EMBRYO ARREST 14, MEE14**. |
| 4.877 | AT5G38340 | **Disease resistance protein (TIR-NBS-LRR class) family**. |
| 4.387 | AT1G02860 | **BAH1, BENZOIC ACID HYPERSENSITIVE 1, NITROGEN LIMITATION ADAPTATION, NLA**. |
| 4.255 | AT2G46370 | **ATGH3.11, FAR-RED INSENSITIVE 219, FIN219, JAR1, JASMONATE RESISTANT 1**. |
| 4.160 | AT3G01500 | **ARABIDOPSIS THALIANA SALICYLIC ACID-BINDING PROTEIN 3, ATBCA1, ATSABP3, BETA CARBONIC ANHYDRASE 1, CA1, CARBONIC ANHYDRASE 1, SABP3, SALICYLIC ACID-BINDING PROTEIN 3**. |
| 4.090 | AT2G31070 | **TCP DOMAIN PROTEIN 10, TCP10**. |
| 4.061 | AT4G08850 | **Leucine-rich repeat receptor-like protein kinase family protein**. |
| 3.819 | AT1G70140 | **ATFH8, FH8, FORMIN 8**. |
| 3.799 | AT1G74360 | **Leucine-rich repeat protein kinase family protein**. |
| 3.781 | AT2G02220 | **ATPSKR1, PHYTOSULFOKIN RECEPTOR 1, PSKR1**. |
| 3.755 | AT3G11660 | **NDR1/HIN1-LIKE 1, NHL1**. |
| 3.711 | AT1G51790 | **Leucine-rich repeat protein kinase family protein**. |
| 3.674 | AT2G14080 | **Disease resistance protein (TIR-NBS-LRR class) family**. |
| 3.672 | AT4G11650 | **ATOSM34, OSM34, OSMOTIN 34**. |
| 3.658 | AT4G31550 | **ATWRKY11, WRKY DNA-BINDING PROTEIN 11, WRKY11**. |
| 3.649 | AT2G19990 | **PATHOGENESIS-RELATED PROTEIN-1-LIKE, PR-1-LIKE**. |
| 3.567 | AT4G31550 | **ATWRKY11, WRKY DNA-BINDING PROTEIN 11, WRKY11**. |
| 3.482 | AT1G75380 | **ATBBD1, BBD1, BIFUNCTIONAL NUCLEASE IN BASAL DEFENSE RESPONSE 1**. |
| 3.420 | AT3G63010 | **ATGID1B, GA INSENSITIVE DWARF1B, GID1B**. |
| 3.377 | AT5G04230 | **ATPAL3, PAL3, PHENYL ALANINE AMMONIA-LYASE 3.** |
| 3.317 | AT2G19190 | **FLG22-INDUCED RECEPTOR-LIKE KINASE 1, FRK1**. |
| 3.309 | ATCG00050 | **RIBOSOMAL PROTEIN S16, RPS16.** |
| 3.269 | AT5G60410 | **ATSIZ1, SIZ1.** |
| 3.119 | AT5G51910 | **TCP family transcription factor**. |
| 3.095 | AT5G20480 | **EF-TU RECEPTOR, EFR**. |
| 3.072 | AT2G35000 | **ARABIDOPSIS TOXICOS EN LEVADURA 9, ATL9**. |
| 3.061 | AT2G37130 | **Peroxidase superfamily protein**. |
| 3.058 | AT3G49860 | **ADP-RIBOSYLATION FACTOR-LIKE A1B, ARLA1B, ATARLA1B.** |
| 3.058 | AT3G43300 | **ATMIN7, BEN1, BFA-VISUALIZED ENDOCYTIC TRAFFICKING DEFECTIVE1, HOPM INTERACTOR 7.** |
| 3.053 | AT1G53230 | **TCP3, TEOSINTE BRANCHED 1, CYCLOIDEA AND PCF TRANSCRIPTION FACTOR 3.** |
| 3.047 | ATCG01090 | **NDHI.** |
| 2.999 | AT4G23320 | **CRK24, CYSTEINE-RICH RLK (RECEPTOR-LIKE PROTEIN KINASE) 24.** |
| 2.973 | AT5G48620 | **Disease resistance protein (CC-NBS-LRR class) family**. |

Biotic stress-related genes (Control vs Ln DOWN)

| | | |
|---|---|---|
| 2.968 | AT2G39210 | **Major facilitator superfamily protein**. |
| 2.925 | AT1G56570 | **PENTATRICOPEPTIDE REPEAT PROTEIN FOR GERMINATION ON NACL, PGN**. |
| 2.863 | AT4G12010 | **Disease resistance protein (TIR-NBS-LRR class) family**. |
| 2.837 | AT5G46270 | **Disease resistance protein (TIR-NBS-LRR class) family.** |
| 2.786 | AT4G26090 | **RESISTANT TO P. SYRINGAE 2, RPS2.** |
| 2.786 | AT1G07630 | **PLL5, POL-LIKE 5.** |
| 2.783 | AT1G26420 | **FAD-binding Berberine family protein.** |
| 2.723 | AT5G04140 | **FD-GOGAT, FERREDOXIN-DEPENDENT GLUTAMATE SYNTHASE, FERREDOXIN-DEPENDENT GLUTAMATE SYNTHASE 1, GLS1, GLU1, GLUS, GLUTAMATE SYNTHASE 1.** |
| 2.718 | AT1G53350 | **Disease resistance protein (CC-NBS-LRR class) family**. |
| 2.697 | AT1G08720 | **ATEDR1, EDR1, ENHANCED DISEASE RESISTANCE 1.** |
| 2.673 | AT3G45290 | **ATMLO3, MILDEW RESISTANCE LOCUS O 3, MLO3.** |
| 2.657 | AT2G44490 | **BETA GLUCOSIDASE 26, BGLU26, PEN2, PENETRATION 2.** |
| 2.656 | AT1G61360 | **S-locus lectin protein kinase family protein.** |
| 2.640 | AT5G65970 | **ATMLO10, MILDEW RESISTANCE LOCUS O 10, MLO10.** |
| 2.632 | AT4G19050 | **NB-ARC domain-containing disease resistance protein.** |
| 2.615 | AT4G36550 | **ARM repeat superfamily protein.** |
| 2.602 | AT1G63880 | Encodes a **TIR-NBS-LRR class of disease resistance protein** effective against Leptosphaeria maculans. |
| 2.585 | AT4G16950 | **RECOGNITION OF PERONOSPORA PARASITICA 5, RPP5.** |
| 2.583 | AT1G27180 | **Disease resistance protein (TIR-NBS-LRR class), putative**. |
| 2.576 | AT1G05010 | **ACO4, EAT1, EFE, ETHYLENE FORMING ENZYME, ETHYLENE-FORMING ENZYME**. |
| 2.576 | ATCG01100 | **NDHA**. |
| 2.572 | AT1G59870 | **ABCG36, ARABIDOPSIS PLEIOTROPIC DRUG RESISTANCE 8, ARABIDOPSIS THALIANA ATP-BINDING CASSETTE G36, ATABCG36, ATP-BINDING CASSETTE G36, ATPDR8, PDR8, PEN3, PENETRATION 3, PLEIOTROPIC DRUG RESISTANCE 8.** |
| 2.561 | AT1G15890 | **Disease resistance protein (CC-NBS-LRR class) family**. |
| 2.559 | AT5G65210 | **TGA1, TGACG SEQUENCE-SPECIFIC BINDING PROTEIN 1.** |
| 2.540 | AT1G72950 | **Disease resistance protein (TIR-NBS class).** |
| 2.534 | AT3G05660 | **ATRLP33, RECEPTOR LIKE PROTEIN 33, RLP33**. |
| 2.530 | AT1G63740 | **Disease resistance protein (TIR-NBS-LRR class) family.** |
| 2.529 | AT1G19660 | **ATBBD2, BBD2, BIFUNCTIONAL NUCLEASE IN BASAL DEFENSE RESPONSE 2**. |
| 2.512 | AT4G37560 | **Acetamidase/Formamidase family protein**. |
| 2.502 | AT5G57220 | **"CYTOCHROME P450, FAMILY 81, SUBFAMILY F, POLYPEPTIDE 2", CYP81F2.** |
| 2.485 | AT1G27170 | **Transmembrane receptors;ATP binding.** |
| 2.482 | AT1G27320 | **AHK3, HISTIDINE KINASE 3, HK3.** |
| 2.462 | AT4G09430 | **Disease resistance protein (TIR-NBS-LRR class) family.** |
| 2.458 | AT1G20440 | **ATCOR47, COLD-REGULATED 47, COR47, RD17.** |
| 2.439 | AT5G13530 | **KEEP ON GOING, KEG.** |
| 2.434 | AT4G37150 | **ARABIDOPSIS THALIANA METHYL ESTERASE 9, ATMES9, MES9, METHYL ESTERASE 9.** |
| 2.417 | AT1G10210 | **ATMPK1, MITOGEN-ACTIVATED PROTEIN KINASE 1, MPK1.** |
| 2.407 | AT4G23810 | **ATWRKY53, WRKY53**. |
| 2.405 | AT5G17680 | **Disease resistance protein (TIR-NBS-LRR class), putative.** |
| 2.401 | AT5G47260 | **ATP binding;GTP binding;nucleotide binding;nucleoside-triphosphatases**. |
| 2.392 | AT2G27170 | **SMC3, STRUCTURAL MAINTENANCE OF CHROMOSOMES 3, TITAN7, TTN7**. |

Biotic stress-related genes (Control vs Ln DOWN)

| | | |
|---|---|---|
| 2.388 | AT2G31260 | **APG9, ATAPG9, AUTOPHAGY 9**. |
| 2.382 | AT4G29140 | **ACTIVATED DISEASE SUSCEPTIBILITY 1, ADS1**. |
| 2.379 | AT1G63730 | **Disease resistance protein (TIR-NBS-LRR class) family.** |
| 2.378 | AT5G55390 | **EDM2, ENHANCED DOWNY MILDEW 2.** |
| 2.375 | AT4G16890 | **BAL, BALL, SNC1, SUPPRESSOR OF NPR1-1, CONSTITUTIVE 1.** |
| 2.366 | AT5G67340 | **ARM repeat superfamily protein.** |
| 2.359 | AT4G21390 | **B120**. |
| 2.359 | AT1G07520 | **GRAS family transcription factor**. |
| 2.357 | AT4G39090 | **RD19, RD19A, RESPONSIVE TO DEHYDRATION 19, RESPONSIVE TO DEHYDRATION 19A.** |
| 2.350 | AT4G08480 | **MAPK/ERK KINASE KINASE 2, MAPKKK9, MEKK2, MITOGEN-ACTIVATED PROTEIN KINASE KINASE KINASE 9, SUMM1, SUPPRESSOR OF MKK1 MKK2 1.** |
| 2.348 | AT3G26910 | **Hydroxyproline-rich glycoprotein family protein.** |
| 2.340 | AT5G48600 | **ARABIDOPSIS THALIANA CHROMOSOME ASSOCIATED PROTEIN-C, ARABIDOPSIS THALIANA STRUCTURAL MAINTENANCE OF CHROMOSOME 4, ATCAP-C, ATSMC3, ATSMC4, SMC3, STRUCTURAL MAINTENANCE OF CHROMOSOME 3.** |
| 2.335 | AT3G03300 | **ATDCL2, DCL2, DICER-LIKE 2**. |
| 2.328 | AT4G15415 | **ATB' GAMMA.** |
| 2.325 | AT5G45060 | **Disease resistance protein (TIR-NBS-LRR class) family.** |
| 2.321 | AT5G55310 | **DNA TOPOISOMERASE 1 BETA, TOP1, TOP1BETA, TOPOISOMERASE 1.** |
| 2.318 | AT5G11250 | **Disease resistance protein (TIR-NBS-LRR class)**. |
| 2.311 | AT3G11340 | **UDP-DEPENDENT GLYCOSYLTRANSFERASE 76B1, UGT76B1.** |
| 2.304 | AT4G00020 | **BRCA2(IV), BRCA2A, BREAST CANCER 2 LIKE 2A, EDA20, EMBRYO SAC DEVELOPMENT ARREST 20, MATERNAL EFFECT EMBRYO ARREST 43, MEE43.** |
| 2.302 | AT2G04430 | **ATNUDT5, NUDIX HYDROLASE HOMOLOG 5, NUDT5**. |
| 2.285 | AT5G46450 | **Disease resistance protein (TIR-NBS-LRR class) family**. |
| 2.282 | AT3G25070 | **RIN4, RPM1 INTERACTING PROTEIN 4.** |
| 2.282 | AT5G43470 | **HRT, HYPERSENSITIVE RESPONSE TO TCV, RCY1, RECOGNITION OF PERONOSPORA PARASITICA 8, RESISTANT TO CMV(Y) 1, RPP8.** |
| 2.276 | AT2G33340 | **MAC3B, MOS4-ASSOCIATED COMPLEX 3B**. |
| 2.262 | AT5G52100 | **CHLORORESPIRATION REDUCTION 1, CRR1.** |
| 2.259 | AT2G16390 | **CHR35, DEFECTIVE IN MERISTEM SILENCING 1, DEFECTIVE IN RNA-DIRECTED DNA METHYLATION 1, DMS1, DRD1**. |
| 2.258 | AT3G50970 | **LOW TEMPERATURE-INDUCED 30, LTI30, XERO2.** |
| 2.253 | AT3G23120 | **ATRLP38, RECEPTOR LIKE PROTEIN 38, RLP38**. |
| 2.253 | AT5G01540 | **L-TYPE LECTIN RECEPTOR KINASE-VI.2, LECRK-VI.2, LECRKA4.1, LECTIN RECEPTOR KINASE A4.1.** |
| 2.251 | AT3G43920 | **ATDCL3, DCL3, DICER-LIKE 3**. |
| 2.250 | AT3G12810 | **CHR13, PHOTOPERIOD-INDEPENDENT EARLY FLOWERING 1, PIE1, SRCAP**. |
| 2.250 | AT3G07040 | **RESISTANCE TO P. SYRINGAE PV MACULICOLA 1, RESISTANCE TO PSEUDOMONAS SYRINGAE 3, RPM1, RPS3.** |
| 2.245 | AT1G59620 | **CW9.** |
| 2.244 | AT1G34750 | **Protein phosphatase 2C family protein**. |
| 2.238 | AT5G43730 | **Disease resistance protein (CC-NBS-LRR class) family**. |
| 2.234 | AT1G66980 | **GDPDL2, GLYCEROPHOSPHODIESTER PHOSPHODIESTERASE (GDPD) LIKE 2, SNC4, SUPPRESSOR OF NPR1-1 CONSTITUTIVE 4**. |
| 2.231 | AT4G31750 | **HOPW1-1-INTERACTING 2, WIN2.** |
| 2.220 | AT4G14465 | **AHL20, AT-HOOK MOTIF NUCLEAR-LOCALIZED PROTEIN 20**. |

Biotic stress-related genes (Control vs Ln DOWN)

| | | |
|---|---|---|
| 2.203 | AT3G51560 | **Disease resistance protein (TIR-NBS-LRR class) family**. |
| 2.202 | AT5G38850 | **Disease resistance protein (TIR-NBS-LRR class).** |
| 2.199 | AT4G23690 | **ARABIDOPSIS THALIANA DIRIGENT PROTEIN 6, ATDIR6, DIR6, DIRIGENT PROTEIN 6.** |
| 2.195 | AT2G33050 | **ATRLP26, RECEPTOR LIKE PROTEIN 26, RLP26**. |
| 2.184 | AT5G03280 | **ATEIN2, CKR1, CYTOKININ RESISTANT 1, EIN2, ENHANCED RESPONSE TO ABA3, ERA3, ETHYLENE INSENSITIVE 2, ORE2, ORE3, ORESARA 2, ORESARA 3, PIR2**. |
| 2.175 | AT1G64610 | **Transducin/WD40 repeat-like superfamily protein**. |
| 2.169 | AT1G08050 | **Zinc finger (C3HC4-type RING finger) family protein**. |
| 2.165 | AT5G47010 | **ATUPF1, LBA1, LOW-LEVEL BETA-AMYLASE 1, UPF1.** |
| 2.155 | AT5G46350 | **ARABIDOPSIS THALIANA WRKY DNA-BINDING PROTEIN 8, ATWRKY8, WRKY DNA-BINDING PROTEIN 8, WRKY8**. |
| 2.155 | AT1G11260 | **ATSTP1, STP1, SUGAR TRANSPORTER 1.** |
| 2.154 | AT4G02410 | **ATLPK1, LECTIN-LIKE PROTEIN KINASE 1, LPK1**. |
| 2.150 | AT5G61600 | **ERF104, ETHYLENE RESPONSE FACTOR 104.** |
| 2.138 | AT4G26850 | **VITAMIN C DEFECTIVE 2, VTC2**. |
| 2.135 | AT5G61420 | **ATMYB28, HAG1, HIGH ALIPHATIC GLUCOSINOLATE 1, MYB DOMAIN PROTEIN 28, MYB28, PMG1, PRODUCTION OF METHIONINE-DERIVED GLUCOSINOLATE 1.** |
| 2.130 | AT1G47128 | **RD21, RD21A, RESPONSIVE TO DEHYDRATION 21, RESPONSIVE TO DEHYDRATION 21A.** |
| 2.117 | AT3G49500 | **RDR6, RNA-DEPENDENT RNA POLYMERASE 6, SDE1, SGS2, SILENCING DEFECTIVE 1, SUPPRESSOR OF GENE SILENCING 2.** |
| 2.112 | AT3G23700 | **Nucleic acid-binding proteins superfamily**. |
| 2.111 | AT1G72860 | **Disease resistance protein (TIR-NBS-LRR class) family**. |
| 2.110 | AT1G34420 | **Leucine-rich repeat transmembrane protein kinase family protein**. |
| 2.079 | AT5G40060 | **Disease resistance protein (NBS-LRR class) family**. |
| 2.079 | AT4G11850 | **MATERNAL EFFECT EMBRYO ARREST 54, MEE54, PHOSPHOLIPASE D GAMMA 1, PLDGAMMA1.** |
| 2.078 | AT1G80460 | **GLI1, NHO1, NONHOST RESISTANCE TO P. S. PHASEOLICOLA 1**. |
| 2.077 | AT5G45250 | **RESISTANT TO P. SYRINGAE 4, RPS4.** |
| 2.076 | AT5G20320 | **ATDCL4, DCL4, DICER-LIKE 4.** |
| 2.074 | AT4G16920 | **Disease resistance protein (TIR-NBS-LRR class) family**. |
| 2.063 | AT1G35710 | **Protein kinase family protein with leucine-rich repeat domain**. |
| 2.061 | AT4G26080 | **ABA INSENSITIVE 1, ABI1, ATABI1**. |
| 2.056 | AT4G38550 | **Arabidopsis phospholipase-like protein (PEARLI 4) family**. |
| 2.044 | AT2G43620 | **Chitinase family protein**. |
| 2.043 | AT1G12280 | **SUMM2, SUPPRESSOR OF MKK1 MKK2 2.** |
| 2.032 | AT4G12560 | **CONSTITUTIVE EXPRESSER OF PR GENES 1, CONSTITUTIVE EXPRESSER OF PR GENES 30, CPR1, CPR30.** |
| 2.030 | AT5G04230 | **ATPAL3, PAL3, PHENYL ALANINE AMMONIA-LYASE 3.** |
| 2.024 | AT1G31280 | **AGO2, ARGONAUTE 2, ATAGO2.** |
| 2.020 | AT1G75460 | **ATP-dependent protease La (LON) domain protein**. |
| 2.020 | AT3G61710 | **ATATG6, ATBECLIN1, ATG6, AUTOPHAGY 6, BECLIN1.** |
| 2.018 | AT3G53180 | **NODGS, NODULIN/GLUTAMINE SYNTHASE-LIKE PROTEIN**. |
| 2.012 | AT2G23320 | **ATWRKY15, WRKY DNA-BINDING PROTEIN 15, WRKY15.** |
| 2.007 | AT4G19510 | **Disease resistance protein (TIR-NBS-LRR class)**. |
| 2.001 | AT3G05370 | **ATRLP31, RECEPTOR LIKE PROTEIN 31, RLP31.** |
| 2.001 | AT4G19500 | **Nucleoside-triphosphatases;transmembrane receptors;nucleotide binding;ATP** |

| | | |
|---|---|---|
| | | **binding.** |
| 2.000 | AT3G09260 | **BGLU23, LEB, LONG ER BODY, PSR3.1, PYK10.** |
| 2.000 | AT3G46710 | **NB-ARC domain-containing disease resistance protein.** |
| | | **Detection of biotic stimulus** |
| 6.989 | AT5G14740 | **BETA CA2, BETA CARBONIC ANHYDRASE 2, CA18, CA2, CARBONIC ANHYDRASE 18, CARBONIC ANHYDRASE 2.** |
| 4.160 | AT3G01500 | **ARABIDOPSIS THALIANA SALICYLIC ACID-BINDING PROTEIN 3, ATBCA1, ATSABP3, BETA CARBONIC ANHYDRASE 1, CA1, CARBONIC ANHYDRASE 1, SABP3, SALICYLIC ACID-BINDING PROTEIN 3.** |
| 3.799 | AT1G74360 | **Leucine-rich repeat protein kinase family protein.** |
| 3.317 | AT2G19190 | **FLG22-INDUCED RECEPTOR-LIKE KINASE 1, FRK1.** |
| 3.095 | AT5G20480 | **EF-TU RECEPTOR, EFR.** |
| 2.786 | AT4G26090 | **RESISTANT TO P. SYRINGAE 2, RPS2.** |
| 2.657 | AT2G44490 | **BETA GLUCOSIDASE 26, BGLU26, PEN2, PENETRATION 2.** |
| 2.576 | AT1G05010 | **ACO4, EAT1, EFE, ETHYLENE FORMING ENZYME, ETHYLENE-FORMING ENZYME.** |
| 2.572 | AT1G59870 | **ABCG36, ARABIDOPSIS PLEIOTROPIC DRUG RESISTANCE 8, ARABIDOPSIS THALIANA ATP-BINDING CASSETTE G36, ATABCG36, ATP-BINDING CASSETTE G36, ATPDR8, PDR8, PEN3, PENETRATION 3, PLEIOTROPIC DRUG RESISTANCE 8.** |
| 2.407 | AT4G23810 | **ATWRKY53, WRKY53.** |
| 2.253 | AT5G01540 | **L-TYPE LECTIN RECEPTOR KINASE-VI.2, LECRK-VI.2, LECRKA4.1, LECTIN RECEPTOR KINASE A4.1.** |
| 2.059 | AT3G09010 | **Protein kinase superfamily protein.** |
| 2.012 | AT1G10340 | **Ankyrin repeat family protein.** |
| | | **Detection of bacterium** |
| 3.317 | AT2G19190 | **FLG22-INDUCED RECEPTOR-LIKE KINASE 1, FRK1.** |
| 3.095 | AT5G20480 | **EF-TU RECEPTOR, EFR.** |
| 2.786 | AT4G26090 | **RESISTANT TO P. SYRINGAE 2, RPS2.** |
| | | **Negative regulation of defense response** |
| 6.989 | AT5G14740 | **BETA CA2, BETA CARBONIC ANHYDRASE 2, CA18, CA2, CARBONIC ANHYDRASE 18, CARBONIC ANHYDRASE 2.** |
| 5.144 | AT3G22400 | **ARABIDOPSIS THALIANA LIPOXYGENASE 5, ATLOX5, LOX5.** |
| 4.255 | AT2G46370 | **ATGH3.11, FAR-RED INSENSITIVE 219, FIN219, JAR1, JASMONATE RESISTANT 1.** |
| 4.160 | AT3G01500 | **ARABIDOPSIS THALIANA SALICYLIC ACID-BINDING PROTEIN 3, ATBCA1, ATSABP3, BETA CARBONIC ANHYDRASE 1, CA1, CARBONIC ANHYDRASE 1, SABP3, SALICYLIC ACID-BINDING PROTEIN 3.** |
| 4.090 | AT2G31070 | **TCP DOMAIN PROTEIN 10, TCP10.** |
| 3.799 | AT1G74360 | **Leucine-rich repeat protein kinase family protein.** |
| 3.420 | AT3G63010 | **ATGID1B, GA INSENSITIVE DWARF1B, GID1B.** |
| 3.317 | AT2G19190 | **FLG22-INDUCED RECEPTOR-LIKE KINASE 1, FRK1.** |
| 3.095 | AT5G20480 | **EF-TU RECEPTOR, EFR.** |
| 3.053 | AT1G53230 | **TCP3, TEOSINTE BRANCHED 1, CYCLOIDEA AND PCF TRANSCRIPTION FACTOR 3.** |
| 2.786 | AT4G26090 | **RESISTANT TO P. SYRINGAE 2, RPS2.** |
| 2.786 | AT1G07630 | **PLL5, POL-LIKE 5.** |
| 2.657 | AT2G44490 | **BETA GLUCOSIDASE 26, BGLU26, PEN2, PENETRATION 2.** |
| 2.576 | AT1G05010 | **ACO4, EAT1, EFE, ETHYLENE FORMING ENZYME, ETHYLENE-FORMING ENZYME.** |

Biotic stress-related genes (Control vs Ln DOWN)

| | | |
|---|---|---|
| 2.572 | AT1G59870 | **ABCG36, ARABIDOPSIS PLEIOTROPIC DRUG RESISTANCE 8, ARABIDOPSIS THALIANA ATP-BINDING CASSETTE G36, ATABCG36, ATP-BINDING CASSETTE G36, ATPDR8, PDR8, PEN3, PENETRATION 3, PLEIOTROPIC DRUG RESISTANCE 8.** |
| 2.534 | AT3G05660 | **ATRLP33, RECEPTOR LIKE PROTEIN 33, RLP33**. |
| 2.417 | AT1G10210 | **ATMPK1, MITOGEN-ACTIVATED PROTEIN KINASE 1, MPK1.** |
| 2.407 | AT4G23810 | **ATWRKY53, WRKY53**. |
| 2.382 | AT4G29140 | **ACTIVATED DISEASE SUSCEPTIBILITY 1, ADS1**. |
| 2.366 | AT5G67340 | **ARM repeat superfamily protein.** |
| 2.328 | AT4G15415 | **ATB' GAMMA.** |
| 2.302 | AT2G04430 | **ATNUDT5, NUDIX HYDROLASE HOMOLOG 5, NUDT5**. |
| 2.253 | AT5G01540 | **L-TYPE LECTIN RECEPTOR KINASE-VI.2, LECRK-VI.2, LECRKA4.1, LECTIN RECEPTOR KINASE A4.1.** |
| 2.184 | AT5G03280 | **ATEIN2, CKR1, CYTOKININ RESISTANT 1, EIN2, ENHANCED RESPONSE TO ABA3, ERA3, ETHYLENE INSENSITIVE 2, ORE2, ORE3, ORESARA 2, ORESARA 3, PIR2**. |
| 2.175 | AT1G64610 | **Transducin/WD40 repeat-like superfamily protein**. |
| 2.169 | AT1G08050 | **Zinc finger (C3HC4-type RING finger) family protein**. |
| 2.154 | AT4G02410 | **ATLPK1, LECTIN-LIKE PROTEIN KINASE 1, LPK1**. |
| 2.110 | AT1G34420 | **Leucine-rich repeat transmembrane protein kinase family protein**. |
| 2.061 | AT4G26080 | **ABA INSENSITIVE 1, ABI1, ATABI1**. |
| 2.032 | AT4G12560 | **CONSTITUTIVE EXPRESSER OF PR GENES 1, CONSTITUTIVE EXPRESSER OF PR GENES 30, CPR1, CPR30.** |
| 2.020 | AT1G75460 | **ATP-dependent protease La (LON) domain protein**. |
| 2.000 | AT3G09260 | **BGLU23, LEB, LONG ER BODY, PSR3.1, PYK10.** |
| | | **Regulation of immune response** |
| 3.317 | AT2G19190 | **FLG22-INDUCED RECEPTOR-LIKE KINASE 1, FRK1**. |
| 3.095 | AT5G20480 | **EF-TU RECEPTOR, EFR**. |
| 2.786 | AT4G26090 | **RESISTANT TO P. SYRINGAE 2, RPS2.** |
| | | **Respiratory burst involved in defense response** |
| 3.819 | AT1G70140 | **ATFH8, FH8, FORMIN 8**. |
| 3.799 | AT1G74360 | **Leucine-rich repeat protein kinase family protein.** |
| 3.711 | AT1G51790 | **Leucine-rich repeat protein kinase family protein.** |
| 3.658 | AT4G31550 | **ATWRKY11, WRKY DNA-BINDING PROTEIN 11, WRKY11**. |
| 2.783 | AT1G26420 | **FAD-binding Berberine family protein.** |
| 2.656 | AT1G61360 | **S-locus lectin protein kinase family protein.** |
| 2.359 | AT4G21390 | **B120**. |
| 2.359 | AT1G07520 | **GRAS family transcription factor**. |
| 2.044 | AT2G43620 | **Chitinase family protein**. |
| 2.018 | AT3G53180 | **NODGS, NODULIN/GLUTAMINE SYNTHASE-LIKE PROTEIN**. |
| 2.012 | AT2G23320 | **ATWRKY15, WRKY DNA-BINDING PROTEIN 15, WRKY15.** |
| | | **Systemic acquired resistance** |
| 10.681 | AT2G22330 | **"CYTOCHROME P450, FAMILY 79, SUBFAMILY B, POLYPEPTIDE 3", CYP79B3**. |
| 6.989 | AT5G14740 | **BETA CA2, BETA CARBONIC ANHYDRASE 2, CA18, CA2, CARBONIC ANHYDRASE 18, CARBONIC ANHYDRASE 2.** |
| 5.874 | AT5G60410 | **ATSIZ1, SIZ1**. |

Biotic stress-related genes (Control vs Ln DOWN)

| | | |
|---|---|---|
| 5.432 | AT5G14740 | **BETA CA2, BETA CARBONIC ANHYDRASE 2, CA18, CA2, CARBONIC ANHYDRASE 18, CARBONIC ANHYDRASE 2**. |
| 5.244 | AT2G47240 | **CER8, ECERIFERUM 8, LACS1, LONG-CHAIN ACYL-COA SYNTHASE 1**. |
| 5.022 | AT5G45800 | **MATERNAL EFFECT EMBRYO ARREST 62, MEE62**. |
| 4.387 | AT1G02860 | **BAH1, BENZOIC ACID HYPERSENSITIVE 1, NITROGEN LIMITATION ADAPTATION, NLA**. |
| 4.255 | AT2G46370 | **ATGH3.11, FAR-RED INSENSITIVE 219, FIN219, JAR1, JASMONATE RESISTANT 1**. |
| 4.207 | AT3G60690 | **SAUR-like auxin-responsive protein family.** |
| 4.160 | AT3G01500 | **ARABIDOPSIS THALIANA SALICYLIC ACID-BINDING PROTEIN 3, ATBCA1, ATSABP3, BETA CARBONIC ANHYDRASE 1, CA1, CARBONIC ANHYDRASE 1, SABP3, SALICYLIC ACID-BINDING PROTEIN 3**. |
| 4.090 | AT2G31070 | **TCP DOMAIN PROTEIN 10, TCP10**. |
| 4.061 | AT4G08850 | **Leucine-rich repeat receptor-like protein kinase family protein**. |
| 3.799 | AT1G74360 | **Leucine-rich repeat protein kinase family protein**. |
| 3.420 | AT3G63010 | **ATGID1B, GA INSENSITIVE DWARF1B, GID1B**. |
| 3.317 | AT2G19190 | **FLG22-INDUCED RECEPTOR-LIKE KINASE 1, FRK1**. |
| 3.269 | AT5G60410 | **ATSIZ1, SIZ1.** |
| 3.176 | AT5G62150 | **Peptidoglycan-binding LysM domain-containing protein.** |
| 3.171 | AT4G27450 | **Aluminium induced protein with YGL and LRDR motifs**. |
| 3.115 | AT2G29120 | **ATGLR2.7, GLR2.7, GLUTAMATE RECEPTOR 2.7, GLUTAMATE RECEPTOR 2.7**. |
| 3.095 | AT5G20480 | **EF-TU RECEPTOR, EFR**. |
| 3.053 | AT1G53230 | **TCP3, TEOSINTE BRANCHED 1, CYCLOIDEA AND PCF TRANSCRIPTION FACTOR 3.** |
| 2.968 | AT2G39210 | **Major facilitator superfamily protein**. |
| 2.786 | AT4G26090 | **RESISTANT TO P. SYRINGAE 2, RPS2.** |
| 2.786 | AT1G07630 | **PLL5, POL-LIKE 5.** |
| 2.760 | AT4G18430 | **ATRABA1E, RAB GTPASE HOMOLOG A1E, RABA1E.** |
| 2.684 | AT1G30900 | **BINDING PROTEIN OF 80 KDA 3;3, BP80-3;3, VACUOLAR SORTING RECEPTOR 3;3, VACUOLAR SORTING RECEPTOR 6, VSR3;3, VSR6**. |
| 2.657 | AT2G44490 | **BETA GLUCOSIDASE 26, BGLU26, PEN2, PENETRATION 2.** |
| 2.615 | AT4G36550 | **ARM repeat superfamily protein.** |
| 2.576 | AT1G05010 | **ACO4, EAT1, EFE, ETHYLENE FORMING ENZYME, ETHYLENE-FORMING ENZYME**. |
| 2.576 | AT2G47800 | **ABCC4, ATMRP4, ATP-BINDING CASSETTE C4, EST3, MRP4, MULTIDRUG RESISTANCE-ASSOCIATED PROTEIN 4.** |
| 2.572 | AT1G59870 | **ABCG36, ARABIDOPSIS PLEIOTROPIC DRUG RESISTANCE 8, ARABIDOPSIS THALIANA ATP-BINDING CASSETTE G36, ATABCG36, ATP-BINDING CASSETTE G36, ATPDR8, PDR8, PEN3, PENETRATION 3, PLEIOTROPIC DRUG RESISTANCE 8.** |
| 2.559 | AT5G65210 | **TGA1, TGACG SEQUENCE-SPECIFIC BINDING PROTEIN 1.** |
| 2.534 | AT3G05660 | **ATRLP33, RECEPTOR LIKE PROTEIN 33, RLP33**. |
| 2.512 | AT4G37560 | **Acetamidase/Formamidase family protein**. |
| 2.434 | AT4G37150 | **ARABIDOPSIS THALIANA METHYL ESTERASE 9, ATMES9, MES9, METHYL ESTERASE 9.** |
| 2.417 | AT1G10210 | **ATMPK1, MITOGEN-ACTIVATED PROTEIN KINASE 1, MPK1.** |
| 2.407 | AT4G23810 | **ATWRKY53, WRKY53**. |
| 2.375 | AT4G16890 | **BAL, BALL, SNC1, SUPPRESSOR OF NPR1-1, CONSTITUTIVE 1.** |
| 2.366 | AT5G67340 | **ARM repeat superfamily protein.** |
| 2.334 | AT5G39680 | **EMB2744, EMBRYO DEFECTIVE 2744.** |
| 2.327 | AT5G43910 | **pfkB-like carbohydrate kinase family protein.** |
| 2.302 | AT2G04430 | **ATNUDT5, NUDIX HYDROLASE HOMOLOG 5, NUDT5**. |

Biotic stress-related genes (Control vs Ln DOWN)

| | | |
|---|---|---|
| 2.255 | AT5G60410 | **ATSIZ1, SIZ1.** |
| 2.253 | AT5G01540 | **L-TYPE LECTIN RECEPTOR KINASE-VI.2, LECRK-VI.2, LECRKA4.1, LECTIN RECEPTOR KINASE A4.1.** |
| 2.244 | AT1G34750 | **Protein phosphatase 2C family protein**. |
| 2.175 | AT1G64610 | **Transducin/WD40 repeat-like superfamily protein**. |
| 2.169 | AT1G08050 | **Zinc finger (C3HC4-type RING finger) family protein**. |
| 2.155 | AT1G11260 | **ATSTP1, STP1, SUGAR TRANSPORTER 1.** |
| 2.154 | AT4G02410 | **ATLPK1, LECTIN-LIKE PROTEIN KINASE 1, LPK1**. |
| 2.110 | AT1G34420 | **Leucine-rich repeat transmembrane protein kinase family protein**. |
| 2.061 | AT4G26080 | **ABA INSENSITIVE 1, ABI1, ATABI1**. |
| 2.056 | AT4G38550 | **Arabidopsis phospholipase-like protein (PEARLI 4) family**. |
| 2.042 | AT5G41410 | **BEL1, BELL 1**. |
| 2.020 | AT1G75460 | **ATP-dependent protease La (LON) domain protein**. |
| 2.012 | AT1G10340 | **Ankyrin repeat family protein**. |
