# Supplementary material for: Transcriptomic profiling of linolenic acid-responsive genes in ROS signaling from RNA-seq data in Arabidopsis
Source: Front Plant Sci. 2015 Mar 17;6:122. doi: 10.3389/fpls.2015.00122 (PMC4362301; doi:10.3389/fpls.2015.00122)
Supplement: Supplemental Table 1 — Fatty acid composition of Arabidopsis thaliana cell suspension cultures (ACSC). [file DataSheet1.ZIP › Table 5.PDF]

Abiotic stress-related genes (Control vs Ln UP)

FC	ID	Abiotic stress
6,928	AT5G62100	ATBAG2, BAG2, BCL-2-ASSOCIATED ATHANOGENE 2.
4,975	AT2G46240	ARABIDOPSIS THALIANA BCL-2-ASSOCIATED ATHANOGENE 6, ATBAG6, BAG6, BCL-2-ASSOCIATED ATHANOGENE 6.
2,975	AT1G61340	ATFBS1, F-BOX STRESS INDUCED 1, FBS1.
		**Response to heat**
24,725	AT2G47180	ATGOLS1, GALACTINOL SYNTHASE 1, GOLS1.
14,799	AT1G52560	HSP20-like chaperones superfamily protein.
14,151	AT4G12400	HOP3.
12,125	AT5G05410	DEHYDRATION-RESPONSIVE ELEMENT BINDING PROTEIN 2, DRE-BINDING PROTEIN 2A, DREB2, DREB2A.
11,405	AT2G26150	ATHSFA2, HEAT SHOCK TRANSCRIPTION FACTOR A2, HSFA2.
9,597	AT4G25200	ATHSP23.6-MITO, HSP23.6-MITO, MITOCHONDRION-LOCALIZED SMALL HEAT SHOCK PROTEIN 23.6.
7,230	AT5G59820	ATZAT12, RESPONSIVE TO HIGH LIGHT 41, RHL41, ZAT12.
6,299	AT5G57560	TCH4, TOUCH 4, XTH22, XYLOGLUCAN ENDOTRANSGLUCOSYLASE/HYDROLASE 22.
5,347	AT5G37770	CALMODULIN-LIKE 24, CML24, TCH2, TOUCH 2.
4,975	AT2G46240	ARABIDOPSIS THALIANA BCL-2-ASSOCIATED ATHANOGENE 6, ATBAG6, BAG6, BCL-2-ASSOCIATED ATHANOGENE 6.
4,870	AT2G20560	DNAJ heat shock family protein.
4,189	AT5G56030	ATHSP90.2, EARLY-RESPONSIVE TO DEHYDRATION 8, ERD8, HEAT SHOCK PROTEIN 90.2, HEAT SHOCK PROTEIN 81-2, HEAT SHOCK PROTEIN 81.2, HEAT SHOCK PROTEIN 90.2, HSP81-2, HSP81.2, HSP90.2.
4,106	AT2G32120	HEAT-SHOCK PROTEIN 70T-2, HSP70T-2.
4,035	AT1G74310	ATHSP101, HEAT SHOCK PROTEIN 101, HOT1, HSP101.
3,784	AT1G21910	DEHYDRATION RESPONSE ELEMENT-BINDING PROTEIN 26, DREB26.
3,260	AT1G14200	RING/U-box superfamily protein.
3,129	AT3G09350	Fes1A.
3,012	AT5G37670	HSP20-like chaperones superfamily protein.
2,873	AT3G16050	A37, ARABIDOPSIS THALIANA PYRIDOXINE BIOSYNTHESIS 1.2, ATPDX1.2, PDX1.2, PYRIDOXINE BIOSYNTHESIS 1.2.
2,817	AT2G38750	ANNAT4, ANNEXIN 4.
2,778	AT5G09240	ssDNA-binding transcriptional regulator.
2,674	AT1G17870	ATEGY3, EGY3, ETHYLENE-DEPENDENT GRAVITROPISM-DEFICIENT AND YELLOW-GREEN-LIKE 3.
2,233	AT4G21320	HEAT-STRESS-ASSOCIATED 32, HSA32.
2,089	AT1G16030	HEAT SHOCK PROTEIN 70B, HSP70B.
2,080	AT5G67180	TARGET OF EARLY ACTIVATION TAGGED (EAT) 3, TOE3.
		**Response to high light intensity**
24,725	AT2G47180	ATGOLS1, GALACTINOL SYNTHASE 1, GOLS1.

14,799	AT1G52560	**HSP20-like chaperones superfamily protein.**
14,151	AT4G12400	**HOP3.**
12,125	AT5G05410	**DEHYDRATION-RESPONSIVE ELEMENT BINDING PROTEIN 2, DRE-BINDING PROTEIN 2A, DREB2, DREB2A.**
11,405	AT2G26150	**ATHSFA2, HEAT SHOCK TRANSCRIPTION FACTOR A2, HSFA2.**
11,382	AT1G17420	**ARABIDOPSIS THALIANA LIPOXYGENASE 3, ATLOX3, LIPOXYGENASE 3, LOX3.**
9,597	AT4G25200	**ATHSP23.6-MITO, HSP23.6-MITO, MITOCHONDRION-LOCALIZED SMALL HEAT SHOCK PROTEIN 23.6.**
5,775	AT1G27730	**SALT TOLERANCE ZINC FINGER, STZ, ZAT10.**
4,975	AT2G46240	**ARABIDOPSIS THALIANA BCL-2-ASSOCIATED ATHANOGENE 6, ATBAG6, BAG6, BCL-2-ASSOCIATED ATHANOGENE 6.**
4,870	AT2G20560	**DNAJ heat shock family protein.**
4,106	AT2G32120	**HEAT-SHOCK PROTEIN 70T-2, HSP70T-2.**
4,035	AT1G74310	**ATHSP101, HEAT SHOCK PROTEIN 101, HOT1, HSP101.**
3,260	AT1G14200	**RING/U-box superfamily protein.**
3,129	AT3G09350	**Fes1A.**
3,012	AT5G37670	**HSP20-like chaperones superfamily protein.**
2,873	AT3G16050	**A37, ARABIDOPSIS THALIANA PYRIDOXINE BIOSYNTHESIS 1.2, ATPDX1.2, PDX1.2, PYRIDOXINE BIOSYNTHESIS 1.2.**
2,674	AT1G17870	**ATEGY3, EGY3, ETHYLENE-DEPENDENT GRAVITROPISM-DEFICIENT AND YELLOW-GREEN-LIKE 3.**
2,233	AT4G21320	**HEAT-STRESS-ASSOCIATED 32, HSA32.**
2,089	AT1G16030	**HEAT SHOCK PROTEIN 70B, HSP70B.**
		**Response to cold**
24,725	AT2G47180	**ATGOLS1, GALACTINOL SYNTHASE 1, GOLS1.**
11,762	AT1G28370	**ATERF11, ERF DOMAIN PROTEIN 11, ERF11.**
8,662	AT5G47230	**ATERF-5, ATERF5, ERF5, ETHYLENE RESPONSIVE ELEMENT BINDING FACTOR 5, ETHYLENE RESPONSIVE ELEMENT BINDING FACTOR- 5.**
7,902	AT1G79410	**ATOCT5, OCT5, ORGANIC CATION/CARNITINE TRANSPORTER5.**
7,230	AT5G59820	**ATZAT12, RESPONSIVE TO HIGH LIGHT 41, RHL41, ZAT12.**
6,299	AT5G57560	**TCH4, TOUCH 4, XTH22, XYLOGLUCAN ENDOTRANSGLUCOSYLASE/HYDROLASE 22.**
5,775	AT1G27730	**SALT TOLERANCE ZINC FINGER, STZ, ZAT10.**
5,347	AT5G37770	**CALMODULIN-LIKE 24, CML24, TCH2, TOUCH 2.**
4,755	AT3G14440	**ATNCED3, NCED3, NINE-CIS-EPOXYCAROTENOID DIOXYGENASE 3, SALT TOLERANT 1, SIS7, STO1, SUGAR INSENSITIVE 7.**
4,624	AT2G05940	**RIPK, RPM1-INDUCED PROTEIN KINASE.**
3,925	AT3G52400	**ATSYP122, SYNTAXIN OF PLANTS 122, SYP122.**
3,764	AT1G09350	**ATGOLS3, GALACTINOL SYNTHASE 3, GOLS3.**
3,501	AT3G50260	**ATERF011, CEJ1, COOPERATIVELY REGULATED BY ETHYLENE AND JASMONATE 1, DEAR1, DREB AND EAR MOTIF PROTEIN 1.**
3,297	AT1G52890	**ANAC019, NAC DOMAIN CONTAINING PROTEIN 19, NAC019.**
3,264	AT3G15500	**ANAC055, ATNAC3, NAC DOMAIN CONTAINING PROTEIN 3, NAC DOMAIN CONTAINING PROTEIN 55, NAC055, NAC3.**
2,975	AT2G46590	**DAG2, DOF AFFECTING GERMINATION 2.**
2,861	AT1G55920	**ATSERAT2;1, SAT1, SAT5, SERAT2;1, SERINE ACETYLTRANSFERASE 1,**

		**SERINE ACETYLTRANSFERASE 2;1, SERINE ACETYLTRANSFERASE 5.**
2,817	AT2G38750	**ANNAT4, ANNEXIN 4.**
2,747	AT4G34150	**Calcium-dependent lipid-binding domain-containing protein.**
2,539	AT4G27410	**ANAC072, ARABIDOPSIS NAC DOMAIN CONTAINING PROTEIN 72, RD26, RESPONSIVE TO DESICCATION 26.**
2,481	AT2G47730	**ARABIDOPSIS THALIANA GLUTATHIONE S-TRANSFERASE PHI 8, ATGSTF5, ATGSTF8, GLUTATHIONE S-TRANSFERASE (CLASS PHI) 5, GLUTATHIONE S-TRANSFERASE PHI 8, GST6, GSTF8.**
2,372	AT3G05890	**RARE-COLD-INDUCIBLE 2B, RCI2B.**
2,268	AT3G17390	**MAT4, METHIONINE ADENOSYLTRANSFERASE 4, METHIONINE OVER-ACCUMULATOR 3, MTO3, S-ADENOSYLMETHIONINE SYNTHETASE 3, SAMS3.**
2,191	AT5G42050	**DCD (Development and Cell Death) domain protein.**
2,034	AT1G80820	**ATCCR2, CCR2, CINNAMOYL COA REDUCTASE.**
		**Response to water deprivation**
36,838	AT5G13220	**JAS1, JASMONATE-ASSOCIATED 1, JASMONATE-ZIM-DOMAIN PROTEIN 10, JAZ10, TIFY DOMAIN PROTEIN 9, TIFY9.**
24,812	AT1G17380	**JASMONATE-ZIM-DOMAIN PROTEIN 5, JAZ5, TIFY11A.**
24,725	AT2G47180	**ATGOLS1, GALACTINOL SYNTHASE 1, GOLS1.**
22,102	AT4G17500	**ATERF-1, ERF-1, ETHYLENE RESPONSIVE ELEMENT BINDING FACTOR 1.**
16,292	AT5G54490	**PBP1, PINOID-BINDING PROTEIN 1.**
14,502	AT1G06620	**Encodes a protein whose sequence is similar to a 2-oxoglutarate-dependent dioxygenase.**
12,125	AT5G05410	**DEHYDRATION-RESPONSIVE ELEMENT BINDING PROTEIN 2, DRE-BINDING PROTEIN 2A, DREB2, DREB2A.**
11,762	AT1G28370	**ATERF11, ERF DOMAIN PROTEIN 11, ERF11.**
9,223	AT3G23250	**ATMYB15, ATY19, MYB DOMAIN PROTEIN 15, MYB15.**
8,813	AT1G74950	**JASMONATE-ZIM-DOMAIN PROTEIN 2, JAZ2, TIFY10B.**
7,902	AT1G79410	**ATOCT5, OCT5, ORGANIC CATION/CARNITINE TRANSPORTER5.**
7,230	AT5G59820	**ATZAT12, RESPONSIVE TO HIGH LIGHT 41, RHL41, ZAT12.**
5,846	AT2G27690	**"CYTOCHROME P450, FAMILY 94, SUBFAMILY C, POLYPEPTIDE 1", CYP94C1.**
5,775	AT1G27730	**SALT TOLERANCE ZINC FINGER, STZ, ZAT10.** R
5,603	AT2G06050	**ATOPR3, DDE1, DELAYED DEHISCENCE 1, OPR3, OXOPHYTODIENOATE-REDUCTASE 3.**
5,404	AT3G06490	**ATMYB108, BOS1, BOTRYTIS-SUSCEPTIBLE1, MYB DOMAIN PROTEIN 108, MYB108.**
5,152	AT1G57560	**ATMYB50, MYB DOMAIN PROTEIN 50, MYB50.**
5,119	AT3G28210	**PMZ, SAP12, STRESS-ASSOCIATED PROTEIN 12.**
5,084	AT1G01720	**ANAC002, ARABIDOPSIS NAC DOMAIN CONTAINING PROTEIN 2, ATAF1.**
5,076	AT2G35930	**ATPUB23, PLANT U-BOX 23, PUB23.**
4,755	AT3G14440	**ATNCED3, NCED3, NINE-CIS-EPOXYCAROTENOID DIOXYGENASE 3, SALT TOLERANT 1, SIS7, STO1, SUGAR INSENSITIVE 7.**
4,624	AT2G05940	**RIPK, RPM1-INDUCED PROTEIN KINASE.**
4,189	AT5G56030	**ATHSP90.2, EARLY-RESPONSIVE TO DEHYDRATION 8, ERD8, HEAT SHOCK PROTEIN 90.2, HEAT SHOCK PROTEIN 81-2, HEAT SHOCK**

		**PROTEIN 81.2, HEAT SHOCK PROTEIN 90.2, HSP81-2, HSP81.2, HSP90.2.**
4,074	AT3G19580	**AZF2, ZF2, ZINC-FINGER PROTEIN 2**.
3,925	AT3G52400	**ATSYP122, SYNTAXIN OF PLANTS 122, SYP122.**
3,846	AT3G09940	**ARABIDOPSIS THALIANA MONODEHYDROASCORBATE REDUCTASE 3, ATMDAR3, MDAR2, MDAR3, MDHAR, MONODEHYDROASCORBATE REDUCTASE, MONODEHYDROASCORBATE REDUCTASE 2, MONODEHYDROASCORBATE REDUCTASE 3.**
3,712	AT1G63840	**RING/U-box superfamily protein**.
3,628	AT1G15520	**ABCG40, ARABIDOPSIS THALIANA ATP-BINDING CASSETTE G40, ATABCG40, ATP-BINDING CASSETTE G40, ATPDR12, PDR12, PLEIOTROPIC DRUG RESISTANCE 12.**
3,541	AT1G74100	**ARABIDOPSIS SULFOTRANSFERASE 5A, ATSOT16, ATST5A, CORI-7, CORONATINE INDUCED-7, SOT16, SULFOTRANSFERASE 16.**
3,501	AT3G50260	**ATERF011, CEJ1, COOPERATIVELY REGULATED BY ETHYLENE AND JASMONATE 1, DEAR1, DREB AND EAR MOTIF PROTEIN 1.**
3,459	AT5G67300	**ARABIDOPSIS THALIANA MYB DOMAIN PROTEIN 44, ATMYB44, ATMYBR1, MYB DOMAIN PROTEIN R1, MYB44, MYBR1**.
3,297	AT1G52890	**ANAC019, NAC DOMAIN CONTAINING PROTEIN 19, NAC019**.
3,264	AT3G15500	**ANAC055, ATNAC3, NAC DOMAIN CONTAINING PROTEIN 3, NAC DOMAIN CONTAINING PROTEIN 55, NAC055, NAC3**.
2,817	AT2G38750	**ANNAT4, ANNEXIN 4.**
2,539	AT4G27410	**ANAC072, ARABIDOPSIS NAC DOMAIN CONTAINING PROTEIN 72, RD26, RESPONSIVE TO DESICCATION 26.**
2,478	AT2G46510	**ABA-INDUCIBLE BHLH-TYPE TRANSCRIPTION FACTOR, AIB, ATAIB.**
2,213	AT5G59550	**ARABIDOPSIS THALIANA RING AND DOMAIN OF UNKNOWN FUNCTION 1117 2, ATRDUF2, RDUF2, RING AND DOMAIN OF UNKNOWN FUNCTION 1117 2**.
		**Response to wounding**
36,838	AT5G13220	**JAS1, JASMONATE-ASSOCIATED 1, JASMONATE-ZIM-DOMAIN PROTEIN 10, JAZ10, TIFY DOMAIN PROTEIN 9, TIFY9**.
36,680	AT5G05600	**2-oxoglutarate (2OG) and Fe(II)-dependent oxygenase superfamily protein.**
32,561	AT1G74930	**ORA47.**
27,503	AT2G26530	**AR781.**
26,669	AT1G80840	**ATWRKY40, WRKY DNA-BINDING PROTEIN 40, WRKY40.**
25,665	AT1G72450	**JASMONATE-ZIM-DOMAIN PROTEIN 6, JAZ6, TIFY DOMAIN PROTEIN 11B, TIFY11B.**
24,812	AT1G17380	**JASMONATE-ZIM-DOMAIN PROTEIN 5, JAZ5, TIFY11A.**
24,266	AT1G44350	**IAA-LEUCINE RESISTANT (ILR)-LIKE GENE 6, ILL6.**
22,834	AT1G72520	**ARABIDOPSIS THALIANA LIPOXYGENASE 4, ATLOX4, LIPOXYGENASE 4, LOX4.**
16,058	AT5G08790	**ANAC081, ARABIDOPSIS NAC DOMAIN CONTAINING PROTEIN 81, ATAF2**.
15,027	AT3G44260	**ATCAF1A, CAF1A, CCR4- ASSOCIATED FACTOR 1A.**
14,502	AT1G06620	**Encodes a protein whose sequence is similar to a 2-oxoglutarate-dependent dioxygenase.**
13,449	AT3G25780	**ALLENE OXIDE CYCLASE 3, AOC3**.

12,956	AT5G13220	**JAS1, JASMONATE-ASSOCIATED 1, JASMONATE-ZIM-DOMAIN PROTEIN 10, JAZ10, TIFY DOMAIN PROTEIN 9, TIFY9**.
12,267	AT1G32640	**ATMYC2, JAI1, JASMONATE INSENSITIVE 1, JIN1, MYC2, RD22BP1, ZBF1.**
11,382	AT1G17420	**ARABIDOPSIS THALIANA LIPOXYGENASE 3, ATLOX3, LIPOXYGENASE 3, LOX3.**
9,711	AT5G42650	**ALLENE OXIDE SYNTHASE, AOS, CYP74A, CYTOCHROME P450 74A, DDE2, DELAYED DEHISCENCE 2.**
9,619	AT1G70700	**JASMONATE-ZIM-DOMAIN PROTEIN 9, JAZ9, TIFY7.**
9,223	AT3G23250	**ATMYB15, ATY19, MYB DOMAIN PROTEIN 15, MYB15**.
8,813	AT1G74950	**JASMONATE-ZIM-DOMAIN PROTEIN 2, JAZ2, TIFY10B**.
8,056	AT5G20230	**ATBCB, BCB, BLUE COPPER BINDING PROTEIN, BLUE-COPPER-BINDING PROTEIN, SAG14, SENESCENCE ASSOCIATED GENE 14**.
7,230	AT5G59820	**ATZAT12, RESPONSIVE TO HIGH LIGHT 41, RHL41, ZAT12**.
7,075	AT5G22630	**ADT5, AROGENATE DEHYDRATASE 5.**
6,480	AT1G05800	**DGL, DONGLE.**
6,299	AT5G57560	**TCH4, TOUCH 4, XTH22, XYLOGLUCAN ENDOTRANSGLUCOSYLASE/HYDROLASE 22**.
5,890	AT1G73500	**ATMKK9, MAP KINASE KINASE 9, MKK9.**
5,846	AT2G27690	**"CYTOCHROME P450, FAMILY 94, SUBFAMILY C, POLYPEPTIDE 1", CYP94C1**.
5,775	AT1G27730	**SALT TOLERANCE ZINC FINGER, STZ, ZAT10.**
5,603	AT2G06050	**ATOPR3, DDE1, DELAYED DEHISCENCE 1, OPR3, OXOPHYTODIENOATE-REDUCTASE 3**.
5,404	AT3G06490	**ATMYB108, BOS1, BOTRYTIS-SUSCEPTIBLE1, MYB DOMAIN PROTEIN 108, MYB108**.
5,347	AT5G37770	**CALMODULIN-LIKE 24, CML24, TCH2, TOUCH 2**
5,119	AT3G28210	**PMZ, SAP12, STRESS-ASSOCIATED PROTEIN 12**.
5,084	AT1G01720	**ANAC002, ARABIDOPSIS NAC DOMAIN CONTAINING PROTEIN 2, ATAF1**.
4,724	AT1G20510	**OPC-8:0 COA LIGASE1, OPCL1**.
4,680	AT5G63450	**"CYTOCHROME P450, FAMILY 94, SUBFAMILY B, POLYPEPTIDE 1", CYP94B1.**
4,624	AT2G05940	**RIPK, RPM1-INDUCED PROTEIN KINASE**.
4,417	AT1G76650	**CALMODULIN-LIKE 38, CML38**.
4,396	AT3G25250	**AGC2, AGC2-1, ATOXI1, OXI1, OXIDATIVE SIGNAL-INDUCIBLE1**.
4,074	AT3G19580	**AZF2, ZF2, ZINC-FINGER PROTEIN 2**.
3,846	AT3G09940	**ARABIDOPSIS THALIANA MONODEHYDROASCORBATE REDUCTASE 3, ATMDAR3, MDAR2, MDAR3, MDHAR, MONODEHYDROASCORBATE REDUCTASE, MONODEHYDROASCORBATE REDUCTASE 2, MONODEHYDROASCORBATE REDUCTASE 3.**
3,797	AT4G24570	**DIC2, DICARBOXYLATE CARRIER 2.**
3,712	AT1G63840	**RING/U-box superfamily protein**.
3,501	AT3G50260	**ATERF011, CEJ1, COOPERATIVELY REGULATED BY ETHYLENE AND JASMONATE 1, DEAR1, DREB AND EAR MOTIF PROTEIN 1.**
3,420	AT3G51450	**Calcium-dependent phosphotriesterase superfamily protein**.
3,419	AT3G04640	**Glycine-rich protein**.
3,297	AT1G52890	**ANAC019, NAC DOMAIN CONTAINING PROTEIN 19, NAC019**.

3,264	AT3G15500	**ANAC055, ATNAC3, NAC DOMAIN CONTAINING PROTEIN 3, NAC DOMAIN CONTAINING PROTEIN 55, NAC055, NAC3**.
3,091	AT5G53750	**CBS domain-containing protein**.
2,975	AT1G61340	**ATFBS1, F-BOX STRESS INDUCED 1, FBS1**.
2,880	AT2G47890	**Zinc finger protein CONSTANS-LIKE 13**.
2,747	AT4G34150	**Calcium-dependent lipid-binding domain-containing protein.**
2,587	AT1G76690	**12-OXOPHYTODIENOATE REDUCTASE 2, ARABIDOPSIS 12-OXOPHYTODIENOATE REDUCTASE 2, ATOPR2, OPR2**.
2,539	AT4G27410	**ANAC072, ARABIDOPSIS NAC DOMAIN CONTAINING PROTEIN 72, RD26, RESPONSIVE TO DESICCATION 26.**
2,478	AT2G46510	**ABA-INDUCIBLE BHLH-TYPE TRANSCRIPTION FACTOR, AIB, ATAIB.**
2,415	AT3G55980	**ATSZF1, SALT-INDUCIBLE ZINC FINGER 1, SZF1**.
2,386	AT2G30020	Encodes **AP2C1**.
2,316	AT4G38620	**ATMYB4, MYB DOMAIN PROTEIN 4, MYB4**.
2,303	AT1G09070	**(AT)SRC2, SOYBEAN GENE REGULATED BY COLD-2, SRC2**.
2,213	AT4G11280	**1-AMINOCYCLOPROPANE-1-CARBOXYLIC ACID (ACC) SYNTHASE 6, ACS6, ATACS6**.
		**Response to osmotic stress**
10,729	AT3G04730	**IAA16, INDOLEACETIC ACID-INDUCED PROTEIN 16.**
4,755	AT3G14440	**ATNCED3, NCED3, NINE-CIS-EPOXYCAROTENOID DIOXYGENASE 3, SALT TOLERANT 1, SIS7, STO1, SUGAR INSENSITIVE 7**.
2,975	AT1G61340	**ATFBS1, F-BOX STRESS INDUCED 1, FBS1**.
2,817	AT2G38750	**ANNAT4, ANNEXIN 4.**
		**Response to salt stress**
24,725	AT2G47180	**ATGOLS1, GALACTINOL SYNTHASE 1, GOLS1.**
13,449	AT3G25780	**ALLENE OXIDE CYCLASE 3, AOC3**.
9,223	AT3G23250	**ATMYB15, ATY19, MYB DOMAIN PROTEIN 15, MYB15**.
7,902	AT1G79410	**ATOCT5, OCT5, ORGANIC CATION/CARNITINE TRANSPORTER5**.
5,934	AT3G06370	**ATNHX4, NHX4, SODIUM HYDROGEN EXCHANGER 4. Member of Sodium proton exchanger family.**
5,890	AT1G73500	**ATMKK9, MAP KINASE KINASE 9, MKK9**.
5,775	AT1G27730	**SALT TOLERANCE ZINC FINGER, STZ, ZAT10**.
5,404	AT3G06490	**ATMYB108, BOS1, BOTRYTIS-SUSCEPTIBLE1, MYB DOMAIN PROTEIN 108, MYB108**.
4,189	AT5G56030	**ATHSP90.2, EARLY-RESPONSIVE TO DEHYDRATION 8, ERD8, HEAT SHOCK PROTEIN 90.2, HEAT SHOCK PROTEIN 81-2, HEAT SHOCK PROTEIN 81.2, HEAT SHOCK PROTEIN 90.2, HSP81-2, HSP81.2, HSP90.2.**
3,846	AT3G09940	**ARABIDOPSIS THALIANA MONODEHYDROASCORBATE REDUCTASE 3, ATMDAR3, MDAR2, MDAR3, MDHAR, MONODEHYDROASCORBATE REDUCTASE, MONODEHYDROASCORBATE REDUCTASE 2, MONODEHYDROASCORBATE REDUCTASE 3.**
3,536	AT1G61890	**MATE efflux family protein**.
3,526	AT2G16720	**ARABIDOPSIS THALIANA MYB DOMAIN PROTEIN 7, ATMYB7, ATY49, MYB DOMAIN PROTEIN 7, MYB7**.
3,492	AT5G62520	**SIMILAR TO RCD ONE 5, SRO5.**
3,459	AT5G67300	**ARABIDOPSIS THALIANA MYB DOMAIN PROTEIN 44, ATMYB44,**

		**ATMYBR1, MYB DOMAIN PROTEIN R1, MYB44, MYBR1.**
3,129	AT3G09350	**Fes1A.**
2,975	AT1G61340	**ATFBS1, F-BOX STRESS INDUCED 1, FBS1.**
2,848	AT4G34990	**ATMYB32, MYB DOMAIN PROTEIN 32, MYB32.**
2,817	AT2G38750	**ANNAT4, ANNEXIN 4.**
2,662	AT1G18330	**EARLY-PHYTOCHROME-RESPONSIVE1, EPR1, REVEILLE 7, RVE7.**
2,481	AT2G47730	**ARABIDOPSIS THALIANA GLUTATHIONE S-TRANSFERASE PHI 8, ATGSTF5, ATGSTF8, GLUTATHIONE S-TRANSFERASE (CLASS PHI) 5, GLUTATHIONE S-TRANSFERASE PHI 8, GST6, GSTF8.**
2,449	AT5G17310	**ATUGP2, UDP-GLUCOSE PYROPHOSPHORYLASE 2, UGP2.**
2,243	AT2G38170	**ATCAX1, CATION EXCHANGER 1, CAX1, RARE COLD INDUCIBLE 4, RCI4.**
2,212	AT5G37260	**CIR1, CIRCADIAN 1, REVEILLE 2, RVE2.**
		**Response to mechanical stimulus**
27,503	AT2G26530	**AR781.**
26,669	AT1G80840	**ATWRKY40, WRKY DNA-BINDING PROTEIN 40, WRKY40.**
15,027	AT3G44260	**ATCAF1A, CAF1A, CCR4- ASSOCIATED FACTOR 1A.**
6,299	AT5G57560	**TCH4, TOUCH 4, XTH22, XYLOGLUCAN ENDOTRANSGLUCOSYLASE/HYDROLASE 22.**
5,775	AT1G27730	**SALT TOLERANCE ZINC FINGER, STZ, ZAT10.**
5,347	AT5G37770	**CALMODULIN-LIKE 24, CML24, TCH2, TOUCH 2.**
4,189	AT5G56030	**ATHSP90.2, EARLY-RESPONSIVE TO DEHYDRATION 8, ERD8, HEAT SHOCK PROTEIN 90.2, HEAT SHOCK PROTEIN 81-2, HEAT SHOCK PROTEIN 81.2, HEAT SHOCK PROTEIN 90.2, HSP81-2, HSP81.2, HSP90.2.**
3,908	AT3G62720	**ATXT1, XT1, XXT1, XYG XYLOSYLTRANSFERASE 1, XYLOSYLTRANSFERASE 1.**
3,797	AT4G24570	**DIC2, DICARBOXYLATE CARRIER 2.**
3,469	AT3G61640	**AGP20, ARABINOGALACTAN PROTEIN 20, ATAGP20.**
3,419	AT3G04640	**Glycine-rich protein.**
2,747	AT4G34150	**Calcium-dependent lipid-binding domain-containing protein.**
2,415	AT3G55980	**ATSZF1, SALT-INDUCIBLE ZINC FINGER 1, SZF1.**
2,303	AT1G09070	**(AT)SRC2, SOYBEAN GENE REGULATED BY COLD-2, SRC2.**
2,213	AT4G11280	**1-AMINOCYCLOPROPANE-1-CARBOXYLIC ACID (ACC) SYNTHASE 6, ACS6, ATACS6.**
		**Response to UV-B**
12,125	AT5G05410	**DEHYDRATION-RESPONSIVE ELEMENT BINDING PROTEIN 2, DRE-BINDING PROTEIN 2A, DREB2, DREB2A.**
7,230	AT5G59820	**ATZAT12, RESPONSIVE TO HIGH LIGHT 41, RHL41, ZAT12.**
2,728	AT3G61220	**SDR1, SHORT-CHAIN DEHYDROGENASE/REDUCTASE 1.**
2,316	AT4G38620	**ATMYB4, MYB DOMAIN PROTEIN 4, MYB4.**
2,008	AT5G15950	**S-adenosylmethionine decarboxylase-like protein.**

Abiotic stress-related genes (Control vs Ln DOWN)

FC	ID	Abiotic stress
2.925	AT1G56570	**PENTATRICOPEPTIDE REPEAT PROTEIN FOR GERMINATION ON NACL, PGN**.
2.218	AT2G40190	**LEAF WILTING 3, LEW3.**
2.098	AT1G35515	**HIGH RESPONSE TO OSMOTIC STRESS 10, HOS10, MYB8.**
2.024	AT2G33380	**ARABIDOPSIS THALIANA CALEOSIN 3, ATCLO3, CALEOSIN 3, CLO-3, CLO3, RD20, RESPONSIVE TO DESSICATION 20.**
		**Response to heat**
4.174	AT1G07390	**ATRLP1, RECEPTOR LIKE PROTEIN 1, RLP1**.
3.486	AT5G07340	**Calreticulin family protein.**
2.983	AT5G12380	**ANNAT8, ANNEXIN 8.**
2.808	AT4G18450	Encodes a member of the **ERF (ethylene response factor) subfamily B-3 of ERF/AP2 transcription factor family**.
2.779	AT3G19930	**ATSTP4, STP4, SUGAR TRANSPORTER 4.**
2.691	AT1G76560	**CP12 DOMAIN-CONTAINING PROTEIN 3, CP12-3**.
2.681	AT4G24240	**ATWRKY7, WRKY DNA-BINDING PROTEIN 7, WRKY7.**
2.534	AT3G05660	**ATRLP33, RECEPTOR LIKE PROTEIN 33, RLP33**.
2.492	AT1G72660	**P-loop containing nucleoside triphosphate hydrolases superfamily protein.**
2.439	AT1G70520	**ALTERED SEED GERMINATION 6, ASG6, CRK2, CYSTEINE-RICH RLK (RECEPTOR-LIKE PROTEIN KINASE) 2.**
2.336	AT3G02885	**GASA5, GAST1 PROTEIN HOMOLOG 5.**
2.313	AT3G28030	**ULTRAVIOLET HYPERSENSITIVE 3, UV REPAIR DEFECTIVE 1, UVH3, UVR1.**
2.298	AT5G37630	**EMB2656, EMBRYO DEFECTIVE 2656**.
2.280	AT3G23610	**DSPTP1, DUAL SPECIFICITY PROTEIN PHOSPHATASE 1.**
2.207	AT5G45710	**AT-HSFA4C, HEAT SHOCK TRANSCRIPTION FACTOR A4C, HSFA4C, RHA1, ROOT HANDEDNESS 1.**
2.184	AT5G03280	**ATEIN2, CKR1, CYTOKININ RESISTANT 1, EIN2, ENHANCED RESPONSE TO ABA3, ERA3, ETHYLENE INSENSITIVE 2, ORE2, ORE3, ORESARA 2, ORESARA 3, PIR2**.
2.160	AT3G06010	**ATCHR12, CHR12, CHROMATIN REMODELING 12**.
2.138	AT4G26850	**VITAMIN C DEFECTIVE 2, VTC2**.
2.061	AT4G26080	**ABA INSENSITIVE 1, ABI1, ATABI1**.
2.022	AT3G12570	**FYD.**
		**Response to high light intensity**
4.174	AT1G07390	**ATRLP1, RECEPTOR LIKE PROTEIN 1, RLP1**.
3.486	AT5G07340	**Calreticulin family protein.**
2.808	AT4G18450	Encodes a member of the **ERF (ethylene response factor) subfamily B-3 of ERF/AP2 transcription factor family**.
2.797	AT3G08940	**LHCB4.2, LIGHT HARVESTING COMPLEX PHOTOSYSTEM II**.
2.779	AT3G19930	**ATSTP4, STP4, SUGAR TRANSPORTER 4.**
2.723	AT5G04140	**FD-GOGAT, FERREDOXIN-DEPENDENT GLUTAMATE SYNTHASE, FERREDOXIN-DEPENDENT GLUTAMATE SYNTHASE 1, GLS1, GLU1, GLUS, GLUTAMATE SYNTHASE 1.**
2.681	AT4G24240	**ATWRKY7, WRKY DNA-BINDING PROTEIN 7, WRKY7.**
2.534	AT3G05660	**ATRLP33, RECEPTOR LIKE PROTEIN 33, RLP33**.

2.492	AT1G72660	**P-loop containing nucleoside triphosphate hydrolases superfamily protein.**
2.439	AT1G70520	**ALTERED SEED GERMINATION 6, ASG6, CRK2, CYSTEINE-RICH RLK (RECEPTOR-LIKE PROTEIN KINASE) 2.**
2.207	AT5G18750	**DNAJ heat shock N-terminal domain-containing protein**.
2.176	AT3G27690	**LHCB2, LHCB2.3, LHCB2.4, LIGHT-HARVESTING CHLOROPHYLL B-BINDING 2, PHOTOSYSTEM II LIGHT HARVESTING COMPLEX GENE 2.3.**
2.094	AT3G46920	**Protein kinase superfamily protein with octicosapeptide/Phox/Bem1p domain**.
2.022	AT3G12570	**FYD.**
2.014	AT5G50380	**ATEXO70F1, EXO70F1, EXOCYST SUBUNIT EXO70 FAMILY PROTEIN F1**.
		**Response to cold**
7.650	AT2G40180	**ATHPP2C5, PHOSPHATASE 2C5, PP2C5**.
7.473	AT5G67450	**AZF1, ZF1, ZINC-FINGER PROTEIN 1.**
7.280	AT2G20990	**ARABIDOPSIS THALIANA SYNAPTOTAGMIN A, ATSYTA, NTMC2T1.1, NTMC2TYPE1.1, SYNAPTOTAGMIN 1, SYNAPTOTAGMIN A, SYT1, SYTA**.
6.989	AT5G14740	**BETA CA2, BETA CARBONIC ANHYDRASE 2, CA18, CA2, CARBONIC ANHYDRASE 18, CARBONIC ANHYDRASE 2.**
5.463	AT1G68130	**ATIDD14, IDD14, IDD14ALPHA, IDD14BETA, INDETERMINATE(ID)-DOMAIN 14.**
5.432	AT5G14740	**BETA CA2, BETA CARBONIC ANHYDRASE 2, CA18, CA2, CARBONIC ANHYDRASE 18, CARBONIC ANHYDRASE 2**.
4.160	AT3G01500	**ARABIDOPSIS THALIANA SALICYLIC ACID-BINDING PROTEIN 3, ATBCA1, ATSABP3, BETA CARBONIC ANHYDRASE 1, CA1, CARBONIC ANHYDRASE 1, SABP3, SALICYLIC ACID-BINDING PROTEIN 3**.
3.790	AT5G52310	**COLD REGULATED 78, COR78, LOW-TEMPERATURE-INDUCED 78, LTI140, LTI78, RD29A, RESPONSIVE TO DESSICATION 29A**.
3.724	AT3G05640	**Protein phosphatase 2C family protein**.
3.477	AT1G01120	**3-KETOACYL-COA SYNTHASE 1, KCS1.**
3.420	AT3G63010	**ATGID1B, GA INSENSITIVE DWARF1B, GID1B**.
3.162	AT3G59710	**NAD(P)-binding Rossmann-fold superfamily protein.**
2.983	AT5G12380	**ANNAT8, ANNEXIN 8.**
2.786	AT1G07630	**PLL5, POL-LIKE 5.**
2.740	AT5G63320	**NPX1, NUCLEAR PROTEIN X1**.
2.723	AT5G04140	**FD-GOGAT, FERREDOXIN-DEPENDENT GLUTAMATE SYNTHASE, FERREDOXIN-DEPENDENT GLUTAMATE SYNTHASE 1, GLS1, GLU1, GLUS, GLUTAMATE SYNTHASE 1.**
2.657	AT2G44490	**BETA GLUCOSIDASE 26, BGLU26, PEN2, PENETRATION 2.**
2.583	AT2G46680	**ARABIDOPSIS THALIANA HOMEOBOX 7, ATHB-7, ATHB7, HB-7, HOMEOBOX 7**.
2.561	AT3G48360	**ATBT2, BT2, BTB AND TAZ DOMAIN PROTEIN 2.**
2.493	AT5G52300	**LOW-TEMPERATURE-INDUCED 65, LTI65, RD29B, RESPONSIVE TO DESSICATION 29B.**
2.482	AT1G27320	**AHK3, HISTIDINE KINASE 3, HK3.**
2.458	AT1G20440	**ATCOR47, COLD-REGULATED 47, COR47, RD17.**
2.449	AT5G35750	**AHK2, HISTIDINE KINASE 2, HK2.**
2.417	AT1G10210	**ATMPK1, MITOGEN-ACTIVATED PROTEIN KINASE 1, MPK1.**
2.366	AT5G67340	**ARM repeat superfamily protein.**
2.359	AT2G43020	**ATPAO2, PAO2, POLYAMINE OXIDASE 2.**
2.334	AT4G26440	**ATWRKY34, MICROSPORE-SPECIFIC PROMOTER 3, MSP3, WRKY DNA-BINDING PROTEIN 34, WRKY34.**
2.280	AT3G23610	**DSPTP1, DUAL SPECIFICITY PROTEIN PHOSPHATASE 1.**

2.276	AT2G16500	**ADC1, ARGDC, ARGDC1, ARGININE DECARBOXYLASE 1, SPE1.**
2.266	AT2G36830	**GAMMA TONOPLAST INTRINSIC PROTEIN, GAMMA TONOPLAST INTRINSIC PROTEIN 1, GAMMA-TIP, GAMMA-TIP1, TIP1;1, TONOPLAST INTRINSIC PROTEIN 1;1.**
2.258	AT3G50970	**LOW TEMPERATURE-INDUCED 30, LTI30, XERO2.**
2.253	AT5G01540	**L-TYPE LECTIN RECEPTOR KINASE-VI.2, LECRK-VI.2, LECRKA4.1, LECTIN RECEPTOR KINASE A4.1.**
2.239	AT2G25930	**EARLY FLOWERING 3, ELF3, PYK20.**
2.201	AT4G35300	**TMT2, TONOPLAST MONOSACCHARIDE TRANSPORTER2.**
2.175	AT1G64610	**Transducin/WD40 repeat-like superfamily protein.**
2.169	AT1G08050	**Zinc finger (C3HC4-type RING finger) family protein.**
2.126	AT1G60930	**ATRECQ4B, RECQ HELICASE L4B, RECQ4B, RECQL4B.**
2.112	AT3G23700	**Nucleic acid-binding proteins superfamily.**
2.092	AT5G63770	**ATDGK2, DGK2, DIACYLGLYCEROL KINASE 2.**
2.081	AT1G56600	**ATGOLS2, GALACTINOL SYNTHASE 2, GOLS2.**
2.061	AT4G26080	**ABA INSENSITIVE 1, ABI1, ATABI1.**
2.036	AT3G05740	**RECQ HELICASE L1, RECQI1.**
2.024	AT2G33380	**ARABIDOPSIS THALIANA CALEOSIN 3, ATCLO3, CALEOSIN 3, CLO-3, CLO3, RD20, RESPONSIVE TO DESSICATION 20.**
2.000	AT3G09260	**BGLU23, LEB, LONG ER BODY, PSR3.1, PYK10.**
2.000	AT5G58780	**ATCPT5, ATHEPS, CIS -PRENYLTRANSFERASE 5, CPT5, HEPS, HEPTAPRENYL DIPHOSPHATE SYNTHASE.**
		**Response to water deprivation**
173.307	AT2G20880	**ATERF53, ERF DOMAIN 53, ERF53.**
7.690	AT2G36270	**ABA INSENSITIVE 5, ABI5, GIA1, GROWTH-INSENSITIVITY TO ABA 1.**
6.491	AT2G01830	**AHK4, ARABIDOPSIS HISTIDINE KINASE 4, ATCRE1, CRE1, CYTOKININ RESPONSE 1, WOL, WOL1, WOODEN LEG, WOODEN LEG 1.**
5.874	AT5G60410	**ATSIZ1, SIZ1.**
5.349	AT5G57660	**ATCOL5, B-BOX DOMAIN PROTEIN 6, BBX6, COL5, CONSTANS-LIKE 5.**
4.622	AT2G26650	**AKT1, ATAKT1, K+ TRANSPORTER 1, KT1.**
3.790	AT5G52310	**COLD REGULATED 78, COR78, LOW-TEMPERATURE-INDUCED 78, LTI140, LTI78, RD29A, RESPONSIVE TO DESSICATION 29A.**
3.724	AT3G05640	**Protein phosphatase 2C family protein.**
3.518	AT5G38710	**Methylenetetrahydrofolate reductase family protein.**
3.484	AT5G25370	**PHOSPHOLIPASE D ALPHA 3, PLDALPHA3.**
3.420	AT3G63010	**ATGID1B, GA INSENSITIVE DWARF1B, GID1B.**
3.269	AT5G60410	**ATSIZ1, SIZ1.**
3.256	AT2G33230	**YUC7, YUCCA 7.**
3.241	AT1G54710	**ATATG18H, ATG18H, HOMOLOG OF YEAST AUTOPHAGY 18 (ATG18) H.**
3.202	AT1G69600	**ARABIDOPSIS THALIANA HOMEOBOX PROTEIN 29, ATHB29, ZFHD1, ZHD11, ZINC FINGER HOMEODOMAIN 1, ZINC FINGER HOMEODOMAIN 11.**
3.118	AT4G08920	**ATCRY1, BLU1, BLUE LIGHT UNINHIBITED 1, CRY1, CRYPTOCHROME 1, ELONGATED HYPOCOTYL 4, HY4, OOP2, OUT OF PHASE 2.**
3.045	AT3G61430	**ARABIDOPSIS THALIANA PLASMA MEMBRANE INTRINSIC PROTEIN 1, ATPIP1, PIP1, PIP1;1, PIP1A, PLASMA MEMBRANE INTRINSIC PROTEIN 1, PLASMA MEMBRANE INTRINSIC PROTEIN 1;1, PLASMA MEMBRANE INTRINSIC PROTEIN 1A.**
2.983	AT5G12380	**ANNAT8, ANNEXIN 8.**

2.912	AT2G37170	PIP2;2, PIP2B, PLASMA MEMBRANE INTRINSIC PROTEIN 2, PLASMA MEMBRANE INTRINSIC PROTEIN 2;2.
2.755	AT1G53310	ATPEPC1, ATPPC1, PEP(PHOSPHOENOLPYRUVATE) CARBOXYLASE 1, PEPC1, PHOSPHOENOLPYRUVATE CARBOXYLASE 1, PPC1.
2.725	AT4G24020	NIN LIKE PROTEIN 7, NLP7.
2.697	AT1G08720	ATEDR1, EDR1, ENHANCED DISEASE RESISTANCE 1.
2.665	AT4G18950	Integrin-linked protein kinase family.
2.583	AT2G46680	ARABIDOPSIS THALIANA HOMEOBOX 7, ATHB-7, ATHB7, HB-7, HOMEOBOX 7.
2.576	AT2G47800	ABCC4, ATMRP4, ATP-BINDING CASSETTE C4, EST3, MRP4, MULTIDRUG RESISTANCE-ASSOCIATED PROTEIN 4.
2.565	AT2G17820	AHK1, ATHK1, HISTIDINE KINASE 1, HK1.
2.537	AT1G02730	ATCSLD5, CELLULOSE SYNTHASE LIKE D5, CELLULOSE SYNTHASE-LIKE D5, CSLD5, SALT OVERLY SENSITIVE 6, SOS6.
2.527	AT5G39610	ANAC092, ARABIDOPSIS NAC DOMAIN CONTAINING PROTEIN 92, ATNAC2, ATNAC6, NAC DOMAIN CONTAINING PROTEIN 2, NAC DOMAIN CONTAINING PROTEIN 6, NAC2, NAC6, ORE1, ORESARA 1.
2.521	AT1G64660	ATMGL, METHIONINE GAMMA-LYASE, MGL.
2.493	AT5G52300	LOW-TEMPERATURE-INDUCED 65, LTI65, RD29B, RESPONSIVE TO DESSICATION 29B.
2.482	AT1G27320	AHK3, HISTIDINE KINASE 3, HK3.
2.474	AT3G17770	Dihydroxyacetone kinase.
2.458	AT1G20440	ATCOR47, COLD-REGULATED 47, COR47, RD17.
2.449	AT5G35750	AHK2, HISTIDINE KINASE 2, HK2.
2.449	AT1G04400	AT-PHH1, ATCRY2, CRY2, CRYPTOCHROME 2, FHA, PHH1.
2.446	AT2G45960	ATHH2, NAMED PLASMA MEMBRANE INTRINSIC PROTEIN 1;2, PIP1;2, PIP1B, PLASMA MEMBRANE INTRINSIC PROTEIN 1B, TMP-A, TRANSMEMBRANE PROTEIN A.
2.445	AT5G26340	ATSTP13, MSS1, STP13, SUGAR TRANSPORT PROTEIN 13.
2.389	AT5G54730	ARABIDOPSIS THALIANA HOMOLOG OF YEAST AUTOPHAGY 18 (ATG18) F, ATATG18F, ATG18F, G18F, HOMOLOG OF YEAST AUTOPHAGY 18 (ATG18) F.
2.367	AT2G45960	ATHH2, NAMED PLASMA MEMBRANE INTRINSIC PROTEIN 1;2, PIP1;2, PIP1B, PLASMA MEMBRANE INTRINSIC PROTEIN 1B, TMP-A, TRANSMEMBRANE PROTEIN A.
2.366	AT5G67340	ARM repeat superfamily protein.
2.359	AT2G43020	ATPAO2, PAO2, POLYAMINE OXIDASE 2.
2.357	AT4G39090	RD19, RD19A, RESPONSIVE TO DEHYDRATION 19, RESPONSIVE TO DEHYDRATION 19A.
2.324	AT5G13820	ATBP-1, ATBP1, ATTBP1, H-PROTEIN PROMOTE, HPPBF-1, TBP1, TELOMERIC DNA BINDING PROTEIN 1.
2.316	AT5G13330	RAP2.6L, RELATED TO AP2 6L.
2.258	AT3G50970	LOW TEMPERATURE-INDUCED 30, LTI30, XERO2.
2.258	AT1G54160	"NUCLEAR FACTOR Y, SUBUNIT A5", NF-YA5, NFYA5, NUCLEAR FACTOR Y A5.
2.255	AT5G60410	ATSIZ1, SIZ1.
2.240	AT5G40390	RAFFINOSE SYNTHASE 5, RS5, SEED IMBIBITION 1-LIKE, SIP1.
2.207	AT5G45710	AT-HSFA4C, HEAT SHOCK TRANSCRIPTION FACTOR A4C, HSFA4C, RHA1, ROOT HANDEDNESS 1.
2.201	AT4G35300	TMT2, TONOPLAST MONOSACCHARIDE TRANSPORTER2.
2.176	AT3G27690	LHCB2, LHCB2.3, LHCB2.4, LIGHT-HARVESTING CHLOROPHYLL B-BINDING 2, PHOTOSYSTEM II LIGHT HARVESTING COMPLEX GENE 2.3.
2.160	AT3G06010	ATCHR12, CHR12, CHROMATIN REMODELING 12.
2.146	AT4G02280	ATSUS3, SUCROSE SYNTHASE 3, SUS3.

2.130	AT1G47128	RD21, RD21A, RESPONSIVE TO DEHYDRATION 21, RESPONSIVE TO DEHYDRATION 21A.
2.124	AT1G12110	ARABIDOPSIS THALIANA NITRATE TRANSPORTER 1, ATNRT1, B-1, CHL1, CHL1-1, CHLORINA 1, NITRATE TRANSPORTER 1, NITRATE TRANSPORTER 1.1, NRT1, NRT1.1.
2.122	AT3G01650	RGLG1, RING DOMAIN LIGASE1.
2.094	AT5G58350	WITH NO LYSINE (K) KINASE 4, WNK4, ZIK2.
2.086	AT2G18050	HIS1-3, HISTONE H1-3.
2.084	AT5G08490	SLG1, SLOW GROWTH 1.
2.081	AT1G56600	ATGOLS2, GALACTINOL SYNTHASE 2, GOLS2.
2.064	AT3G14050	AT-RSH2, ATRSH2, RELA-SPOT HOMOLOG 2, RELA/SPOT HOMOLOG 2, RSH2.
2.061	AT4G26080	ABA INSENSITIVE 1, ABI1, ATABI1.
2.039	AT3G23920	ATBAM1, BAM1, BETA-AMYLASE 1, BETA-AMYLASE 7, BMY7, TR-BAMY.
2.038	AT1G29670	GDSL-like Lipase/Acylhydrolase superfamily protein.
2.036	AT3G05740	RECQ HELICASE L1, RECQI1.
2.024	AT2G33380	ARABIDOPSIS THALIANA CALEOSIN 3, ATCLO3, CALEOSIN 3, CLO-3, CLO3, RD20, RESPONSIVE TO DESSICATION 20.
2.001	AT5G48400	ATGLR1.2, GLR1.2, GLUTAMATE RECEPTOR 1.2.
		**Response to wounding**
11.125	AT5G46050	ARABIDOPSIS THALIANA PEPTIDE TRANSPORTER 3, ATPTR3, PEPTIDE TRANSPORTER 3, PTR3.
10.681	AT2G22330	"CYTOCHROME P450, FAMILY 79, SUBFAMILY B, POLYPEPTIDE 3", CYP79B3.
5.144	AT3G22400	ARABIDOPSIS THALIANA LIPOXYGENASE 5, ATLOX5, LOX5.
4.618	AT1G52200	PLAC8 family protein.
4.255	AT2G46370	ATGH3.11, FAR-RED INSENSITIVE 219, FIN219, JAR1, JASMONATE RESISTANT 1.
3.781	AT2G02220	ATPSKR1, PHYTOSULFOKIN RECEPTOR 1, PSKR1.
3.518	AT5G38710	Methylenetetrahydrofolate reductase family protein.
3.477	AT1G01120	3-KETOACYL-COA SYNTHASE 1, KCS1.
3.377	AT5G04230	ATPAL3, PAL3, PHENYL ALANINE AMMONIA-LYASE 3.
3.231	AT1G55280	Lipase/lipooxygenase.
3.004	AT1G71697	ATCK1, CHOLINE KINASE, CHOLINE KINASE 1, CK, CK1.
2.665	AT4G18950	Integrin-linked protein kinase family.
2.576	AT2G47800	ABCC4, ATMRP4, ATP-BINDING CASSETTE C4, EST3, MRP4, MULTIDRUG RESISTANCE-ASSOCIATED PROTEIN 4.
2.561	AT3G48360	ATBT2, BT2, BTB AND TAZ DOMAIN PROTEIN 2.
2.529	AT1G19660	ATBBD2, BBD2, BIFUNCTIONAL NUCLEASE IN BASAL DEFENSE RESPONSE 2.
2.455	AT4G13660	ATPRR2, PINORESINOL REDUCTASE 2, PRR2.
2.383	AT1G51680	4-COUMARATE:COA LIGASE 1, 4CL.1, 4CL1, ARABIDOPSIS THALIANA 4-COUMARATE:COA LIGASE 1, AT4CL1.
2.366	AT5G67340	ARM repeat superfamily protein.
2.359	AT4G21390	B120.
2.348	AT5G05140	Transcription elongation factor (TFIIS) family protein.
2.332	AT3G61060	ATPP2-A13, PHLOEM PROTEIN 2-A13, PP2-A13.
2.301	AT5G22500	FAR1, FATTY ACID REDUCTASE 1.
2.280	AT3G23610	DSPTP1, DUAL SPECIFICITY PROTEIN PHOSPHATASE 1.
2.266	AT1G05630	5PTASE13, ARABIDOPSIS THALIANA INOSITOL-POLYPHOSPHATE 5-PHOSPHATASE 13, AT5PTASE13, INOSITOL-POLYPHOSPHATASE 5-PHOSPHATASE 13.
2.242	AT3G16340	ABCG29, ATABCG29, ATP-BINDING CASSETTE G29, ATPDR1, PDR1, PLEIOTROPIC DRUG

		**RESISTANCE 1.**
2.179	AT1G51400	**Photosystem II 5 kD protein.**
2.155	AT3G59050	**ATPAO3, PAO3, POLYAMINE OXIDASE 3.**
2.150	AT5G61600	**ERF104, ETHYLENE RESPONSE FACTOR 104.**
2.092	AT5G63770	**ATDGK2, DGK2, DIACYLGLYCEROL KINASE 2.**
2.064	AT3G14050	**AT-RSH2, ATRSH2, RELA-SPOT HOMOLOG 2, RELA/SPOT HOMOLOG 2, RSH2.**
2.061	AT4G26080	**ABA INSENSITIVE 1, ABI1, ATABI1.**
2.047	AT2G26170	**CYP711A1, CYTOCHROME P450, FAMILY 711, SUBFAMILY A, POLYPEPTIDE 1, MAX1, MORE AXILLARY BRANCHES 1.**
2.030	AT5G04230	**ATPAL3, PAL3, PHENYL ALANINE AMMONIA-LYASE 3.**
		**Response to osmotic stress**
7.650	AT2G40180	**ATHPP2C5, PHOSPHATASE 2C5, PP2C5.**
5.447	AT1G15100	**RHA2A, RING-H2 FINGER A2A.**
3.790	AT5G52310	**COLD REGULATED 78, COR78, LOW-TEMPERATURE-INDUCED 78, LTI140, LTI78, RD29A, RESPONSIVE TO DESSICATION 29A.**
3.518	AT5G38710	**Methylenetetrahydrofolate reductase family protein.**
3.299	AT2G04240	**XERICO.**
3.288	AT1G53300	**TETRATRICOPEPTIDE-REPEAT THIOREDOXIN-LIKE 1, TTL1.**
2.621	AT5G13170	**ATSWEET15, SAG29, SENESCENCE-ASSOCIATED GENE 29, SWEET15.**
2.565	AT2G17820	**AHK1, ATHK1, HISTIDINE KINASE 1, HK1.**
2.537	AT1G02730	**ATCSLD5, CELLULOSE SYNTHASE LIKE D5, CELLULOSE SYNTHASE-LIKE D5, CSLD5, SALT OVERLY SENSITIVE 6, SOS6.**
2.482	AT1G27320	**AHK3, HISTIDINE KINASE 3, HK3.**
2.461	AT2G47900	**ATTLP3, TLP3, TUBBY LIKE PROTEIN 3.**
2.458	AT1G20440	**ATCOR47, COLD-REGULATED 47, COR47, RD17.**
2.449	AT5G35750	**AHK2, HISTIDINE KINASE 2, HK2.**
2.417	AT1G10210	**ATMPK1, MITOGEN-ACTIVATED PROTEIN KINASE 1, MPK1.**
2.370	AT2G23030	**SNF1-RELATED PROTEIN KINASE 2.9, SNRK2-9, SNRK2.9, SUCROSE NONFERMENTING 1-RELATED PROTEIN KINASE 2-9.**
2.357	AT4G39090	**RD19, RD19A, RESPONSIVE TO DEHYDRATION 19, RESPONSIVE TO DEHYDRATION 19A.**
2.319	AT3G58620	**TETRATRICOPETIDE-REPEAT THIOREDOXIN-LIKE 4, TTL4.**
2.314	AT1G58200	**MSCS-LIKE 3, MSL3.**
2.285	AT5G49450	**ATBZIP1, BASIC LEUCINE-ZIPPER 1, BZIP1.**
2.218	AT2G40190	**LEAF WILTING 3, LEW3.**
2.184	AT5G03280	**ATEIN2, CKR1, CYTOKININ RESISTANT 1, EIN2, ENHANCED RESPONSE TO ABA3, ERA3, ETHYLENE INSENSITIVE 2, ORE2, ORE3, ORESARA 2, ORESARA 3, PIR2.**
2.138	AT1G78290	**SNF1-RELATED PROTEIN KINASE 2-8, SNF1-RELATED PROTEIN KINASE 2.8, SNF1-RELATED PROTEIN KINASE 2C, SNRK2-8, SNRK2.8, SRK2C.**
2.098	AT1G35515	**HIGH RESPONSE TO OSMOTIC STRESS 10, HOS10, MYB8.**
2.000	AT3G09260	**BGLU23, LEB, LONG ER BODY, PSR3.1, PYK10.**
		**Response to salt stress**
173.307	AT2G20880	**ATERF53, ERF DOMAIN 53, ERF53.**
7.690	AT2G36270	**ABA INSENSITIVE 5, ABI5, GIA1, GROWTH-INSENSITIVITY TO ABA 1.**
6.681	AT5G59780	**ATMYB59, ATMYB59-1, ATMYB59-2, ATMYB59-3, MYB DOMAIN PROTEIN 59, MYB59.**
5.447	AT1G15100	**RHA2A, RING-H2 FINGER A2A.**

5.083	AT5G44190	**ATGLK2, GBF'S PRO-RICH REGION-INTERACTING FACTOR 2, GLK2, GOLDEN2-LIKE 2, GPRI2**.
4.622	AT2G26650	**AKT1, ATAKT1, K+ TRANSPORTER 1, KT1**.
3.790	AT5G52310	**COLD REGULATED 78, COR78, LOW-TEMPERATURE-INDUCED 78, LTI140, LTI78, RD29A, RESPONSIVE TO DESSICATION 29A**.
3.672	AT4G11650	**ATOSM34, OSM34, OSMOTIN 34**.
3.484	AT5G25370	**PHOSPHOLIPASE D ALPHA 3, PLDALPHA3**.
3.483	AT4G24800	**ECIP1, EIN2 C-TERMINUS INTERACTING PROTEIN 1**.
3.299	AT2G04240	**XERICO**.
3.288	AT1G53300	**TETRATRICOPEPTIDE-REPEAT THIOREDOXIN-LIKE 1, TTL1**.
3.066	AT2G31180	**ARABIDOPSIS THALIANA MYB DOMAIN PROTEIN 14, ATMYB14, MYB DOMAIN PROTEIN 14, MYB14, MYB14AT**.
3.045	AT3G61430	**ARABIDOPSIS THALIANA PLASMA MEMBRANE INTRINSIC PROTEIN 1, ATPIP1, PIP1, PIP1;1, PIP1A, PLASMA MEMBRANE INTRINSIC PROTEIN 1, PLASMA MEMBRANE INTRINSIC PROTEIN 1;1, PLASMA MEMBRANE INTRINSIC PROTEIN 1A**.
3.018	AT4G03400	**DFL2, DWARF IN LIGHT 2, GH3-10.**
2.983	AT5G12380	**ANNAT8, ANNEXIN 8.**
2.925	AT1G56570	**PENTATRICOPEPTIDE REPEAT PROTEIN FOR GERMINATION ON NACL, PGN**.
2.836	AT4G15430	**ERD (early-responsive to dehydration stress) family protein.**
2.755	AT1G53310	**ATPEPC1, ATPPC1, PEP(PHOSPHOENOLPYRUVATE) CARBOXYLASE 1, PEPC1, PHOSPHOENOLPYRUVATE CARBOXYLASE 1, PPC1.**
2.751	AT5G38895	**RING/U-box superfamily protein.**
2.740	AT5G63320	**NPX1, NUCLEAR PROTEIN X1**.
2.723	AT5G04140	**FD-GOGAT, FERREDOXIN-DEPENDENT GLUTAMATE SYNTHASE, FERREDOXIN-DEPENDENT GLUTAMATE SYNTHASE 1, GLS1, GLU1, GLUS, GLUTAMATE SYNTHASE 1.**
2.612	AT1G61210	**DWA3, DWD HYPERSENSITIVE TO ABA 3.**
2.561	AT3G48360	**ATBT2, BT2, BTB AND TAZ DOMAIN PROTEIN 2.**
2.537	AT1G02730	**ATCSLD5, CELLULOSE SYNTHASE LIKE D5, CELLULOSE SYNTHASE-LIKE D5, CSLD5, SALT OVERLY SENSITIVE 6, SOS6.**
2.527	AT5G39610	**ANAC092, ARABIDOPSIS NAC DOMAIN CONTAINING PROTEIN 92, ATNAC2, ATNAC6, NAC DOMAIN CONTAINING PROTEIN 2, NAC DOMAIN CONTAINING PROTEIN 6, NAC2, NAC6, ORE1, ORESARA 1.**
2.493	AT5G52300	**LOW-TEMPERATURE-INDUCED 65, LTI65, RD29B, RESPONSIVE TO DESSICATION 29B.**
2.482	AT1G27320	**AHK3, HISTIDINE KINASE 3, HK3.**
2.461	AT2G47900	**ATTLP3, TLP3, TUBBY LIKE PROTEIN 3.**
2.458	AT1G20440	**ATCOR47, COLD-REGULATED 47, COR47, RD17.**
2.449	AT5G35750	**AHK2, HISTIDINE KINASE 2, HK2.**
2.446	AT2G45960	**ATHH2, NAMED PLASMA MEMBRANE INTRINSIC PROTEIN 1;2, PIP1;2, PIP1B, PLASMA MEMBRANE INTRINSIC PROTEIN 1B, TMP-A, TRANSMEMBRANE PROTEIN A**.
2.445	AT5G26340	**ATSTP13, MSS1, STP13, SUGAR TRANSPORT PROTEIN 13.**
2.435	AT3G02250	**O-fucosyltransferase family protein**.
2.432	AT5G14640	**ATSK13, SHAGGY-LIKE KINASE 13, SK13.**
2.417	AT1G10210	**ATMPK1, MITOGEN-ACTIVATED PROTEIN KINASE 1, MPK1.**
2.367	AT2G45960	**ATHH2, NAMED PLASMA MEMBRANE INTRINSIC PROTEIN 1;2, PIP1;2, PIP1B, PLASMA MEMBRANE INTRINSIC PROTEIN 1B, TMP-A, TRANSMEMBRANE PROTEIN A**.
2.357	AT4G39090	**RD19, RD19A, RESPONSIVE TO DEHYDRATION 19, RESPONSIVE TO DEHYDRATION 19A.**
2.340	AT2G30520	**ROOT PHOTOTROPISM 2, RPT2.**

2.316	AT5G13330	**RAP2.6L, RELATED TO AP2 6L.**
2.301	AT5G22500	**FAR1, FATTY ACID REDUCTASE 1.**
2.291	AT4G24560	**UBIQUITIN-SPECIFIC PROTEASE 16, UBP16.**
2.285	AT5G49450	**ATBZIP1, BASIC LEUCINE-ZIPPER 1, BZIP1.**
2.281	AT4G18710	**ATSK21, BIN2, BRASSINOSTEROID-INSENSITIVE 2, DWARF 12, DWF12, SHAGGY-LIKE KINASE 21, SK21, UCU1, ULTRACURVATA 1.**
2.280	AT3G23610	**DSPTP1, DUAL SPECIFICITY PROTEIN PHOSPHATASE 1.**
2.276	AT2G16500	**ADC1, ARGDC, ARGDC1, ARGININE DECARBOXYLASE 1, SPE1**.
2.266	AT2G36830	**GAMMA TONOPLAST INTRINSIC PROTEIN, GAMMA TONOPLAST INTRINSIC PROTEIN 1, GAMMA-TIP, GAMMA-TIP1, TIP1;1, TONOPLAST INTRINSIC PROTEIN 1;1.**
2.237	AT5G19330	**ARIA, ARM REPEAT PROTEIN INTERACTING WITH ABF2.**
2.230	AT4G39100	**SHL1, SHORT LIFE.**
2.184	AT5G03280	**ATEIN2, CKR1, CYTOKININ RESISTANT 1, EIN2, ENHANCED RESPONSE TO ABA3, ERA3, ETHYLENE INSENSITIVE 2, ORE2, ORE3, ORESARA 2, ORESARA 3, PIR2**.
2.160	AT3G06010	**ATCHR12, CHR12, CHROMATIN REMODELING 12**.
2.130	AT1G47128	**RD21, RD21A, RESPONSIVE TO DEHYDRATION 21, RESPONSIVE TO DEHYDRATION 21A**.
2.122	AT3G47950	**AHA4, H(+)-ATPASE 4, HA4.**
2.098	AT1G35515	**HIGH RESPONSE TO OSMOTIC STRESS 10, HOS10, MYB8.**
2.081	AT1G56600	**ATGOLS2, GALACTINOL SYNTHASE 2, GOLS2.**
2.050	AT2G17270	**MITOCHONDRIAL PHOSPHATE TRANSPORTER 1, MPT1, PHOSPHATE TRANSPORTER 3;3, PHT3;3**.
2.024	AT2G33380	**ARABIDOPSIS THALIANA CALEOSIN 3, ATCLO3, CALEOSIN 3, CLO-3, CLO3, RD20, RESPONSIVE TO DESSICATION 20.**
2.000	AT3G09260	**BGLU23, LEB, LONG ER BODY, PSR3.1, PYK10.**
		**Response to mechanical stimulus**
8.139	AT5G15830	**ATBZIP3, BASIC LEUCINE-ZIPPER 3, BZIP3**.
3.006	AT1G08320	**BZIP21, TGA9, TGACG (TGA) MOTIF-BINDING PROTEIN 9**.
2.708	AT1G58110	**Basic-leucine zipper (bZIP) transcription factor family protein.**
2.150	AT5G61600	**ERF104, ETHYLENE RESPONSE FACTOR 104.**
		**Response to UV-B**
4.255	AT2G46370	**ATGH3.11, FAR-RED INSENSITIVE 219, FIN219, JAR1, JASMONATE RESISTANT 1**.
2.614	AT2G24540	**AFR, ATTENUATED FAR-RED RESPONSE.**
2.313	AT3G28030	**ULTRAVIOLET HYPERSENSITIVE 3, UV REPAIR DEFECTIVE 1, UVH3, UVR1.**
2.179	AT1G51400	**Photosystem II 5 kD protein**.
2.161	AT2G43800	**Actin-binding FH2 (formin homology 2) family protein.**
