# Supplementary material for: Transcriptomic profiling of linolenic acid-responsive genes in ROS signaling from RNA-seq data in Arabidopsis
Source: Front Plant Sci. 2015 Mar 17;6:122. doi: 10.3389/fpls.2015.00122 (PMC4362301; doi:10.3389/fpls.2015.00122)
Supplement: Supplemental Table 1 — Fatty acid composition of Arabidopsis thaliana cell suspension cultures (ACSC). [file DataSheet1.ZIP › Table 6.PDF]

Oxidative stress-related genes (Control vs Ln UP)

FC	ID	Oxidative stress
92,539	AT4G21830	MSRB7.
24,725	AT2G47180	ATGOLS1, GALACTINOL SYNTHASE 1, GOLS1.
12,267	AT1G32640	ATMYC2, JAI1, JASMONATE INSENSITIVE 1, JIN1, MYC2, RD22BP1, ZBF1.
8,056	AT5G20230	ATBCB, BCB, BLUE COPPER BINDING PROTEIN, BLUE-COPPER-BINDING PROTEIN, SAG14, SENESCENCE ASSOCIATED GENE 14.
7,230	AT5G59820	ATZAT12, RESPONSIVE TO HIGH LIGHT 41, RHL41, ZAT12.
5,775	AT1G27730	SALT TOLERANCE ZINC FINGER, STZ, ZAT10.
4,901	AT5G16980	Zinc-binding dehydrogenase family protein.
4,396	AT3G25250	AGC2, AGC2-1, ATOXI1, OXI1, OXIDATIVE SIGNAL-INDUCIBLE1.
3,783	AT2G29450	ARABIDOPSIS THALIANA GLUTATHIONE S-TRANSFERASE TAU 1, AT103-1A, ATGSTU1, ATGSTU5, GLUTATHIONE S-TRANSFERASE TAU 5, GSTU5.
3,764	AT1G09350	ATGOLS3, GALACTINOL SYNTHASE 3, GOLS3.
3,300	AT1G30460	ARABIDOPSIS THALIANA CLEAVAGE AND POLYADENYLATION SPECIFICITY FACTOR 30, ATCPSF30, CLEAVAGE AND POLYADENYLATION SPECIFICITY FACTOR 30, CPSF30.
2,646	AT5G17000	Zinc-binding dehydrogenase family protein.
2,448	AT5G16970	AER, ALKENAL REDUCTASE, AT-AER.
2,213	AT4G11280	1-AMINOCYCLOPROPANE-1-CARBOXYLIC ACID (ACC) SYNTHASE 6, ACS6, ATACS6.
2,117	AT3G28200	Peroxidase superfamily protein.
		**Response to hydrogen peroxide**
24,725	AT2G47180	ATGOLS1, GALACTINOL SYNTHASE 1, GOLS1.
14,799	AT1G52560	HSP20-like chaperones superfamily protein.
14,151	AT4G12400	HOP3.
12,125	AT5G05410	DEHYDRATION-RESPONSIVE ELEMENT BINDING PROTEIN 2, DRE-BINDING PROTEIN 2A, DREB2, DREB2A.
11,405	AT2G26150	ATHSFA2, HEAT SHOCK TRANSCRIPTION FACTOR A2, HSFA2.
9,597	AT4G25200	ATHSP23.6-MITO, HSP23.6-MITO, MITOCHONDRION-LOCALIZED SMALL HEAT SHOCK PROTEIN 23.6.
7,912	AT5G63160	BT1, BTB AND TAZ DOMAIN PROTEIN 1.
5,347	AT5G37770	CALMODULIN-LIKE 24, CML24, TCH2, TOUCH 2.
4,975	AT2G46240	ARABIDOPSIS THALIANA BCL-2-ASSOCIATED ATHANOGENE 6, ATBAG6, BAG6, BCL-2-ASSOCIATED ATHANOGENE 6.
4,870	AT2G20560	DNAJ heat shock family protein.
4,106	AT2G32120	HEAT-SHOCK PROTEIN 70T-2, HSP70T-2.
4,035	AT1G74310	ATHSP101, HEAT SHOCK PROTEIN 101, HOT1, HSP101.
3,260	AT1G14200	RING/U-box superfamily protein.
3,129	AT3G09350	Fes1A.
3,012	AT5G37670	HSP20-like chaperones superfamily protein.
2,873	AT3G16050	A37, ARABIDOPSIS THALIANA PYRIDOXINE BIOSYNTHESIS 1.2, ATPDX1.2, PDX1.2, PYRIDOXINE BIOSYNTHESIS 1.2.
2,674	AT1G17870	ATEGY3, EGY3, ETHYLENE-DEPENDENT GRAVITROPISM-DEFICIENT

		**AND YELLOW-GREEN-LIKE 3**.
2,233	AT4G21320	**HEAT-STRESS-ASSOCIATED 32, HSA32**.
2,089	AT1G16030	**HEAT SHOCK PROTEIN 70B, HSP70B**.
		**Carbonyls**
2,728	AT3G61220	**SDR1, SHORT-CHAIN DEHYDROGENASE/REDUCTASE 1**.
2,448	AT5G16970	**AER, ALKENAL REDUCTASE, AT-AER**.
		**Oxidation-reduction process**
36,680	AT5G05600	**2-oxoglutarate (2OG) and Fe(II)-dependent oxygenase superfamily protein.**
18,400	AT2G38240	**2-oxoglutarate (2OG) and Fe(II)-dependent oxygenase superfamily protein.**
15,903	AT5G06900	**"CYTOCHROME P450, FAMILY 93, SUBFAMILY D, POLYPEPTIDE 1", CYP93D1**.
14,502	AT1G06620	**Encodes a protein whose sequence is similar to a 2-oxoglutarate-dependent dioxygenase.**
9,711	AT5G42650	**ALLENE OXIDE SYNTHASE, AOS, CYP74A, CYTOCHROME P450 74A, DDE2, DELAYED DEHISCENCE 2.**
8,666	AT4G19230	**"CYTOCHROME P450, FAMILY 707, SUBFAMILY A, POLYPEPTIDE 1", CYP707A1**.
6,509	AT1G43800	**FLORAL TRANSITION AT THE MERISTEM1, FTM1**.
6,294	AT1G64950	**"CYTOCHROME P450, FAMILY 89, SUBFAMILY A, POLYPEPTIDE 5", CYP89A5**.
5,846	AT2G27690	**"CYTOCHROME P450, FAMILY 94, SUBFAMILY C, POLYPEPTIDE 1", CYP94C1**.
5,603	AT2G06050	**ATOPR3, DDE1, DELAYED DEHISCENCE 1, OPR3, OXOPHYTODIENOATE-REDUCTASE 3**.
4,901	AT5G16980	**Zinc-binding dehydrogenase family protein**.
4,680	AT5G63450	**"CYTOCHROME P450, FAMILY 94, SUBFAMILY B, POLYPEPTIDE 1", CYP94B1.**
4,051	AT5G43040	**Cysteine/Histidine-rich C1 domain family protein.**
3,846	AT3G09940	**ARABIDOPSIS THALIANA MONODEHYDROASCORBATE REDUCTASE 3, ATMDAR3, MDAR2, MDAR3, MDHAR, MONODEHYDROASCORBATE REDUCTASE, MONODEHYDROASCORBATE REDUCTASE 2, MONODEHYDROASCORBATE REDUCTASE 3.**
3,748	AT3G07000	**Cysteine/Histidine-rich C1 domain family protein**.
3,493	AT5G47990	**"CYTOCHROME P450, FAMILY 705, SUBFAMILY A, POLYPEPTIDE 5", CYP705A5, THAD, THAD1, THALIAN-DIOL DESATURASE.**
3,375	AT3G27500	**Cysteine/Histidine-rich C1 domain family protein**.
3,182	AT1G32350	**ALTERNATIVE OXIDASE 1D, AOX1D**.
3,000	AT1G06120	**Delta-9 desaturase-like 3 protein**.
2,877	AT4G15393	**"CYTOCHROME P450, FAMILY 702, SUBFAMILY A, POLYPEPTIDE 5", CYP702A5**.
2,827	AT4G21990	**APR3, APS REDUCTASE 3, ATAPR3, PAPS REDUCTASE HOMOLOG 26, PRH-26, PRH26**.
2,728	AT3G61220	**SDR1, SHORT-CHAIN DEHYDROGENASE/REDUCTASE 1**.
2,646	AT5G17000	**Zinc-binding dehydrogenase family protein**.
2,590	AT5G06905	**"CYTOCHROME P450, FAMILY 712, SUBFAMILY A, POLYPEPTIDE 2", CYP712A2**.

2,587	AT1G76690	**12-OXOPHYTODIENOATE REDUCTASE 2, ARABIDOPSIS 12-OXOPHYTODIENOATE REDUCTASE 2, ATOPR2, OPR2**.
2,354	AT3G06990	**Cysteine/Histidine-rich C1 domain family protein**.
2,351	AT3G44190	**FAD/NAD(P)-binding oxidoreductase family protein**.
2,335	AT4G31500	**"CYTOCHROME P450, FAMILY 83, SUBFAMILY B, POLYPEPTIDE 1", ALTERED TRYPTOPHAN REGULATION 4, ATR4, CYP83B1, RED ELONGATED 1, RED1, RNT1, RUNT 1, SUPERROOT 2, SUR2.**
2,281	AT1G62560	**FLAVIN-MONOOXYGENASE GLUCOSINOLATE S-OXYGENASE 3, FMO GS-OX3.**
2,185	AT4G21990	**APR3, APS REDUCTASE 3, ATAPR3, PAPS REDUCTASE HOMOLOG 26, PRH-26, PRH26**.
2,117	AT3G28200	**Peroxidase superfamily protein**.
2,019	AT5G25180	**"CYTOCHROME P450, FAMILY 71, SUBFAMILY B, POLYPEPTIDE 14", CYP71B14**.
		**Cell redox homeostasis**
6,363	AT4G29670	**ACHT2, ATYPICAL CYS HIS RICH THIOREDOXIN 2**.
2,827	AT4G21990	**APR3, APS REDUCTASE 3, ATAPR3, PAPS REDUCTASE HOMOLOG 26, PRH-26, PRH26**.
2,463	AT4G31360	**Selenium binding protein**. Involved in: cell redox homeostasis.
		**Peroxidase**
5,081	AT2G47270	**UPB1, UPBEAT1**.
2,117	AT3G28200	**Peroxidase superfamily protein**.

Oxidative stress-related genes (Control vs Ln DOWN)

FC	ID	Oxidative stress
8.358	AT2G19810	**ATOZF1, ATTZF2, OXIDATION-RELATED ZINC FINGER 1, OZF1, TANDEM ZINC FINGER 2, TZF2**.
4.618	AT1G52200	**PLAC8 family protein**.
3.705	AT4G16270	**Peroxidase superfamily protein.**
3.422	AT1G14540	**PER4, PEROXIDASE 4**.
3.407	AT5G14130	**Peroxidase superfamily protein.**
3.061	AT2G37130	**Peroxidase superfamily protein**.
3.041	AT2G41900	**OXIDATIVE STRESS 2, OXS2**.
2.931	AT5G56550	**ATOXS3, OXIDATIVE STRESS 3, OXS3.**
2.702	AT5G37980	**Zinc-binding dehydrogenase family protein.**
2.680	AT4G30170	**Peroxidase family protein**.
2.557	AT3G49160	Expression of the gene is downregulated in the presence of paraquat, an inducer of photoxidative stress. Involved in: glycolysis.
2.527	AT5G39610	**ANAC092, ARABIDOPSIS NAC DOMAIN CONTAINING PROTEIN 92, ATNAC2, ATNAC6, NAC DOMAIN CONTAINING PROTEIN 2, NAC DOMAIN CONTAINING PROTEIN 6, NAC2, NAC6, ORE1, ORESARA 1.**
2.365	AT2G22420	**Peroxidase superfamily protein.**
2.359	AT4G21390	**B120**.
2.276	AT2G16500	**ADC1, ARGDC, ARGDC1, ARGININE DECARBOXYLASE 1, SPE1**.
2.240	AT5G40390	**RAFFINOSE SYNTHASE 5, RS5, SEED IMBIBITION 1-LIKE, SIP1.**
2.195	AT2G15560	**Putative endonuclease or glycosyl hydrolase**.
2.184	AT5G03280	**ATEIN2, CKR1, CYTOKININ RESISTANT 1, EIN2, ENHANCED RESPONSE TO ABA3, ERA3, ETHYLENE INSENSITIVE 2, ORE2, ORE3, ORESARA 2, ORESARA 3, PIR2**.
2.133	AT2G24150	**HEPTAHELICAL PROTEIN 3, HHP3**.
2.081	AT1G56600	**ATGOLS2, GALACTINOL SYNTHASE 2, GOLS2.**
2.079	AT4G11850	**MATERNAL EFFECT EMBRYO ARREST 54, MEE54, PHOSPHOLIPASE D GAMMA 1, PLDGAMMA1.**
2.066	AT5G42180	**PER64, PEROXIDASE 64.**
2.060	AT5G41150	**ATRAD1, RAD1, ULTRAVIOLET HYPERSENSITIVE 1, UVH1.**
2.000	AT5G47000	**Peroxidase superfamily protein**.
		**Response to hydrogen peroxide**
4.174	AT1G07390	**ATRLP1, RECEPTOR LIKE PROTEIN 1, RLP1**.
3.486	AT5G07340	**Calreticulin family protein.**
2.808	AT4G18450	Encodes a member of the **ERF (ethylene response factor) subfamily B-3 of ERF/AP2 transcription factor family**.
2.779	AT3G19930	**ATSTP4, STP4, SUGAR TRANSPORTER 4.**
2.681	AT4G24240	**ATWRKY7, WRKY DNA-BINDING PROTEIN 7, WRKY7.**
2.561	AT3G48360	**ATBT2, BT2, BTB AND TAZ DOMAIN PROTEIN 2.**
2.534	AT3G05660	**ATRLP33, RECEPTOR LIKE PROTEIN 33, RLP33**.
2.492	AT1G72660	**P-loop containing nucleoside triphosphate hydrolases superfamily protein.**
2.461	AT2G47900	**ATTLP3, TLP3, TUBBY LIKE PROTEIN 3.**
2.439	AT1G70520	**ALTERED SEED GERMINATION 6, ASG6, CRK2, CYSTEINE-RICH RLK (RECEPTOR-LIKE PROTEIN KINASE) 2.**

2.407	AT4G23810	**ATWRKY53, WRKY53.**
2.207	AT5G18750	**DNAJ heat shock N-terminal domain-containing protein.**
2.155	AT5G46350	**ARABIDOPSIS THALIANA WRKY DNA-BINDING PROTEIN 8, ATWRKY8, WRKY DNA-BINDING PROTEIN 8, WRKY8.**
2.094	AT3G46920	**Protein kinase superfamily protein with octicosapeptide/Phox/Bem1p domain.**
2.022	AT3G12570	**FYD.**
2.014	AT5G50380	**ATEXO70F1, EXO70F1, EXOCYST SUBUNIT EXO70 FAMILY PROTEIN F1.**
		**Response to superoxide**
3.420	AT3G63010	**ATGID1B, GA INSENSITIVE DWARF1B, GID1B.**
2.751	AT5G38895	**RING/U-box superfamily protein.**
2.527	AT5G39610	**ANAC092, ARABIDOPSIS NAC DOMAIN CONTAINING PROTEIN 92, ATNAC2, ATNAC6, NAC DOMAIN CONTAINING PROTEIN 2, NAC DOMAIN CONTAINING PROTEIN 6, NAC2, NAC6, ORE1, ORESARA 1.**
2.482	AT1G27320	**AHK3, HISTIDINE KINASE 3, HK3.**
2.456	AT4G29380	**ARABIDOPSIS THALIANA VACUOLAR PROTEIN SORTING 15, ATVPS15, VACUOLAR PROTEIN SORTING 15, VPS15.**
2.417	AT1G10210	**ATMPK1, MITOGEN-ACTIVATED PROTEIN KINASE 1, MPK1.**
2.175	AT1G64610	**Transducin/WD40 repeat-like superfamily protein.**
2.061	AT4G26080	**ABA INSENSITIVE 1, ABI1, ATABI1.**
		**Oxidation-reduction process**
28.366	AT2G07785	**NAD1.**
13.819	AT2G07689	**NADH-Ubiquinone/plastoquinone (complex I) protein.**
8.762	ATMG00650	**NAD4L, NADH DEHYDROGENASE SUBUNIT 4L.**
7.951	ATMG00285	**NAD2, NAD2.1, NAD2A, NADH DEHYDROGENASE 2, NADH DEHYDROGENASE 2.1, NADH DEHYDROGENASE 2A.**
7.429	AT4G16790	**NAD9, NADH DEHYDROGENASE SUBUNIT 9.**
7.363	AT1G74550	**CYP98A9, CYTOCHROME P450, FAMILY 98, SUBFAMILY A, POLYPEPTIDE 9.**
6.191	AT4G12330	**"CYTOCHROME P450, FAMILY 706, SUBFAMILY A, POLYPEPTIDE 7", CYP706A7.**
6.092	ATMG01360	**COX1, CYTOCHROME OXIDASE 1.**
5.536	AT2G44380	**Cysteine/Histidine-rich C1 domain family protein.**
4.812	AT1G18270	**Ketose-bisphosphate aldolase class-II family protein.**
4.713	AT4G12320	**"CYTOCHROME P450, FAMILY 706, SUBFAMILY A, POLYPEPTIDE 6", CYP706A6.**
4.422	AT5G49730	**ATFRO6, FERRIC REDUCTION OXIDASE 6, FRO6.**
4.380	AT1G13150	**"CYTOCHROME P450, FAMILY 86, SUBFAMILY C, POLYPEPTIDE 4", CYP86C4.**
4.320	AT5G28310	**NAD(P)-binding Rossmann-fold superfamily protein.**
4.165	AT4G37310	**"CYTOCHROME P450, FAMILY 81, SUBFAMILY H, POLYPEPTIDE 1", CYP81H1.**
4.106	AT2G44370	**Cysteine/Histidine-rich C1 domain family protein.**
4.000	AT3G02590	**Fatty acid hydroxylase superfamily protein.**
3.972	AT1G67110	**"CYTOCHROME P450, FAMILY 735, SUBFAMILY A, POLYPEPTIDE 2", CYP735A2.**
3.953	AT4G16310	**LDL3, LSD1-LIKE 3.**
3.763	AT5G49740	**ATFRO7, FERRIC REDUCTION OXIDASE 7, FRO7.**
3.756	AT4G01740	**Cysteine/Histidine-rich C1 domain family protein.**
3.756	ATMG00513	**NAD5, NAD5.1, NAD5A, NADH DEHYDROGENASE 5.1, NADH DEHYDROGENASE 5A, NADH DEHYDROGENASE SUBUNIT 5.**
3.752	AT1G01580	**ATFRO2, FERRIC CHELATE REDUCTASE DEFECTIVE 1, FERRIC REDUCTION OXIDASE 2,**

		**FRD1, FRO2**.
3.705	AT4G16270	**Peroxidase superfamily protein.**
3.685	AT4G37320	**"CYTOCHROME P450, FAMILY 81, SUBFAMILY D, POLYPEPTIDE 5", CYP81D5**.
3.588	AT5G24900	**CYP714A2, CYTOCHROME P450, FAMILY 714, SUBFAMILY A, POLYPEPTIDE 2, ELA2, EUI-LIKE P450 A2**.
3.518	AT5G38710	**Methylenetetrahydrofolate reductase family protein**.
3.499	AT2G37820	**Cysteine/Histidine-rich C1 domain family protein.**
3.424	AT3G26160	**"CYTOCHROME P450, FAMILY 71, SUBFAMILY B, POLYPEPTIDE 17", CYP71B17.**
3.422	AT1G14540	**PER4, PEROXIDASE 4**.
3.407	AT5G14130	**Peroxidase superfamily protein.**
3.397	AT3G06690	**Acyl-CoA oxidases;oxidoreductases**, acting on the CH-CH group of donors.
3.366	AT4G12310	**"CYTOCHROME P450, FAMILY 706, SUBFAMILY A, POLYPEPTIDE 5", CYP706A5**.
3.264	ATCG00890	**NDHB.1**.
3.264	ATCG01250	**NDHB.2.**
3.257	AT4G31530	**NAD(P)-binding Rossmann-fold superfamily protein.**
3.256	AT2G33230	**YUC7, YUCCA 7.**
3.178	ATMG00580	**NAD4, NADH DEHYDROGENASE SUBUNIT 4.**
3.150	AT1G68080	**2-oxoglutarate (2OG) and Fe(II)-dependent oxygenase superfamily protein.**
3.118	AT4G08920	**ATCRY1, BLU1, BLUE LIGHT UNINHIBITED 1, CRY1, CRYPTOCHROME 1, ELONGATED HYPOCOTYL 4, HY4, OOP2, OUT OF PHASE 2**.
3.062	ATMG00990	**NAD3, NADH DEHYDROGENASE 3.**
3.061	AT2G37130	**Peroxidase superfamily protein**.
3.047	ATCG01090	**NDHI.**
3.039	AT5G35790	**G6PD1, GLUCOSE-6-PHOSPHATE DEHYDROGENASE 1**.
3.017	AT1G77330	**Similar to 1-aminocyclopropane-1-carboxylate oxidase**.
3.013	AT1G50560	**"CYTOCHROME P450, FAMILY 705, SUBFAMILY A, POLYPEPTIDE 25", CYP705A25.**
2.990	AT3G26125	**"CYTOCHROME P450, FAMILY 86, SUBFAMILY C, POLYPEPTIDE 2", CYP86C2.**
2.979	AT3G13730	**"CYTOCHROME P450, FAMILY 90, SUBFAMILY D, POLYPEPTIDE 1", CYP90D1.**
2.920	AT5G43520	**Cysteine/Histidine-rich C1 domain family protein**.
2.912	AT4G00360	**"CYTOCHROME P450, FAMILY 86, SUBFAMILY A, POLYPEPTIDE 2", ABERRANT INDUCTION OF TYPE THREE 1, ATT1, CYP86A2.**
2.830	ATMG00510	**NAD7, NADH DEHYDROGENASE SUBUNIT 7.**
2.829	AT4G33150	**LKR, LKR/SDH, LYSINE-KETOGLUTARATE REDUCTASE/SACCHAROPINE DEHYDROGENASE, SACCHAROPINE DEHYDROGENASE, SDH.**
2.802	AT1G04380	Encodes a protein similar to a **2-oxoglutarate-dependent dioxygenase**.
2.783	AT1G26420	**FAD-binding Berberine family protein.**
2.737	ATCG01080	**NDHG**.
2.723	AT5G04140	**FD-GOGAT, FERREDOXIN-DEPENDENT GLUTAMATE SYNTHASE, FERREDOXIN-DEPENDENT GLUTAMATE SYNTHASE 1, GLS1, GLU1, GLUS, GLUTAMATE SYNTHASE 1.**
2.702	AT5G37980	**Zinc-binding dehydrogenase family protein.**
2.702	ATCG01070	**NDHE.**
2.693	AT1G13710	**"CYTOCHROME P450, FAMILY 78, SUBFAMILY A, POLYPEPTIDE 5", CYP78A5, KLU, KLUH.**
2.680	AT4G30170	**Peroxidase family protein**.
2.657	AT1G55290	Encodes a protein whose sequence is similar to **oxidoreductase, 2OG-Fe(II) oxygenase**.
2.593	AT5G25900	**ARABIDOPSIS THALIANA ENT-KAURENE OXIDASE 1, ATKO1, CYP701A3, CYTOCHROME P450 701 A3, GA REQUIRING 3, GA3.**

2.586	AT4G13260	**ATYUC2, YUC2, YUCCA2.**
2.580	AT1G04580	**AAO4, ALDEHYDE OXIDASE 2, ALDEHYDE OXIDASE 4, AO4, ARABDOPSIS THALIANA ALDEHYDE OXIDASE 4, ATAO-4, ATAO2.**
2.576	AT1G05010	**ACO4, EAT1, EFE, ETHYLENE FORMING ENZYME, ETHYLENE-FORMING ENZYME**.
2.576	ATCG01100	**NDHA**.
2.570	AT1G69150	**Cysteine/Histidine-rich C1 domain family protein.**
2.557	AT5G21482	**ARABIDOPSIS THALIANA CYTOKININ OXIDASE 5, ATCKX5, CKX7, CYTOKININ OXIDASE 7.**
2.554	AT3G10390	**FLD, FLOWERING LOCUS D.**
2.542	AT2G46570	**LAC6, LACCASE 6.**
2.497	AT4G01925	**Cysteine/Histidine-rich C1 domain family protein.**
2.443	AT4G04610	**APR, APR1, APS REDUCTASE 1, ATAPR1, PAPS REDUCTASE HOMOLOG 19, PRH19.**
2.424	AT3G43670	**Copper amine oxidase family protein.**
2.424	AT5G10600	**"CYTOCHROME P450, FAMILY 81, SUBFAMILY K, POLYPEPTIDE 2", CYP81K2.**
2.422	AT5G25120	**"CYTOCHROME P450, FAMILY 71, SUBFAMILY B, POLYPEPTIDE 11", CYP71B11.**
2.399	AT1G13140	**"CYTOCHROME P450, FAMILY 86, SUBFAMILY C, POLYPEPTIDE 3", CYP86C3**.
2.399	AT1G30720	**FAD-binding Berberine family protein.**
2.387	AT1G17060	**CHI2, CHIBI 2, CYP72C1, CYTOCHROME P450 72C1, SHK1, SHRINK 1, SOB7, SUPPRESSOR OF PHYB-4 7.**
2.378	AT3G20080	**"CYTOCHROME P450, FAMILY 705, SUBFAMILY A, POLYPEPTIDE 15", CYP705A15.**
2.366	AT1G52800	**2-oxoglutarate (2OG) and Fe(II)-dependent oxygenase superfamily protein**.
2.365	AT2G22420	**Peroxidase superfamily protein.**
2.359	ATCG01050	**NDHD.**
2.359	AT2G43020	**ATPAO2, PAO2, POLYAMINE OXIDASE 2.**
2.347	AT4G33150	**LKR, LKR/SDH, LYSINE-KETOGLUTARATE REDUCTASE/SACCHAROPINE DEHYDROGENASE, SACCHAROPINE DEHYDROGENASE, SDH.**
2.329	AT1G04610	**YUC3, YUCCA 3.**
2.301	AT5G22500	**FAR1, FATTY ACID REDUCTASE 1.**
2.292	AT5G44400	**FAD-binding Berberine family protein**.
2.262	AT5G52100	**CHLORORESPIRATION REDUCTION 1, CRR1.**
2.248	AT1G70760	**CHLORORESPIRATORY REDUCTION 23, CRR23, NADH DEHYDROGENASE-LIKE COMPLEX L, NDHL**.
2.229	AT2G04690	**Pyridoxamine 5'-phosphate oxidase family protein**.
2.227	AT2G07751	**NADH:ubiquinone/plastoquinone oxidoreductase, chain 3 protein**.
2.202	AT4G25650	**ACD1-LIKE, PROTOCHLOROPHYLLIDE-DEPENDENT TRANSLOCON COMPONENT, 52 KDA, PTC52, TIC55-IV, TRANSLOCON AT THE INNER ENVELOPE MEMBRANE OF CHLOROPLASTS, 55 KDA - IV**.
2.162	AT5G50160	**ATFRO8, FERRIC REDUCTION OXIDASE 8, FRO8.**
2.158	AT5G65110	**ACX2, ACYL-COA OXIDASE 2, ATACX2.**
2.155	AT3G59050	**ATPAO3, PAO3, POLYAMINE OXIDASE 3.**
2.147	AT5G20960	**AAO1, ALDEHYDE OXIDASE 1, ALDEHYDE OXIDASE ALPHA, AO1, AOALPHA, ARABIDOPSIS THALIANA ALDEHYDE OXIDASE 1, AT-AO1, ATAO, ATAO1.**
2.120	AT3G13682	**LDL2, LSD1-LIKE2.**
2.100	AT1G30760	**FAD-binding Berberine family protein**.
2.084	AT4G01910	**Cysteine/Histidine-rich C1 domain family protein**.
2.083	ATCG01010	**NDHF**.
2.073	AT4G32360	**Pyridine nucleotide-disulphide oxidoreductase family protein**.

2.066	AT5G42180	**PER64, PEROXIDASE 64.**
2.047	AT2G26170	**CYP711A1, CYTOCHROME P450, FAMILY 711, SUBFAMILY A, POLYPEPTIDE 1, MAX1, MORE AXILLARY BRANCHES 1**.
2.037	AT2G41220	**GLU2, GLUTAMATE SYNTHASE 2**.
2.018	AT1G80320	**2-oxoglutarate (2OG) and Fe(II)-dependent oxygenase superfamily protein**.
2.000	AT5G47000	**Peroxidase superfamily protein**.
2.000	AT3G48300	**"CYTOCHROME P450, FAMILY 71, SUBFAMILY A, POLYPEPTIDE 23", CYP71A23.**
		**Cell redox homeostasis**
8.214	AT5G61440	**ACHT5, ATYPICAL CYS HIS RICH THIOREDOXIN 5**.
3.582	AT1G08570	**ACHT4, ATYPICAL CYS HIS RICH THIOREDOXIN 4**.
3.288	AT1G53300	**TETRATRICOPEPTIDE-REPEAT THIOREDOXIN-LIKE 1, TTL1**.
2.443	AT4G04610	**APR, APR1, APS REDUCTASE 1, ATAPR1, PAPS REDUCTASE HOMOLOG 19, PRH19.**
2.275	AT4G33040	**Thioredoxin superfamily protein**.
2.256	AT1G34780	**APR-LIKE 4, APRL4, ATAPRL4.**
		**Peroxidase**
3.705	AT4G16270	**Peroxidase superfamily protein.**
3.422	AT1G14540	**PER4, PEROXIDASE 4**.
3.407	AT5G14130	**Peroxidase superfamily protein.**
3.061	AT2G37130	**Peroxidase superfamily protein**.
2.680	AT4G30170	**Peroxidase family protein**.
2.365	AT2G22420	**Peroxidase superfamily protein.**
2.066	AT5G42180	**PER64, PEROXIDASE 64.**
2.000	AT5G47000	**Peroxidase superfamily protein**.
