# Supplementary material for: Transcriptomic profiling of linolenic acid-responsive genes in ROS signaling from RNA-seq data in Arabidopsis
Source: Front Plant Sci. 2015 Mar 17;6:122. doi: 10.3389/fpls.2015.00122 (PMC4362301; doi:10.3389/fpls.2015.00122)
Supplement: Supplemental Table 1 — Fatty acid composition of Arabidopsis thaliana cell suspension cultures (ACSC). [file DataSheet1.ZIP › Table 7.PDF]

Supplementary table 7. Oligonucleotides used for qRT-PCR.

Gene ID	Gene symbol	Forward 5’-> 3’	Reverse 5’-> 3’
**AT3G46520**	ATACT12	ATATCAGTTTTTCACCCTGAAGCCATTTGC	TGACTGATCTGTTTTTTTAAGTTGCTTTGTTGC
**AT2G44065**	ATL2	ACAAATCAGCTAGTGTCAAGAAGAAGAAGAAGC	AAAAACTCTCAAAAAACAGATTCAATAACGTAACC
**Locus X16077**	ARNr 18S	TTTGATGGTACCTGCTACTCGGATAACC	CTCTCCGGAATCGAACCCTAATTCTCC
**AT4G34410**	RRTF1	CAGCTATTCGACAAACAATGGCATCTCC	GGTAACAGTTGATAACCCTTTTATTTCGCTTCC
**AT4G21830**	MSRB7	GTCTCTCTCAAATTCTCTTCTGCTGGTTCC	TACATGAGTATGGGAAAGAAACCTAGCAAGC
**AT1G19180**	JAZ1	GAAGTGATCAACTTGGCGAGCAAAGG	TGGCTCTTGTGTTGTGGTTTTTCTTGG
**AT4G35180**	LH7	CATACAATTTGTGGCTCGACGTAAATAACG	AAGGAGAACCACCACTTTCTTGTGATTCC
**AT2G47180**	GOLS1	CAGGAGTTAAAATTGTCGGTGGATCAGG	AACGTCCAAAGCATTGGAACCAGAGAC
**AT1G72520**	LOX4	AGTATATAATTAGATTTTGACGCAGTATGGTTTATTTGC	TTATTTAAAGCCTTGATTTTTAATTATTTGTGTCG
**AT5G62020**	HSFB2A	GGTTGAGACATTATAATCGAACCAATCAGAGAG	GCTTGTCTTAAAAAGCTGTAATCGTCTACTTCG
**AT2G23250**	GSTU3	CAAGAATTTGAAAGAAATTGAGATCGTGAGG	ACATCACTGGATCAAAGAGTATCATGTTGTCTC
**AT3G45060**	NRT2.6	GGATCTCCGGCTCATGTCTAGTGAAGG	CATAAGAGGAACATAAAACATGCAAACATAAAGC
**AT3G44990**	XTH31	TCCGAAAAGAGACCATACCCAAACACC	ACGCTCAACTGCTTCAATATAGTTTCCAGTG
**AT4G23700**	CHX17	CGGAATTTCGAAGAACGTCACGACTG	TGTTATTATACAAGCCAATCTTTTGACCTTTCG
**AT5G46050**	PTR3	CACAAAAGATGGAACAGTTGATCTTCAAGG	CGTAATAAGCCATCCGTTCAAACACCTC
**AT1G69530**	EXPA1	GTTTTATGTTATACTGATGCAGAGGTGGTATTGAG	TAATAAAATCTAAAACTCTCCCTCTCGCTTCG
**AT2G27250**	CLV3	CAGCTCCCTTGACCTAATCTCTTGTTGC	AAGCTTACCAAACGAAACAGATTGCACAC
**AT2G19810**	OZF1	ACACAAACACAAAAAACAAAACCAACTCAGAC	GAAAGTGGCCATGGAGGAATATGAACC
**AT3G12980**	HAC5	TGTGATTTTGATTCAATTTTTGGTGATTCTG	TTTGCCAAAAATACCACATATACAATACGAAGG
