# Supplementary material for: Transcriptomic profiling of linolenic acid-responsive genes in ROS signaling from RNA-seq data in Arabidopsis
Source: Front Plant Sci. 2015 Mar 17;6:122. doi: 10.3389/fpls.2015.00122 (PMC4362301; doi:10.3389/fpls.2015.00122)
Supplement: Supplemental Table 1 — Fatty acid composition of Arabidopsis thaliana cell suspension cultures (ACSC). [file DataSheet1.ZIP › Table 8.PDF]

ID	Name	Fold change
AT4G34410	RRTF1	496.963
AT1G30135	JAZ8	368.105
AT5G43650	BHLH92	242.104
AT1G28480	GRX480	154.372
AT2G44840	ERF13	119.395
AT4G36950	MAPKKK21	105.570
AT4G21830	MSRB7	92.539
AT4G14560	IAA1	91.347
AT3G62100	IAA30	54.658
AT3G25770	AOC2	49.263
AT1G19180	JAZ1	47.792
AT2G21235	Basic-leucine z	47.390
AT2G23170	GH3.3	45.940
AT3G03660	WOX11	45.254
AT1G43160	RAP2.6	44.344
AT2G23060	acyl-CoA N-acy	44.184
AT5G47370	HAT2	41.076
AT4G35180	LHT7	38.872
AT5G13220	JAZ10	36.838
AT5G05600	oxidoreductas	36.680
AT1G74930	ORA47	32.561
AT4G17490	ERF6	30.219
AT1G12980	ESR1	29.928
AT5G19110	Eukaryotic asp	29.888
AT2G26530	AR781	27.503
AT1G80840	WRKY40	26.669
AT1G72450	JAZ6	25.665
AT1G17380	JAZ5	24.812
AT2G47180	GolS1	24.725
AT1G44350	ILL6	24.266
AT1G72520	lipoxygenase 4	22.834
AT4G17500	ERF-1	22.102
AT4G15248	B-box type zin	19.920
AT5G62020	HSFB2A	19.423
AT1G64195	defensin-like p	19.299
AT2G38240	2-oxoglutarate	18.400
AT5G24320	transducin	18.164
AT4G37010	CEN2	17.048
AT5G54490	PBP1	16.292
AT1G32910	HXXXD-type ac	16.163
AT5G08790	ATAF2	16.058
AT5G06900	CYP93D1	15.903
AT2G32140	transmembran	15.292
AT3G44260	putative CCR4	15.027
AT1G52560	HSP20-like cha	14.799
AT1G70130	concanavalin A	14.628
AT1G06620	1-aminocyclop	14.502
AT4G12400	putative stress	14.151
AT3G25780	AOC3	13.449

AT4G37010	CEN2	13.266
AT5G13220	JAZ10	12.956
AT4G32800	ethylene-resp	12.639
AT2G29480	GSTU2	12.549
AT1G32640	MYC2	12.267
AT5G05410	DREB2A	12.125
AT4G25810	XTR6	11.876
AT1G28370	ERF11	11.762
AT3G23030	IAA2	11.626
AT2G26150	HSFA2	11.405
AT1G17420	LOX3	11.382
AT3G04730	IAA16	10.729
AT3G11930	adenine nucle	10.405
AT1G21550	putative calciu	10.143
AT5G42650	AOS	9.711
AT1G70700	TIFY7	9.619
AT4G25200	HSP23.6-MITC	9.597
AT5G58680	armadillo	9.373
AT3G23250	MYB15	9.223
AT5G43380	TOPP6	8.838
AT1G70390	putative F-box	8.818
AT1G74950	TIFY10B	8.813
AT4G33560	Wound-respor	8.725
AT4G19230	CYP707A1	8.666
AT5G47230	ERF5	8.662
AT3G15210	ERF4	8.635
AT2G24762	GDU4	8.384
AT1G22600	Late embryoge	8.170
AT5G24320	transducin	8.148
AT3G49580	LSU1	8.080
AT5G20230	BCB	8.056
AT5G63790	NAC102	7.975
AT5G63160	BT1	7.912
AT1G66090	TIR-NBS class (	7.910
AT1G79410	05-oct	7.902
AT1G32770	NAC012	7.878
AT5G04340	ZAT6	7.847
AT5G52050	mate efflux dc	7.597
AT3G25710	BHLH32	7.532
AT2G33310	IAA13	7.525
AT2G20350	ethylene-resp	7.483
AT1G66400	CML23	7.481
AT5G13200	GEM-like prot	7.433
AT2G44578	RING	7.390
AT2G29470	GSTU3	7.348
AT5G59820	RHL41	7.230
AT5G28237	tryptophan sy	7.223
AT4G13395	RTFL12	7.202
AT1G71520	ethylene-resp	7.101
AT5G65690	PCK2	7.087

AT5G22630	ADT5	7.075
AT5G05365	metal ion binc	7.011
AT5G62100	BAG2	6.928
AT2G10535	LCR29	6.859
AT2G33310	IAA13	6.781
AT2G27080	late embryoge	6.766
AT5G06890	ubiquitin-like f	6.630
AT1G43800	stearoyl-acyl-c	6.509
AT1G05800	DGL	6.480
AT3G46080	C2H2-type zin	6.474
AT1G50750	Plant mobile c	6.457
AT4G15100	scpl30	6.444
AT1G74890	ARR15	6.422
AT4G29670	ACHT2	6.363
AT3G14370	WAG2	6.351
AT5G57560	TCH4	6.299
AT1G64950	CYP89A5	6.294
AT1G54020	GDSL esterase	6.172
AT1G35140	PHI-1	6.169
AT1G22110	structural con:	6.109
AT2G22880	VQ motif-cont	5.990
AT3G06370	NHX4	5.934
AT1G73500	MKK9	5.890
AT5G24770	VSP2	5.880
AT2G27690	CYP94C1	5.846
AT5G47220	ERF2	5.839
AT4G34760	SAUR-like auxi	5.800
AT1G12580	PEPKR1	5.788
AT1G27730	STZ	5.775
AT1G66160	CMPG1	5.648
AT5G48850	ATSDI1	5.618
AT2G06050	OPR3	5.603
AT1G67856	RING	5.571
AT1G60960	IRT3	5.549
AT1G79680	WAKL10	5.421
AT3G06490	MYB108	5.404
AT1G74430	MYB95	5.390
AT2G30400	OFP2	5.386
AT1G68620	alpha	5.349
AT5G37770	TCH2	5.347
AT3G10525	LGO	5.305
AT5G06750	putative prote	5.294
AT1G18300	NUDT4	5.282
AT2G29420	GSTU7	5.160
AT1G57560	MYB50	5.152
AT3G28210	PMZ	5.119
AT2G45680	transcription f	5.103
AT2G37430	C2H2 and C2H	5.092
AT1G01720	ATAF1	5.084
AT2G47270	transcription f	5.081

AT5G03380	heavy metal tı	5.079
AT2G35930	PUB23	5.076
AT5G23280	transcription f	5.068
AT4G12410	SAUR-like auxi	5.023
AT4G23280	CRK20	5.000
AT1G58170	Disease resista	4.982
AT2G46240	BAG6	4.975
AT1G71450	ethylene-resp	4.970
AT3G18690	MKS1	4.951
AT2G29490	GSTU1	4.914
AT5G16980	2-alkenal redu	4.901
AT3G51680	Rossmann-fol	4.879
AT2G20560	DNAJ heat sho	4.870
AT1G55330	AGP21	4.863
AT1G33760	ethylene-resp	4.861
AT5G43380	TOPP6	4.841
AT3G15540	IAA19	4.810
AT3G14440	NCED3	4.755
AT3G50060	MYB77	4.738
AT3G15530	methyltransfe	4.731
AT2G18060	VND1	4.726
AT1G20510	OPCL1	4.724
AT3G19300	protein kinase	4.721
AT4G09030	AGP10	4.704
AT1G49450	WD40 domain	4.702
AT2G44070	translation ini	4.681
AT5G63450	CYP94B1	4.680
AT3G50400	GDSL esterase	4.677
AT5G47980	BAHD acyltran	4.636
AT2G05940	kinase-like pro	4.624
AT5G55250	IAMT1	4.600
AT2G32150	haloacid deha	4.576
AT2G13820	Non-specific li	4.556
AT4G14680	APS3	4.507
AT1G72430	SAUR-like auxi	4.488
AT3G23635	RTFL13	4.447
AT3G48450	RPM1-interact	4.421
AT3G55700	UDP-glycosyltı	4.418
AT1G76650	CML38	4.417
AT2G26380	Leucine-rich re	4.401
AT4G33070	pyruvate deca	4.397
AT3G25250	AGC2-1	4.396
AT2G21050	LAX2	4.387
AT1G24130	WD40 domain	4.376
AT5G38280	PR5K	4.329
AT3G13520	AGP12	4.265
AT1G78410	VQ motif-cont	4.250
AT2G41410	putative calciu	4.225
AT5G56030	HSP81-2	4.189
AT1G09950	RAS1	4.171

AT2G02680	cysteine	4.162
AT3G21270	DOF2	4.156
AT1G72800	RNA-binding	4.133
AT2G25210	60S ribosomal	4.121
AT1G73590	PIN1	4.110
AT2G32120	HSP70T-2	4.106
AT5G01380	transcription f	4.098
AT3G20830	AGC	4.090
AT3G19580	ZF2	4.074
AT5G43040	cysteine	4.051
AT1G74310	HSP101	4.035
AT5G42200	E3 ubiquitin-p	4.034
AT2G47520	HRE2	4.026
AT5G16200	50S ribosomal	4.026
AT4G17660	protein kinase	3.976
AT3G16510	Calcium-depe	3.938
AT1G53060	legume lectin-	3.926
AT3G52400	SYP122	3.925
AT1G32960	SBT3.3	3.911
AT3G62720	XT1	3.908
AT3G23240	ERF1	3.906
AT1G03050	putative clath	3.888
AT1G04550	IAA12	3.870
AT1G72140	putative pepti	3.868
AT3G09940	MDHAR	3.846
AT5G49480	CP1	3.813
AT4G33905	Peroxisomal n	3.813
AT4G24570	dic-02	3.797
AT1G21910	ethylene-resp	3.784
AT2G29450	GSTU5	3.783
AT4G23680	SRPBCC ligand	3.771
AT1G09350	GolS3	3.764
AT4G16140	proline-rich fa	3.750
AT3G07000	cysteine	3.748
AT1G77640	ethylene-resp	3.734
AT4G30450	glycine-rich pr	3.716
AT1G63840	RING	3.712
AT4G31000	Calmodulin-bi	3.699
AT4G37260	MYB73	3.680
AT1G63090	PP2-A11	3.668
AT3G46090	ZAT7	3.667
AT5G07000	ST2B	3.662
AT1G62300	WRKY6	3.655
AT1G02450	NIMIN1	3.637
AT5G25190	ethylene-resp	3.629
AT1G15520	PDR12	3.628
AT4G31985	60S ribosomal	3.628
AT2G35980	YLS9	3.622
AT5G35735	putative auxin	3.615
AT1G04570	integral memb	3.586

AT3G60490	ethylene-resp	3.561
AT5G03590	GDSL esterase	3.544
AT1G74100	SOT16	3.541
AT1G61890	MATE efflux fa	3.536
AT1G04100	IAA10	3.534
AT1G50590	Pirin-like prot	3.527
AT2G16720	MYB7	3.526
AT4G12400	putative stress	3.508
AT3G50260	CEJ1	3.501
AT5G47990	CYP705A5	3.493
AT5G62520	SRO5	3.492
AT3G61640	AGP20	3.469
AT2G43290	MSS3	3.465
AT5G38540	jacalin lectin fa	3.464
AT5G16453	defensin-like p	3.464
AT5G67300	MYBR1	3.459
AT1G78100	F-box protein	3.429
AT3G17611	RBL14	3.428
AT5G42380	CML37	3.426
AT3G28910	MYB30	3.425
AT3G51450	strictosidine sy	3.420
AT3G04640	glycine-rich pr	3.419
AT1G64065	late embryoge	3.410
AT3G45960	EXLA3	3.391
AT4G09350	chaperone Dn	3.391
AT5G38200	class I glutami	3.389
AT3G27500	cysteine	3.375
AT4G39030	EDS5	3.373
AT4G31730	GDU1	3.365
AT4G02320	pectinesterase	3.350
AT2G01420	PIN4	3.322
AT1G30460	CPSF30	3.300
AT1G52890	NAC019	3.297
AT3G15500	NAC3	3.264
AT1G14200	RING	3.260
AT3G22510	Pre-rRNA-proc	3.253
AT5G12880	proline-rich fa	3.248
AT2G32510	MAPKKK17	3.234
AT2G46600	calcium-bindir	3.232
AT2G28500	LBD11	3.214
AT4G39670	glycolipid tran	3.193
AT5G19100	Eukaryotic asp	3.191
AT1G32350	AOX1D	3.182
AT4G17770	TPS5	3.170
AT3G12587	Oligosaccarylt	3.160
AT3G28150	TBL22	3.152
AT2G29040	exostosin-like	3.149
AT5G65640	bHLH093	3.135
AT2G33580	protein kinase	3.134
AT3G09350	Fes1A	3.129

AT2G44080	ARL	3.098
AT3G14200	chaperone Dn	3.093
AT1G11670	MATE efflux fa	3.093
AT5G53750	CBS domain-co	3.091
AT2G32270	ZIP3	3.080
AT4G12810	putative F-box	3.073
AT1G28360	ERF12	3.049
AT2G29460	GSTU4	3.035
AT3G52420	OEP7	3.021
AT5G09930	GCN2	3.021
AT5G18150	Methyltransfe	3.018
AT5G11740	AGP15	3.015
AT5G37670	heat shock pro	3.012
AT5G62160	ZIP12	3.012
AT3G06985	LCR44	3.000
AT1G69120	AP1	3.000
AT4G17215	Pollen Ole e 1	3.000
AT2G27630	Ubiquitin carb	3.000
AT1G06120	delta-9 desatu	3.000
AT2G46590	DAG2	2.975
AT1G61340	F-box protein	2.975
AT4G08260	putative prote	2.965
AT4G17800	putative AT-ho	2.947
AT2G20490	NOP10	2.943
AT3G22800	leucine-rich re	2.934
AT5G54940	Translation ini	2.918
AT1G75080	BZR1	2.915
AT5G05150	G18E	2.912
AT1G24140	matrix metallo	2.905
AT2G47890	zinc finger pro	2.880
AT4G33865	40S ribosomal	2.878
AT4G15393	CYP702A5	2.877
AT3G16050	PDX1.2	2.873
AT1G12500	Nucleotide-su	2.868
AT4G25390	protein kinase	2.867
AT1G55920	SERAT2	2.861
AT4G34990	MYB32	2.848
AT3G20750	GATA29	2.846
AT1G54000	GDSL esterase	2.843
AT4G00140	EDA34	2.835
AT2G14960	GH3.1	2.828
AT4G21990	APR3	2.827
AT5G23530	CXE18	2.825
AT2G38750	ANNAT4	2.817
AT1G01920	SET domain-co	2.817
AT3G52200	LTA3	2.804
AT5G49300	GATA16	2.792
AT5G03610	GDSL esterase	2.791
AT5G09240	ssDNA-binding	2.778
AT5G66070	NEP1-interact	2.772

AT5G18690	AGP25	2.767
AT3G60440	phosphoglycer	2.764
AT5G33406	hAT family din	2.755
AT4G34150	calcium-deper	2.747
AT2G30040	MAPKKK14	2.739
AT3G61220		2.728
AT3G26470	RPW8 domain	2.721
AT3G06680	60S ribosomal	2.706
AT2G47440	tetratricopept	2.703
AT3G28180	CSLC04	2.696
AT1G31880	BRX	2.681
AT1G17870	EGY3	2.674
AT1G18330	EPR1	2.662
AT4G37890	EDA40	2.654
AT5G05370	Cytochrome b	2.653
AT3G23730	XTH16	2.647
AT5G17000	2-alkenal redu	2.646
AT3G60530	GATA4	2.636
AT1G59590	ZCF37	2.636
AT2G28510	Dof zinc finger	2.635
AT4G30280	XTH18	2.627
AT3G06360	AGP27	2.622
AT5G54130	calcium-bindir	2.618
AT5G40730	AGP24	2.610
AT1G67360	Rubber elonga	2.601
AT3G50280	uncharacterize	2.596
AT2G32030	GCN5-related	2.594
AT5G06905	CYP712A2	2.590
AT1G76690	OPR2	2.587
AT1G69780	ATHB13	2.586
AT2G22500	UCP5	2.573
AT2G01150	RHA2B	2.569
AT4G14370	TIR-NBS-LRR c	2.565
AT5G07860	HXXXD-type a	2.564
AT2G05050	protein phosp	2.562
AT4G24160	hydrolase, alp	2.553
AT3G61910	NAC066	2.552
AT5G15350	ENODL17	2.552
AT4G36900	RAP2.10	2.542
AT4G27410	RD26	2.539
AT4G15150	glycine-rich pr	2.526
AT3G20470	GRP5	2.505
AT3G27220	Kelch repeat-c	2.494
AT3G58990	IPMI1	2.489
AT2G47730	GSTF8	2.481
AT2G46510	AIB	2.478
AT1G78230	Outer arm dyr	2.477
AT1G35160	GF14 PHI	2.476
AT5G52020	ethylene-resp	2.470
AT4G33920	putative prote	2.465

AT4G31360	selenium bind	2.463
AT2G13820	Non-specific li	2.456
AT2G45400	BEN1	2.454
AT5G17310	UGP2	2.449
AT5G16970	AER	2.448
AT4G38400	EXLA2	2.443
AT3G05200	ATL6	2.443
AT4G24340	phosphorylase	2.434
AT1G78090	TPPB	2.431
AT5G25930	Protein kinase	2.430
AT5G25170	PPPDE putativ	2.427
AT5G01900	WRKY62	2.422
AT5G47960	RABA4C	2.417
AT3G55980	SZF1	2.415
AT5G50370	adenylate kina	2.415
AT1G17170	GSTU24	2.413
AT3G52870	IQ calmodulin-	2.412
AT3G23167	LCR39	2.407
AT1G27695	glycine-rich pr	2.404
AT5G20190	tetratricopept	2.389
AT2G30020	putative prote	2.386
AT5G64870	Flotillin-like pr	2.382
AT1G05300	ZIP5	2.380
AT3G05890	RCI2B	2.372
AT1G07870	putative serin	2.370
AT5G07870	HXXXD-type a	2.366
AT5G64310	AGP1	2.357
AT3G06990	cysteine	2.354
AT3G44190	FAD	2.351
AT4G30460	glycine-rich pr	2.347
AT2G44830	protein kinase	2.345
AT5G54380	THE1	2.342
AT4G31500	CYP83B1	2.335
AT4G38620	MYB4	2.316
AT1G66180	aspartyl prote	2.315
AT1G09070	SRC2	2.303
AT1G75270	DHAR2	2.296
AT5G60490	FLA12	2.295
AT1G21980	PIP5K1	2.292
AT2G05510	glycine-rich pr	2.291
AT1G72470	EXO70D1	2.289
AT5G52750	heavy metal tı	2.282
AT4G01895	systemic acqu	2.281
AT4G36160	NAC076	2.281
AT1G62560	FMO GS-OX3	2.281
AT1G18350	MKK7	2.278
AT4G30470	Rossmann-fol	2.275
AT3G17390	MTO3	2.268
AT1G18590	SOT17	2.268
AT3G11840	PUB24	2.266

AT3G13310	chaperone Dn	2.264
AT3G05490	RALFL22	2.263
AT3G52800	zinc finger A2(	2.249
AT2G38170	CAX1	2.243
AT1G35910	haloacid deha	2.235
AT2G29440	GSTU6	2.235
AT4G21320	HSA32	2.233
AT1G73325	Kunitz family t	2.230
AT3G15370	EXPA12	2.227
AT2G38170	CAX1	2.226
AT5G48010	THAS1	2.223
AT2G43530	defensin-like p	2.215
AT4G11280	ACS6	2.213
AT5G59550	C3H4 type zin	2.213
AT4G21850	MSRB9	2.212
AT5G37260	RVE2	2.212
AT5G60850	OBP4	2.207
AT5G25450	ubiquinol-cytc	2.200
AT3G46900	COPT2	2.197
AT4G30440	GAE1	2.196
AT5G42050	DCD	2.191
AT1G31540	TIR-NBS-LRR c	2.186
AT4G21990	APR3	2.185
AT1G49410	TOM6	2.178
AT1G02880	TPK1	2.177
AT5G43140	Peroxisomal m	2.177
AT2G41660	MIZ1	2.168
AT1G11740	ankyrin repeat	2.168
AT3G16860	COBL8	2.162
AT5G27910	NF-YC8	2.162
AT1G27100	Actin cross-lin	2.152
AT3G51670	patellin-6	2.152
AT4G30290	XTH19	2.144
AT2G25790	Leucine-rich re	2.140
AT4G33930	glycine-rich pr	2.139
AT3G44610	protein kinase	2.133
AT1G69580	transcription f	2.129
AT1G50740	Transmembra	2.126
AT5G65683	C3H4 type zin	2.123
AT5G24655	LSU4	2.120
AT3G28200	peroxidase 31	2.117
AT5G10430	AGP4	2.115
AT5G24330	ATXR6	2.114
AT3G48970	heavy metal tı	2.109
AT1G02810	pectinesterase	2.102
AT1G03870	FLA9	2.091
AT1G16030	Hsp70b	2.089
AT5G25160	ZFP3	2.087
AT3G51870	putative envel	2.085
AT3G45970	EXLA1	2.085

AT1G57990	PUP18	2.084
AT5G67180	TOE3	2.080
AT1G75370	sec.4-like pho:	2.078
AT1G79310	MC7	2.075
AT5G06710	HAT14	2.063
AT2G44300	xylogen-like pı	2.059
AT3G22830	HSFA6B	2.059
AT1G44830	ethylene-resp	2.057
AT5G02170	Transmembra	2.057
AT4G32440	tudor-like RN/	2.057
AT4G14040	SBP2	2.055
AT4G05220	late embryoge	2.053
AT3G54200	late embryoge	2.049
AT5G22570	WRKY38	2.044
AT1G05100	MAPKKK18	2.042
AT1G63140	O-methyltrans	2.042
AT2G38090	myb	2.036
AT1G80820	CCR2	2.034
AT4G25790	allergen V5	2.028
AT3G45230	hydroxyprolin	2.027
AT5G25180	CYP71B14	2.019
AT3G11700	FLA18	2.019
AT1G49520	SWIB complex	2.012
AT5G61890	ethylene-resp	2.010
AT5G15950	S-adenosylme	2.008
AT1G79830	GC5	2.005
AT1G18520	TET11	2.000
AT2G36640	ECP63	2.000
AT4G25010	nodulin MtN3	2.000
AT2G47560	RING-H2 finge	2.000
AT4G01890	glycoside hydr	2.000
AT3G23550	mate efflux dc	2.000
AT5G23360	GEM-like prot	2.000
AT3G48700	CXE13	2.000
