# Supplementary material for: Transcriptomic profiling of linolenic acid-responsive genes in ROS signaling from RNA-seq data in Arabidopsis
Source: Front Plant Sci. 2015 Mar 17;6:122. doi: 10.3389/fpls.2015.00122 (PMC4362301; doi:10.3389/fpls.2015.00122)
Supplement: Supplemental Table 1 — Fatty acid composition of Arabidopsis thaliana cell suspension cultures (ACSC). [file DataSheet1.ZIP › Table 9.PDF]

ID	Name	Fold change
AT2G20880	ethylene-responsive trans	173.307
AT2G07785	nad1	28.366
AT1G58340	ZF14	25.749
AT1G03840	MGP	24.968
AT1G64380	ethylene-responsive trans	24.112
AT3G45060	NRT2.6	22.577
AT3G61900	SAUR-like auxin-responsiv	20.436
AT1G60280	NAC023	18.670
AT4G39780	ethylene-responsive trans	16.585
AT5G18300	NAC088	16.244
ATMG00180	ccb452	15.799
AT3G24420	hydrolase, alpha	15.797
AT2G40200	transcription factor bHLH5	15.619
ATMG01275	nad1	15.550
AT4G28140	ethylene-responsive trans	15.507
AT3G44990	XTR8	15.048
ATMG00110	ccb206	14.793
AT2G37140	terpene synthase	14.310
AT4G27460	cystathionine beta-syntha	14.234
AT2G07689	NADH-Ubiquinone	13.819
AT4G39070	B-box type zinc finger-con	13.357
AT4G23700	CHX17	13.203
AT5G11320	YUC4	12.652
AT3G30530	bZIP42	12.077
ATMG01275	nad1	12.024
AT2G02750	pentatricopeptide repeat-	11.851
AT1G65910	NAC028	11.654
AT4G18340	glycosyl hydrolase family 1	11.509
AT2G19340	Oligosaccharyltransferase	11.429
AT5G47450	TIP2	11.405
AT1G69440	ago-07	11.140
AT5G46050	PTR3	11.125
AT5G19650	OFP8	10.995
AT2G07734	ribosomal protein S4	10.868
ATMG00290	rps4	10.868
AT2G22330	CYP79B3	10.681
AT5G59990	CCT motif family protein	10.624
AT1G62320	ERD	10.325
AT5G08130	BIM1	10.313
AT1G69530	EXPA1	10.191
AT1G08500	ENODL18	10.073
AT3G24230	putative pectate lyase 9	10.047
AT1G48120	serine	10.037
AT2G27250	CLV3	9.899
AT2G46660	CYP78A6	9.791
AT5G01820	SR1	9.590
ATMG00560	rpl2	9.408
AT1G69490	NAP	9.300
AT3G49930	C2H2 and C2HC zinc finger	9.271
AT1G56010	NAC1	9.017

AT5G39090	HXXXD-type acyl-transfera	8.914
ATMG00650	nad4L	8.762
AT5G53110	RING	8.482
AT5G41380	CCT motif family protein	8.406
AT1G69530	EXPA1	8.374
AT2G19810	zinc finger CCCH domain-c	8.358
AT1G77660	putative phosphatidylinos	8.327
AT4G18350	NCED2	8.326
ATMG01275	nad1	8.284
AT5G61440	ACHT5	8.214
AT5G44210	ERF9	8.198
AT4G24050	Rossmann-fold NAD	8.183
AT5G15830	bZIP3	8.139
AT1G16090	WAKL7	8.088
ATMG00285	nad2	7.951
AT5G52830	WRKY27	7.798
AT1G56220	dormancy	7.796
ATMG00210	rpl5	7.741
AT2G07725	60S ribosomal protein L5	7.741
AT2G36270	ABI5	7.690
AT2G40180	PP2C5	7.650
AT1G62720	pentatricopeptide repeat-	7.628
AT5G49665	C3H4 type zinc finger prot	7.612
AT3G10590	SANT DNA-binding domair	7.576
AT5G58787	RING	7.557
AT5G24490	putative 30S ribosomal pr	7.497
AT2G07715	60S ribosomal protein L2	7.484
AT5G67450	ZF1	7.473
AT4G16790	hydroxyproline-rich glycop	7.429
AT1G54070	Dormancy	7.406
AT1G74550	CYP98A9	7.363
AT2G20990	SYTA	7.280
AT2G27250	CLV3	7.249
ATMG01170	atp6-2	7.117
AT2G25900	ATCTH	7.071
AT1G63180	UGE3	7.057
AT5G14740	CA2	6.989
AT5G67430	GCN5-related N-acetyltrar	6.968
AT1G58390	CC-NBS-LRR class disease r	6.964
AT3G12890	ASML2	6.960
AT1G72830	NF-YA3	6.890
AT5G41070	DRB5	6.852
AT1G79130	SAUR-like auxin-responsiv	6.817
AT3G19890	F-box protein	6.779
AT1G64390	GH9C2	6.722
AT3G56000	CSLA14	6.711
AT1G48100	polygalacturonase	6.684
AT5G59780	MYB59	6.681
AT4G26050	PIRL8	6.598
AT1G21210	WAK4	6.589

AT1G36060	ethylene-responsive trans	6.522
AT2G01830	WOL	6.491
AT1G08650	PPCK1	6.491
AT2G03250	phosphate transporter PH	6.487
AT5G67090	Subtilisin-like serine endo|	6.467
AT5G24080	protein kinase family prot	6.422
AT5G53100	Rossmann-fold NAD	6.402
AT3G07040	rps3	6.399
AT1G07200	P-loop containing nucleosi	6.399
AT2G46430	CNGC3	6.393
AT1G64583	tetratricopeptide repeat-li	6.392
ATMG01275	nad1	6.323
AT1G49320	USPL1	6.291
AT4G12330	CYP706A7	6.191
AT3G24880	Helicase	6.174
AT4G13345	MEE55	6.160
AT2G14870	RNA recognition motif-cor	6.157
ATMG01360	cox1	6.092
AT5G44680	DNA-3-methyladenine gly	6.089
AT2G15890	MEE14	6.049
AT3G14820	GDSL esterase	6.030
AT3G54780	C3HC4-type RING finger pı	6.007
AT3G28007	nodulin MtN3-like protein	5.958
AT5G59070	glycosyl transferase family	5.916
AT1G42560	MLO9	5.903
AT5G60410	SIZ1	5.874
AT1G52580	RBL5	5.842
AT1G80920	J8	5.840
AT1G64590	putative short-chain dehy	5.820
AT4G27950	CRF4	5.791
AT5G59670	Receptor-like protein kina	5.779
AT2G02060	transcription factor	5.748
AT4G28240	putative wound-responsiv	5.697
AT3G17630	CHX19	5.692
AT3G09790	UBQ8	5.671
AT1G52330	late embryogenesis abund	5.621
AT2G40230	HXXXD-type acyl-transfera	5.620
AT1G52315	Regulator of Vps4 activity	5.559
AT3G19190	ATG2	5.554
AT2G44380	cysteine	5.536
AT5G03310	SAUR-like auxin-responsiv	5.477
AT2G28930	PK1B	5.471
AT5G52140	RING	5.465
AT1G68130	IDD14	5.463
AT1G15100	RHA2A	5.447
AT5G14740	CA2	5.432
AT2G36890	RAX2	5.425
AT1G47578	biotin	5.425
AT4G27470	RMA3	5.425
AT1G07570	APK1A	5.400

AT1G75580	SAUR-like auxin-responsiv	5.383
AT3G49790	Carbohydrate-binding pro	5.360
AT5G65550	UDP-glucoronosyl	5.351
AT5G57660	COL5	5.349
AT1G09550	Pectin lyase-like protein	5.349
ATMG01380	rrn5	5.348
AT1G60300	No Apical Meristem doma	5.325
AT5G50940	RNA-binding KH domain-c	5.320
AT3G27650	LBD25	5.312
AT5G14880	Potassium transporter 8	5.281
AT1G09390	GDSL esterase	5.260
AT1G78000	SULTR1	5.256
AT2G47240	LACS1	5.244
AT5G64410	OPT4	5.228
AT3G52770	ZPR3	5.227
AT1G70540	EDA24	5.189
AT2G05910	LURP-one-related 6 protei	5.173
AT3G22400	LOX5	5.144
AT3G27660	OLEO4	5.105
AT1G21920	putative phosphatidylinos	5.097
AT2G40850	PI4K GAMMA 1	5.091
AT5G44190	GLK2	5.083
AT3G16180	major facilitator protein	5.057
AT5G38070	RING	5.052
AT3G30340	nodulin MtN21	5.045
AT3G16520	UGT88A1	5.044
AT5G45800	MEE62	5.022
AT1G13210	ACA.l	5.017
AT3G12980	HAC5	5.010
AT1G30080	glycosyl hydrolases family	4.980
AT2G15890	MEE14	4.946
AT2G07727	cytochrome b	4.935
AT1G72300	Tyrosine-sulfated glycope	4.929
ATMG01275	nad1	4.928
AT1G69530	EXPA1	4.925
AT5G47150	YDG	4.898
AT5G57620	MYB36	4.893
AT5G38340	TIR-NBS-LRR class disease	4.877
AT4G13710	pectate lyase	4.870
AT3G23630	IPT7	4.845
AT3G05470	formin-like protein 11	4.824
AT5G19580	glyoxal oxidase-related pr	4.820
AT1G18270	ketose-bisphosphate aldol	4.812
AT1G52810	2-oxoglutarate-dependent	4.800
AT5G40780	LHT1	4.792
AT2G05160	zinc finger CCCH domain-c	4.790
AT3G12977	no apical meristem-domai	4.786
AT4G36260	STY2	4.769
AT1G49160	WNK7	4.743
AT1G28330	DYL1	4.713

AT4G16620	nodulin MtN21	4.713
AT4G12320	CYP706A6	4.713
AT5G54400	S-adenosyl-L-methionine-(	4.678
AT4G20320	putative CTP synthase	4.644
AT2G44000	late embryogenesis abund	4.636
AT2G34650	PID	4.625
AT2G26650	KT1	4.622
AT3G62320	nucleic acid binding protei	4.621
AT1G52200	PLAC8 family protein	4.618
AT4G37100	catalytic	4.609
AT3G10420	P-loop containing nucleosi	4.606
AT5G41630	putative F-box	4.605
AT5G49180	Putative pectinesterase	4.600
AT1G61290	SYP124	4.598
AT3G59580	RWP-RK domain-containir	4.584
AT5G27510	protein kinase family prot	4.559
AT1G71490	pentatricopeptide repeat-	4.550
AT1G63030	ddf2	4.549
AT2G32480	ARASP	4.530
AT3G49760	bZIP5	4.526
AT1G10010	AAP8	4.521
AT5G60780	NRT2.3	4.501
AT1G35730	PUM9	4.499
AT4G03480	Ankyrin repeat family prot	4.486
AT1G50030	TOR	4.485
AT4G05540	DNA helicase domain-cont	4.484
ATCG00040	matK	4.482
AT1G77800	PHD finger-containing pro	4.481
AT3G23880	F-box	4.467
AT2G28930	PK1B	4.463
ATMG01275	nad1	4.454
AT5G43590	acyl transferase	4.449
AT3G24450	heavy metal transport	4.449
AT1G70430	protein kinase-like protein	4.442
AT2G19150	putative pectinesterase 1(	4.426
AT5G49730	FRO6	4.422
AT1G55350	DEK1	4.413
AT1G02860	NLA	4.387
AT1G13150	CYP86C4	4.380
AT3G54800	Pleckstrin homology	4.378
AT2G22680	C3HC4-type RING finger d	4.373
AT1G67340	zinc finger	4.370
AT1G60140	TPS10	4.369
AT4G35110	phospholipase-like	4.358
AT3G06100	NIP7	4.355
AT1G09540	MYB61	4.332
AT1G79700	AP2-like ethylene-respons	4.322
AT5G28310	oxidoreductase-related pr	4.320
AT1G23000	heavy-metal-associated d	4.319
AT2G39230	LOJ	4.317

AT5G04770	CAT6	4.305
AT3G03240	alpha	4.300
AT1G26260	CIB5	4.295
AT2G40020	Nucleolar histone methylt	4.281
AT3G25950	TRAM, LAG1 and CLN8	4.262
AT2G46370	JAR1	4.255
AT3G17040	HCF107	4.251
AT5G06500	AGL96	4.243
AT1G16440	RSH3	4.235
AT1G56220	dormancy	4.213
AT3G48770	ATP	4.208
AT3G60690	SAUR-like auxin-responsiv	4.207
AT5G39660	CDF2	4.188
AT1G07390	RLP1	4.174
AT4G37310	CYP81H1	4.165
AT3G01500	CA1	4.160
AT5G04210	CCCH-type zinc fingerfami	4.138
AT5G25810	tny	4.127
AT5G10340	F-box protein	4.118
AT2G03090	EXPA15	4.114
AT2G44370	cysteine	4.106
AT5G43610	SUC6	4.106
AT1G66170	MMD1	4.099
AT5G63390	O-fucosyltransferase famil	4.093
AT3G14570	GSL04	4.090
AT2G31070	TCP10	4.090
AT2G46480	GAUT2	4.090
AT4G28010	pentatricopeptide repeat-	4.089
AT1G31350	KUF1	4.089
AT1G25440	zinc finger protein CONST/	4.085
AT5G57710	heat shock protein-like pr(	4.062
AT4G08850	Leucine-rich repeat-contai	4.061
AT1G01180	S-adenosyl-L-methionine-(	4.050
AT5G13400	putative peptide	4.048
AT1G77680	Ribonuclease II	4.039
AT2G43018	CPuORF17	4.025
AT1G32700	PLATZ transcription factor	4.021
AT3G51970	ASAT1	4.010
AT3G02590	Delta	4.000
AT1G29400	ML5	3.991
AT5G46080	protein kinase family prot(	3.984
AT4G13420	HAK5	3.981
AT3G02940	MYB107	3.978
AT2G33770	PHO2	3.976
AT1G67110	CYP735A2	3.972
AT4G05320	UBQ10	3.965
AT1G30330	ARF6	3.961
AT4G16310	LDL3	3.953
AT2G43870	putative polygalacturonas	3.946
AT1G68150	WRKY9	3.941

AT5G62630	HIPL2	3.937
AT3G46590	TRFL1	3.933
AT2G47670	plant invertase	3.930
AT1G06520	GPAT1	3.929
AT4G12440	APT4	3.927
AT3G02170	LNG2	3.924
AT3G54780	C3HC4-type RING finger pr	3.923
AT1G70300	KUP6	3.923
AT4G19960	KUP9	3.918
AT1G66230	MYB20	3.918
AT4G02670	IDD12	3.915
AT5G10170	MIPS3	3.915
AT3G45070	sulfotransferase family pro	3.911
AT3G55630	DFD	3.909
AT5G53850	Enolase-phosphatase E1	3.898
AT4G31320	SAUR-like auxin-responsiv	3.888
AT2G46810	transcription factor bHLH7	3.883
AT1G66720	AtPP-like protein	3.882
AT1G75030	TLP-3	3.868
AT2G18700	TPS11	3.856
AT1G62590	pentatricopeptide repeat-	3.850
AT3G15030	TCP4	3.845
AT5G38040	UDP-glycosyltransferase-li	3.841
AT2G27110	FRS3	3.840
AT4G00210	LBD31	3.833
AT5G63410	Leucine-rich repeat protei	3.826
AT2G36480	ENTH	3.822
AT1G70140	FH8	3.819
AT3G12955	SAUR-like auxin-responsiv	3.818
AT5G04200	MC9	3.816
AT3G05260	glucose and ribitol dehydr	3.808
AT1G28550	RABA1i	3.806
AT1G03080	kinase interacting	3.805
AT1G74360	putative LRR receptor-like	3.799
AT4G08250	scarecrow-like protein 26	3.797
AT1G61500	S-locus lectin protein kinas	3.790
AT5G52310	LTI78	3.790
AT1G15110	phosphatidylserine syntha	3.789
AT1G01360	RCAR1	3.788
AT1G77230	tetratricopeptide repeat-c	3.785
AT2G02220	PSKR1	3.781
AT1G77460	armadillo	3.780
AT5G06820	SRF2	3.780
AT4G15180	SDG2	3.773
AT5G49740	FRO7	3.763
AT4G12780	auxilin-related protein 1	3.762
AT4G01740	cysteine	3.756
ATMG00513	nad5	3.756
AT3G11660	NHL1	3.755
AT1G01580	FRO2	3.752

AT1G54470	RPP27	3.749
AT5G43420	RING-H2 finger protein AT	3.746
AT3G29380	transcription initiation fac	3.740
AT4G03270	CYCD6	3.736
AT2G46070	MPK12	3.734
AT1G63150	pentatricopeptide repeat-	3.734
AT1G71880	SUC1	3.727
AT1G51805	leucine-rich repeat proteir	3.725
AT1G28330	DYL1	3.724
AT3G05640	putative protein phosphat	3.724
AT4G34060	DML3	3.722
AT5G03640	protein kinase family prot	3.722
AT1G03380	ATG18G	3.715
AT1G51790	leucine-rich repeat proteir	3.711
AT4G05320	UBQ10	3.707
AT4G16270	peroxidase 40	3.705
AT5G22240	OFP10	3.697
AT4G35440	CLC-E	3.690
AT4G37320	CYP81D5	3.685
AT5G61950	ubiquitin carboxyl-termina	3.682
AT2G14080	TIR-NBS-LRR class disease	3.674
AT4G11650	OSM34	3.672
AT1G02250	NAC005	3.662
AT1G77530	O-methyltransferase famil	3.662
AT1G29230	CIPK18	3.661
AT1G79000	HAC1	3.658
AT4G31550	WRKY11	3.658
AT2G19990	PR-1-LIKE	3.649
AT5G48110	terpene cyclase, C1 domai	3.619
ATCG00270	psbD	3.617
AT5G56100	glycine-rich protein	3.611
AT1G16710	HAC12	3.601
AT4G35420	DRL1	3.596
AT5G24900	CYP714A2	3.588
AT1G55860	UPL1	3.587
AT5G49360	BXL1	3.583
AT4G30110	HMA2	3.582
AT1G08570	ACHT4	3.582
AT1G29870	tRNA synthetase class II	3.581
AT4G20000	VQ motif-containing prote	3.578
AT4G04970	GSL1	3.577
AT1G30820	CTP synthase-like protein	3.577
AT4G20140	GSO1	3.568
AT4G31550	WRKY11	3.567
AT2G07698	F-type H+-transporting AT	3.567
AT2G40130	heat shock-related proteir	3.565
AT2G43610	putative chitinase	3.557
AT4G38190	CSLD4	3.556
AT3G59030	TT12	3.552
AT2G46020	BRM	3.545

AT5G02400	PLL2	3.544
AT4G30370	RING-H2 finger protein AT	3.544
AT1G56430	NAS4	3.542
AT1G60160	putative potassium transp	3.541
AT5G45428	CPuORF24	3.534
AT4G14140	DMT2	3.533
AT4G09760	protein kinase family prot	3.532
AT1G23450	pentatricopeptide repeat-	3.531
AT5G18670	BMY3	3.527
AT5G38710	proline dehydrogenase 2	3.518
AT1G66920	protein kinase-like protein	3.508
AT1G70290	TPS8	3.506
AT2G37820	cysteine	3.499
AT5G01520	RING	3.498
AT3G61950	transcription factor bHLH6	3.496
AT4G00905	NC domain-containing pro	3.496
AT5G12300	calcium-dependent lipid-b	3.495
AT3G15450	aluminum induced protein	3.488
AT3G14020	NF-YA6	3.486
AT1G29770	haloacid dehalogenase-lik	3.486
AT5G07340	calnexin2	3.486
AT5G25370	PLDALPHA3	3.484
AT4G02075	PIT1	3.484
AT5G43530	Helicase protein with RING	3.483
AT4G24800	MA3 domain-containing p	3.483
AT1G75380	BBD1	3.482
AT1G01120	KCS1	3.477
AT1G61800	GPT2	3.474
AT2G07687	cytochrome c oxidase sub	3.473
ATMG00730	cox3	3.473
AT5G48900	putative pectate lyase 20	3.469
AT1G68795	CLE12	3.468
AT3G26820	Esterase	3.466
AT2G17470	Aluminium activated mala	3.464
AT1G30860	RING	3.458
AT4G18600	WAVE5	3.457
AT1G62910	pentatricopeptide repeat-	3.457
AT2G26950	MYB104	3.455
AT1G72820	Mitochondrial substrate ca	3.449
AT4G38810	EF-hand, calcium binding r	3.448
AT1G02220	NAC003	3.445
AT2G18890	protein kinase-like protein	3.440
AT3G04430	NAC049	3.439
AT5G46871	defensin-like protein 308	3.427
AT1G71250	GDSL esterase	3.426
AT4G28250	EXPB3	3.424
AT3G26160	CYP71B17	3.424
AT1G14540	peroxidase 4	3.422
AT3G63010	GID1B	3.420
AT5G46690	bHLH071	3.412

AT5G50860	protein kinase family prot	3.411
AT3G41979	rRNA	3.411
AT2G01020	5S ribosomal RNA	3.411
AT5G14130	peroxidase 55	3.407
AT1G01340	CNGC10	3.404
AT4G31230	Protein kinase protein wit	3.402
AT3G06690	acyl-CoA dehydrogenase c	3.397
AT1G31650	ROPGEF14	3.397
AT5G44180	Homeodomain-like transc	3.391
AT5G09730	BXL3	3.390
AT1G02080	CCR4-NOT transcription cc	3.389
AT3G27150	F-box	3.386
AT1G12940	NRT2.5	3.385
AT2G34190	nucleobase-ascorbate trar	3.379
AT1G29380	carbohydrate-binding X8 c	3.378
AT5G04230	PAL3	3.377
AT4G35560	transducin	3.377
AT4G03292	RNase H domain-containir	3.376
AT5G66770	scarecrow-like protein 4	3.371
AT1G04300	MATH domain-containing	3.369
AT4G12310	CYP706A5	3.366
AT5G56890	protein kinase family prot	3.366
AT2G28250	NCRK	3.366
AT5G25890	IAA28	3.365
AT1G51640	EXO70G2	3.363
AT5G59780	MYB59	3.354
AT1G30110	NUDX25	3.352
AT3G28100	nodulin MtN21	3.350
AT5G59810	SBT5.4	3.350
AT5G15540	EMB2773	3.344
ATCG00350	psaA	3.344
AT5G52040	ATRSP41	3.343
AT2G15320	leucine-rich repeat-contai	3.342
AT2G40900	nodulin MtN21	3.342
AT1G71010	FAB1C	3.340
AT5G60830	bZIP70	3.329
AT2G18650	MEE16	3.328
AT4G16380	metal ion binding protein	3.327
AT3G27290	F-box protein	3.324
AT3G52840	BGAL2	3.318
AT2G19190	FRK1	3.317
AT1G22360	UGT85A2	3.310
AT5G05320	FAD	3.309
ATCG00050	rps16	3.309
AT2G45430	AHL22	3.309
AT5G11590	TINY2	3.309
AT1G32510	NAC011	3.309
AT2G46690	SAUR-like auxin-responsiv	3.308
AT5G25830	GATA12	3.303
AT2G04240	XERICO	3.299

AT1G27045	homeobox-leucine zipper	3.292
AT1G58230	WD40 and Beach domain-	3.291
AT4G01023	RING	3.291
AT3G10360	PUM4	3.291
AT1G11340	putative S-locus lectin pro	3.288
AT1G53300	TTL1	3.288
AT2G45880	BAM7	3.282
AT5G55930	OPT1	3.282
AT5G27950	kinesin motor protein-like	3.276
AT4G30080	ARF16	3.276
AT1G18050	SWAP	3.276
AT5G15200	40S ribosomal protein S9-[illegible]	3.273
ATCG00065	rps12	3.271
AT2G07675	ribosomal protein S12	3.271
AT5G60410	SIZ1	3.269
AT5G04780	pentatricopeptide repeat-	3.266
AT1G59890	SNL5	3.264
ATCG00890	ndhB	3.264
ATCG01250	ndhB	3.264
AT1G68230	reticulon-like protein B14	3.262
AT2G23340	DEAR3	3.262
AT4G31530	Rossmann-fold NAD	3.257
AT2G33230	YUC7	3.256
AT2G19980	allergen V5	3.256
AT4G03110	RBP-DR1	3.253
AT2G39705	RTFL8	3.253
AT5G67520	APK4	3.249
AT2G27060	Leucine-rich repeat protei	3.245
AT1G54710	ATG18H	3.241
AT1G18860	WRKY61	3.240
AT4G11800	calcineurin-like phosphoes	3.237
AT2G45170	ATG8E	3.234
AT5G11530	EMF1	3.232
AT1G55280	Lipase	3.231
AT1G74190	RLP15	3.230
AT2G03140	alpha	3.227
AT2G35140	DCD	3.223
AT3G59850	polygalacturonase	3.220
AT1G67220	HAC2	3.217
AT5G49430	WD40	3.216
AT1G23540	IGI1	3.213
AT2G25660	emb2410	3.211
AT1G69600	ZFHD1	3.202
AT4G38160	pde191	3.200
AT1G29780	haloacid dehalogenase-lik	3.199
AT5G43430	ETFBETA	3.195
AT3G15120	AAA-type ATPase family p	3.191
AT4G16600	GT8-glycogenin domain-co	3.187
AT3G24110	putative calcium-binding p	3.186
AT3G04070	NAC047	3.183

AT3G15650	phospholipase	3.182
AT5G23150	HUA2	3.178
ATMG00580	nad4	3.178
AT1G04300	MATH domain-containing	3.177
AT5G62150	peptidoglycan-binding Lys	3.176
AT5G39080	HXXXD-type acyl-transfera	3.172
AT4G27450	aluminum induced protein	3.171
AT3G05680	EMB2016	3.162
AT5G61180	Putative endonuclease or	3.162
AT1G18960	myb-like HTH transcription	3.162
AT3G59710	Rossmann-fold NAD	3.162
AT5G37020	ARF8	3.161
AT2G29200	PUM1	3.160
AT3G48340	putative cysteine proteina	3.156
AT1G68080	2-oxoglutarate	3.150
AT5G60710	C3H4 type zinc finger prot	3.144
AT3G59480	fructokinase	3.142
AT2G18760	CHR8	3.140
AT1G56710	pectin lyase-like protein	3.139
AT5G07620	protein kinase family prot	3.136
AT1G52240	ROPGEF11	3.133
AT4G32850	nPAP	3.132
AT2G18750	Calmodulin-binding protei	3.129
AT4G18197	PUP7	3.126
AT1G24320	mannosyl-oligosaccharide	3.124
AT5G03480	RNA-binding	3.123
AT5G51910	transcription factor TCP19	3.119
AT4G08920	CRY1	3.118
AT2G22920	SCPL12	3.117
AT5G03190	CPUORF47	3.116
AT2G29120	GLR2.7	3.115
AT1G50180	NB-ARC domain-containin	3.113
AT5G56270	WRKY2	3.113
AT4G32620	Enhancer of polycomb-like	3.112
AT5G26220	ChaC-like family protein	3.109
AT5G67100	ICU2	3.108
AT5G46460	pentatricopeptide repeat-	3.108
AT1G06710	pentatricopeptide repeat-	3.102
AT3G62900	CW-type zinc-finger protei	3.100
AT1G67530	U-box domain-containing	3.098
AT2G36210	SAUR-like auxin-responsiv	3.097
AT3G53510	ABC transporter G family r	3.097
AT2G01510	pentatricopeptide repeat-	3.096
AT5G20480	EFR	3.095
AT1G14380	IQD28	3.095
AT3G27870	phospholipid-translocating	3.083
AT3G63340	putative protein phosphat	3.081
AT3G22425	IGPD	3.081
AT3G46370	leucine-rich repeat protein	3.080
AT2G05070	LHCB2.2	3.079

AT1G35530	fanconi anemia group M p	3.078
AT3G10320	Glycosyltransferase family	3.077
AT4G10940	RING	3.073
AT2G35000	E3 ubiquitin-protein ligase	3.072
AT4G35270	RWP-RK domain-containir	3.069
AT2G31180	MYB14	3.066
AT1G74370	RING	3.064
ATMG00990	NADH dehydrogenase sub	3.062
AT2G37130	peroxidase	3.061
AT3G53668	CPuORF51	3.061
AT3G49860	ARLA1B	3.058
AT3G43300	ATMIN7	3.058
AT1G53230	TCP3	3.053
ATCG01090	ndhI	3.047
AT2G39220	PLP6	3.046
AT3G55515	RTFL7	3.045
AT3G61430	PIP1A	3.045
AT1G72960	Root hair defective 3 GTP-	3.045
ATMG00520	matR	3.042
AT2G41900	zinc finger CCCH domain-c	3.041
AT4G35380	SEC7-like guanine nucleoti	3.040
AT5G35790	G6PD1	3.039
AT3G26720	alpha-mannosidase	3.039
AT1G67480	F-box	3.036
AT5G19000	BPM1	3.033
AT3G48810	pentatricopeptide repeat-	3.031
AT2G27610	pentatricopeptide repeat-	3.031
AT5G60270	concanavalin A-like lectin	3.030
AT3G14940	PPC3	3.028
AT2G22920	SCPL12	3.027
AT1G58250	SAB	3.026
AT5G14870	CNGC18	3.025
AT1G23870	TPS9	3.025
ATCG00180	rpoC1	3.019
AT4G03400	DFL2	3.018
AT2G26790	pentatricopeptide repeat-	3.017
AT1G77330	aminocyclopropanecarbox	3.017
AT1G50560	CYP705A25	3.013
AT1G77240	putative AMP-binding pro	3.011
AT1G08320	bZIP transcription factor-li	3.006
AT1G71697	CK1	3.004
AT4G01410	late embryogenesis abund	3.003
AT5G11060	KNAT4	3.003
AT1G67310	calmodulin-binding transc	3.000
AT4G23320	CRK24	2.999
AT1G52070	jacalin-like lectin domain-c	2.999
AT4G01160	BTB	2.999
AT2G35770	scpl28	2.999
AT4G23630	BTI1	2.994
AT3G25890	ethylene-responsive trans	2.994

AT3G25060	pentatricopeptide repeat-	2.994
AT2G40150	TBL28	2.993
AT1G10000	Ribonuclease H-like protei	2.992
AT3G66652	fip1 motif-containing prot	2.991
AT3G26125	CYP86C2	2.990
AT2G23300	Leucine-rich repeat protei	2.987
AT1G20720	RAD3-like DNA-binding he	2.985
AT1G74660	MIF1	2.984
AT2G38970	C3HC4-type RING finger-c	2.984
AT4G17220	MAP70-5	2.984
AT5G12380	ANNAT8	2.983
AT2G18670	RING-H2 finger protein AT	2.979
AT3G13730	CYP90D1	2.979
AT4G28270	RMA2	2.978
AT1G77380	AAP3	2.977
ATCG00580	psbE	2.975
AT5G48620	putative disease resistanc	2.973
AT3G46660	UGT76E12	2.973
AT4G28700	AMT1	2.973
AT1G74630	pentatricopeptide repeat-	2.972
AT1G58220	myb family transcription f	2.971
AT5G07980	dentin sialophosphoprotei	2.971
AT1G36732	CPuORF19	2.969
AT2G39210	major facilitator protein	2.968
AT5G66950	catalytic	2.968
AT2G35910	RING-H2 finger protein AT	2.962
AT3G18770	Autophagy-related protein	2.961
AT4G29950	RabGAP	2.958
AT3G47160	RING	2.957
AT1G51140	transcription factor bHLH1	2.953
ATCG00680	psbB	2.949
AT4G28380	leucine-rich repeat-contai	2.947
AT1G18560	BED zinc finger and hAT di	2.947
AT1G32750	HAF01	2.946
AT4G28080	tetratricopeptide repeat d	2.946
AT3G58920	putative F-box	2.945
AT4G03340	Core-2	2.943
AT5G10790	UBP22	2.934
AT4G09770	TRAF-like family protein	2.933
AT1G54460	TPX2	2.933
AT1G62930	pentatricopeptide repeat-	2.933
AT2G43700	concanavalin A-like lectin	2.932
AT3G52250	duplicated SANT DNA-binc	2.931
AT5G56550	OXS3	2.931
AT5G24710	transducin	2.930
AT1G36160	ACC1	2.928
AT2G17690	SDC	2.926
AT1G06490	GSL07	2.925
AT1G56570	pentatricopeptide repeat-	2.925
AT5G02070	wall-associated receptor k	2.923

AT5G15720	GLIP7	2.922
AT5G44080	basic leucine zipper transc	2.922
AT5G43520	cysteine	2.920
AT3G27670	RST1	2.920
AT1G79570	octicosapeptide	2.920
AT1G55325	GCT	2.919
AT3G54280	RGD3	2.919
AT3G12560	TRFL9	2.918
AT5G47560	TDT	2.916
AT1G49210	E3 ubiquitin-protein ligase	2.915
AT5G66640	DAR3	2.913
AT2G43445	F-box	2.912
AT2G45600	alpha	2.912
AT5G66020	ATSAC1B	2.912
AT2G37170	PIP2B	2.912
AT4G00360	CYP86A2	2.912
AT2G33100	CSLD1	2.909
AT2G46880	PAP14	2.906
AT4G33240	FAB1A	2.905
AT3G60720	PDLP8	2.905
AT3G23590	RFR1	2.904
AT1G07590	pentatricopeptide repeat-	2.901
AT5G60210	RIP5	2.899
AT4G30100	tRNA-splicing endonucleas	2.898
AT1G21590	adenine nucleotide alpha l	2.898
AT5G03140	concanavalin A-like lectin l	2.898
AT1G08260	TIL1	2.890
AT3G01460	MBD9	2.888
AT5G22740	CSLA02	2.887
AT2G42280	transcription factor bHLH1	2.884
AT1G71890	SUC5	2.884
AT1G58360	AAP1	2.882
AT1G67820	putative protein phosphat	2.879
AT4G22970	ESP	2.877
AT3G62980	TIR1	2.876
AT1G55970	HAC4	2.872
AT1G79670	RFO1	2.866
AT5G47690	sister chromatid cohesion	2.866
AT4G12010	TIR-NBS-LRR class disease	2.863
AT1G64570	DUO3	2.862
AT2G02150	tetratricopeptide repeat-c	2.862
AT5G65170	VQ motif-containing prote	2.861
AT1G15110	phosphatidylserine syntha	2.859
AT3G22180	putative S-acyltransferase	2.859
AT1G79840	GL2	2.859
AT1G16260	wall-associated receptor k	2.857
AT2G40030	NRPD1B	2.857
AT2G37050	putative receptor protein	2.855
AT4G28040	nodulin MtN21	2.855
AT2G37050	putative receptor protein	2.855

AT3G02240	RGF7	2.854
AT1G74200	RLP16	2.852
AT4G00900	ECA2	2.851
AT5G17500	glycosyl hydrolase family 5	2.851
AT3G57300	INO80	2.848
AT1G15740	leucine-rich repeat-contai	2.846
AT3G10740	ASD1	2.843
AT1G03540	pentatricopeptide repeat-	2.842
AT3G57380	Glycosyltransferase family	2.841
AT1G77620	P-loop containing nucleosi	2.839
AT1G08060	MOM	2.838
AT4G18390	TCP2	2.838
AT5G46270	Disease resistance protein	2.837
AT3G55740	PROT2	2.837
AT4G15430	ERD	2.836
AT3G49210	O-acyltransferase	2.834
AT1G78990	HXXXD-type acyl-transfera	2.831
ATMG00510	nad7	2.830
AT4G33150	lysine-ketoglutarate reduc	2.829
AT2G31020	ORP1A	2.828
AT1G67390	putative F-box protein	2.828
AT1G09170	calponin homology and ki	2.826
AT2G42600	PPC2	2.824
AT4G16480	INT4	2.823
AT4G25672	CPuORF12	2.823
AT5G43440	putative 2-oxoglutarate-d	2.821
AT1G29800	RING	2.821
AT2G25470	RLP21	2.819
AT1G29750	RKF1	2.818
AT1G79640	protein kinase domain-cor	2.816
AT1G06260	cysteine proteinase-like pr	2.814
AT1G08320	bZIP transcription factor-li	2.813
AT4G19860	Lecithine-cholesterol acylt	2.812
AT3G09040	pentatricopeptide repeat-	2.810
AT5G04560	DME	2.809
AT4G18450	ethylene-responsive trans	2.808
AT4G31160	DCAF1	2.807
AT3G54100	O-fucosyltransferase famil	2.807
AT2G37150	RING	2.803
AT1G35230	AGP5	2.803
AT1G04380	2-oxoglutarate	2.802
AT5G35890	Beta-galactosidase relatec	2.801
AT5G17800	MYB56	2.800
AT3G60030	SPL12	2.799
AT4G19220	pentatricopeptide repeat-	2.799
AT4G14990	Topoisomerase II-associat	2.798
AT3G08940	LHCB4.2	2.797
AT5G48560	transcription factor bHLH7	2.795
AT3G45600	TET3	2.794
AT1G28420	HB-1	2.793

AT4G33080	AGC	2.793
AT5G41610	CHX18	2.793
AT5G02880	UPL4	2.792
AT1G58470	RBP1	2.790
AT3G54140	PTR1	2.788
AT1G08070	OTP82	2.787
AT4G26090	RPS2	2.786
AT5G53090	Rossmann-fold NAD	2.786
AT1G07630	PLL5	2.786
AT1G27080	NRT1.6	2.783
AT4G28160	hydroxyproline-rich glycop	2.783
ATCG00360	ycf3	2.783
AT1G26420	FAD-binding and BBE dom	2.783
AT4G35560	transducin	2.782
AT2G33830	dormancy	2.781
AT1G29280	WRKY65	2.781
AT3G19930	STP4	2.779
AT2G32390	GLR3.5	2.779
AT2G26960	MYB81	2.776
AT3G27320	hydrolase	2.775
AT3G17220	PMEI2	2.773
AT4G10960	UGE5	2.773
AT1G77300	EFS	2.771
AT3G17470	CRSH	2.769
AT4G29180	RHS16	2.768
AT3G27970	exonuclease-like protein	2.768
AT5G65790	MYB68	2.767
AT2G46225	ABIL1	2.766
AT3G04420	NAC048	2.765
AT5G08350	GEM-like protein 4	2.765
AT4G14150	PAKRP1	2.763
AT4G33240	FAB1A	2.762
AT1G64170	CHX16	2.762
AT5G42010	transducin	2.760
AT4G18430	RABA1e	2.760
AT4G35520	MLH3	2.759
AT1G05570	CALS1	2.758
AT2G40110	yippee-like protein	2.758
AT5G25630	pentatricopeptide repeat-	2.755
AT1G53310	PPC1	2.755
AT5G59260	concanavalin A-like lectin	2.755
AT2G21540	SFH3	2.754
AT4G40060	HB16	2.753
AT3G57430	OTP84	2.752
AT1G08900	sugar transporter ERD6-lik	2.752
AT5G04895	DEA	2.751
AT5G38895	RING	2.751
AT1G30500	NF-YA7	2.750
AT1G74150	kelch motif-containing prc	2.750
AT4G18940	RNA ligase	2.748

AT5G65740	zinc ion binding protein	2.748
AT3G15930	pentatricopeptide repeat-	2.747
AT2G21300	ATP binding microtubule r	2.746
AT1G18000	transmembrane transport	2.746
AT1G18010	transmembrane transport	2.746
AT5G08520	duplicated SANT DNA-binc	2.744
AT1G28260	Telomerase activating pro	2.744
AT1G52120	jacalin-like lectin domain-c	2.742
AT2G04860	pentatricopeptide repeat-	2.741
AT5G63320	NPX1	2.740
AT1G15290	tetratricopeptide repeat-c	2.739
AT5G64690	neurofilament triplet H pr	2.738
AT2G30933	carbohydrate-binding X8 c	2.738
ATCG01080	ndhG	2.737
AT2G16380	sec.4-like phosphatidylino	2.736
AT1G13350	protein kinase domain-cor	2.735
AT5G43900	MYA2	2.734
AT3G10770	Single-stranded nucleic ac	2.734
AT1G47900	filament-like plant protein	2.733
AT2G19120	tRNA-splicing endonucleas	2.733
AT1G53680	GSTU28	2.733
AT2G03380	pentatricopeptide repeat-	2.732
AT5G49160	MET1	2.729
AT4G24430	Rhamnogalacturonate lyas	2.729
AT5G21160	LA RNA-binding protein	2.728
AT5G56230	PRA1.G2	2.728
AT2G45850	AT hook motif DNA-bindin	2.725
AT4G24020	NLP7	2.725
AT5G04140	GLU1	2.723
AT3G06130	heavy-metal-associated do	2.723
AT1G80650	RTL1	2.721
AT4G37250	putative LRR receptor-like	2.719
AT1G53350	putative disease resistance	2.718
AT3G59140	MRP14	2.718
AT1G62960	ACS10	2.717
AT2G22070	pentatricopeptide repeat-	2.715
AT5G59610	chaperone DnaJ-domain c	2.715
AT5G45650	subtilase family protein	2.714
AT5G15700	DNA-directed RNA polyme	2.713
AT4G14760	kinase interacting-like pro	2.711
AT5G07150	leucine-rich repeat protein	2.710
AT2G47430	CKI1	2.710
AT1G58110	basic helix-loop-helix dom	2.708
AT1G77850	ARF17	2.708
AT1G36180	ACC2	2.706
AT5G16640	pentatricopeptide repeat-	2.706
AT3G09920	PIP5K9	2.705
AT3G57630	exostosin family protein	2.704
AT5G46100	pentatricopeptide repeat-	2.703
AT5G47610	RING-H2 finger protein AT	2.702

AT5G37980	2-alkenal reductase	2.702
ATCG01070	ndhE	2.702
AT3G29270	RING	2.701
AT2G39700	EXPA4	2.700
AT1G08720	EDR1	2.697
AT1G33420	PHD finger protein	2.695
AT3G28550	Proline-rich extensin-like f	2.695
AT3G22380	TIC	2.694
AT3G54950	PLA IIIA	2.694
AT1G13710	CYP78A5	2.693
AT1G76560	CP12-3	2.691
AT2G07719	Putative membrane lipopr	2.690
AT3G61460	BRH1	2.690
AT5G05770	WOX7	2.689
AT1G16150	WAKL4	2.688
AT1G55270	F-box	2.688
AT1G63100	scarecrow-like protein 28	2.687
AT5G41330	BTB	2.687
AT5G28340	pentatricopeptide repeat-	2.686
AT1G56130	putative LRR receptor-like	2.686
AT4G35870	early-responsive to dehyd	2.686
AT4G10695	CDC68-related protein	2.686
AT5G66500	pentatricopeptide repeat-	2.685
AT1G30900	VSR6	2.684
AT3G61150	HDG1	2.683
AT2G03590	UPS1	2.682
AT4G36770	UDP-glycosyltransferase-li	2.681
AT4G24240	WRKY7	2.681
AT4G30170	peroxidase 45	2.680
AT1G24300	GYF domain-containing pr	2.680
AT1G73450	protein kinase domain-cor	2.679
AT2G35680	phosphotyrosine protein p	2.679
AT1G20650	protein kinase-like protein	2.679
AT1G53600	pentatricopeptide repeat-	2.679
AT1G61550	S-locus lectin protein kinas	2.678
AT1G14240	GDA1	2.677
AT1G05700	Leucine-rich repeat transn	2.677
AT5G54280	ATM2	2.674
AT2G37840	unc51-like kinase	2.673
AT1G26330	DNA binding protein	2.673
AT5G64380	fructose-1,6-bisphosphata	2.673
AT5G66850	MAPKKK5	2.673
AT3G45290	MLO3	2.673
AT2G32610	CSLB01	2.673
AT3G21070	NADK1	2.672
AT5G63540	RMI1	2.672
AT3G43800	GSTU27	2.671
AT4G23000	hydrolase	2.670
AT1G18670	IBS1	2.668
AT3G13065	SRF4	2.667

AT5G02010	ROPGEF7	2.666
AT4G24780	putative pectate lyase 18	2.666
AT4G14270	Polyadenylate-binding pro	2.666
AT4G18950	Integrin-linked protein kin	2.665
AT1G34260	FAB1D	2.665
AT4G00910	aluminum activated malat	2.664
AT4G24970	histidine kinase-like ATPas	2.664
AT1G59750	ARF1	2.662
AT4G35130	pentatricopeptide repeat-	2.662
AT1G33260	protein kinase domain-cor	2.658
AT3G63210	MARD1	2.658
AT2G44490	PEN2	2.657
AT1G55290	2-oxoglutarate	2.657
AT1G61360	S-like receptor protein kin	2.656
AT4G17610	tRNA	2.655
AT1G72180	leucine-rich receptor-like p	2.654
AT1G03830	guanylate-binding protein	2.651
AT5G64700	nodulin MtN21	2.651
ATCG00340	psaB	2.651
AT2G40770	RING-finger, DEAD-like hel	2.650
AT1G17920	HDG12	2.650
AT3G13770	pentatricopeptide repeat-	2.650
AT3G47820	PUB39	2.649
AT1G13800	pentatricopeptide repeat-	2.648
AT1G11190	BFN1	2.647
AT4G35800	NRPB1	2.646
AT2G41700	ABCA1	2.645
AT2G41310	RR3	2.642
AT5G65970	MLO10	2.640
AT3G46680	UDP-glycosyltransferase-li	2.640
AT5G52290	SHOC1	2.639
AT2G42980	aspartyl protease-like prot	2.637
AT1G01660	putative U-box domain-co	2.636
AT2G13370	CHR5	2.636
AT1G20080	SYTB	2.635
AT4G25560	MYB18	2.632
AT4G19050	NB-ARC domain-containin	2.632
AT5G42490	ATP binding microtubule r	2.632
AT2G28110	FRA8	2.630
AT3G63070	Tudor	2.630
AT2G21350	RNA-binding CRS1	2.628
AT3G09030	BTB	2.626
AT1G24706	THO2	2.624
AT3G20280	PHD finger protein	2.624
AT3G14270	FAB1B	2.623
AT5G13170	SAG29	2.621
AT1G63500	Protein kinase protein wit	2.621
AT1G68990	MGP3	2.620
AT1G68710	phospholipid-translocating	2.619
AT2G45220	pectinesterase 17	2.619

AT1G23390	Kelch repeat-containing F-	2.619
AT5G39260	EXPA21	2.619
AT1G63130	pentatricopeptide repeat-	2.618
AT5G02310	PRT6	2.617
AT2G45210	SAUR-like auxin-responsiv	2.617
AT1G66050	VIM2	2.615
AT4G36550	armadillo	2.615
AT5G67280	RLK	2.615
AT2G24540	AFR	2.614
AT3G51325	RING	2.613
AT3G12950	trypsin-like protein	2.613
AT1G61210	transducin	2.612
AT5G25350	EBF2	2.612
AT5G42210	major facilitator protein	2.611
AT5G39760	HB23	2.611
AT5G41580	RING	2.609
AT5G13270	RARE1	2.607
AT1G33410	SAR1	2.607
AT1G26930	F-box	2.606
AT1G19700	BEL10	2.606
AT5G47640	NF-YB2	2.605
AT4G01026	PYL7	2.603
AT1G50060	putative pathogenesis-rela	2.603
AT1G63880	TIR-NBS-LRR class disease	2.602
AT1G71930	VND7	2.601
AT1G12300	pentatricopeptide repeat-	2.601
AT1G72250	Di-glucose binding protein	2.600
AT1G22275	ZYP1b	2.599
AT2G01620	MEE11	2.598
AT2G15820	OTP51	2.596
AT2G19130	S-locus lectin protein kina:	2.596
AT1G15125	S-adenosyl-L-methionine-(	2.595
AT4G04955	ALN	2.594
AT1G67140	SWEETIE	2.593
AT5G25900	GA3	2.593
AT2G37478	CPuORF52	2.592
AT2G20050	protein phosphatase 2C ar	2.591
AT5G18320	putative U-box domain-co	2.591
AT5G50330	protein kinase-like protein	2.590
AT1G75370	sec.4-like phosphatidylino	2.589
AT3G06920	pentatricopeptide repeat-	2.589
AT3G59052	CPuORF18	2.588
AT4G38780	Pre-mRNA-processing-spli	2.586
AT5G52910	ATIM	2.586
AT3G11960	Cleavage and polyadenyla	2.586
AT1G01320	tetratricopeptide repeat-c	2.586
AT4G13260	YUC2	2.586
AT4G16950	RPP5	2.585
AT1G55760	BTB	2.584
AT5G06670	kinesin heavy chain-like pr	2.584

AT1G72010	transcription factor TCP22	2.584
AT1G27180	TIR-NBS-LRR class disease	2.583
AT4G29050	concanavalin A-like lectin	2.583
AT2G46680	HB-7	2.583
AT3G13672	seven in absentia	2.582
AT1G28690	pentatricopeptide repeat-	2.581
AT1G04580	AO4	2.580
AT2G01820	leucine-rich repeat protei	2.580
AT1G33770	protein kinase-like protein	2.577
AT5G53150	DNAJ heat shock N-termin	2.577
AT1G65800	RK2	2.576
AT1G05010	EFE	2.576
AT2G47800	MRP4	2.576
AT1G09930	OPT2	2.576
ATCG01100	ndhA	2.576
AT4G09760	protein kinase family prot	2.575
ATCG00730	petD	2.574
AT1G59870	PEN3	2.572
AT3G16320	CDC27a	2.571
AT4G30190	HA2	2.571
AT1G69150	cysteine	2.570
AT2G26890	GRV2	2.569
AT1G30610	EMB2279	2.568
AT2G16485	DNA binding	2.566
AT4G38600	KAK	2.566
AT5G15680	armadillo	2.565
AT2G17820	HK1	2.565
AT3G57680	Peptidase S41 family prote	2.563
AT2G17525	pentatricopeptide repeat-	2.561
AT1G15890	CC-NBS-LRR class disease	2.561
AT3G48360	BT2	2.561
AT5G65210	TGA1	2.559
AT5G05560	EMB2771	2.558
AT2G39260	RNA binding protein	2.558
AT4G32620	Enhancer of polycomb-like	2.557
AT3G49160	pyruvate kinase	2.557
AT5G21482	CKX7	2.557
AT1G58520	RXW8	2.557
AT3G23080	SRPBCC ligand-binding do	2.556
AT3G29190	terpene cyclase, C1 domai	2.555
AT4G15890	condensin-2 complex sub	2.555
AT4G35290	GLUR2	2.555
AT4G37770	ACS8	2.555
AT3G10390	FLD	2.554
AT3G57060	condensin complex subun	2.553
AT5G15340	pentatricopeptide repeat-	2.553
AT5G54250	CNGC4	2.552
AT1G02310	MAN1	2.551
AT2G24740	SDG21	2.549
AT3G19380	PUB25	2.549

AT5G67420	LBD37	2.549
AT5G41160	PUP12	2.548
AT5G42950	GYF domain-containing pr	2.547
AT5G60450	ARF4	2.547
AT3G49142	tetratricopeptide repeat-li	2.547
AT1G30560	putative glycerol-3-phospl	2.547
AT1G15165	RING	2.546
AT2G30010	TBL45	2.546
AT5G55050	GDSL esterase	2.546
AT1G21110	O-methyltransferase famil	2.545
AT1G06140	pentatricopeptide repeat-	2.544
AT2G46570	LAC6	2.542
AT4G37480	chaperone DnaJ-domain-c	2.542
AT1G61210	transducin	2.541
AT1G50770	Aminotransferase-like, pla	2.540
AT1G72950	TIR-NBS class of disease re	2.540
AT4G16470	pentatricopeptide	2.538
AT1G02730	CSLD5	2.537
AT3G26000	F-box protein SKIP14	2.537
AT5G65210	TGA1	2.537
AT4G32700	DNA polymerase theta sul	2.537
AT2G46790	PRR9	2.537
AT4G31620	B3 domain-containing pro	2.536
AT4G14270	Polyadenylate-binding pro	2.535
AT3G05660	RLP33	2.534
AT5G38780	putative S-adenosylmethic	2.534
AT1G68190	putative zinc finger protei	2.533
AT1G54130	RSH3	2.532
AT1G63740	TIR-NBS-LRR class disease	2.530
AT1G08900	sugar transporter ERD6-lik	2.530
AT1G19660	putative wound-responsiv	2.529
AT1G69850	NRT1:2	2.529
AT1G60370	putative F-box protein	2.528
AT1G30320	Remorin family protein	2.528
AT2G15900	phox domain-containing p	2.528
ATCG00720	petB	2.528
AT5G39610	NAC6	2.527
AT1G55580	LAS	2.526
AT4G13030	P-loop containing nucleosi	2.525
AT5G62680	putative peptide	2.522
AT1G53190	RING	2.522
AT5G57210	microtubule-associated pr	2.522
AT2G32470	F-box associated ubiquitin	2.522
AT1G64660	MGL	2.521
AT1G25570	Di-glucose binding protein	2.521
AT2G03810	18S pre-ribosomal assemk	2.520
AT1G14687	HB32	2.520
AT3G21550	DMP2	2.520
AT3G01660	S-adenosylmethionine-de	2.520
AT5G35210	DNA binding and zinc-fing	2.519

AT1G80070	SUS2	2.517
AT5G38990	interleukin-1 receptor-ass	2.517
AT2G17450	RHA3A	2.517
AT5G05940	ROPGEF5	2.516
AT2G25290	octicosapeptide	2.516
AT5G16940	carbon-sulfur lyase	2.515
AT5G22820	ARM repeat-containing pr	2.514
AT4G37560	formamidase	2.512
AT1G21580	zinc finger CCCH domain-c	2.512
AT1G05670	uncharacterized UDP-gluc	2.512
AT1G14240	GDA1	2.511
AT2G28040	aspartyl protease-like prot	2.510
AT4G08210	pentatricopeptide repeat-	2.508
AT5G57250	pentatricopeptide repeat-	2.507
AT2G43880	putative polygalacturonas	2.506
AT1G55540	emb1011	2.504
AT3G22410	Sec14p-like phosphatidylir	2.504
AT5G44530	Subtilase family protein	2.503
AT5G57220	CYP81F2	2.502
AT3G22770	F-box associated ubiquitin	2.502
AT3G61380	Phosphatidylinositol N-ace	2.502
AT3G30460	RING	2.501
AT1G78480	prenyltransferase-like pro	2.501
AT2G43330	INT1	2.501
AT3G63340	putative protein phosphat	2.500
AT5G49370	Pleckstrin homology	2.499
AT4G01925	cysteine	2.497
AT2G22125	cellulose synthase-interac	2.495
AT2G27430	armadillo	2.494
AT5G52300	LTI65	2.493
AT1G72660	P-loop containing nucleosi	2.492
AT5G60930	kinesin family member 4	2.491
AT3G01580	pentatricopeptide repeat-	2.491
AT4G00800	transducin family protein	2.490
AT3G11964	RNA binding protein	2.490
AT1G14330	F-box	2.490
AT5G64260	EXL2	2.489
AT2G31290	ubiquitin carboxyl-termina	2.488
AT4G26350	putative F-box	2.488
AT5G61140	U5 small nuclear ribonucle	2.487
AT5G13230	pentatricopeptide repeat-	2.487
AT1G27430	GYF domain-containing pr	2.487
AT4G03440	ankyrin repeat-containing	2.486
AT1G47960	C	2.485
AT5G18525	protein serine	2.485
AT1G27170	transmembrane receptors	2.485
AT2G20290	XIG	2.485
AT3G53720	CHX20	2.483
AT1G08315	armadillo	2.483
AT3G13080	MRP3	2.482

AT1G27320	HK3	2.482
AT4G31100	wall-associated receptor k	2.481
AT1G07540	TRFL2	2.481
AT3G59550	SYN3	2.480
AT3G08850	RAPTOR1	2.480
AT5G12430	Heat shock protein DnaJ w	2.480
AT5G07820	calmodulin-binding protei	2.479
AT5G24220	lipase class 3-related prot	2.479
AT2G36960	TKI1	2.479
AT1G20960	emb1507	2.478
AT3G16940	calmodulin-binding transc	2.478
AT4G28530	NAC074	2.478
AT2G36960	TKI1	2.476
AT5G24830	pentatricopeptide repeat-	2.475
AT1G17540	Protein kinase protein wit	2.475
AT4G39270	leucine-rich repeat protei	2.474
AT3G17770	Dihydroxyacetone kinase	2.474
AT5G62760	ZAP - like protein	2.473
AT2G18120	SRS4	2.472
AT3G61690	nucleotidyltransferase	2.472
AT2G35740	INT3	2.471
AT2G28080	UDP-glycosyltransferase-li	2.471
AT2G22310	UBP4	2.470
AT2G22690	zinc ion binding protein	2.470
AT4G18610	LSH9	2.470
AT5G49110	fanconi anemia group I pr	2.469
AT3G20200	Protein kinase protein wit	2.467
AT3G06530	U3 small nucleolar RNA-as	2.467
ATMG01390	rrn18	2.466
AT5G12840	NF-YA1	2.465
AT5G54250	CNGC4	2.465
AT5G21222	SNF1-like protein kinase	2.464
AT5G07740	actin binding protein	2.464
AT2G34620	transcription termination	2.464
AT4G09430	TIR-NBS-LRR class disease	2.462
AT4G02110	transcription coactivator p	2.461
AT4G20770	pentatricopeptide repeat-	2.461
AT2G47900	TLP3	2.461
AT1G30010	Intron maturase, type II-lik	2.460
AT2G44710	RNA recognition motif-cor	2.460
AT2G19110	HMA4	2.460
AT1G42550	PMI1	2.460
AT5G49800	lipid-binding START domai	2.460
AT3G47910	Ubiquitin carboxyl-termina	2.459
AT3G60220	ATL4	2.459
AT4G22760	pentatricopeptide repeat-	2.458
AT1G20440	COR47	2.458
AT1G64470	ubiquitin-like protein	2.458
AT1G80520	Sterile alpha motif	2.458
AT1G27040	putative peptide	2.457

AT4G29380	phosphoinositide-3-kinase	2.456
AT3G18230	octicosapeptide	2.456
AT1G12680	PEPKR2	2.455
AT4G13660	PRR2	2.455
AT1G63630	pentatricopeptide	2.454
AT5G12400	PHD-finger and DNA bindi	2.454
AT1G72560	PSD	2.454
AT5G23270	STP11	2.454
AT1G32090	early-responsive to dehyd	2.453
AT2G17010	mechanosensitive ion cha	2.452
AT5G46740	UBP21	2.451
AT3G52060	Core-2	2.451
AT1G30840	PUP4	2.451
AT2G40520	nucleotidyltransferase pro	2.450
AT2G43730	jacalin-like lectin domain-c	2.449
AT5G35750	HK2	2.449
AT1G04400	CRY2	2.449
AT3G13830	putative F-box protein	2.449
AT4G33020	ZIP9	2.449
AT1G34060	Pyridoxal phosphate-depe	2.448
AT1G10060	BCAT-1	2.448
AT2G45960	PIP1B	2.446
AT5G26340	MSS1	2.445
AT2G03730	ACR5	2.445
AT3G27700	zinc finger CCCH domain-c	2.444
AT1G79950	regulator of telomere elor	2.443
AT4G04610	APR1	2.443
AT5G10900	serine	2.442
AT2G02061	nucleotide-diphospho-sug	2.442
AT5G51690	ACS12	2.441
AT1G61390	putative S-locus protein ki	2.439
AT5G13530	KEG	2.439
AT1G70520	CRK2	2.439
AT5G53550	YSL3	2.438
AT1G53440	putative LRR receptor-like	2.436
AT4G02680	EOL1	2.436
AT4G31710	GLR2.4	2.436
AT3G55480	PAT2	2.435
AT3G02250	O-fucosyltransferase-like p	2.435
AT1G16110	WAKL6	2.434
AT4G37150	MES9	2.434
AT5G23000	MYB37	2.433
AT3G07540	formin-like protein 10	2.433
AT5G14640	SK13	2.432
AT4G12640	RNA recognition motif	2.432
AT3G04980	DNAJ heat shock N-termin	2.429
AT1G48400	F-box	2.429
AT1G54450	calcium-binding EF-hand-c	2.429
AT3G53800	Fes1B	2.429
AT3G07860	U11	2.428

AT4G30360	CNGC17	2.428
AT5G28830	EF-hand, calcium binding r	2.428
AT5G38210	protein kinase-like protein	2.427
AT1G70210	CYCD1	2.427
AT5G49150	GEX2	2.426
AT3G55540	nuclear transport factor 2	2.426
AT2G20470	putative serine	2.425
AT3G43670	putative copper amine oxi	2.424
AT1G08590	leucine-rich receptor-like p	2.424
AT5G10600	CYP81K2	2.424
AT4G22540	ORP2A	2.422
AT1G13700	PGL1	2.422
AT2G39480	PGP6	2.422
AT5G25120	CYP71B11	2.422
AT2G25010	aminotransferase-like, pla	2.421
AT5G52850	pentatricopeptide repeat-	2.420
AT5G46400	PRP39-2	2.420
AT3G04910	WNK1	2.420
AT1G69450	Early-responsive to dehyd	2.419
AT2G01900	endonuclease	2.418
AT3G01570	oleosin 5	2.417
AT1G10210	MPK1	2.417
AT1G20640	RWP-RK domain-containin	2.416
AT4G39180	SEC14	2.415
AT3G09850	D111	2.415
AT2G20320	DENN	2.412
AT4G38480	transducin	2.411
AT5G03910	ATH12	2.410
AT5G08310	pentatricopeptide repeat-	2.407
AT4G23810	WRKY53	2.407
AT1G12775	pentatricopeptide repeat-	2.406
AT5G17680	putative TIR-NBS-LRR class	2.405
AT1G16270	octicosapeptide	2.404
AT1G26810	GALT1	2.404
AT3G51480	GLR3.6	2.403
AT3G45630	CCR4-NOT transcription cc	2.403
AT1G51530	RNA recognition motif-cor	2.401
AT5G47260	putative disease resistance	2.401
AT2G24240	BTB	2.401
AT1G31790	pentatricopeptide repeat-	2.400
AT1G13140	CYP86C3	2.399
AT1G30720	FAD-binding and BBE dom	2.399
AT3G29340	C2H2-type zinc finger prot	2.397
AT5G21170	AKINBETA1	2.397
AT5G55600	agenet domain-containing	2.397
AT2G46050	pentatricopeptide repeat-	2.395
AT1G27750	nucleic acid binding protei	2.394
AT4G14180	PRD1	2.393
AT5G09950	pentatricopeptide repeat-	2.393
AT1G79030	DNAJ heat shock N-termin	2.393

AT5G44220	F-box protein	2.393
AT5G10370	helicase , IBR and zinc fing	2.393
AT4G08580	microfibrillar-associated p	2.392
AT3G50420	pentatricopeptide repeat-	2.392
AT1G61410	DNA double-strand break	2.392
AT2G27170	TTN7	2.392
AT1G05460	SDE3	2.392
AT2G35050	octicosapeptide	2.391
AT4G05150	octicosapeptide	2.391
AT5G56780	ET2	2.390
AT1G43850	SEU	2.390
AT2G38410	VHS and GAT domain-cont	2.390
AT5G52882	putative ATP binding prote	2.389
AT2G44460	BGLU28	2.389
AT5G48410	GLR1.3	2.389
AT5G54730	G18F	2.389
AT4G00240	PLDBETA2	2.388
AT2G31260	APG9	2.388
AT1G17060	CYP72C1	2.387
AT2G28940	protein kinase family prot	2.384
AT3G55550	concanavalin A-like lectin	2.384
AT3G15720	polygalacturonase	2.383
AT3G54910	leucine rich repeat and F-E	2.383
AT5G20420	CHR42	2.383
AT1G51680	4CL1	2.383
AT4G31060	ethylene-responsive trans	2.382
AT4G26680	pentatricopeptide repeat-	2.382
AT4G29140	mate efflux domain-conta	2.382
AT5G52552	CPuORF14	2.381
AT3G60050	pentatricopeptide repeat-	2.380
AT2G41210	PIP5K5	2.379
AT1G63730	TIR-NBS-LRR class disease	2.379
AT1G50240	FU	2.379
AT5G08190	NF-YB12	2.379
AT5G10250	DOT3	2.379
AT5G55390	EDM2	2.378
AT3G20080	CYP705A15	2.378
AT5G12840	NF-YA1	2.378
AT1G25270	putative MtN21 nodulin p	2.378
AT3G27910	kelch repeat-containing pr	2.378
AT3G48050	BAH and TFIIS domain-con	2.377
AT1G61370	S-locus lectin protein kina	2.376
AT5G53020	Ribonuclease P protein sul	2.375
AT1G49160	WNK7	2.375
AT4G16890	SNC1	2.375
AT5G07670	F-box protein	2.375
AT3G22460	OASA2	2.374
AT2G16050	Cysteine	2.373
AT4G21323	Subtilase family protein	2.373
AT1G68930	pentatricopeptide repeat-	2.372

AT1G29920	CAB2	2.371
AT3G20040	ATHXK4	2.370
AT3G54460	F-box protein	2.370
AT2G23030	SNRK2.9	2.370
AT1G58060	helicase associated domai	2.369
AT3G61680	lipase class 3 family protei	2.369
AT1G05835	PHD finger protein	2.369
AT3G18930	RING-H2 finger protein AT	2.368
AT5G45790	ubiquitin carboxyl-termina	2.367
AT1G14070	FUT7	2.367
AT2G45960	PIP1B	2.367
AT3G61780	emb1703	2.366
AT2G35150	EXL1	2.366
AT5G67340	U-box domain-containing	2.366
AT1G52800	oxidoreductase, 2OG-Fe	2.366
AT2G22420	peroxidase	2.365
AT5G10890	myosin heavy chain-like pı	2.364
AT5G13000	GSL12	2.364
AT5G43920	transducin	2.364
AT3G54350	emb1967	2.364
AT5G18475	pentatricopeptide repeat-	2.364
AT1G70060	SNL4	2.363
AT5G60690	REV	2.362
AT5G01930	mannan endo-1,4-beta-ma	2.361
AT3G14840	Leucine-rich repeat transn	2.361
AT1G08620	PKDM7D	2.360
AT1G20860	PHT1	2.359
AT4G21390	B120	2.359
AT2G36350	protein kinase-like proteir	2.359
AT1G07520	GRAS family transcription	2.359
ATCG01050	ndhD	2.359
AT2G43020	PAO2	2.359
AT3G55320	PGP20	2.359
AT4G02710	kinase interacting-like pro	2.357
AT1G01790	KEA1	2.357
AT1G36370	SHM7	2.357
AT4G39090	RD19	2.357
AT5G23750	Remorin family protein	2.357
AT5G39380	calmodulin-binding proteir	2.356
AT3G18020	pentatricopeptide repeat-	2.356
AT3G58810	MTPA2	2.356
AT5G51260	HAD superfamily, subfami	2.356
AT2G41450	N-acetyltransferase	2.355
AT1G77860	KOM	2.354
AT3G03600	rps2	2.354
AT5G17790	VAR3	2.354
AT1G67810	SUFE2	2.354
AT5G18260	protein binding	2.353
AT4G25860	ORP4A	2.353
AT2G37290	RabGAP	2.352

AT3G61960	protein kinase family prot	2.352
AT3G48060	BAH and TFIIS domain-con	2.351
AT3G54230	SUA	2.351
AT3G27440	UKL5	2.350
AT4G08480	MAPKKK9	2.350
AT3G61630	CRF6	2.350
AT5G41240	GSTT2	2.349
AT4G23930	late embryogenesis abund	2.349
AT2G41710	AP2-like ethylene-respons	2.349
AT4G16610	C2H2-like zinc finger prote	2.349
AT5G05140	Transcription elongation f	2.348
AT3G26910	hydroxyproline-rich glycop	2.348
AT4G33150	lysine-ketoglutarate reduc	2.347
AT2G43680	IQD14	2.347
AT4G23550	WRKY29	2.347
AT4G14590	emb2739	2.347
AT5G23340	leucine-rich repeats	2.345
AT3G09780	CCR1	2.344
AT1G17500	phospholipid-translocating	2.344
AT4G20460	putative UDP-arabinose 4-	2.342
AT1G03800	ERF10	2.342
AT3G22150	pentatricopeptide repeat-	2.341
AT1G60500	DRP4C	2.341
AT3G51340	aspartyl protease family p	2.340
AT5G48600	SMC3	2.340
AT2G30520	RPT2	2.340
AT5G50260	putative cysteine proteina	2.340
AT1G11960	putative ERD4 protein	2.338
AT5G53900	Serine	2.337
AT3G02885	GASA5	2.336
AT2G24650	sequence-specific DNA bir	2.336
AT1G50830	Aminotransferase-like, pla	2.336
AT3G03300	DCL2	2.335
AT3G26922	leucine rich repeat and F-b	2.334
AT5G39680	EMB2744	2.334
AT4G26440	WRKY34	2.334
AT4G03415	putative protein phosphat	2.333
AT4G35470	PIRL4	2.333
AT1G66970	SVL2	2.332
AT1G16710	HAC12	2.332
AT3G61060	PP2-A13	2.332
AT2G03150	emb1579	2.331
AT1G45233	THO5	2.331
AT4G39850	PXA1	2.331
AT4G20040	Pectin lyase-like protein	2.331
AT2G25850	PAPS2	2.330
AT1G74120	Mitochondrial transcriptio	2.330
AT1G04610	YUC3	2.329
AT4G28710	XIH	2.329
AT2G28830	PUB12	2.328

AT4G15415	ATB' GAMMA	2.328
AT5G18630	alpha	2.328
AT4G17080	Histone H3 K4-specific me	2.328
AT3G26810	AFB2	2.328
AT1G75660	XRN3	2.327
AT5G19420	regulator of chromosome	2.327
AT2G28470	BGAL8	2.327
AT5G43910	pfkB-like carbohydrate kin	2.327
AT5G12420	O-acyltransferase	2.327
AT1G08650	PPCK1	2.327
AT5G45060	TIR-NBS-LRR class disease	2.325
AT5G13820	TBP1	2.324
AT5G23080	TGH	2.323
AT5G01770	RAPTOR2	2.323
AT5G49330	MYB111	2.323
AT3G56440	ATG18D	2.323
AT3G04050	pyruvate kinase	2.322
AT5G55310	TOP1BETA	2.321
AT5G59980	ribonuclease P subunit Rp	2.321
AT5G47690	sister chromatid cohesion	2.320
AT3G58620	TTL4	2.319
AT2G13600	pentatricopeptide repeat-	2.318
AT5G11250	TIR-NBS-LRR class disease	2.318
AT5G52800	DNA primase	2.318
AT5G42450	pentatricopeptide repeat-	2.317
AT5G03790	HB51	2.317
AT2G28620	P-loop containing nucleosi	2.317
AT2G33435	RNA recognition motif-cor	2.316
AT3G26740	CCL	2.316
AT5G13330	Rap2.6L	2.316
AT5G49310	IMPA-5	2.316
AT5G16680	RING	2.315
AT5G07970	dentin sialophosphoprotei	2.315
AT1G58200	MSL3	2.314
AT4G28430	reticulon-like protein B18	2.313
AT3G28030	UVH3	2.313
AT5G58040	FIP1	2.312
AT3G12280	RBR1	2.312
AT3G11340	UDP-glycosyltransferase-li	2.311
AT3G62330	CCHC-type zinc knuckle pr	2.310
AT3G58590	pentatricopeptide repeat-	2.310
AT3G07160	GSL10	2.310
AT2G31960	GSL03	2.309
AT4G11900	S-locus lectin protein kina	2.309
AT4G00060	MEE44	2.309
AT3G57660	NRPA1	2.308
AT1G04730	CTF18	2.308
AT2G17620	CYCB2	2.308
AT1G29730	Leucine-rich repeat transn	2.308
AT2G26700	PID2	2.308

AT4G20420	Tapetum specific protein T	2.308
AT5G58160	formin-like protein 13	2.308
AT3G48195	Phox	2.307
AT3G51110	Half-A-TPR repeat-contain	2.307
AT1G13220	LINC2	2.306
AT4G20090	EMB1025	2.305
AT4G00020	BRCA2	2.304
AT1G17680	tetratricopeptide repeat	2.304
AT1G68110	putative clathrin assembly	2.304
AT5G54670	ATK3	2.304
AT5G35390	leucine-rich repeat protein	2.303
AT2G04430	NUDT5	2.302
AT1G22930	T-complex protein 11	2.302
AT5G53500	transducin	2.302
AT1G02060	pentatricopeptide repeat-	2.302
AT1G48490	protein kinase domain-cor	2.302
AT1G49900	C2H2 type zinc finger trans	2.302
AT1G77030	DEAD-box helicase domain	2.301
AT5G22500	FAR1	2.301
AT4G16835	tetratricopeptide repeat d	2.301
AT1G74580	pentatricopeptide repeat-	2.300
AT3G13340	transducin	2.300
AT5G40400	pentatricopeptide repeat-	2.300
AT5G57610	octicosapeptide	2.299
AT5G19010	MPK16	2.299
AT5G51270	U-box domain-containing	2.299
AT5G37630	EMB2656	2.298
AT2G38640	protein LURP-one-related	2.298
AT1G01320	tetratricopeptide repeat-c	2.297
AT5G03680	PTL	2.297
AT3G49400	transducin	2.297
AT2G29065	GRAS family transcription	2.296
AT1G52870	Peroxisomal membrane 2	2.296
AT5G38050	RNA polymerase II transcr	2.295
AT1G10810	NAD	2.294
AT5G14080	pentatricopeptide repeat-	2.294
AT1G69570	Dof zinc finger protein DO	2.294
AT2G02070	IDD5	2.294
AT3G18524	MSH2	2.293
AT1G15060	PhaC and hydrolase doma	2.293
AT1G73750	esterase	2.292
AT5G44400	FAD-binding and BBE dom	2.292
AT1G30810	transcription factor jumon	2.291
AT3G01320	SNL1	2.291
AT4G24560	UBP16	2.291
AT3G28580	AAA-type ATPase family p	2.290
AT2G35630	MOR1	2.290
AT4G01020	zinc finger-related and hel	2.290
AT1G08730	XIC	2.289
AT3G51080	GATA6	2.289

AT5G24620	pathogenesis-related thau	2.288
AT1G17580	MYA1	2.288
AT2G28260	CNGC15	2.288
AT1G35830	VQ motif-containing prote	2.285
AT5G46450	TIR-NBS-LRR class disease	2.285
AT5G49450	bZIP1	2.285
AT1G67840	CSK	2.284
AT4G02070	MSH6	2.284
AT5G43370	PHT1	2.283
AT3G25070	RIN4	2.282
AT5G43470	RPP8	2.282
AT4G18710	BIN2	2.281
AT2G42880	MPK20	2.281
AT4G13650	pentatricopeptide repeat-	2.281
AT1G33440	putative peptide	2.281
AT3G23610	DSPTP1	2.280
AT1G23210	GH9B6	2.280
AT1G58190	RLP9	2.280
AT1G03780	TPX2	2.279
AT4G12350	MYB42	2.279
AT1G04210	leucine-rich repeat proteir	2.278
AT5G03780	TRFL10	2.277
AT4G20720	dentin sialophosphoprotei	2.277
AT2G33340	MAC3B	2.276
AT2G16500	ADC1	2.276
AT2G33240	XID	2.275
AT4G33040	glutaredoxin-C6	2.275
AT1G67160	putative F-box protein	2.275
AT1G69080	adenine nucleotide alpha I	2.273
AT1G17240	RLP2	2.273
AT1G59740	putative peptide	2.272
AT1G68400	leucine-rich repeat transm	2.271
AT1G50420	SCL3	2.270
AT1G03100	pentatricopeptide repeat-	2.269
AT1G67710	ARR11	2.269
AT3G07520	GLR1.4	2.268
AT3G13020	hAT dimerization domain-	2.268
AT5G19920	transducin	2.268
AT3G12940	dioxygenase-like protein	2.267
AT1G61490	putative S-locus protein ki	2.267
AT2G36490	DML1	2.267
AT4G11570	haloacid dehalogenase-lik	2.267
AT1G05630	5PTASE13	2.266
AT2G36830	GAMMA-TIP	2.266
AT3G51740	IMK2	2.265
AT4G18750	DOT4	2.265
AT1G09410	pentatricopeptide repeat-	2.265
ATCG00490	rbcL	2.265
AT1G63770	Peptidase M1 family prote	2.263
AT5G09840	Putative endonuclease or	2.262

AT1G53330	pentatricopeptide repeat-	2.262
AT2G38440	SCAR2	2.262
AT5G52100	crr1	2.262
AT5G19400	SMG7	2.261
AT1G07270	cell division control protei	2.261
AT4G39160	DNA binding	2.260
AT2G20190	CLASP	2.260
AT3G15130	pentatricopeptide repeat-	2.259
AT2G16390	DRD1	2.259
AT3G52890	KIPK	2.259
ATCG00280	psbC	2.259
AT3G50970	LTI30	2.258
AT1G54160	NF-YA5	2.258
AT5G27930	putative protein phosphat	2.257
AT5G22760	PHD finger family protein	2.257
AT1G34780	APRL4	2.256
AT4G20110	VSR7	2.255
AT3G07550	F-box	2.255
AT5G60410	SIZ1	2.255
AT1G53510	MPK18	2.255
AT1G27910	PUB45	2.255
AT4G17410	DWNN domain-containing	2.254
AT3G42670	CHR38	2.254
AT5G07710	exonuclease family protei	2.254
AT3G23120	RLP38	2.253
AT2G37450	nodulin MtN21	2.253
AT4G38770	PRP4	2.253
AT5G01540	LECRKA4.1	2.253
AT3G07530	integrator complex subuni	2.253
AT1G02610	RING	2.253
AT2G43930	protein kinase-like protein	2.252
AT1G50620	RING	2.252
AT3G13380	BRL3	2.252
AT1G58050	helicase domain-containin	2.252
AT3G43920	DCL3	2.251
AT4G04850	KEA3	2.251
AT3G12810	PIE1	2.250
AT4G03260	Outer arm dynein light cha	2.250
AT3G07040	RPM1	2.250
AT3G13110	SERAT2	2.249
AT5G15020	SNL2	2.249
AT2G37420	ATP binding microtubule r	2.248
AT2G19330	PIRL6	2.248
AT1G70760	CRR23	2.248
AT1G73400	pentatricopeptide repeat-	2.248
AT4G30520	leucine-rich repeat protei	2.247
AT1G55250	HUB2	2.245
AT5G47510	Sec14p-like phosphatidylir	2.245
AT1G59620	CW9	2.245
AT1G34750	putative protein phosphat	2.244

AT3G15750	Yae1 domain-containing p	2.244
AT4G33160	F-box only protein 13	2.244
AT5G25280	serine-rich protein-like pro	2.244
AT4G11970	YT521-B-like protein	2.243
AT1G53400	Ubiquitin domain-containi	2.243
AT2G01390	pentatricopeptide repeat-	2.242
AT5G64840	GCN5	2.242
AT3G16340	PDR1	2.242
AT1G65440	GTB1	2.241
AT3G49800	BSD domain-containing pr	2.241
AT1G22860	Vacuolar sorting protein 3	2.241
AT2G34730	WPP domain-associated p	2.240
ATCG00170	rpoC2	2.240
AT5G47500	putative pectinesterase 68	2.240
AT4G25150	HAD superfamily, subfami	2.240
AT5G40390	SIP1	2.240
AT1G74680	exostosin-like protein	2.239
AT2G25930	ELF3	2.239
AT4G14970	fanconi anemia group D2 p	2.238
AT5G43730	CC-NBS-LRR class disease r	2.238
AT5G05330	HMG-box	2.238
AT3G24715	octicosapeptide	2.237
AT5G24240	phosphatidylinositol 3- an	2.237
AT3G49650	P-loop containing nucleosi	2.237
AT5G19330	ARIA	2.237
AT1G72000	putative invertase	2.237
AT4G11460	CRK30	2.236
AT5G42760	Leucine carboxyl methyltr	2.235
AT4G24890	PAP24	2.235
AT1G52430	Ubiquitin carboxyl-termina	2.235
AT1G66980	SNC4	2.234
AT1G08090	NRT2:1	2.234
AT5G46370	KCO2	2.234
AT5G63930	leucine-rich repeat protein	2.233
AT1G77600	sister chromatid cohesion	2.232
AT5G56200	C2H2 type zinc finger trans	2.231
AT4G25880	PUM6	2.231
AT4G31750	WIN2	2.231
AT1G35560	transcription factor TCP23	2.231
AT1G63480	AT hook motif DNA-bindin	2.230
AT3G26930	FBD and leucine rich repea	2.230
AT1G48110	ECT7	2.230
AT4G39100	SHL1	2.230
AT1G34355	PS1	2.229
AT1G07480	Transcription factor IIA, alp	2.229
AT4G23240	CRK16	2.229
AT2G04690	Pyridoxamine 5'-phosphat	2.229
AT1G64960	condensin-2 complex subu	2.228
AT3G23670	KINESIN-12B	2.227
AT4G38120	armadillo	2.227

AT1G05805	transcription factor bHLH1	2.227
AT1G29700	metallo-beta-lactamase do	2.227
AT4G00500	lipase class 3 family protei	2.227
AT2G07751	NADH dehydrogenase I su	2.227
AT1G01610	GPAT4	2.226
AT3G61420	transcription initiation fac	2.226
AT5G15210	HB30	2.226
AT5G41840	putative F-box	2.225
AT2G34680	AIR9	2.225
AT2G30890	Cytochrome b561	2.225
AT2G34780	MEE22	2.224
AT2G23520	catalytic	2.224
AT3G11460	pentatricopeptide repeat-	2.223
AT5G57690	DGK4	2.223
AT5G61228	CPuORF15	2.222
AT1G78130	UNE2	2.222
AT5G16210	HEAT repeat-containing pr	2.221
AT5G63130	octicosapeptide	2.221
AT1G19850	MP	2.220
AT4G20850	TPP2	2.220
AT4G14465	AHL20	2.220
AT3G20150	Kinesin motor family prote	2.219
AT1G14780	MAC	2.219
AT1G78320	GSTU23	2.218
AT1G48650	DEA	2.218
AT2G40190	LEW3	2.218
AT1G24190	SNL3	2.217
AT3G18710	PUB29	2.217
AT1G18370	HIK	2.216
AT4G17895	UBP20	2.216
AT4G32820	tetratricopeptide repeat d	2.216
AT1G78930	Mitochondrial transcriptio	2.216
AT2G34520	rps14	2.216
AT1G73710	pentatricopeptide repeat-	2.216
AT3G27560	ATN1	2.215
AT5G27030	TPR3	2.215
AT3G11670	DGD1	2.215
AT1G63380	Rossmann-fold NAD	2.215
AT1G47560	SEC3B	2.214
AT4G18975	pentatricopeptide repeat-	2.214
AT5G40420	OLEO2	2.213
AT4G13960	putative F-box	2.213
AT5G58870	ftsh9	2.213
AT1G62490	Mitochondrial transcriptio	2.213
AT4G29880	PIRL7	2.213
AT1G06580	pentatricopeptide repeat-	2.213
AT5G43360	PHT1	2.212
AT5G65350	HTR11	2.212
AT4G18020	APRR2	2.212
AT4G01720	WRKY47	2.212

AT4G03250	homeobox-leucine zipper	2.211
AT4G21120	AAT1	2.211
AT3G04590	AT hook motif DNA-bindin	2.211
AT1G73860	putative Kinesin motor pro	2.210
AT3G62270	putative boron transporte	2.210
AT2G02250	PP2-B2	2.209
AT5G17580	Phototropic-responsive NI	2.209
AT3G57410	VLN3	2.209
AT3G06910	ULP1A	2.209
AT2G25540	CESA10	2.208
AT1G68890	2-oxoglutarate decarboxyl	2.208
AT1G05710	basic helix-loop-helix dom	2.208
AT2G17760	aspartyl protease-like prot	2.208
AT5G18750	DNAJ heat shock N-termin	2.207
AT1G10760	SEX1	2.207
AT1G53390	ABC transporter G-24	2.207
AT5G45710	RHA1	2.207
AT1G70250	putative receptor serine	2.206
AT1G45160	protein kinase-like protein	2.206
AT5G49760	leucine-rich repeat protein	2.206
AT4G10120	ATSPS4F	2.206
AT2G17260	GLR2	2.204
AT2G34920	EDA18	2.204
AT4G25270	pentatricopeptide repeat-	2.204
AT3G51560	TIR-NBS-LRR class disease	2.203
AT5G41820	RGTA2	2.203
AT5G47140	GATA27	2.203
AT2G42290	leucine-rich repeat-like pro	2.203
AT5G38850	TIR-NBS-LRR class disease	2.202
AT1G06230	GTE4	2.202
AT4G25650	ACD1-LIKE	2.202
AT3G63200	PLP9	2.202
AT4G35300	TMT2	2.201
AT5G66270	zinc finger CCCH domain-c	2.201
AT1G14340	RNA recognition motif-cor	2.201
AT1G14460	AAA-type ATPase family pr	2.200
AT5G11510	MYB3R-4	2.200
AT3G22600	bifunctional inhibitor	2.200
AT2G33760	pentatricopeptide repeat-	2.200
AT3G63370	OTP86	2.199
AT4G23690	disease resistance-respons	2.199
ATCG00520	ycf4	2.199
AT5G18140	chaperone DnaJ-domain c	2.198
AT5G45428	CPuORF24	2.198
AT2G39360	putative receptor-like prot	2.198
AT1G02460	Pectin lyase-like protein	2.198
AT1G17950	MYB52	2.197
AT2G15300	leucine-rich repeat protein	2.197
AT1G51690	B ALPHA	2.197
AT1G70895	CLE17	2.197

AT5G67460	O-Glycosyl hydrolases fam	2.197
AT1G32850	UBP11	2.197
AT3G51120	zinc finger CCCH domain-c	2.196
AT2G33050	RLP26	2.195
AT4G25120	SRS2	2.195
AT5G44090	protein phosphatase 2	2.195
AT1G70620	cyclin-related protein	2.195
AT5G13630	GUN5	2.195
AT2G15560	Putative endonuclease or	2.195
AT1G73950	Transmembrane Fragile-X-	2.194
AT4G05160	4-coumarate--CoA ligase-l	2.194
AT2G32930	ZFN2	2.194
AT5G47790	SMAD	2.194
AT5G40480	EMB3012	2.193
AT5G07400	forkhead-associated doma	2.193
AT3G49710	pentatricopeptide repeat-	2.192
AT1G69710	regulator of chromosome	2.192
AT1G09570	PHYA	2.192
AT1G61140	EDA16	2.192
AT3G22860	TIF3C2	2.191
AT2G03340	WRKY3	2.191
AT4G04670	tRNA wybutosine synthesi	2.190
AT1G29630	exonuclease 1	2.190
AT1G22370	UGT85A5	2.190
AT5G51380	F-box protein	2.190
AT1G21270	WAK2	2.190
AT5G13010	EMB3011	2.189
AT3G17750	protein kinase family prot	2.189
AT1G07470	transcription initiation fac	2.189
AT3G57720	putative protein kinase	2.187
AT5G25820	Exostosin family protein	2.187
AT1G77580	filament-like plant protein	2.186
AT3G07010	putative pectate lyase 8	2.186
AT1G20470	SAUR-like auxin-responsiv	2.185
AT4G35300	TMT2	2.185
AT1G64180	intracellular protein trans	2.185
AT3G45420	concanavalin A-like lectin	2.184
AT5G03280	EIN2	2.184
AT1G07880	ATMPK13	2.183
AT5G52530	dentin sialophosphoprote	2.183
AT5G46700	TRN2	2.183
AT2G36485	ENTH	2.183
AT2G36850	GSL8	2.182
AT1G18880	putative peptide	2.181
AT3G18620	putative S-acyltransferase	2.181
AT3G27730	RCK	2.180
AT2G42920	pentatricopeptide repeat-	2.180
AT1G21160	eukaryotic translation initi	2.180
AT3G03140	PWWP domain-containing	2.180
AT1G51400	Photosystem II 5 kD prote	2.179

AT2G48160	Tudor	2.178
AT5G37180	SUS5	2.177
AT1G09220	pentatricopeptide repeat-	2.177
AT3G10310	myosin and kinesin motor	2.176
AT3G15300	VQ motif-containing prote	2.176
AT3G27690	LHCB2.3	2.176
AT3G52120	Splicing factor 4-like prote	2.175
AT1G64610	WD40 domain-containing	2.175
AT3G01370	CFM2	2.175
AT3G59150	F-box domain-containing p	2.174
AT3G13880	pentatricopeptide repeat-	2.174
AT2G40130	heat shock-related proteir	2.174
AT1G75120	RRA1	2.174
AT2G29760	OTP81	2.173
AT5G18590	kelch repeat-containing pr	2.173
AT4G08470	MEKK3	2.172
AT5G04240	ELF6	2.172
AT1G79740	hAT family dimerization do	2.172
AT4G39710	FKBP16-2	2.172
AT2G35110	GRL	2.172
AT3G06140	RING	2.172
AT2G01320	ABC transporter G family r	2.171
AT5G49980	AFB5	2.171
AT5G42440	protein kinase family prot	2.170
AT1G08050	C3HC4-type RING finger-co	2.169
ATCG00500	accD	2.169
AT5G01630	BRCA2B	2.168
AT1G20230	pentatricopeptide repeat-	2.168
AT5G52390	PAR1 protein	2.167
AT2G17770	BZIP27	2.167
AT5G20490	XIK	2.166
AT1G76630	superkiller protein 3-like p	2.166
AT1G73920	alpha	2.166
AT5G47010	LBA1	2.165
AT1G28340	RLP4	2.165
AT1G31850	putative methyltransferas	2.165
AT5G52120	PP2-A14	2.164
AT4G17330	G2484-1	2.163
AT1G77570	Winged helix-turn-helix tra	2.163
AT2G27950	RING	2.163
AT5G39980	pentatricopeptide repeat-	2.163
AT1G02470	SRPBCC ligand-binding dor	2.163
AT5G50160	FRO8	2.162
AT2G43800	formin-like protein 2	2.161
AT1G19000	myb family transcription fa	2.161
AT1G06270	pentatricopeptide repeat-	2.160
AT3G06010	ATCHR12	2.160
AT2G25320	TRAF-like protein	2.160
AT4G32940	GAMMA-VPE	2.160
AT4G18020	APRR2	2.160

AT5G48920	TED7	2.160
AT5G18240	MYR1	2.159
AT3G23950	F-box protein	2.159
AT5G57360	ZTL	2.159
AT5G01400	ESP4	2.158
AT3G01600	NAC044	2.158
AT5G65110	ACX2	2.158
AT3G01610	CDC48C	2.157
AT2G42240	RNA recognition motif-cor	2.157
AT2G39350	ABC transporter G family r	2.157
AT2G23450	wall-associated receptor k	2.157
AT3G10180	centromeric protein E	2.156
AT4G08540	DNA-directed RNA polyme	2.156
AT4G04790	pentatricopeptide repeat-	2.156
AT5G51230	EMF2	2.155
AT5G46350	WRKY8	2.155
AT3G20080	CYP705A15	2.155
AT1G26500	pentatricopeptide repeat-	2.155
AT3G04630	WDL1	2.155
AT1G11260	STP1	2.155
AT1G57700	protein kinase-like protein	2.155
AT5G27320	GID1C	2.155
AT3G59050	PAO3	2.155
AT4G03100	Rho GTPase activating pro	2.154
AT2G03240	phosphate transporter PH	2.154
AT5G57740	XBAT32	2.154
AT4G02410	concanavalin A-like lectin	2.154
AT1G09600	protein kinase-like protein	2.154
AT3G54230	SUA	2.153
AT3G13030	hAT dimerization domain-	2.153
AT2G46560	transducin family protein	2.152
AT3G17450	hAT dimerization domain-	2.152
AT5G04040	SDP1	2.152
AT4G13990	Exostosin family protein	2.152
AT3G60670	PLATZ transcription factor	2.152
AT4G24200	Transcription elongation fa	2.151
AT3G15620	UVR3	2.151
AT5G61600	ERF104	2.150
AT4G12750	Homeodomain-like transc	2.150
AT1G75700	HVA22G	2.150
AT5G15170	TDP1	2.149
AT1G73960	TAF2	2.149
AT3G03700	Plasma-membrane choline	2.149
AT5G18240	MYR1	2.148
AT4G38230	CPK26	2.147
AT5G20960	AO1	2.147
AT1G67030	ZFP6	2.147
AT3G22380	TIC	2.147
AT1G05030	putative plastidic glucose t	2.146
AT2G46920	POL	2.146

AT4G12090	Cornichon family protein	2.146
AT2G41710	AP2-like ethylene-respons	2.146
AT4G02280	SUS3	2.146
AT5G14490	NAC085	2.145
AT1G52940	PAP5	2.145
AT1G71960	ABCG25	2.145
AT1G52620	pentatricopeptide repeat-	2.145
AT5G07610	F-box protein	2.145
AT2G26030	F-box	2.144
AT2G28890	PLL4	2.144
AT3G46780	PTAC16	2.143
AT3G21530	endonuclease	2.143
AT4G24860	AAA-type ATPase family pı	2.140
AT2G27980	acyl-CoA N-acyltransferase	2.140
AT4G39560	F-box	2.138
AT4G26850	VTC2	2.138
AT3G56550	pentatricopeptide repeat-	2.138
AT1G78290	serine	2.138
AT5G62240	Cell cycle regulated microt	2.138
AT5G57190	PSD2	2.138
AT1G51350	armadillo	2.138
AT5G35370	S-locus lectin protein kina:	2.138
AT1G80440	F-box	2.138
AT5G39420	cdc2cAt	2.138
AT1G27595	symplekin	2.137
AT5G37930	E3 ubiquitin-protein ligase	2.136
AT4G05190	ATK5	2.135
AT3G04670	WRKY39	2.135
AT4G21060	Galactosyltransferase fam	2.135
AT2G29190	PUM2	2.135
AT5G61420	MYB28	2.135
AT3G52370	FLA15	2.135
AT3G12020	kinesin motor protein-like	2.134
AT5G44600	S-adenosyl-L-methionine-(	2.134
AT5G22700	F-box	2.134
AT1G64460	protein kinase-like protein	2.134
AT2G30505	late embryogenesis abund	2.133
AT2G24150	HHP3	2.133
AT5G48390	ATZIP4	2.132
AT1G22985	ethylene-responsive trans	2.132
AT5G39670	putative calcium-binding p	2.132
AT1G68920	transcription factor bHLH4	2.131
AT3G18730	TSK	2.130
AT1G47128	RD21	2.130
AT2G32850	AP2-associated kinase	2.129
AT4G36060	bHLH11	2.129
AT5G27970	armadillo	2.129
AT4G24680	MOS1	2.128
AT3G24020	disease resistance-respon:	2.128
AT3G45000	VPS24.2	2.128

AT3G15354	SPA3	2.128
AT3G29680	HXXXD-type acyl-transfera	2.127
AT4G02050	STP7	2.127
AT2G32250	FRS2	2.127
AT1G60930	RECQ4B	2.126
AT2G26780	proteasome component E	2.126
AT4G27730	OPT6	2.126
AT3G14770	nodulin MtN3-like protein	2.126
AT1G05065	CLE20	2.126
AT3G18070	BGLU43	2.126
AT3G22790	kinase interacting KIP1-lik	2.125
AT2G34420	LHB1B2	2.125
AT3G12280	RBR1	2.125
AT3G56040	UGP3	2.124
AT1G12110	NRT1.1	2.124
AT5G51560	leucine-rich repeat protei	2.122
AT3G01650	RGLG1	2.122
AT3G47950	HA4	2.122
AT4G12900	Gamma interferon respon	2.121
AT2G47900	TLP3	2.121
AT1G49340	ATPI4K ALPHA	2.121
AT2G42890	ML2	2.121
AT4G35750	SEC14 cytosolic factor fam	2.121
AT5G12350	Regulator of chromosome	2.121
AT3G13682	LDL2	2.120
AT5G64420	DNA polymerase phi subu	2.119
AT4G19060	putative disease resistanc	2.118
AT1G04390	BTB	2.118
AT1G26770	EXPA10	2.118
AT2G19920	RNA-dependent RNA poly	2.117
AT3G49500	RDR6	2.117
AT1G43245	SET domain-containing pr	2.116
AT5G18240	MYR1	2.116
AT5G65910	BSD domain-containing pr	2.116
AT4G19890	Pentatricopeptide repeat	2.115
AT5G37830	OXP1	2.114
AT4G32600	C3H4 type zinc finger prot	2.114
AT5G65980	auxin efflux carrier family	2.114
AT5G09350	PI-4KBETA2	2.113
AT1G18470	Transmembrane Fragile-X-	2.113
AT1G04840	pentatricopeptide repeat-	2.113
AT4G13580	disease resistance-respon	2.112
AT3G09670	PWWP domain-containing	2.112
AT1G50840	POLGAMMA2	2.112
AT3G23700	ribosomal protein S1-like	2.112
AT1G24440	RING	2.112
AT4G21630	Subtilase family protein	2.112
AT1G72860	TIR-NBS-LRR class disease	2.111
AT4G02750	pentatricopeptide repeat-	2.110
AT1G34420	leucine-rich repeat transm	2.110

AT3G47060	ftsh7	2.110
AT4G11110	SPA2	2.110
AT3G04060	NAC046	2.110
AT1G77720	PPK1	2.109
AT1G66130	Rossmann-fold NAD	2.109
AT2G32250	FRS2	2.108
AT5G65460	KAC2	2.107
AT3G60860	SEC7-like guanine nucleoti	2.107
AT2G25350	Phox	2.107
AT3G02380	COL2	2.107
AT2G17140	pentatricopeptide repeat-	2.107
AT1G49920	MuDR family transposase	2.107
AT5G58210	hydroxyproline-rich glycop	2.105
AT2G18876	Afadin	2.105
AT3G21480	BRCT domain-containing D	2.104
AT3G44820	Phototropic-responsive NI	2.104
AT5G64220	calmodulin-binding transc	2.104
AT1G17940	Endosomal targeting BRO1	2.104
AT4G35620	CYCB2	2.102
AT1G62680	pentatricopeptide repeat-	2.101
AT1G80150	pentatricopeptide repeat-	2.100
AT1G30760	FAD-binding and BBE dom	2.100
AT4G19191	pentatricopeptide repeat-	2.100
AT3G20250	PUM5	2.099
AT3G05040	HST	2.099
AT4G01950	GPAT3	2.099
AT4G38310	galactosyl transferase GM	2.099
AT4G35985	senescence	2.099
ATCG00660	rpl20	2.099
AT1G35515	HOS10	2.098
AT3G06210	armadillo	2.098
AT1G17040	SHA	2.098
AT5G39350	pentatricopeptide repeat-	2.097
AT2G37980	O-fucosyltransferase-like p	2.097
AT4G35300	TMT2	2.097
AT5G43600	UAH	2.097
AT5G58787	RING	2.096
AT1G59720	CRR28	2.095
AT1G78955	CAMS1	2.095
AT3G46920	octicosapeptide	2.094
AT5G60280	concanavalin A-like lectin	2.094
AT5G58350	WNK4	2.094
AT2G01810	RING	2.094
AT4G19190	uncharacterized zinc finge	2.093
AT4G30000	Dihydropterin pyrophosph	2.093
AT5G58410	HEAT	2.093
AT3G13330	PA200	2.093
AT5G35970	putative DNA-binding prot	2.093
AT2G30780	pentatricopeptide repeat-	2.093
AT5G51710	KEA5	2.092

AT5G63770	DGK2	2.092
AT4G21430	B160	2.091
AT1G55040	Zn-finger in Ran binding do	2.091
AT1G08440	aluminum activated malat	2.091
AT1G25360	pentatricopeptide repeat-	2.091
AT4G01030	pentatricopeptide repeat-	2.090
AT3G43220	Phosphoinositide phospha	2.090
AT3G06630	protein kinase family prot	2.089
AT1G73655	FKBP-like peptidyl-prolyl c	2.089
AT1G13630	PPR repeat-containing pro	2.089
AT3G44050	kinesin motor protein-like	2.087
AT5G21970	ubiquitin carboxyl-termina	2.087
AT2G22840	GRF1	2.087
AT2G18050	HIS1-3	2.086
AT5G24280	GMI1	2.085
AT4G30350	heat shock-related protein	2.085
AT1G20823	RING-H2 finger protein AT	2.085
AT5G63830	HIT-type Zinc finger family	2.084
AT3G11910	UBP13	2.084
AT3G62310	pre-mRNA-splicing factor /	2.084
AT5G08490	pentatricopeptide repeat-	2.084
AT4G01910	cysteine	2.084
AT5G16900	putative LRR receptor-like	2.083
AT3G22270	Topoisomerase II-associat	2.083
ATCG01010	ndhF	2.083
AT1G45010	TRAM, LAG1 and CLN8	2.083
AT1G17110	UBP15	2.082
AT5G47800	Phototropic-responsive NI	2.082
AT3G63000	NPL41	2.082
AT4G28650	putative leucine-rich repea	2.082
AT5G37570	pentatricopeptide repeat-	2.082
AT4G39530	pentatricopeptide repeat-	2.081
AT5G15700	DNA-directed RNA polyme	2.081
AT1G56600	GolS2	2.081
AT3G06290	SAC3	2.080
AT5G65570	pentatricopeptide repeat-	2.080
AT2G34830	WRKY35	2.080
AT5G40060	TIR-NBS-LRR class disease	2.079
AT1G59750	ARF1	2.079
AT1G56145	Leucine-rich repeat transm	2.079
AT4G11850	PLDGAMMA1	2.079
AT1G80460	NHO1	2.078
AT2G47160	BOR1	2.078
AT3G24340	chr40	2.078
AT3G28960	Transmembrane amino ac	2.078
AT5G45250	RPS4	2.077
AT5G47460	pentatricopeptide repeat-	2.077
AT1G17210	ILP1	2.077
AT1G56690	pentatricopeptide repeat-	2.077
AT3G16490	IQD26	2.077

AT5G20320	DCL4	2.076
AT3G05430	PWWP domain-containing	2.076
AT2G40810	ATG18C	2.076
AT1G69670	CUL3B	2.076
AT4G35640	SERAT3	2.076
AT4G19112	CPuORF25	2.076
AT3G60900	FLA10	2.076
AT3G50710	putative FBD-associated F-	2.075
AT4G22920	NYE1	2.075
AT2G40540	KT2	2.075
AT5G38820	Transmembrane amino ac	2.075
AT4G16920	TIR-NBS-LRR class disease	2.074
AT3G45850	P-loop containing nucleosi	2.074
AT1G68120	BPC3	2.074
AT5G62990	emb1692	2.073
AT4G32360	ferredoxin--NADP+ reduct	2.073
AT1G68920	transcription factor bHLH4	2.073
AT2G19280	pentatricopeptide repeat-	2.073
AT4G09560	protease-associated	2.073
AT4G14540	NF-YB3	2.072
AT5G46760	transcription factor ATR2	2.072
AT3G48670	IDN2	2.071
AT1G49220	RING-H2 finger protein AT	2.071
AT3G15240	serine	2.071
AT1G22620	ATSAC1	2.071
AT1G70670	Caleosin-related family pr	2.071
AT4G00752	UBX domain-containing pr	2.070
AT5G56368	putative defensin-like prot	2.070
AT5G08305	pentatricopeptide repeat	2.069
AT3G51630	WNK5	2.069
AT2G03260	phosphate transporter PH	2.069
AT1G67580	protein kinase-like protein	2.069
AT1G03090	MCCA	2.069
AT5G03555	nucleobase:cation sympor	2.069
AT5G12310	RING	2.068
AT5G20885	RING	2.067
AT4G24690	ubiquitin-associated	2.067
AT1G05120	Helicase protein with RINC	2.067
AT5G09460	transcription factor bHLH1	2.067
AT5G04670	Enhancer of polycomb-like	2.067
AT5G42180	peroxidase 64	2.066
AT5G23210	SCPL34	2.066
AT1G76020	Thioredoxin superfamily p	2.066
AT5G59200	OTP80	2.065
AT3G14050	RSH2	2.064
AT5G09461	CPuORF43	2.064
AT1G35710	putative leucine-rich repe	2.063
AT1G80190	PSF1	2.063
AT4G11360	RHA1B	2.063
AT1G08890	sugar transporter ERD6-lik	2.063

AT1G01760	Adenosine-deaminase	2.063
AT3G22440	FRIGIDA-like protein	2.063
AT3G51180	zinc finger CCCH domain-c	2.062
AT2G38800	calmodulin-binding protei	2.062
AT4G02560	LD	2.062
AT2G03280	O-fucosyltransferase-like p	2.062
AT5G27350	SFP1	2.062
AT5G36870	GSL09	2.061
AT3G12170	chaperone DnaJ-domain c	2.061
AT1G62914	pentatricopeptide repeat-	2.061
AT4G26080	ABI1	2.061
AT5G41150	UVH1	2.060
AT3G27170	CLC-B	2.059
AT3G09010	protein kinase domain-cor	2.059
AT1G21840	UREF	2.059
AT1G56310	3'-5' exonuclease domain-	2.059
AT5G19560	ROPGEF10	2.059
AT5G18770	F-box	2.058
AT3G04380	SUVR4	2.058
AT5G04810	pentatricopeptide	2.058
AT2G26030	F-box	2.058
AT5G24120	SIGE	2.058
AT1G76580	squamosa promoter-bindi	2.058
AT4G11440	Mitochondrial substrate ca	2.058
AT2G29710	UDP-glycosyltransferase-li	2.057
AT5G46070	Guanylate-binding protein	2.056
AT2G33480	NAC041	2.056
AT4G25520	SLK1	2.056
AT4G38550	phospholipase like protein	2.056
AT4G05120	FUR1	2.056
AT1G27340	F-box only protein 6	2.056
AT5G55040	DNA-binding bromodomai	2.055
AT3G49050	alpha	2.055
AT1G68660	ATP-dependent Clp protea	2.055
AT3G53240	RLP45	2.054
AT4G08350	GTA2	2.054
AT5G22720	putative FBD-associated F-	2.054
AT1G54490	XRN4	2.054
AT4G25960	PGP2	2.054
AT4G04370	pentatricopeptide repeat-	2.053
AT4G32510	putative boron transporte	2.053
AT3G19960	ATM1	2.053
AT3G62700	MRP10	2.053
AT4G16370	OPT3	2.053
ATCG00020	psbA	2.053
AT3G20010	SNF2 and helicase domain	2.052
AT1G34120	IP5PI	2.052
AT1G61215	BRD4	2.052
AT1G34110	leucine-rich receptor-like p	2.052
AT2G07680	MRP11	2.050

AT5G23210	SCPL34	2.050
AT2G17270	PHT3	2.050
AT4G39350	CESA2	2.049
AT5G58500	LSH5	2.049
AT5G42390	Insulinase	2.049
AT2G27340	N-acetylglucosaminylphos	2.048
AT2G46710	Rho GTPase activating pro	2.048
AT2G33110	VAMP723	2.048
AT2G07140	F-box and associated inter	2.048
AT2G26170	CYP711A1	2.047
AT5G53450	ORG1	2.047
AT1G76900	TLP1	2.047
AT5G66730	C2H2-like zinc finger prote	2.047
AT2G35340	MEE29	2.046
AT2G20550	HSP40	2.046
AT2G34660	MRP2	2.046
AT3G12910	no apical meristem domai	2.046
AT4G11690	pentatricopeptide repeat-	2.046
AT3G25610	phospholipid-transporting	2.046
AT2G01460	P-loop containing nucleosi	2.044
AT1G58210	EMB1674	2.044
AT2G43620	chitinase-like protein	2.044
AT3G29400	EXO70E1	2.044
AT4G13830	J20	2.044
AT1G17270	peptide-O-fucosyltransfer	2.043
AT1G12280	LRR and NB-ARC domain-c	2.043
AT3G55450	PBL1	2.043
AT4G18700	CIPK12	2.043
AT4G02480	AAA-type ATPase family p	2.042
AT5G41410	BEL1	2.042
AT5G17600	RING-H2 finger protein AT	2.042
AT3G52850	VSR1	2.041
AT3G07650	COL9	2.041
AT4G24790	AAA-type ATPase family p	2.041
AT1G32100	PRR1	2.040
AT1G03740	protein kinase domain-cor	2.040
AT3G33530	transducin	2.039
AT3G23920	BAM1	2.039
AT4G16250	PHYD	2.039
AT5G41650	lactoylglutathione lyase fa	2.038
AT3G47890	Ubiquitin carboxyl-termin	2.038
AT1G29670	GDSL esterase	2.038
AT2G25170	PKL	2.038
AT2G46780	RNA-binding	2.038
AT2G46060	transmembrane protein-li	2.038
AT1G15490	alpha	2.038
AT1G07570	APK1A	2.038
AT2G01750	MAP70-3	2.037
AT4G03000	putative E3 ubiquitin-prot	2.037
AT2G41220	GLU2	2.037

AT3G05740	RECQI1		2.036
AT5G43930	transducin		2.036
AT1G21320	nucleic acid		2.036
AT3G18370	ATSYTF		2.036
AT1G11280	S-locus lectin protein kina:		2.035
AT3G62200	Putative endonuclease or		2.035
AT5G10180	SULTR2		2.035
AT3G50630	KRP2		2.035
AT1G52880	NAM		2.035
AT5G42400	SDG25		2.034
AT1G07530	SCL14		2.033
AT5G09630	LisH		2.033
AT5G05230	U-box domain-containing		2.033
AT2G32590	condensin complex subun		2.032
AT4G18240	SS4		2.032
AT2G13960	myb family transcription fa		2.032
AT1G65440	GTB1		2.032
AT4G12560	CPR30		2.032
AT1G63855	Putative methyltransferas		2.032
AT1G16330	CYCB3		2.031
AT2G47500	putative kinesin heavy cha		2.030
AT5G54270	LHCB3		2.030
AT1G07730	disease resistance-respons		2.030
AT5G04230	PAL3		2.030
AT2G45910	U-box domain-containing		2.029
AT3G54670	TTN8		2.029
AT3G49060	U-box domain-containing		2.029
AT1G07230	NPC1		2.029
AT4G23540	NUC173 domain-containir		2.028
AT4G22340	CDS2		2.028
AT5G05130	DNA		2.028
AT3G01770	BET10		2.027
AT3G56780	putative F-box		2.027
AT1G44900	MCM2		2.027
AT3G28880	Ankyrin repeat family prot		2.027
AT5G02830	pentatricopeptide repeat-		2.027
AT4G23730	glucose-6-phosphate 1-ep		2.027
AT5G65180	ENTH		2.026
AT1G11280	S-locus lectin protein kina:		2.026
AT1G25580	SOG1		2.026
AT2G05120	nucleoporin, Nup133		2.025
AT1G56610	RNI		2.025
AT5G40880	zinc finger CCCH domain-c		2.025
AT2G47920	kinase interacting-like pro		2.024
AT5G07580	ERF		2.024
AT1G31280		ago-02	2.024
AT3G08860	PYD4		2.024
AT2G23460	XLG1		2.024
AT1G56670	GDSL esterase		2.024
AT2G33380	RD20		2.024

AT3G54850	PUB14	2.024
AT3G12570	FYD	2.022
AT1G65580	FRA3	2.022
AT3G52670	FBD-associated F-box prot	2.022
AT2G30800	HVT1	2.021
AT5G08110	UBQ, helicase-c and DEAD	2.021
AT5G01450	RING	2.021
AT4G15560	CLA1	2.021
AT4G32650	KAT3	2.021
AT1G13960	WRKY4	2.021
AT1G75460	ATP-dependent protease l	2.020
AT3G61710	ATG6	2.020
AT3G28450	leucine-rich repeat proteir	2.020
AT4G37220	cold acclimation protein W	2.020
AT1G21640	NADK2	2.020
AT5G53050	hydrolase, alpha	2.020
AT3G10010	DML2	2.019
AT3G14205	Phosphoinositide phospha	2.019
AT3G26540	pentatricopeptide repeat-	2.019
AT1G67760	TCP-1	2.019
AT5G59600	pentatricopeptide repeat-	2.019
AT1G80320	2-oxoglutarate	2.018
AT1G76460	RNA recognition motif-cor	2.018
AT3G53180	glutamate-ammonia ligase	2.018
AT3G28860	ABCB19	2.018
AT2G37320	pentatricopeptide repeat-	2.018
AT5G52230	MBD13	2.017
AT4G19990	FRS1	2.017
AT4G10730	protein kinase-like proteir	2.017
AT2G41080	pentatricopeptide repeat-	2.016
AT5G33280	putative chloride channel-	2.016
AT5G22750	RAD5	2.015
AT5G27970	armadillo	2.015
AT5G58550	EOL2	2.015
AT2G16440	MCM4	2.014
AT5G50380	EXO70F1	2.014
AT5G57840	HXXXD-type acyl-transfera	2.014
AT1G11280	S-locus lectin protein kina:	2.013
AT5G28350	Quinoprotein amine dehy(	2.013
AT2G23320	WRKY15	2.012
AT5G16860	pentatricopeptide repeat-	2.012
AT4G34310	alpha	2.012
AT1G10340	ankyrin repeat-containing	2.012
AT5G46190	RNA-binding KH domain-c	2.011
AT5G42930	lipase class 3-like protein	2.011
AT3G59470	Far-red impaired responsi\	2.010
AT3G18970	MEF20	2.010
AT1G61660	transcription factor bHLH1	2.010
AT4G18550	lipase class 3 family protei	2.010
AT3G27400	pectate lyase	2.010

AT2G19240	RabGAP	2.010
AT3G04440	Plasma-membrane choline	2.009
AT1G63440	HMA5	2.009
AT1G76350	RWP-RK domain-containin	2.009
AT5G20150	SPX1	2.009
AT3G47340	ASN1	2.008
AT2G36200	kinesin family member 11	2.008
AT4G19510	TIR-NBS-LRR class disease	2.007
AT3G50590	transducin	2.007
AT4G21710	NRPB2	2.007
AT4G36270	ATP binding protein	2.007
AT5G15470	GAUT14	2.007
AT2G38830	Ubiquitin-conjugating enzy	2.007
AT3G18773	RING-H2 finger protein AT	2.006
AT2G24420	ATPase-related DNA repai	2.006
AT1G61820	BGLU46	2.006
AT3G47500	CDF3	2.006
AT1G63210	transcription elongation fa	2.004
AT5G18640	lipase class 3 family protei	2.004
AT1G53590	NTMC2T6.1	2.003
AT1G11280	S-locus lectin protein kinas	2.003
AT3G49510	F-box protein	2.003
AT2G04865	Aminotransferase-like, pla	2.002
AT3G22220	hAT dimerization domain-	2.002
AT3G05370	RLP31	2.001
AT4G14490	SMAD	2.001
AT1G21730	P-loop containing nucleosi	2.001
AT5G48400	ATGLR1.2	2.001
AT4G19500	P-loop NTPase and Toll	2.001
AT2G33835	FES1	2.001
AT2G38840	guanylate-binding-like pro	2.001
AT3G49880	glycosyl hydrolase family p	2.001
AT3G09260	PYK10	2.000
AT5G47000	peroxidase 65	2.000
AT3G53150	UGT73D1	2.000
AT5G20620	UBQ4	2.000
AT2G43690	concanavalin A-like lectin p	2.000
AT5G18230	CCR4-NOT transcription co	2.000
AT2G48110	REF4	2.000
AT3G02290	E3 ubiquitin-protein ligase	2.000
AT1G01660	zinc finger CCCH domain-c	2.000
AT4G27430	CIP7	2.000
AT4G24510	CER2	2.000
AT5G58780	dehydrodolichyl diphospha	2.000
AT3G01700	AGP11	2.000
AT4G36490	SFH12	2.000
AT3G46710	putative disease resistance	2.000
AT3G48300	CYP71A23	2.000
AT5G33355	defensin-like protein 207	2.000
AT5G31412	hAT family dimerization do	2.000

AT3G51360	aspartyl protease family p	2.000
