# Supplementary material for: Transcriptomic profiling of linolenic acid-responsive genes in ROS signaling from RNA-seq data in Arabidopsis
Source: Front Plant Sci. 2015 Mar 17;6:122. doi: 10.3389/fpls.2015.00122 (PMC4362301; doi:10.3389/fpls.2015.00122)
Supplement: Supplemental Table 1 — Fatty acid composition of Arabidopsis thaliana cell suspension cultures (ACSC). [file DataSheet1.ZIP › Table 1.PDF]

Table 1. Fatty acid composition of *Arabidopsis thaliana* cell suspension cultures (ACSC).

**Fatty acid**	**Percentage**
Palmitic acid (16:0)	17.85
Palmitoleic acid (16:1)	0.44
Stearic acid (18:0)	4.04
Oleic acid (18:1)	3.86
Linoleic acid (18:2)	19.97
Linolenic acid (18:3)	50.43
Others	3.42
