# Supplementary material for: Transcriptomic profiling of linolenic acid-responsive genes in ROS signaling from RNA-seq data in Arabidopsis
Source: Front Plant Sci. 2015 Mar 17;6:122. doi: 10.3389/fpls.2015.00122 (PMC4362301; doi:10.3389/fpls.2015.00122)
Supplement: Supplemental Table 1 — Fatty acid composition of Arabidopsis thaliana cell suspension cultures (ACSC). [file DataSheet1.ZIP › Table 2.pdf]

Supplemental table 2. Oxidative stress markers. (A) Malondialdehyde (MDA) content, (**B)** Hydrogen peroxide (H_2O_2) production and **(C)** protein carbonyls determination assayed in ACSC incubated with 1 mM of linolenic acid for 1 hour. Distilled water and methanol (vehicle) were used as controls.

	Control	Vehicle	Ln 1 mM
(A) pmol MDA x g^{-1} FW	3.8 ± 0.11	4.0 ± 0.07	4.1 ± 0.27
(B) µmol H_2O_2 x g^{-1} FW	6.3 ± 0.30	6.3 ± 0.27	7.0 ± 0.32
(C) nmol DNPH x g^{-1} FW	9.0 ± 0.3	8.93 ± 0.3	8.65 ± 0.3
