# Supplementary material for: Transcriptomic profiling of linolenic acid-responsive genes in ROS signaling from RNA-seq data in Arabidopsis
Source: Front Plant Sci. 2015 Mar 17;6:122. doi: 10.3389/fpls.2015.00122 (PMC4362301; doi:10.3389/fpls.2015.00122)
Supplement: Supplemental Table 1 — Fatty acid composition of Arabidopsis thaliana cell suspension cultures (ACSC). [file DataSheet1.ZIP › Table 3.PDF]

JA-related genes (Control vs Ln UP)

FC	ID	Jasmonic acid biosynthetic process
36,838	AT5G13220	**JAS1, JASMONATE-ASSOCIATED 1, JASMONATE-ZIM-DOMAIN PROTEIN 10, JAZ10, TIFY DOMAIN PROTEIN 9, TIFY9.**
36,680	AT5G05600	**2-oxoglutarate (2OG) and Fe(II)-dependent oxygenase superfamily protein.**
26,669	AT1G80840	**ATWRKY40, WRKY DNA-BINDING PROTEIN 40, WRKY40.**
25,665	AT1G72450	**JASMONATE-ZIM-DOMAIN PROTEIN 6, JAZ6, TIFY DOMAIN PROTEIN 11B, TIFY11B..**
24,812	AT1G17380	**JASMONATE-ZIM-DOMAIN PROTEIN 5, JAZ5, TIFY11A.**
24,266	AT1G44350	**IAA-LEUCINE RESISTANT (ILR)-LIKE GENE 6, ILL6.**
22,834	AT1G72520	**ARABIDOPSIS THALIANA LIPOXYGENASE 4, ATLOX4, LIPOXYGENASE 4, LOX4.**
13,449	AT3G25780	**ALLENE OXIDE CYCLASE 3, AOC3.**
12,956	AT5G13220	**JAS1, JASMONATE-ASSOCIATED 1, JASMONATE-ZIM-DOMAIN PROTEIN 10, JAZ10, TIFY DOMAIN PROTEIN 9, TIFY9.**
12,267	AT1G32640	**ATMYC2, JAI1, JASMONATE INSENSITIVE 1, JIN1, MYC2, RD22BP1, ZBF1.**
11,382	AT1G17420	**ARABIDOPSIS THALIANA LIPOXYGENASE 3, ATLOX3, LIPOXYGENASE 3, LOX3.**
9,711	AT5G42650	**ALLENE OXIDE SYNTHASE, AOS, CYP74A, CYTOCHROME P450 74A, DDE2, DELAYED DEHISCENCE 2.**
9,619	AT1G70700	**JASMONATE-ZIM-DOMAIN PROTEIN 9, JAZ9, TIFY7.**
9,223	AT3G23250	**ATMYB15, ATY19, MYB DOMAIN PROTEIN 15, MYB15.**
8,813	AT1G74950	**JASMONATE-ZIM-DOMAIN PROTEIN 2, JAZ2, TIFY10B.**
8,056	AT5G20230	**ATBCB, BCB, BLUE COPPER BINDING PROTEIN, BLUE-COPPER-BINDING PROTEIN, SAG14, SENESCENCE ASSOCIATED GENE 14.**
7,230	AT5G59820	**ATZAT12, RESPONSIVE TO HIGH LIGHT 41, RHL41, ZAT12.**
6,480	AT1G05800	**DGL, DONGLE.**
5,846	AT2G27690	**"CYTOCHROME P450, FAMILY 94, SUBFAMILY C, POLYPEPTIDE 1", CYP94C1.**
5,775	AT1G27730	**SALT TOLERANCE ZINC FINGER, STZ, ZAT10.**
5,603	AT2G06050	**ATOPR3, DDE1, DELAYED DEHISCENCE 1, OPR3, OXOPHYTODIENOATE-REDUCTASE 3.**
5,404	AT3G06490	**ATMYB108, BOS1, BOTRYTIS-SUSCEPTIBLE1, MYB DOMAIN PROTEIN 108, MYB108.**
5,084	AT1G01720	**ANAC002, ARABIDOPSIS NAC DOMAIN CONTAINING PROTEIN 2, ATAF1.**
4,724	AT1G20510	**OPC-8:0 COA LIGASE1, OPCL1.**
4,417	AT1G76650	**CALMODULIN-LIKE 38, CML38.**
3,846	AT3G09940	**ARABIDOPSIS THALIANA MONODEHYDROASCORBATE REDUCTASE 3, ATMDAR3, MDAR2, MDAR3, MDHAR, MONODEHYDROASCORBATE REDUCTASE, MONODEHYDROASCORBATE REDUCTASE 2, MONODEHYDROASCORBATE REDUCTASE 3.**
3,420	AT3G51450	**Calcium-dependent phosphotriesterase superfamily protein.**
3,373	AT4G39030	**EDS5, ENHANCED DISEASE SUSCEPTIBILITY 5, SALICYLIC ACID INDUCTION DEFICIENT 1, SCORD3, SID1, SUSCEPTIBLE TO CORONATINE-DEFICIENT PST DC3000 3.**

JA-related genes (Control vs Ln UP)

3,264	AT3G15500	**ANAC055, ATNAC3, NAC DOMAIN CONTAINING PROTEIN 3, NAC DOMAIN CONTAINING PROTEIN 55, NAC055, NAC3.**
3,091	AT5G53750	**CBS domain-containing protein.**
2,478	AT2G46510	**ABA-INDUCIBLE BHLH-TYPE TRANSCRIPTION FACTOR, AIB, ATAIB.**
2,335	AT4G31500	**"CYTOCHROME P450, FAMILY 83, SUBFAMILY B, POLYPEPTIDE 1", ALTERED TRYPTOPHAN REGULATION 4, ATR4, CYP83B1, RED ELONGATED 1, RED1, RNT1, RUNT 1, SUPERROOT 2, SUR2.**
		**Jasmonic acid mediated signaling pathway& Regulation JA mediated signaling pathway**
36,838	AT5G13220	**JAS1, JASMONATE-ASSOCIATED 1, JASMONATE-ZIM-DOMAIN PROTEIN 10, JAZ10, TIFY DOMAIN PROTEIN 9, TIFY9.**
36,680	AT5G05600	**2-oxoglutarate (2OG) and Fe(II)-dependent oxygenase superfamily protein.**
27,503	AT2G26530	**AR781.**
26,669	AT1G80840	**ATWRKY40, WRKY DNA-BINDING PROTEIN 40, WRKY40.**
25,665	AT1G72450	**JASMONATE-ZIM-DOMAIN PROTEIN 6, JAZ6, TIFY DOMAIN PROTEIN 11B, TIFY11B.**
24,812	AT1G17380	**JASMONATE-ZIM-DOMAIN PROTEIN 5, JAZ5, TIFY11A.**
14,502	AT1G06620	**Encodes a protein whose sequence is similar to a 2-oxoglutarate-dependent dioxygenase.**
12,956	AT5G13220	**JAS1, JASMONATE-ASSOCIATED 1, JASMONATE-ZIM-DOMAIN PROTEIN 10, JAZ10, TIFY DOMAIN PROTEIN 9, TIFY9.**
12,267	AT1G32640	**ATMYC2, JAI1, JASMONATE INSENSITIVE 1, JIN1, MYC2, RD22BP1, ZBF1.**
11,762	AT1G28370	**ATERF11, ERF DOMAIN PROTEIN 11, ERF11.**
9,711	AT5G42650	**ALLENE OXIDE SYNTHASE, AOS, CYP74A, CYTOCHROME P450 74A, DDE2, DELAYED DEHISCENCE 2.**
9,619	AT1G70700	**JASMONATE-ZIM-DOMAIN PROTEIN 9, JAZ9, TIFY7.**
9,223	AT3G23250	**ATMYB15, ATY19, MYB DOMAIN PROTEIN 15, MYB15.**
8,813	AT1G74950	**JASMONATE-ZIM-DOMAIN PROTEIN 2, JAZ2, TIFY10B.**
8,635	AT3G15210	**ATERF-4, ATERF4, ERF4, ETHYLENE RESPONSIVE ELEMENT BINDING FACTOR 4, RAP2.5, RELATED TO AP2 5.**
7,910	AT1G66090	**Disease resistance protein.**
5,846	AT2G27690	**"CYTOCHROME P450, FAMILY 94, SUBFAMILY C, POLYPEPTIDE 1", CYP94C1.**
5,839	AT5G47220	**ATERF-2, ATERF2, ERF2, ETHYLENE RESPONSE FACTOR- 2, ETHYLENE RESPONSIVE ELEMENT BINDING FACTOR 2.**
5,603	AT2G06050	**ATOPR3, DDE1, DELAYED DEHISCENCE 1, OPR3, OXOPHYTODIENOATE-REDUCTASE 3.**
5,404	AT3G06490	**ATMYB108, BOS1, BOTRYTIS-SUSCEPTIBLE1, MYB DOMAIN PROTEIN 108, MYB108.**
5,119	AT3G28210	**PMZ, SAP12, STRESS-ASSOCIATED PROTEIN 12.**
5,076	AT2G35930	**ATPUB23, PLANT U-BOX 23, PUB23.**
4,624	AT2G05940	**RIPK, RPM1-INDUCED PROTEIN KINASE.**
3,925	AT3G52400	**ATSYP122, SYNTAXIN OF PLANTS 122, SYP122.**
3,906	AT3G23240	**ATERF1, ERF1, ETHYLENE RESPONSE FACTOR 1.**

JA-related genes (Control vs Ln UP)

3,846	AT3G09940	**ARABIDOPSIS THALIANA MONODEHYDROASCORBATE REDUCTASE 3, ATMDAR3, MDAR2, MDAR3, MDHAR, MONODEHYDROASCORBATE REDUCTASE, MONODEHYDROASCORBATE REDUCTASE 2, MONODEHYDROASCORBATE REDUCTASE 3.**
3,637	AT1G02450	**NIM1-INTERACTING 1, NIMIN-1, NIMIN1.**
3,628	AT1G15520	**ABCG40, ARABIDOPSIS THALIANA ATP-BINDING CASSETTE G40, ATABCG40, ATP-BINDING CASSETTE G40, ATPDR12, PDR12, PLEIOTROPIC DRUG RESISTANCE 12.**
3,622	AT2G35980	**ARABIDOPSIS NDR1/HIN1-LIKE 10, ATNHL10, NDR1/HIN1-LIKE, NHL10, YELLOW-LEAF-SPECIFIC GENE 9, YLS9**.
3,459	AT5G67300	**ARABIDOPSIS THALIANA MYB DOMAIN PROTEIN 44, ATMYB44, ATMYBR1, MYB DOMAIN PROTEIN R1, MYB44, MYBR1**.
3,373	AT4G39030	**EDS5, ENHANCED DISEASE SUSCEPTIBILITY 5, SALICYLIC ACID INDUCTION DEFICIENT 1, SCORD3, SID1, SUSCEPTIBLE TO CORONATINE-DEFICIENT PST DC3000 3**.
3,297	AT1G52890	**ANAC019, NAC DOMAIN CONTAINING PROTEIN 19, NAC019**.
3,264	AT3G15500	**ANAC055, ATNAC3, NAC DOMAIN CONTAINING PROTEIN 3, NAC DOMAIN CONTAINING PROTEIN 55, NAC055, NAC3**.
3,193	AT4G39670	**Glycolipid transfer protein (GLTP) family protein**.
2,867	AT4G25390	**Protein kinase family protein**.
2,478	AT2G46510	**ABA-INDUCIBLE BHLH-TYPE TRANSCRIPTION FACTOR, AIB, ATAIB.**
2,266	AT3G11840	**PLANT U-BOX 24, PUB24**.
2,191	AT5G42050	**DCD (Development and Cell Death) domain protein**.
2,044	AT5G22570	**ARABIDOPSIS THALIANA WRKY DNA-BINDING PROTEIN 38, ATWRKY38, WRKY DNA-BINDING PROTEIN 38, WRKY38.**
		**Response to Jasmonic acid stimulus**
36,838	AT5G13220	**JAS1, JASMONATE-ASSOCIATED 1, JASMONATE-ZIM-DOMAIN PROTEIN 10, JAZ10, TIFY DOMAIN PROTEIN 9, TIFY9**.
36,680	AT5G05600	**2-oxoglutarate (2OG) and Fe(II)-dependent oxygenase superfamily protein.**
26,669	AT1G80840	**ATWRKY40, WRKY DNA-BINDING PROTEIN 40, WRKY40.**
25,665	AT1G72450	**JASMONATE-ZIM-DOMAIN PROTEIN 6, JAZ6, TIFY DOMAIN PROTEIN 11B, TIFY11B.**
24,812	AT1G17380	**JASMONATE-ZIM-DOMAIN PROTEIN 5, JAZ5, TIFY11A.**
24,266	AT1G44350	**IAA-LEUCINE RESISTANT (ILR)-LIKE GENE 6, ILL6.**
22,834	AT1G72520	**ARABIDOPSIS THALIANA LIPOXYGENASE 4, ATLOX4, LIPOXYGENASE 4, LOX4.**
16,292	AT5G54490	**PBP1, PINOID-BINDING PROTEIN 1.**
16,058	AT5G08790	**ANAC081, ARABIDOPSIS NAC DOMAIN CONTAINING PROTEIN 81, ATAF2**.
13,449	AT3G25780	**ALLENE OXIDE CYCLASE 3, AOC3**.
12,956	AT5G13220	**JAS1, JASMONATE-ASSOCIATED 1, JASMONATE-ZIM-DOMAIN PROTEIN 10, JAZ10, TIFY DOMAIN PROTEIN 9, TIFY9**.
12,267	AT1G32640	**ATMYC2, JAI1, JASMONATE INSENSITIVE 1, JIN1, MYC2, RD22BP1, ZBF1.**
11,762	AT1G28370	**ATERF11, ERF DOMAIN PROTEIN 11, ERF11.**
11,382	AT1G17420	**ARABIDOPSIS THALIANA LIPOXYGENASE 3, ATLOX3, LIPOXYGENASE**

JA-related genes (Control vs Ln UP)

		**3, LOX3.** LOX3 encode a Lipoxygenase. Lipoxygenases (LOXs) catalyze the oxygenation of fatty acids (FAs). Involved in: anther dehiscence, anther development, defense response, ethylene biosynthetic process, growth, jasmonic acid biosynthetic process, lipid oxidation, pollen development, response to chitin, response to fungus, response to high light intensity, response to jasmonic acid stimulus, response to wounding, stamen filament development.
9,711	AT5G42650	**ALLENE OXIDE SYNTHASE, AOS, CYP74A, CYTOCHROME P450 74A, DDE2, DELAYED DEHISCENCE 2.**
9,619	AT1G70700	**JASMONATE-ZIM-DOMAIN PROTEIN 9, JAZ9, TIFY7.**
9,223	AT3G23250	**ATMYB15, ATY19, MYB DOMAIN PROTEIN 15, MYB15**.
8,813	AT1G74950	**JASMONATE-ZIM-DOMAIN PROTEIN 2, JAZ2, TIFY10B**.
8,056	AT5G20230	**ATBCB, BCB, BLUE COPPER BINDING PROTEIN, BLUE-COPPER-BINDING PROTEIN, SAG14, SENESCENCE ASSOCIATED GENE 14**.
7,230	AT5G59820	**ATZAT12, RESPONSIVE TO HIGH LIGHT 41, RHL41, ZAT12**.
5,846	AT2G27690	**"CYTOCHROME P450, FAMILY 94, SUBFAMILY C, POLYPEPTIDE 1", CYP94C1**.
5,775	AT1G27730	**SALT TOLERANCE ZINC FINGER, STZ, ZAT10**.
5,603	AT2G06050	**ATOPR3, DDE1, DELAYED DEHISCENCE 1, OPR3, OXOPHYTODIENOATE-REDUCTASE 3**.
5,404	AT3G06490	**ATMYB108, BOS1, BOTRYTIS-SUSCEPTIBLE1, MYB DOMAIN PROTEIN 108, MYB108**.
5,390	AT1G74430	**ARABIDOPSIS THALIANA MYB DOMAIN CONTAINING PROTEIN 66, ARABIDOPSIS THALIANA MYB DOMAIN PROTEIN 95, ATMYB95, ATMYBCP66, MYB DOMAIN PROTEIN 95, MYB95**.
5,152	AT1G57560	**ATMYB50, MYB DOMAIN PROTEIN 50, MYB50**.
5,119	AT3G28210	**PMZ, SAP12, STRESS-ASSOCIATED PROTEIN 12**.
5,084	AT1G01720	**ANAC002, ARABIDOPSIS NAC DOMAIN CONTAINING PROTEIN 2, ATAF1**.
4,724	AT1G20510	**OPC-8:0 COA LIGASE1, OPCL1**.
4,680	AT5G63450	**"CYTOCHROME P450, FAMILY 94, SUBFAMILY B, POLYPEPTIDE 1", CYP94B1.**
4,417	AT1G76650	**CALMODULIN-LIKE 38, CML38**.
4,074	AT3G19580	**AZF2, ZF2, ZINC-FINGER PROTEIN 2**.
3,925	AT3G52400	**ATSYP122, SYNTAXIN OF PLANTS 122, SYP122.**
3,846	AT3G09940	**ARABIDOPSIS THALIANA MONODEHYDROASCORBATE REDUCTASE 3, ATMDAR3, MDAR2, MDAR3, MDHAR, MONODEHYDROASCORBATE REDUCTASE, MONODEHYDROASCORBATE REDUCTASE 2, MONODEHYDROASCORBATE REDUCTASE 3.**
3,813	AT4G33905	**Peroxisomal membrane 22 kDa (Mpv17/PMP22) family protein**.
3,784	AT1G21910	**DEHYDRATION RESPONSE ELEMENT-BINDING PROTEIN 26, DREB26**.
3,783	AT2G29450	**ARABIDOPSIS THALIANA GLUTATHIONE S-TRANSFERASE TAU 1, AT103-1A, ATGSTU1, ATGSTU5, GLUTATHIONE S-TRANSFERASE TAU 5, GSTU5.**
3,712	AT1G63840	**RING/U-box superfamily protein**.
3,680	AT4G37260	**ATMYB73, MYB DOMAIN PROTEIN 73, MYB73.**
3,628	AT1G15520	**ABCG40, ARABIDOPSIS THALIANA ATP-BINDING CASSETTE G40, ATABCG40, ATP-BINDING CASSETTE G40, ATPDR12, PDR12,**

JA-related genes (Control vs Ln UP)

		**PLEIOTROPIC DRUG RESISTANCE 12.**
3,541	AT1G74100	**ARABIDOPSIS SULFOTRANSFERASE 5A, ATSOT16, ATST5A, CORI-7, CORONATINE INDUCED-7, SOT16, SULFOTRANSFERASE 16.**
3,536	AT1G61890	**MATE efflux family protein.**
3,526	AT2G16720	**ARABIDOPSIS THALIANA MYB DOMAIN PROTEIN 7, ATMYB7, ATY49, MYB DOMAIN PROTEIN 7, MYB7.**
3,459	AT5G67300	**ARABIDOPSIS THALIANA MYB DOMAIN PROTEIN 44, ATMYB44, ATMYBR1, MYB DOMAIN PROTEIN R1, MYB44, MYBR1.**
3,425	AT3G28910	**ATMYB30, MYB DOMAIN PROTEIN 30, MYB30.**
3,420	AT3G51450	**Calcium-dependent phosphotriesterase superfamily protein.**
3,297	AT1G52890	**ANAC019, NAC DOMAIN CONTAINING PROTEIN 19, NAC019.**
3,264	AT3G15500	**ANAC055, ATNAC3, NAC DOMAIN CONTAINING PROTEIN 3, NAC DOMAIN CONTAINING PROTEIN 55, NAC055, NAC3.**
3,091	AT5G53750	**CBS domain-containing protein.**
2,975	AT1G61340	**ATFBS1, F-BOX STRESS INDUCED 1, FBS1.**
2,848	AT4G34990	**ATMYB32, MYB DOMAIN PROTEIN 32, MYB32.**
2,739	AT2G30040	**MAPKKK14, MITOGEN-ACTIVATED PROTEIN KINASE KINASE KINASE 14.**
2,539	AT4G27410	**ANAC072, ARABIDOPSIS NAC DOMAIN CONTAINING PROTEIN 72, RD26, RESPONSIVE TO DESICCATION 26.**
2,478	AT2G46510	**ABA-INDUCIBLE BHLH-TYPE TRANSCRIPTION FACTOR, AIB, ATAIB.**
2,316	AT4G38620	**ATMYB4, MYB DOMAIN PROTEIN 4, MYB4.**
2,213	AT4G11280	**1-AMINOCYCLOPROPANE-1-CARBOXYLIC ACID (ACC) SYNTHASE 6, ACS6, ATACS6.**
2,212	AT5G37260	**CIR1, CIRCADIAN 1, REVEILLE 2, RVE2.**
		**Jasmonic acid metabolic process**
25,665	AT1G72450	**JASMONATE-ZIM-DOMAIN PROTEIN 6, JAZ6, TIFY DOMAIN PROTEIN 11B, TIFY11B.**
24,266	AT1G44350	**IAA-LEUCINE RESISTANT (ILR)-LIKE GENE 6, ILL6.**
12,267	AT1G32640	**ATMYC2, JAI1, JASMONATE INSENSITIVE 1, JIN1, MYC2, RD22BP1, ZBF1.**
9,711	AT5G42650	**ALLENE OXIDE SYNTHASE, AOS, CYP74A, CYTOCHROME P450 74A, DDE2, DELAYED DEHISCENCE 2.**
9,619	AT1G70700	**JASMONATE-ZIM-DOMAIN PROTEIN 9, JAZ9, TIFY7.**
5,603	AT2G06050	**ATOPR3, DDE1, DELAYED DEHISCENCE 1, OPR3, OXOPHYTODIENOATE-REDUCTASE 3.**
5,390	AT1G74430	**ARABIDOPSIS THALIANA MYB DOMAIN CONTAINING PROTEIN 66, ARABIDOPSIS THALIANA MYB DOMAIN PROTEIN 95, ATMYB95, ATMYBCP66, MYB DOMAIN PROTEIN 95, MYB95.**
4,680	AT5G63450	**"CYTOCHROME P450, FAMILY 94, SUBFAMILY B, POLYPEPTIDE 1", CYP94B1.**
3,813	AT4G33905	**Peroxisomal membrane 22 kDa (Mpv17/PMP22) family protein.**
3,783	AT2G29450	**ARABIDOPSIS THALIANA GLUTATHIONE S-TRANSFERASE TAU 1, AT103-1A, ATGSTU1, ATGSTU5, GLUTATHIONE S-TRANSFERASE TAU 5, GSTU5.**
3,536	AT1G61890	**MATE efflux family protein.**
3,420	AT3G51450	**Calcium-dependent phosphotriesterase superfamily protein.**

JA-related genes (Control vs Ln DOWN)

FC	ID	Jasmonic acid biosynthetic process
10.681	AT2G22330	"CYTOCHROME P450, FAMILY 79, SUBFAMILY B, POLYPEPTIDE 3", CYP79B3.
5.144	AT3G22400	ARABIDOPSIS THALIANA LIPOXYGENASE 5, ATLOX5, LOX5.
3.518	AT5G38710	Methylenetetrahydrofolate reductase family protein.
3.231	AT1G55280	Lipase/lipooxygenase.
3.004	AT1G71697	ATCK1, CHOLINE KINASE, CHOLINE KINASE 1, CK, CK1.
2.702	ATCG01070	NDHE.
2.665	AT4G18950	Integrin-linked protein kinase family.
2.348	AT5G05140	Transcription elongation factor (TFIIS) family protein.
2.194	AT4G05160	Encodes a peroxisomal protein involved in the activation of fatty acids through esterification with CoA.
2.064	AT3G14050	AT-RSH2, ATRSH2, RELA-SPOT HOMOLOG 2, RELA/SPOT HOMOLOG 2, RSH2.
2.021	AT4G15560	1-DEOXY-D-XYLULOSE 5-PHOSPHATE (DXP) SYNTHASE 1, 1-DEOXY-D-XYLULOSE 5-PHOSPHATE SYNTHASE, 1-DEOXY-D-XYLULOSE 5-PHOSPHATE SYNTHASE 2, ATCLA1, CLA, CLA1, CLOROPLASTOS ALTERADOS 1, DEF, DXPS2, DXS, DXS1.
		**Jasmonic acid mediated signaling pathway& Regulation JA mediated signaling pathway**
6.989	AT5G14740	BETA CA2, BETA CARBONIC ANHYDRASE 2, CA18, CA2, CARBONIC ANHYDRASE 18, CARBONIC ANHYDRASE 2.
5.874	AT5G60410	ATSIZ1, SIZ1.
4.255	AT2G46370	ATGH3.11, FAR-RED INSENSITIVE 219, FIN219, JAR1, JASMONATE RESISTANT 1.
4.160	AT3G01500	ARABIDOPSIS THALIANA SALICYLIC ACID-BINDING PROTEIN 3, ATBCA1, ATSABP3, BETA CARBONIC ANHYDRASE 1, CA1, CARBONIC ANHYDRASE 1, SABP3, SALICYLIC ACID-BINDING PROTEIN 3.
4.090	AT2G31070	TCP DOMAIN PROTEIN 10, TCP10.
3.245	AT2G27060	Leucine-rich repeat protein kinase family protein.
3.518	AT5G38710	Methylenetetrahydrofolate reductase family protein.
3.420	AT3G63010	ATGID1B, GA INSENSITIVE DWARF1B, GID1B.
3.317	AT2G19190	FLG22-INDUCED RECEPTOR-LIKE KINASE 1, FRK1.
3.095	AT5G20480	EF-TU RECEPTOR, EFR.
3.053	AT1G53230	TCP3, TEOSINTE BRANCHED 1, CYCLOIDEA AND PCF TRANSCRIPTION FACTOR 3.
2.786	AT4G26090	RESISTANT TO P. SYRINGAE 2, RPS2.
2.786	AT1G07630	PLL5, POL-LIKE 5.
2.665	AT4G18950	Integrin-linked protein kinase family.
2.657	AT2G44490	BETA GLUCOSIDASE 26, BGLU26, PEN2, PENETRATION 2.
2.576	AT1G05010	ACO4, EAT1, EFE, ETHYLENE FORMING ENZYME, ETHYLENE-FORMING ENZYME.
2.572	AT1G59870	ABCG36, ARABIDOPSIS PLEIOTROPIC DRUG RESISTANCE 8, ARABIDOPSIS THALIANA ATP-BINDING CASSETTE G36, ATABCG36, ATP-BINDING CASSETTE G36, ATPDR8, PDR8, PEN3, PENETRATION

JA-related genes (Control vs Ln DOWN)

		**3, PLEIOTROPIC DRUG RESISTANCE 8.**
2.534	AT3G05660	**ATRLP33, RECEPTOR LIKE PROTEIN 33, RLP33.**
2.417	AT1G10210	**ATMPK1, MITOGEN-ACTIVATED PROTEIN KINASE 1, MPK1. I**
2.407	AT4G23810	**ATWRKY53, WRKY53.**
2.366	AT5G67340	**ARM repeat superfamily protein.**
2.253	AT5G01540	**L-TYPE LECTIN RECEPTOR KINASE-VI.2, LECRK-VI.2, LECRKA4.1, LECTIN RECEPTOR KINASE A4.1.**
2.175	AT1G64610	**Transducin/WD40 repeat-like superfamily protein.**
2.169	AT1G08050	**Zinc finger (C3HC4-type RING finger) family protein.**
2.155	AT5G27320	**ATGID1C, GA INSENSITIVE DWARF1C, GID1C.**
2.061	AT4G26080	**ABA INSENSITIVE 1, ABI1, ATABI1.**
2.020	AT1G75460	**ATP-dependent protease La (LON) domain protein.**
		**Response to JA stimulus**
11.125	AT5G46050	**ARABIDOPSIS THALIANA PEPTIDE TRANSPORTER 3, ATPTR3, PEPTIDE TRANSPORTER 3, PTR3.**
6.681	AT5G59780	**ATMYB59, ATMYB59-1, ATMYB59-2, ATMYB59-3, MYB DOMAIN PROTEIN 59, MYB59.**
5.425	AT2G36890	**ATMYB38, BIT1, BLUE INSENSITIVE TRAIT 1, MYB DOMAIN PROTEIN 38, MYB38, RAX2, REGULATOR OF AXILLARY MERISTEMS 2.**
4.255	AT2G46370	**ATGH3.11, FAR-RED INSENSITIVE 219, FIN219, JAR1, JASMONATE RESISTANT 1.**
3.933	AT3G46590	**ATTRP2, TRF-LIKE 1, TRFL1, TRP2.**
3.518	AT5G38710	**Methylenetetrahydrofolate reductase family protein.**
3.420	AT3G63010	**ATGID1B, GA INSENSITIVE DWARF1B, GID1B.**
3.066	AT2G31180	**ARABIDOPSIS THALIANA MYB DOMAIN PROTEIN 14, ATMYB14, MYB DOMAIN PROTEIN 14, MYB14, MYB14AT.**
3.004	AT1G71697	**ATCK1, CHOLINE KINASE, CHOLINE KINASE 1, CK, CK1.**
2.971	AT1G58220	**Homeodomain-like superfamily protein.**
2.836	AT4G15430	**ERD (early-responsive to dehydration stress) family protein.**
2.665	AT4G18950	**Integrin-linked protein kinase family.**
2.561	AT3G48360	**ATBT2, BT2, BTB AND TAZ DOMAIN PROTEIN 2.**
2.527	AT5G39610	**ANAC092, ARABIDOPSIS NAC DOMAIN CONTAINING PROTEIN 92, ATNAC2, ATNAC6, NAC DOMAIN CONTAINING PROTEIN 2, NAC DOMAIN CONTAINING PROTEIN 6, NAC2, NAC6, ORE1, ORESARA 1.**
2.348	AT5G05140	**Transcription elongation factor (TFIIS) family protein.**
2.316	AT5G13330	**RAP2.6L, RELATED TO AP2 6L.**
2.314	AT1G58200	**MSCS-LIKE 3, MSL3.**
2.207	AT5G45710	**AT-HSFA4C, HEAT SHOCK TRANSCRIPTION FACTOR A4C, HSFA4C, RHA1, ROOT HANDEDNESS 1.**
2.184	AT5G03280	**ATEIN2, CKR1, CYTOKININ RESISTANT 1, EIN2, ENHANCED RESPONSE TO ABA3, ERA3, ETHYLENE INSENSITIVE 2, ORE2, ORE3, ORESARA 2, ORESARA 3, PIR2.**
2.138	AT4G26850	**VITAMIN C DEFECTIVE 2, VTC2.**
2.135	AT5G61420	**ATMYB28, HAG1, HIGH ALIPHATIC GLUCOSINOLATE 1, MYB DOMAIN PROTEIN 28, MYB28, PMG1, PRODUCTION OF METHIONINE-DERIVED GLUCOSINOLATE 1.**
2.064	AT3G14050	**AT-RSH2, ATRSH2, RELA-SPOT HOMOLOG 2, RELA/SPOT HOMOLOG**

		**2, RSH2**.
2.061	AT4G26080	**ABA INSENSITIVE 1, ABI1, ATABI1**.
		**JA metabolic process**
4.255	AT2G46370	**ATGH3.11, FAR-RED INSENSITIVE 219, FIN219, JAR1, JASMONATE RESISTANT 1**.
2.434	AT4G37150	**ARABIDOPSIS THALIANA METHYL ESTERASE 9, ATMES9, MES9, METHYL ESTERASE 9.**
2.314	AT1G58200	**MSCS-LIKE 3, MSL3.**
